# Dihalohydration of Alkynols: A Versatile Approach to Diverse Halogenated Molecules

**DOI:** 10.1002/ejoc.201800668

**Published:** 2018-07-18

**Authors:** Samantha M. Gibson, Jarryl M. D'Oyley, Joe I. Higham, Kate Sanders, Victor Laserna, Abil E. Aliev, Tom D. Sheppard

**Affiliations:** ^1^ Christopher Ingold Laboratories University College London 20 Gordon St, London WC1H 0AJ UK

**Keywords:** Halogenation, Alkynes, Alcohols, Heterocycles, Ketones

## Abstract

In this paper we outline how dihalohydration reactions of propargylic alcohols can be used to access a wide variety of useful halogenated building blocks. A novel procedure for dibromohydration of alkynes has been developed, and a selection of dichloro and dibromo diols and cyclic ethers were synthesized. The dihalohydration of homo‐propargylic alcohols provides a useful route to 3‐halofurans, which were shown to readily undergo cycloaddition reactions under mild conditions. Finally, a novel ring expansion of propargylic alcohols containing a cyclopropylalkyne provides access to halogenated alkenylcyclobutanes.

## Introduction

Halogenated molecules play an important role in organic chemistry, both as synthetic targets themselves, and as useful reactants for a wide range of metal‐catalysed reactions. For example, chlorinated and brominated functional groups are widely present in agrochemicals^1^ and in flame retardants, although the latter are causing increasing environmental concerns.^2^ They are also widely used starting materials for a range of organometallic cross‐coupling methodologies.^3^ Geminal dihalides have found application as carbene or carbenoid precursors which undergo reaction with metals (e.g. Zn), metal salts (e.g. CrCl_2_), or organometallic reagents (e.g. Et_2_Zn) to initiate cyclopropanation or olefination reactions.^4^ Dihaloketones can also serve as useful precursors to enolates under reducing conditions.^5^ The synthesis of functionalized geminal dihalides is rare, however, as most halogenation processes require harsh conditions. For example, geminal dihalides can be generated from carbonyl groups using deoxohalogenation reagents such as PCl_5_, or via halogenation of the hydrazine or oxime derivatives.^6^ Alternative methods via double carbometallation of alkynes are also incompatible with functionalized substrates.^7^ We,^8^ and others,^9^, ^10^ have observed that dihalohydration of alkynes offers a mild and preparatively useful approach to α,α‐dihaloketones. These reactions usually proceed with high regioselectivity, and provide functionalized dihaloketones from readily available precursors. To date, however, the potentially interesting chemistry of these functionalized dihalides has not been explored in great detail.

**Scheme 1:**
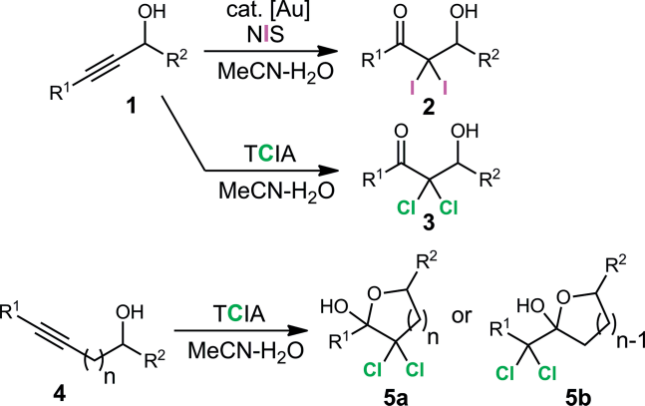
Previous work on the diiodohydration and dichlorohydration of alkynols.^8^

As part of our ongoing interest in the development of novel chemistry employing propargylic alcohols,^11^ we recently reported two methods for the dihalohydration of these systems **1** (Scheme 1), with a gold‐catalysed iodination reaction providing access to previously unreported α,α‐diiodo‐β‐hydroxyketones **2**, and a catalyst‐free procedure using trichloroisocyanuric acid (TCIA) giving α,α‐dichloro‐β‐hydroxyketones **3**. The latter procedure could also be extended to alkynols **4** giving access to dichlorolactols of general structure **5a** or **5b**.

In this article, we describe the extension of this work to the dibromohydration of alkynols, and we also describe preliminary studies on the application of geminal dibromides and dichlorides in further synthetic transformations to access a diverse range of potentially useful halogenated molecules, many of which constitute previously unreported structural frameworks.

## Results and Discussion

We set out to develop a method to convert readily accessible propargylic alcohols into the corresponding dibromohydroxyketones, whose properties remain relatively unexplored,^10^ although they have recently been demonstrated as effective bioisosteres for hydrated ketones in quorum‐sensing inhibitors.^12^ The required dibromohydration of propargylic alcohols was achieved efficiently using dibromoisocyanuric acid (DBIA)^13^ under closely related conditions to our previously described dichlorohydration reaction.^8^ Our initial experiments suggested that the reaction was more efficient with a higher proportion of water in the reaction medium (30 % rather than 10 % H_2_O in MeCN).

**Scheme 2:**
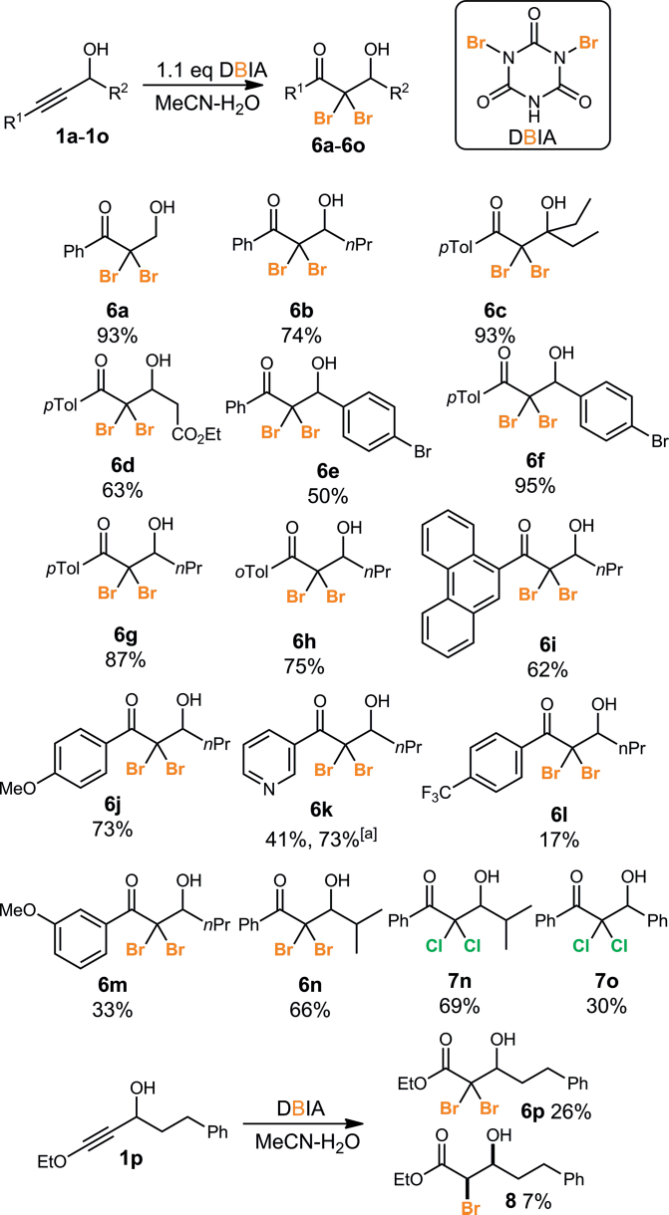
Dibromohydration of propargylic alcohols. ^[a]^ 5 equiv. of DBIA were used.

**Scheme 3:**
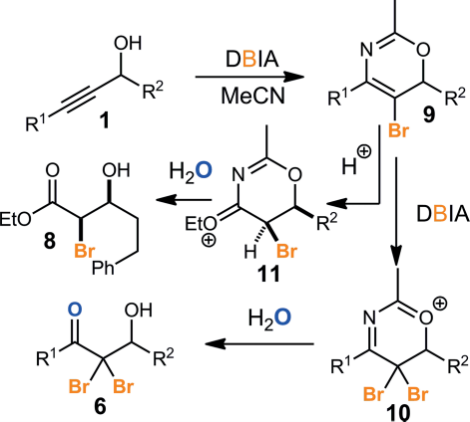
Proposed mechanism for dibromohydration.

Pleasingly, the reaction could be applied to primary, secondary and tertiary propargylic alcohols (Scheme 2, e.g. **6a**, **6b**, **6c**). As noted previously, an aryl group on the alkyne was necessary for efficient reaction. However, unsubstituted phenyl rings (**6a**, **6b**, **6e**, **6n**) and both electron rich (**6c**, **6d**, **6g**, **6h**, **6j**, **6m**) and electron deficient (**6l**, **6m**) aryl groups could be used, including examples bearing polyaromatic rings (**6i**) and a pyridine heterocycle (**6k**). A very electron‐deficient benzene ring (**6l**) led to a low yield of the desired dibromide, however. Both aryl and alkyl chains could be incorporated as the R^2^ substituent, including some functional groups (ester **6d**, aryl bromide **6e**–**6f**). The novel dichlorides **7n** and **7o** were also prepared using our previously reported dichlorohydration method. Interestingly, the dibromohydration of an ethoxyacetylene **1p** led to the formation of both the desired dibromide **6p** and the monobrominated compound **8**. The formation of this latter compound as a single diastereoisomer is consistent with our proposed mechanism in which the halogenation reaction proceeds via formation of heterocycle **9** through incorporation of a molecule of acetonitrile (Scheme 3). Heterocycle **9** can then undergo a second bromination to give dibromide **10** which then hydrolyses to yield the dibromoketone product **6**. Alternatively, protonation of highly electron rich heterocycle **9** (where R^1^ = OEt) may take place selectively on the least hindered side to give **11** which will yield *syn*‐bromohydrin **8** as the major diastereoisomer after hydrolysis.

**Scheme 4:**
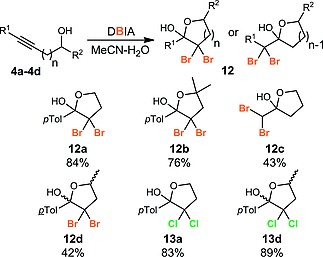
Dibromohydration and dichlorohydration of extended alkynols.

The bromination reaction could be extended to the synthesis of dibromolactols **12a**–**12d** through reaction of extended alkynol derivatives (Scheme 4), including compounds containing primary (**12a**, **12c**), secondary (**12d**) and tertiary (**12b**) alcohols. Dichlorides **13a** and **13d** were also prepared via dichlorohydration of the alkynes using TCIA.

**Scheme 5:**
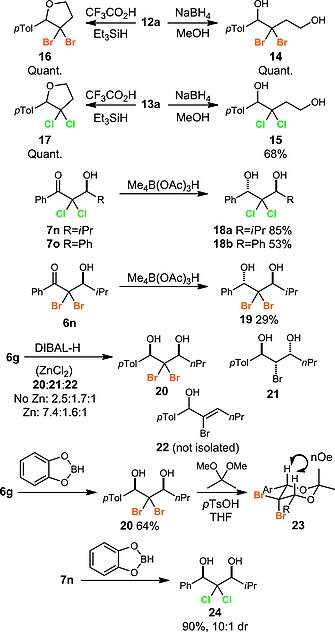
Reduction of geminal dihalides to access halogenated diols and cyclic ethers.

With a selection of dihalohydroxyketones and dihalolactols in hand, we examined their reduction to access halogenated diols and cyclic ethers. Pleasingly, lactols **12a** and **13a** could be converted into the corresponding dihalo‐1,4‐diols **14**/**15** or dihalogenated tetrahydrofurans **16**/**17** through treatment with NaBH_4_ or TFA/Et_3_SiH respectively. Dichlorohydroxyketones **7n** and **7o** could readily be converted with high diastereoselectivity into the *anti*‐dichlorodiols **18** via reduction with Me_4_NB(OAc)_3_H.^14^ The same procedure was also used to access *anti*‐bromodiol **19**. We next set out to investigate a complementary method to access the corresponding *syn*‐dihalodiols. Reduction of **6g** with DIBAL‐H in the presence or absence of ZnCl_2_ 15 led to the formation of a mixture of products including desired *syn*‐diol **20**, monobrominated *anti*‐diol **21**, and brominated allylic alcohol **22**, with the zinc chloride leading to an enhanced selectivity in favour of the desired diol **20**. An alternative reduction protocol using catechol borane^16^ gave a mixture of *syn* and *anti* diols in 82 % overall yield (82:18 *dr*), from which the desired *syn* diol **20** could be isolated in 64 % yield. The stereochemistry of **20** was confirmed through formation of the acetonide **23** which displayed an nOe between the two axial protons indicated. Using the same method, dichlorohydroxyketone **7n** was converted into the corresponding *syn*‐dichlorodiol **24** in 90 % yield. Interestingly, dibromo‐1,3‐diols such as **19** and **20** have never previously been synthesised (Scheme 5).^17^

**Scheme 6:**
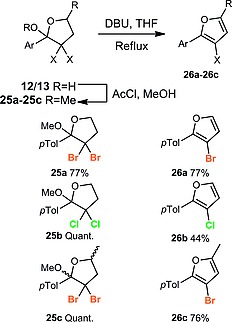
Synthesis of 3‐halofurans.

We envisaged that lactols such as **12a** could readily be converted into synthetically useful 3‐halofurans^18^ through formal elimination of water and HX. Attempts to convert **12a** directly into the 3‐bromofuran were unsuccessful. However, further investigation determined that 3‐halofurans could be successfully obtained through conversion of the lactols **12a**, **13a** and **13d** into the mixed ketal derivatives **25a**–**25c** with AcCl/MeOH, followed by basic elimination of methanol and HX. Using this sequence, we were able to access 3‐bromofuran **26a**, 3‐chlorofuran **26b** and trisubstituted bromofuran **26c**; with complete control over the substitution pattern in the latter compound. This approach is complementary to routes employing ynones,[18a,b,e] providing access to a different regioisomer of the 3‐halofuran (Scheme 6).

**Scheme 7:**
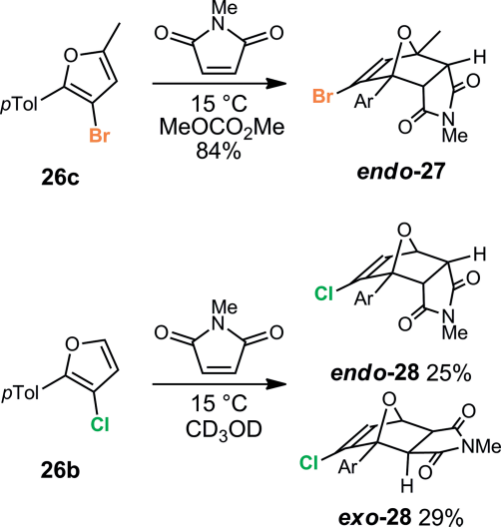
Cycloaddition reactions of 3‐halofurans.

We have previously reported that 3‐alkoxyfurans are readily able to undergo Diels–Alder reaction with maleimides to provide *endo*‐cantharimide derivatives, which are promising lead‐like molecules for medicinal chemistry.^19^ 3‐Halofurans have been reported to show enhanced reactivity in intramolecular Diels–Alder reactions,^20^, ^21^ so we were therefore interested in exploring their reactivity with maleimides to access useful halogenated cantharimide derivatives. 3‐Bromofuran **26c** gave the *endo* cantharimide **27** as a single diatereoisomer in excellent yield upon reaction with *N*‐methylmaleimide. However, 3‐chlorofuran **26b** gave a mixture of the separable *endo* and *exo* cantharimides **28** in moderate overall yield under similar conditions (Scheme 7).

**Scheme 8:**
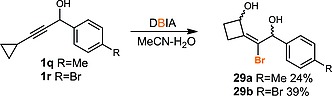
Synthesis of cyclobutanes via ring expansion of propargylic alcohols containing cyclopropylacetylenes.

A further interesting transformation was uncovered upon attempted dibromination of cyclopropyl‐containing propargylic alcohol **1q**, which yielded the vinylcyclobutane **29a** as the major product as a mixture of diastereoisomers. This reaction was extended to the synthesis of **29b**.^22^ Although ring expansion of cyclopropanes to cyclobutanes is precedented,^23^ the direct ring expansion of cyclopropylacetylenes has rarely been observed (Scheme 8).^24^

## Conclusions

We have reported a new method for the dibromohydration of alkynols to give dibromoketones that is applicable to a wide range of substrates, along with the extension of our previously reported dichlorohydration reaction to new substrates. The dihaloketones obtained from these reactions can be used to prepare structurally diverse halogenated molecules including dihalodiols, dihalogenated tetrahydrofurans, 3‐halofurans, and halogenated cantharimides. We have also discovered a novel halogenation/ring expansion of alkynylcyclopropanes that provides alkenylcyclobutanes. Many of these classes of halogenated compound have never previously been prepared. Further work is underway to optimize these methods, and to explore the interesting properties of these structurally unusual molecules.

## Experimental Section

Full experimental procedures for the preparation of all compounds, along with ^1^H and ^13^C spectra for all novel compounds can be found in the supporting information.

**General Procedure for Dibromohydration Reactions:** Dibromoisocyanuric acid (1.1 equiv.) was added to a stirring solution of alkynyl alcohol (1.0 equiv.) in MeCN/H_2_O (7:3, 2 mL mmol^–1^). The solution was stirred for 10 min after which solvent was removed in vacuo and the resultant residue purified by column chromatography (10 % EtOAc in petrol) to give the dibromohydroxyketone.

**General Procedure for Dichlorohydration Reactions:** Trichloroisocyanuric acid (1 equiv.) was added to a stirring solution of alkynyl alcohol (1 equiv.) in MeCN/H_2_O (10:1, 4 mL mmol^–1^). After 30 min the solvent was removed in vacuo and the residue was purified by column chromatography to give the dichlorohydroxyketone.

**2,2‐Dibromo‐3‐hydroxy‐1‐phenylpropan‐1‐one (6a):** 149 mg, 93 %, yellow oil. ^1^H NMR (600 MHz, CDCl_3_): *δ* = 8.40 (dd, *J *= 8.6, 1.2 Hz, 2 H, 2 × ArH), 7.61 (tt, *J *= 7.4, 1.2 Hz, 1 H, ArH), 7.49 (dd, *J *= 8.6, 7.4 Hz, 2 H, 2 × ArH), 4.35 (s, 2 H, CH_2_), 3.12 (br. s, 1 H, OH) ppm. ^13^C NMR (150 MHz, CDCl_3_): *δ* = 189.5, 134.3, 131.7, 131.4, 128.3, 72.1, 63.4 ppm. HRMS: Found (CI): [M + H]^+^ 306.89622, C_9_H_9_Br_2_O_2_: calcd. 306.89693. IR (film): ν̃_max_ = 3422 (O–H), 2918 (C–H), 1667 (C=O), 1446 (C=C) cm^–1^. The synthesis of this compound has been previously reported using a different method.[10b]

**2,2‐Dibromo‐3‐hydroxy‐1‐phenylhexan‐1‐one (6b):** 818 mg, 74 %, yellow solid, m.p. 54–55 °C. ^1^H NMR (CDCl_3_, 600 MHz): *δ* = 8.37 (m, 2 H, ArH), 7.59 (m, 1 H, ArH), 7.48 (m, 2 H, ArH), 4.23 (dd, *J *= 9.6, 1.4 Hz, 1 H, CH), 3.34 (br. s, 1 H, OH), 2.07 (m, 1 H, 1 × CHC*H*_2_), 1.80 (m, 1 H, 1 × CHC*H_2_*), 1.74 (m, 1 H, 1 × C*H*_2_CH_3_), 1.52 (m, 1 H, 1 × *CH*_2_CH_3_), 1.02 (t,* J *= 7.4 Hz, 3 H, CH_3_) ppm. ^13^C NMR (CDCl_3_, 150 MHz): *δ* = 190.4, 133.9, 132.6, 131.5, 128.1, 77.2, 71.4, 34.9, 19.5, 14.1 ppm. LRMS (NSI): *m/z* = 375 (49, [M + Na]^+^, 2 × ^81^Br), 373 (100, [M + Na]^+^, ^79^Br, ^81^Br), 371 (50, [M + Na]^+^, 2 × ^79^Br). HRMS Found 370.9252, C_12_H_14_Br_2_O_2_Na ([M + Na]^+^): calcd. 370.9253. IR (solid): ν̃_max_ = 3544 (O–H), 2955 (C–H), 2927 (C–H), 2864 (C–H), 1667 (C=O), 1594 (C=C), 1574 (C=C), 1445 cm^–1^.

**2,2‐Dibromo‐3‐ethyl‐3‐hydroxy‐1‐(*p*‐tolyl)pentan‐1‐one (6c):** 175 mg, 93 %, yellow oil. ^1^H NMR (CDCl_3_, 600 MHz): *δ* = 8.21 (d,* J *= 7.9 Hz, 2 H, ArH), 7.26 (d,* J *= 7.9 Hz, 2 H, ArH), 4.23 (br. s, 1 H, OH), 2.45 (s, 3 H, Ar*CH*_3_), 2.09 (q,* J *= 7.4 Hz, 4 H, 2 × *CH*_2_CH_3_), 1.09 (t,* J *= 7.4 Hz, 6 H, 2 × CH_2_ *CH*_3_) ppm. ^13^C NMR (CDCl_3_, 150 MHz): *δ* = 192.9, 144.6, 131.42, 131.38, 128.6, 80.8, 30.7, 21.8, 9.7 ppm. HRMS: Found 398.9553, C_14_H_19_Br_2_O_2_ ([M + H]^+^): calcd. 398.9566. IR (solid): ν̃_max_ = 3517 (O–H), 2969 (C–H), 2940 (C–H), 2879 (C–H), 1650 (C=O), 1603 (C=C), 1568 (C=C) cm^–1^.

**Ethyl 4,4‐Dibromo‐3‐hydroxy‐5‐oxo‐5‐(*p*‐tolyl)pentanoate (6d):** 128 mg, 63 %, pale yellow oil. ^1^H NMR (600 MHz, CDCl_3_): *δ* = 8.28 (d,* J *= 8.2 Hz, 2 H, 2 × ArH), 7.27 (d,* J *= 8.2 Hz, 2 H, 2 × ArH), 4.88 (dd, *J *= 9.6, 2.2 Hz, 1 H, *CH*OH), 4.23 (q,* J *= 7.2 Hz, 2 H, CH_2_CH_3_), 3.71 (br. s, 1 H, OH), 3.18 (dd, *J *= 16.0, 2.2 Hz, 1 H, CHOH*CH*H), 2.88 (dd, *J *= 16.0, 9.6 Hz, 1 H, CHOH*C*H*H*), 2.44 (s, 3 H, ArCH_3_), 1.30 (t,* J *= 7.2 Hz, 3 H, CH_2_ *CH*_3_) ppm. ^13^C NMR (150 MHz, CDCl_3_): *δ* = 189.2, 171.1, 145.4, 131.7, 129.4, 128.9, 74.4, 68.5, 61.2, 39.0, 21.9, 14.3 ppm. HRMS: Found (CI): [M + H]^+^ 406.94910, C_14_H_17_Br_2_O_4_: calcd. 406.94936. IR (film): ν̃_max_ = 3514 (O–H), 2980 (C–H), 1732 (C=O), 1666 (C=O) 1568 (Ar) cm^–1^.

**2,2‐Dibromo‐3‐(4‐bromophenyl)‐3‐hydroxy‐1‐phenylpropan‐1‐one (6e):** 101 mg, 50 %, pale brown solid, m.p. 157–159 °C. ^1^H NMR (CDCl_3_, 500 MHz): *δ* = 8.39 (d,* J *= 8.5 Hz, 2 H, ArH), 7.63–7.59 (m, 1 H, ArH), 7.56 (d,* J *= 8.6 Hz, 2 H, ArH), 7.53 (d,* J *= 8.6 Hz, 2 H, ArH), 7.50–7.46 (m, 2 H, ArH), 5.41 (d,* J *= 3.7 Hz, 1 H, CH), 3.96 (d,* J *= 3.7 Hz, 1 H, OH) ppm. ^13^C NMR (CDCl_3_, 125 MHz): *δ* = 190.9, 135.5, 134.1, 132.4, 131.9, 131.6, 130.6, 128.1, 123.2, 77.8, 69.1 ppm. LRMS (NSI) 489 (11 %, [M + Na]^+^, 3 × ^81^Br), 487 (32 %, [M + Na]^+^, 2 × ^81^Br, ^79^Br), 485 (35 %, [M + Na]^+^, 2 × ^79^Br, ^81^Br), 483 (10 %, [M + Na]^+^, 3 × ^79^Br), 345 (100 %). HRMS Found 482.8197, C_15_H_11_Br_3_O_2_Na ([M + Na]^+^): calcd. 482.8201. IR (solid): ν̃_max_ = 3530 (O–H), 2923 (C–H), 2853 (C–H), 1657 (C=O), 1591 (C=C), 1574 (C=C) cm^–1^.

**2,2‐Dibromo‐3‐(4‐bromophenyl)‐3‐hydroxy‐1‐(*p*‐tolyl)propan‐1‐one (6f):** 190 mg, 95 %, off white solid, m.p. 171–172 °C. ^1^H NMR (CDCl_3_, 600 MHz): *δ* = 8.32 (d,* J *= 8.4 Hz, 2 H, ArH), 7.56 (d,* J *= 8.5 Hz, 2 H, ArH), 7.53 (d,* J *= 8.5 Hz, 2 H, ArH), 7.28 (d,* J *= 8.4 Hz, 2 H, ArH), 5.40 (br. d, *J *= 2.4 Hz, 1 H, CH), 4.05 (br. d, *J *= 3.3 Hz, 1 H, OH), 2.45 (s, 3 H, CH_3_) ppm. ^13^C NMR (CDCl_3_, 150 MHz): *δ* = 190.6, 145.6, 135.6, 132.0, 131.9, 130.6, 129.6, 128.9, 123.3, 77.9, 69.2, 21.9 ppm. LRMS (CI) 480 (3 %, [M]^+^, 3 × ^81^Br), 478 (6 %, M^+^, 2 × ^81^Br, ^79^Br), 476 (7 %, [M]^+^, ^81^Br, 2 × ^79^Br), 474 (3 %, [M]^+^, 3 × ^79^Br), 413 (11 %), 400 (51 %, [M – Br]^+^, 2 × ^81^Br) 398 (100 %, [M – Br]^+^, ^81^Br, ^79^Br), 396 (54 %, [M – Br]^+^, 2 × ^79^Br), 383 (16 %), 381(31 %), 379 (15 %). HRMS Found 473.8459, C_16_H_13_Br_3_O_2_: calcd. 473.8466. IR (solid): ν̃_max_ = 3554 (O–H), 1657 (C=O), 1598 (C=C), 1297, 1215 cm^–1^.

**2,2‐Dibromo‐3‐hydroxy‐1‐(*p*‐tolyl)hexan‐1‐one (6 g):** 111 mg, 87 %, pale yellow solid, m.p. 71–73 °C. ^1^H NMR (CDCl_3_, 600 MHz): *δ* = 8.29 (d,* J *= 8.4 Hz, 2 H, ArH), 7.27 (d,* J *= 8.4 Hz, 2 H, ArH), 4.22 (dd, *J *= 9.6, 1.6 Hz, 1 H, CH), 3.46 (br. s, 1 H, OH), 2.44 (s, 3 H, Ar*CH*_3_), 2.12–2.02 (m, 1 H, 1 × CH*CH*_2_), 1.85–1.76 (m, 1 H, 1 × CH*CH*_2_), 1.76–1.69 (m, 1 H, 1 × *CH*_2_CH_3_), 1.59–1.47 (m, 1 H, 1 × *CH*_2_CH_3_), 1.02 (t,* J *= 7.4 Hz, 3 H, CH_2_ *CH*_3_) ppm. ^13^C NMR (CDCl_3_, 600 MHz): *δ* = 190.1, 145.2, 131.7, 129.8, 128.9, 77.2, 71.5, 34.9, 21.9, 19.6, 14.1 ppm. LRMS (ESI^+^) 389 (34 %, [M + Na^+^], 2 × ^81^Br), 387 (64 %, [M + Na^+^], ^79^Br, ^81^Br), 385 (31 %, [M + Na^+^], 2 × ^79^Br), 367 (34 %, [M + H]^+^, 2 × ^81^Br), 365 (100 %, ^79^Br, ^81^Br), 363 (48 %, 2 × ^79^Br). HRMS Found 362.9579, C_13_H_17_Br_2_O_2_: calcd. 362.9595. IR (film): ν̃_max_ = 3502 (O–H), 2958 (C–H), 2865 (C–H), 1658 (C=O), 1600, 1565 cm^–1^.

**2,2‐Dibromo‐3‐hydroxy‐1‐(*o*‐tolyl)hexan‐1‐one (6h):** 136 mg, 75 %, pale yellow oil, ^1^H NMR (CDCl_3_, 600 MHz): *δ* = 8.08–8.01 (m, 1 H, ArH), 7.38 (td, *J *= 7.6 and 1.2 Hz, 1 H, ArH), 7.29–7.26 (m, 1 H, ArH), 7.25–7.21 (m, 1 H, ArH), 4.36–4.27 (m, 1 H, *CH*OH), 2.77 (d,* J *= 6.1 Hz, 1 H, OH), 2.36 (s, 3 H, Ar*CH*_3_), 2.10–2.03 (m, 1 H, 1 × CH*CH*_2_), 1.79–1.65 (m, 2 H, 1 × CH*CH*_2_, 1 × *CH*_2_CH_3_), 1.57–1.46 (m, 1 H, 1 × *CH*_2_CH_3_), 1.02 (t,* J *= 7.3 Hz, 3 H, CH_3_) ppm. ^13^C NMR (CDCl_3_, 150 MHz): *δ* = 195.9, 137.6, 136.2, 131.3, 131.0, 128.1, 125.0, 76.9, 74.3, 35.4, 20.6, 19.4, 14.0 ppm. HRMS Found 362.9594, C_13_H_17_Br_2_O_2_: calcd. 362.9595. IR (film): ν̃_max_ = 3479 (O–H), 2958 (C–H), 2929 (C–H), 2871 (C–H), 1694 (C=O), 1600 (C=C), 1456 (C=C) cm^–1^.

**2,2‐Dibromo‐3‐hydroxy‐1‐(phenanthren‐9‐yl)hexan‐1‐one (6i):** 139 mg, 62 %, pale brown solid, m.p. 109–110 °C. ^1^H NMR (CDCl_3_, 600 MHz): *δ* = 8.74 (d,* J *= 8.3 Hz, 1 H, ArH), 8.71 (d,* J *= 8.3 Hz, 1 H, ArH), 7.99 (dd, *J *= 9.9, 1.0 Hz, 1 H, ArH), 7.91 (dd, *J *= 8.2, 1.0 Hz, 1 H, ArH), 7.76 (ddd, *J *= 8.3, 7.0, 1.4 Hz, 1 H, ArH), 7.71 (ddd, *J *= 8.3, 7.0, 1.3 Hz, 1 H, ArH), 7.69–7.65 (m, 1 H, ArH), 7.63 (ddd, *J *= 8.3, 7.0, 1.3 Hz, 1 H, ArH), 4.52–4.46 (m, 1 H, CH), 2.80 (d,* J *= 7.5 Hz, 1 H, OH), 1.85–1.71 (m, 2 H, *CH*_2_CH), 1.60–1.50 (m, 2 H, *CH*_2_CH_3_), 1.05 (t,* J *= 7.4 Hz, 3 H, CH_3_) ppm. ^13^C NMR (CDCl_3_, 150 MHz): *δ* = 195.9, 133.3, 131.2, 130.5, 130.0, 129.7, 129.3, 128.7, 127.8, 127.41, 127.39, 127.3, 126.4, 123.1, 122.8, 77.1, 75.0, 35.6, 19.4, 14.1 ppm. LRMS (EI) 452 (3 %, [M]^+^, 2 × ^81^Br), 450 (7 %, [M]^+^, ^79^Br, ^81^Br), 448 (4 %, [M]^+^, 2 × ^79^Br), 205 (100 %, [M – C_5_H_9_Br_2_O]^+^). HRMS Found 447.9647, C_20_H_18_Br_2_O_2_: calcd. 447.9668. IR (film): ν̃_max_ = 3572 (O–H), 2957 (C–H), 1642 (C=O), 1447 cm^–1^.

**2,2‐Dibromo‐3‐hydroxy‐1‐(4‐methoxyphenyl)hexan‐1‐one (6j):** 138 mg, 73 %, pale yellow solid, m.p. 81–84 °C. ^1^H NMR (CDCl_3_, 600 MHz): *δ* = 8.40 (d,* J *= 9.1 Hz, 2 H, ArH), 6.93 (d,* J *= 9.1 Hz, 2 H, ArH), 4.19 (ddd, *J *= 9.6, 4.2, 1.6 Hz, 1 H, CH), 3.90 (s, 3 H, OCH_3_), 3.52 (br. d, *J *= 4.2 Hz, 1 H, OH), 2.11–2.01 (m, 1 H, 1 × CH*CH*_2_), 1.85–1.77 (m, 1 H, 1 × CH*CH*_2_), 1.77–1.68 (m, 1 H, 1 × *CH*_2_CH_3_), 1.57–1.45 (m, 1 H, 1 × *CH*_2_CH_3_), 1.01 (t,* J *= 7.3 Hz, 3 H, CH_3_) ppm. ^13^C NMR (CDCl_3_, 150 MHz): *δ* = 189.0, 164.1, 134.2, 124.8, 113.3, 77.3, 71.4, 55.6, 34.8, 19.5, 14.0 ppm. LRMS (ES^+^) 405 (12 %, [M + Na]^+^, 2 × ^81^Br), 403 (26 %, [M + Na]^+^, ^79^Br, ^81^Br), 401 (11 %, [M + Na]^+^, 2 × ^79^Br), 383 (48 %, [M + H]^+^, 2 × ^81^Br), 381 (100 %, [M + H]^+^, ^79^Br, ^81^Br), 379 (50 %, [M + H]^+^, 2 × ^79^Br). HRMS Found 378.9539, C_13_H_17_Br_2_O_3_: calcd. 378.9544. IR (film): ν̃_max_ = 3511 (O–H), 2957 (C–H), 1656 (C=O), 1596, 1568 (C=C), 1510 (C=C) cm^–1^.

**2,2‐Dibromo‐3‐hydroxy‐1‐(pyridin‐3‐yl)hexan‐1‐one (6k):** Purified by column chromatography using 1 % MeOH in CH_2_Cl_2_: 72 mg, 41 %, orange gum (23 % SM recovered). ^1^H NMR (CDCl_3_, 500 MHz): *δ* = 9.53 (d,* J *= 1.4 Hz, 1 H, ArH), 8.78 (d,* J *= 3.6 Hz, 1 H, ArH), 8.62 (dt, *J *= 8.1, 2.0 Hz, 1 H, ArH), 7.42 (dd, *J *= 8.1, 4.8 Hz, 1 H, ArH), 4.23 (br. d, *J *= 9.4 Hz, 1 H, *CH*CH_2_), 3.29 (br. s, 1 H, OH), 2.11–1.97 (m, 1 H, 1 × CH*CH*_2_), 1.84–1.74 (m, 1 H, 1 × CH*CH*_2_), 1.73–1.67 (m, 1 H, 1 × *CH*_2_CH_3_), 1.60–1.44 (m, 1 H, 1 × *CH*_2_CH_3_), 1.02 (t,* J *= 7.3 Hz, 3 H, CH_3_) ppm. ^13^C NMR (CDCl_3_, 125 MHz): *δ* = 189.1, 153.6, 152.1, 138.6, 128.7, 122.9, 76.8, 71.2, 34.8, 19.4, 14.0 ppm. LRMS (ESI) 354 (49 %, [M + H]^+^, 2 × ^81^Br), 352 (100 %, [M + H]^+^, ^79^Br, ^81^Br), 350 (52 %, [M + H]^+^, 2 × ^79^Br). HRMS Found 349.9383, C_11_H_14_Br_2_NO_2_: calcd. 349.9386. IR (film): ν̃_max_ = 3113 (O–H), 2962 (C–H), 2927 (C–H), 2872 (C–H), 1681 (C=O), 1585 (C=C), 1417 cm^–1^.

**2,2‐Dibromo‐3‐hydroxy‐1‐[4‐(trifluoromethyl)phenyl]hexan‐1‐one (6l):** 35 mg, 17 %, yellow oil. ^1^H NMR (CDCl_3_, 500 MHz): *δ* = 8.44 (d,* J *= 8.3 Hz, 2 H, ArH), 7.73 (d,* J *= 8.3 Hz, 2 H, ArH), 4.23 (br. d, *J *= 9.3 Hz, 1 H, CH), 3.19 (br. s, 1 H, OH), 2.10–2.00 (m, 1 H, 1 × CH*CH*_2_), 1.82–1.74 (m, 1 H, 1 × CH*CH*_2_) 1.74–1.67 (m, 1 H, 1 × *CH*_2_CH_3_), 1.59–1.46 (m, 1 H, 1 × *CH*_2_CH_3_), 1.02 (t, *J *= 7.3 Hz, 3 H, CH_3_) ppm. ^13^C NMR (CDCl_3_ 125 MHz): *δ* = 189.4, 135.8, 134.7 (q, *J_C,F_* = 32.9 Hz), 131.6, 125.1 (q, *J_C,F_* = 3.7 Hz), 122.4 (q, *J_C,F_* = 273.0 Hz), 77.0, 71.2, 34.9, 19.4, 14.0 ppm. LRMS (EI) 401 (1 %, [M – H_2_O]^+^, 2 × ^81^Br), 399 (2 %, [M – H_2_O]^+^, ^79^Br, ^81^Br), 397 (1 %, [M – H_2_O]^+^, 2 × ^79^Br), 348 (11 %, [M – CF_3_]^+^, 2 × ^81^Br), 346 (24 %, [M – CF_3_]^+^, ^79^Br, ^81^Br), 344 (13 %, [M – CF_3_]^+^, 2 × ^79^Br), 230 (6 %), 228 (14 %), 226 (7 %) 173 (100 %, [M – C_5_H_9_Br_2_O]^+^). HRMS Found 433.9573, C_13_H_17_Br_2_F_3_NO_2_ ([M + NH_4_]^+^): calcd. 433.9573. IR (film): ν̃_max_ = 3562 (O–H), 2962 (C–H), 2933 (C–H), 2874 (C–H), 1678 (C=O), 1461 cm^–1^.

**2,2‐Dibromo‐3‐hydroxy‐1‐(3‐methoxyphenyl)hexan‐1‐one (6m):** 618 mg, 31 %, ^1^H NMR (CDCl_3_, 500 MHz): *δ* = 7.97 (dd, *J *= 7.9 Hz, 2.4 1 H, Ar), 7.81 (dd, *J *= 2.4, 1.7 Hz, 1 H, Ar), 7.34 (t,* J *= 8.0 Hz, 1 H, Ar), 7.10 (dd, *J *= 8.2 Hz, 2.5 1 H, Ar), 4.22 (d,* J *= 9.1 Hz, 1 H, *CH*OH), 3.84 (s, 3 H, OCH_3_), 3.39 (br. s, 1 H, OH), 2.04 (m, 1 H, C*H*_2_CH),1.73 (m, 2 H, C*H*_2_CH and C*H*_2_CH_3_), 1.51 (m, 1 H, C*H*_2_CH_3_),1.00 (t,* J *= 7.4 Hz, 3 H, *CH*_3_) ppm. ^13^C NMR (CDCl_3_ 125 MHz): *δ* = 190.1, 159.1, 133.8, 129.0, 123.9, 121.0, 116.0, 77.1, 71.7, 55.5, 34.9, 19.5, 14.0 ppm. LRMS (CI) 380.9 ([M + H]^+^, 40), 362.9 ([M – H_2_O]^+^, 24), 308.8 (20), 283.0 (100), 221.1 (12), 205.1 (11), 135.0 (30). HRMS Found 378.9540 C_13_H_17_Br_2_O_3_ [M + H]^+^: calcd. 378.9539.

**2,2‐Dibromo‐3‐hydroxy‐4‐methyl‐1‐phenylpentan‐1‐one (6n):** Yield: 665 mg, 66 %; m.p. 62–65 °C. ^1^H NMR (600 MHz, CDCl_3_): *δ* = 8.30 (dd, *J *= 8.4, 1.1 Hz, 2 H, ArH), 7.58 (t,* J *= 7.4 Hz, 1 H, ArH), 7.46 (t,* J *= 7.9 Hz, 2 H, ArH), 4.21 (dd, *J *= 5.8, 3.5 Hz, 1 H, *CH*OH), 3.18 (d,* J *= 6.0 Hz, 1 H, OH), 2.40 [heptd, *J *= 6.8, 3.5 Hz, 1 H, *CH*(CH_3_)_2_], 1.17 [d, *J *= 6.8 Hz, 3 H, CH(*CH*_3_)_2_], 1.12 [d, *J *= 7.0 Hz, 3 H, CH(*CH*_3_)_2_] ppm. ^13^C NMR (151 MHz, CDCl_3_): *δ* = 190.7, 133.6, 133.0, 131.2, 128.1, 80.1, 71.8, 31.9, 23.2, 17.9 ppm. LRMS (CI) 366 (51 %, [M + NH_4_]^+^, 2 × ^79^Br), 368 (100 %, [M + NH_4_]^+^, ^79^Br, ^81^Br); 370 (49 %, [M + NH_4_]^+^, 2 × ^81^Br). HRMS (CI^+^) found 365.96978 C_12_H_14_Br_2_O_2_NH_4_: calcd. 265.96988 ([M + NH_4_]^+^); ν̃_max_ = (film) 3539 (O–H), 2964 (C–H), 2932 (C–H), 2874 (C–H), 1671 (C=O), 1235 (C=C), 691 cm^–1^ (C–Br).

**Ethyl 2,2‐Dibromo‐3‐hydroxy‐5‐phenylpentanoate (6p):** Dibromoisocyanuric acid (0.6 equiv.) was added to a stirring solution of propargylic alcohol (1.0 equiv.) in MeCN/H_2_O (7:3, 2 mL mmol^–1^). The solution was stirred for 10 min after which solvent was removed in vacuo and the resultant residue purified by column chromatography (10 % EtOAc in petrol) to give **6p** as a colourless oil (49 mg, 26 %). ^1^H NMR (CDCl_3_, 600 MHz): *δ* = 7.35–7.27 (m, 2 H, ArH), 7.26–7.17 (m, 3 H, ArH), 4.34 (q,* J *= 7.1 Hz, 2 H, *CH*_2_CH_3_), 4.05 (dd, *J *= 9.9, 3.1 Hz, 1 H, CH), 3.09–2.94 (m, 2 H, OH, 1 × CH*CH*_2_), 2.77 (ddd, *J *= 13.8, 9.0, 7.8 Hz, 1 H, 1 × CH*CH*_2_), 2.35–2.23 (m, 1 H, 1 × *CH*_2_Ar), 2.08–1.95 (m, 1 H, 1 × *CH*_2_Ar), 1.34 (t,* J *= 7.1 Hz, 3 H, CH_3_) ppm. ^13^C NMR (CDCl_3_, 150 MHz): *δ* = 166.6, 141.3, 128.7, 128.6, 126.3, 7.8, 66.2, 64.2, 34.6, 32.1, 13.8 ppm. LRMS (CI) 400 (51 %, [M + NH_4_]^+^, 2 × ^81^Br), 398 (100 %, [M + NH_4_]^+^, ^79^Br, ^81^Br), 396 (50 %, [M + NH_4_]^+^, 2 × ^79^Br). HRMS Found 395.9805, C_13_H_20_Br_2_NO_3_ ([M + NH_4_]^+^): calcd. 395.9804. IR (film): ν̃_max_ = 3283 (O–H), 3027 (C–H), 2931 (C–H), 1727 (C=O), 1623, 1536 (C=C), 1496 (C=C), 1453 cm^–1^.

**2,2‐Dichloro‐3‐hydroxy‐4‐methyl‐1‐phenylpentan‐1‐one (7n):** Purified via column chromatography (20 % EtOAc in petrol) to give **7n** as a colourless oil which crystallises on standing (6.621 g, 69 %); m.p. 38–44 °C. ^1^H NMR (CDCl_3_, 400 MHz): *δ* = 8.24–8.22 (m, 2 H, ArH), 7.61–7.59 (m, 1 H, ArH), 7.48–7.46 (m, 2 H, ArH), 4.32 (dd, *J *= 5.6, 3.6 Hz, 1 H, *CH*OH), 3.03 (d,* J *= 6.0 Hz, 1 H, OH), 2.38 [heptd, *J *= 6.9, 3.6 Hz, 1 H, *CH*(CH_3_)_2_], 1.15 [d, *J *= 6.8 Hz, 3 H, CH(*CH*_3_)_2_], 1.13 [d, *J *= 7.0 Hz, 3 H, CH(*CH*_3_)_2_] ppm. ^13^C NMR (CDCl_3_, 176 MHz): *δ* = 190.5, 133.8, 132.3, 131.1, 128.3, 88.9, 79.6, 30.1, 22.8, 17.5 ppm. LRMS (ES^+^) 261 (100 %, [M]^+^, 2 × ^35^Cl), 263 (60 %, [M]^+^, ^35^Cl, ^37^Cl), 265 (10 %, [M]^+^, 2 × ^37^Cl). HRMS (ES^+^) found 261.0448, C_12_H_15_Cl_2_O_2_^+^: calcd. 261.0449 ([M]^+^). IR (film): ν̃_max_ = 3546 (O–H), 2962 (C–H), 2930 (C–H), 2872 (C–H), 1678 (C=O), 1235, 821, 691 cm^–1^.

**2,2‐Dichloro‐3‐hydroxy‐1,3‐diphenylpropan‐1‐one (7o):** Trichloroisocyanuric acid (1 equiv.) was added to a stirring solution of propargylic alcohol (1 equiv.) in MeCN/H_2_O (7:3 4 mL. mmol^–1^). The solution was stirred until the reaction was finished (as determined by TLC). the solvent was removed in vacuo and the residue was purified via column chromatography (20 % EtOAc in petrol) which afforded chlorinated hydroxykeytone **7o** as a yellow solid (632 mg; 30 %; m.p. 121–128 °C). ^1^H NMR (600 MHz, CDCl_3_): *δ* = 8.30 (dd, *J *= 8.5, 1.1 Hz, 2 H, ArH), 7.63–7.61 (m, 3 H, ArH), 7.49 (t,* J *= 7.9 Hz, 2 H, ArH), 7.41–7.40 (m, 3 H, ArH), 5.59 (d,* J *= 4.2 Hz, 1 H, *CH*OH), 3.75 (d,* J *= 4.2 Hz, 1 H, OH) ppm. ^13^C NMR (151 MHz, CDCl_3_): *δ* = 190.9, 135.7, 134.2, 132.0, 131.40, 129.90, 129.0, 128.3, 127.7, 86.7, 78.1 ppm. LRMS (NSI) 317 (100 %, [M + Na]^+^, 2 × ^35^Cl), 319 (60 %, M + Na)^+^, ^35^Cl, ^37^Cl, 321 (10 %, [M + Na]^+^, 2 × ^37^Cl). HRMS Found 317.0107, C_15_H_12_Cl_2_O_2_Na: calcd. 317.0109 ([M + Na]^+^). IR (film): ν̃_max_ = 3539 (O–H), 3060 (C–H), 3031 (C–H), 2917 (C–H), 1677 (C=O), 1239, 693 cm^–1^.

**Ethyl 2‐Bromo‐3‐hydroxy‐5‐phenylpentanoate (8):** Isolated from the reaction used to synthesise **6p** (10 mg, 7 %); colourless oil. ^1^H NMR (CDCl_3_, 600 MHz): *δ* = 7.32–7.28 (m, 2 H, ArH), 7.23–7.19 (m, 3 H, ArH), 4.30–4.18 (m, 3 H, CHBr, *CH*_2_CH_3_), 3.90 (dq, *J *= 7.9, 3.6 Hz, 1 H, *CH*OH), 2.94 (d,* J *= 3.6 Hz, 1 H, OH), 2.86 (ddd, *J *= 14.3, 9.4, 5.3 Hz, 1 H, 1 × CH*CH*_2_), 2.72 (ddd, *J *= 13.8, 9.4, 7.8 Hz, 1 H, 1 × CH*CH*_2_), 2.02–1.92 (m, 1 H, 1 × *CH*_2_Ar), 1.81–1.76 (m, 1 H, 1 × *CH*_2_Ar), 1.29 (t,* J *= 7.1 Hz, 3 H, CH_3_) ppm. ^13^C NMR (CDCl_3_, 150 MHz): *δ* = 169.5, 141.3, 128.62, 128.61, 126.2, 70.0, 62.6, 52.2, 35.9, 31.7, 14.0 ppm. LRMS (CI) 320 (98 %, [M + H]^+^, ^81^Br), 318 (100 %, [M + H]^+^, ^79^Br). HRMS Found 318.0699, C_13_H_18_BrO_3_: calcd. 318.0699. IR (film): ν̃_max_ = 3524 (O–H), 3026 (C–H), 2982 (C–H), 2934 (C–H), 1736 (C=O), 1495 (C=C), 1454 (C=C) cm^–1^. The *anti* diastereoisomer of this compound has previously been reported in the literature.^25^

**3,3‐Dibromo‐2‐(*p*‐tolyl)tetrahydrofuran‐2‐ol (12a):** 3.55 g, 84 %, white solid, m.p. 116–117 °C. ^1^H NMR (CDCl_3_, 500 MHz): *δ* = 8.27 (d,* J *= 8.4 Hz, 2 H, minor ArH), 7.71 (d,* J *= 8.2 Hz, 2 H, major ArH), 7.26 (d,* J *= 8.4 Hz, 2 H, minor ArH), 7.21 (d,* J *= 8.1 Hz, 2 H, major ArH), 4.33–4.25 (m, 1 H, major 1 × OCH_2_), 4.21–4.14 (m, 1 H, major 1 × OCH_2_), 4.01 (t,* J *= 6.2 Hz, 2 H, minor *CH*_2_OH), 3.46 (dt, *J *= 13.5, 9.2 Hz, 1 H, major 1 × CH_2_CBr_2_), 3.11–3.02 (m, 1 H, major 1 × CH_2_CBr_2_; m, 2 H, minor CH_2_CBr_2_), 2.44 (s, 3 H, minor CH_3_), 2.39 (s, 3 H, major CH_3_) ppm. ^13^C NMR (CDCl_3_, 125 MHz): *δ* = 188.3, 144.8, 139.4, 135.4, 131.5, 129.3, 128.8, 128.4, 127.4, 108.6, 105.9, 68.0, 65.2, 61.6, 49.8, 47.1, 21.8, 21.3 ppm. LRMS (EI) 338 (4 %, [M]^+^, 2 × ^81^Br), 336 (9 %, [M]^+^, ^79^Br, ^81^Br), 334 (5 %, [M]^+^, 2 × ^79^Br), 136 (82 %), 119 (100 %). HRMS Found 333.9200 C_11_H_12_Br_2_O_2_: calcd. 333.9204. IR (film): ν̃_max_ = 3409 (O–H), 2971 (C–H), 2908 (C–H), 1401 cm^–1^.

**3,3‐Dibromo‐5,5‐dimethyl‐2‐(*p*‐tolyl)tetrahydrofuran‐2‐ol (12b):** Yellow solid, 76 %; m.p. 65–67 °C. ^1^H NMR (600 MHz, CDCl_3_): *δ* = 7.71 (d,* J *= 8.2 Hz, 2 H, 2 × ArH), 7.20 (d,* J *= 8.2 Hz, 2 H, 2 × ArH), 3.41 (d,* J *= 14.1 Hz, 1 H, C*H*H), 3.18 (d,* J *= 14.1 Hz, 1 H, CH*H*), 2.97 (s, 1 H, OH), 2.38 (s, 3 H, Ar*CH*_3_), 1.62 (s, 3 H, CH_3_), 1.52 (s, 3 H, CH_3_) ppm. ^13^C NMR (150 MHz, CDCl_3_): *δ* = 139.3, 136.3, 128.4, 127.2, 106.8, 81.5, 68.2, 58.8, 32.6, 28.7, 21.4 ppm. HRMS: Found (CI): [M]^+^ 361.95116, C_13_H_16_Br_2_O_2_: calcd. 361.95170. IR (film): ν̃_max_ = 3519 (O–H), 2970 (C–H) cm^–1^.

**2‐(Dibromomethyl)tetrahydrofuran‐2‐ol (12c):** White crystals, 43 %; m.p. 70–71 °C. ^1^H NMR (600 MHz, CDCl_3_): *δ* = 5.72 (s, 1 H, CH), 4.17–4.23 (m, 1 H, OC*H*H), 4.00–4.07 (m, 1 H, OCH*H*), 3.05 (br. s, 1 H, OH), 2.19–2.29 (m, 3 H, OCH_2_ *CH*_2_C*H*H), 2.01–2.10 (m, 1 H, OCH_2_CH_2_CH*H*) ppm. ^13^C NMR (150 MHz, CDCl_3_): *δ* = 105.6, 70.4, 51.6, 35.2, 25.6 ppm. HRMS: Found (ES): [M – H]^+^ 256.8821, C_5_H_7_Br_2_O_2_: calcd. 256.8813. IR (film): ν̃_max_ = 3354 (O–H), 3014 (C–H) cm^–1^.

**3,3‐Dibromo‐5‐methyl‐2‐(*p*‐tolyl)tetrahydrofuran‐2‐ol (12d):** Isolated as a mixture of diastereomers (1:2.5); 294 mg, 42 %, white solid, m.p. 98–99 °C. ^1^H NMR (CDCl_3_, 600 MHz): *δ* = 7.71 (d,* J *= 8.2 Hz, 2 H, minor ArH), 7.68 (d,* J *= 8.2 Hz, 2 H, major ArH), 7.21 (d,* J *= 8.2 Hz, 2 H, major ArH), 7.19 (d,* J *= 8.2 Hz, 2 H, minor ArH), 4.65 (dp, *J *= 9.3, 6.0 Hz, 1 H, major CH), 4.29 (dqd, *J *= 9.8, 6.5, 3.4 Hz, 1 H, minor CH), 3.74 (s, 1 H, major OH), 3.55 (dd, *J *= 14.1, 9.8 Hz, 1 H, minor 1 × CH_2_), 3.49 (s, 1 H, minor OH), 3.16 (dd, *J *= 13.3, 6.0 Hz, 1 H, major 1 × CH_2_), 3.10 (dd, *J *= 13.3, 9.3 Hz, 1 H, major 1 × CH_2_), 2.93 (dd, *J *= 14.1, 3.4 Hz, 1 H, minor 1 × CH_2_), 2.40 (s, minor ArCH_3_), 1.49 (d,* J *= 6.5 Hz, 3 H, minor CH*CH*_3_), 1.45 (d,* J *= 6.0 Hz, 3 H, major CH*CH*_3_) ppm. ^13^C NMR (CDCl_3_, 150 MHz): *δ* = 139.3, 136.0, 135.4, 131.6, 128.4, 128.4, 127.5, 127.5, 106.4, 106.1, 74.7, 72.1, 68.8, 67.3, 54.4, 53.4, 22.4, 21.5, 21.4, 21.1 ppm. LRMS (CI) 370 (8 %, [M + NH_4_]^+^, 2 × ^81^Br), 368 (20 %, [M + NH_4_]^+^, ^79^Br, ^81^Br), 366 (10 %, [M + NH_4_]^+^, 2 × ^79^Br), 335 (51 %, [M – CH_3_]^+^, 2 × ^81^Br), 333 (100 %, [M – CH_3_]^+^, ^79^Br, ^81^Br), 331 (48 %, [M – CH_3_]^+^, 2 × ^79^Br). HRMS Found 365.9698, C12H18Br2NO2 ([M + NH_4_]^+^): calcd. 365.9699. IR (film): ν̃_max_ = 3342 (O–H), 2963 (C–H), 1913, 1510 (C=C) cm^–1^.

**3,3‐Dichloro‐2‐(*p*‐tolyl)tetrahydrofuran‐2‐ol (13a):** Isolated as a mixture of isomers (1:8.3); 1.83 g, 83 %, white solid, m.p. 122–124 °C. ^1^H NMR (CDCl_3_, 500 MHz): *δ* = 8.22 (d,* J *= 8.4 Hz, 2 H, minor ArH), 7.65 (d,* J *= 8.2 Hz, 2 H, major ArH), 7.30 (d,* J *= 8.4 Hz, 2 H, minor ArH), 7.22 (d,* J *= 8.2 Hz, 2 H, major ArH), 4.24 (ddd, *J *= 9.2, 8.3, 7.1 Hz, 1 H, major 1 × OCH_2_), 4.15 (ddd, *J *= 9.4, 8.3, 2.2 Hz, 1 H, major 1 × OCH_2_), 3.99 (t,* J *= 6.3 Hz, 2 H, minor CH_2_OH), 3.50 (br. s, 1 H, major OH), 3.20 (dt, *J *= 13.1, 9.3 Hz, 1 H, major 1 × CH_2_CCl_2_), 2.91 (ddd, *J *= 13.1, 7.1, 2.2 Hz, 1 H, major 1 × CH_2_CCl_2_), 2.84 (t,* J *= 6.3 Hz, 2 H, minor CH_2_CCl_2_), 2.47 (s, 3 H, minor CH_3_), 2.41 (s, 3 H, major CH_3_) ppm. ^13^C NMR (CDCl_3_, 125 MHz): *δ* = 188.2, 145.0, 139.3, 134.4, 131.4, 129.0, 128.8, 128.5, 127.4, 105.9, 91.1, 85.4, 64.5, 59.3, 47.4, 44.4, 21.8, 21.3 ppm. LRMS (EI) 250 (12 %, M^+^, 2 × ^37^Cl), 248 (67 %, M^+^, ^35^Cl, ^37^Cl), 246 (100 %, M^+^, 2 × ^35^Cl), 229, 215, 194, 136. HRMS Found 246.02087, C_11_H_12_Cl_2_O_2_: calcd. 246.0209. IR (film): ν̃_max_ = 3360 (O–H), 3036 (C–H), 2973 (C–H), 2926 (C–H), 2910 (C–H), 1608 (C=O), 1510, 1482 (C=C), 1441, 1400 cm^–1^.

**3,3‐Dichloro‐5‐methyl‐2‐(*p*‐tolyl)tetrahydrofuran‐2‐ol (13d):** 8 Isolated as a mixture of diastereomers (1:1.8); 463 mg, 89 %, pale yellow oil. ^1^H NMR (CDCl_3_, 600 MHz): *δ* = 7.67 (d,* J *= 8.1 Hz, 2 H, major ArH), 7.63 (d,* J *= 8.2 Hz, 2 H, minor ArH), 7.23–7.18 (m, 4 H, major ArH, minor ArH), 4.64–4.57 (m, 1 H, minor CH), 4.57–4.49 (m, 1 H, major CH), 3.29 (dd, *J *= 13.7, 9.2 Hz, 1 H, major 1 × CH_2_), 3.16 (br. s, 1 H, major OH), 3.09 (br. s, 1 H, minor OH), 3.01 (dd, *J *= 13.0, 5.9 Hz, 1 H, minor 1 × CH_2_), 2.88 (dd, *J *= 13.0, 9.5 Hz, 1 H, minor 1 × CH_2_), 2.70 (dd, *J *= 13.7, 3.3 Hz, 1 H, major 1 × CH_2_), 2.39 (s, 3 H, major Ar*CH*_3_), 2.38 (s, 3 H, minor Ar*CH*_3_), 1.49 (d,* J *= 6.4 Hz, 3 H, major CH*CH*_3_), 1.47 (d,* J *= 6.3 Hz, 3 H, minor CH*CH*_3_) ppm. ^13^C NMR (CDCl_3_, 150 MHz): *δ* = 139.4, 139.4, 134.9, 134.6, 128.5, 128.5, 127.3, 127.3, 106.5, 106.1, 91.8, 91.2, 74.0, 72.0, 51.8, 51.0, 22.7, 21.4, 21.4, 21.2 ppm. LRMS (CI) 264 (1 %, [M + H]^+^, 2 × ^37^Cl), 262 (5 %, [M + H]^+^, ^35^Cl, ^37^Cl), 260 (6 %, [M + H]^+^, 2 × ^35^Cl), 247 (14 %, [M – CH_3_]^+^), 245 (65 %, [M – CH_3_]^+^), 243 (100 %, [M – CH_3_]^+^).

**Preparation of 1,4 diols:** NaBH_4_ (23 mg, 0.60 mmol) was added to a stirred solution of lactol **12**/**13** (0.50 mmol) in MeOH (4 mL) at 0 °C. After 10 min, NH_4_Cl (5 mL) was added and the organic phase extracted with EtOAc (2 × 5 mL). The organic layer was washed with brine (2 × 5 mL), dried (MgSO_4_), filtered and concentrated in vacuo. The resultant residue was purified by column chromatography (30 % EtOAc in petrol) to give the diol.

**2,2‐Dibromo‐1‐(*p*‐tolyl)butane‐1,4‐diol (14):** 198 mg, quantitative, white solid, m.p. 105–107 °C. ^1^H NMR (CDCl_3_, 600 MHz): *δ* = 7.47 (d,* J *= 8.0 Hz, 2 H, ArH), 7.18 (d,* J *= 8.0 Hz, 2 H, ArH), 5.01 (d,* J *= 1.9 Hz, 1 H, CH), 4.11–4.00 (m, 2 H, *CH*_2_OH), 3.84 (d,* J *= 1.6 Hz, 1 H, *C*H*OH*), 2.76 (dt, *J *= 15.0, 6.4 Hz, 1 H, 1 × CH_2_CBr_2_), 2.63 (dt, *J *= 15.0, 5.7 Hz, 1 H, 1 × CH_2_CBr_2_), 3.04 (s, 3 H, CH_3_), 2.26 (s, 1 H, CH_2_ *OH*) ppm. ^13^C NMR (CDCl_3_, 150 MHz): *δ* = 138.9, 134.1, 129.1, 128.6, 82.3, 79.4, 61.7, 48.1, 21.4 ppm. LRMS (EI) 340 (42 %, M^+^, 2 × ^81^Br), 338 (84 %, M^+^, ^79^Br, ^81^Br), 336 (41 %, M^+^, 2 × ^81^Br), 160 (100 %). HRMS Found 335.9352, C_11_H_14_Br_2_O_2_: calcd. 335.9355. IR (film): ν̃_max_ = 3338 (O–H), 2902 (C–H), 1515 (C=C) cm^–1^.

**2,2‐Dichloro‐1‐(*p*‐tolyl)butane‐1,4‐diol (15):** 84 mg, 68 %, white solid, m.p. 92–93 °C. ^1^H NMR (CDCl_3_, 600 MHz): *δ* = 7.42 (d,* J *= 8.0 Hz, 2 H, ArH), 7.18 (d,* J *= 8.0 Hz, 2 H, ArH), 5.01 (s, 1 H, CH), 4.07–3.95 (m, 2 H, CH_2_CCl_2_), 2.61 (ddd, *J *= 15.0, 7.1, 5.6 Hz, 1 H, 1 × CH_2_CBr_2_), 2.54 (s, 1 H, CH_2_ *OH*), 2.45 (dt, *J *= 15.0, 5.5 Hz, 1 H, 1 × CH_2_CCl_2_), 2.37 (s, 3 H, CH_3_) ppm. ^13^C NMR (CDCl_3_, 150 MHz): *δ* = 138.8, 133.8, 128.9, 128.6, 95.5, 81.2, 59.4, 46.1, 21.4 ppm. LRMS (EI) 252 (1 %, [M]^+^, 2 × 37Cl), 250 (5 %, [M]^+^, ^35^Cl, ^37^Cl), 248 (7 %, [M]^+^, 2 × 35Cl), 167 (6 %), 149 (11 %), 121 (100 %). HRMS Found 248.0365, C_11_H_14_Cl_2_O_2_: calcd. 248.0365. IR (film): ν̃_max_ = 3220 (O–H), 2900 (C–H), 2849 (C–H) cm^–1^.

**Preparation of Dihalogenated Cyclic Ethers:** Using flame dried glassware and under an argon atmosphere, BF_3_ **·**OEt_2_ (213 mg, 1.50 mmol) was added to a solution of lactol **12** (0.50 mmol) and Et_3_SiH (116 mg, 1.00 mmol) in CH_2_Cl_2_ (1 mL) at –78 °C. The reaction was stirred at –78 °C for 5 min and then warmed to room temperature over 30 min. Solvent was removed in vacuo and the resultant residue purified by column chromatography (10 % EtOAc in petrol).

**3,3‐Dibromo‐2‐(*p*‐tolyl)tetrahydrofuran (16):** 158 mg, quantitative, colourless oil. ^1^H NMR (CDCl_3_, 600 MHz): *δ* = 7.49 (d,* J *= 8.1 Hz, 2 H, ArH), 7.21 (d,* J *= 8.1 Hz, 2 H, ArH), 5.02 (s, 1 H, ArCH), 4.29 (td, *J *= 8.5, 7.4 Hz, 1 H, 1 × CH_2_O), 4.16–4.07 (m, 1 H, 1 × CH_2_O), 3.23–3.16 (m, 2 H, CH_2_CBr_2_), 2.39 (s, 3 H, CH_3_) ppm. ^13^C NMR (CDCl_3_, 150 MHz): *δ* = 138.8, 133.2, 128.6, 127.5, 90.7, 66.8, 65.7, 50.2, 21.5 ppm. LRMS (APCI+) 323 (34 %, [M + H]^+^, 2 × ^81^Br), 321 (42 %, [M + H]^+^, ^79^Br, ^81^Br), 319 (30 %, [M + H]^+^, 2 × ^79^Br), 305 (34 %, [M + H – H_2_O]^+^, 2 × ^81^Br), 303 (61 %, [M + H – H_2_O]^+^, ^79^Br, ^81^Br), 301 (34 %, [M + H – H_2_O]^+^, 2 × ^79^Br), 241 (100 %, [M + H – Br]^+^, ^81^Br), 239 (96 %, [M + H – Br]^+^, ^79^Br). HRMS Found 300.9225, C_11_H_11_Br_2_ ([M + H – H_2_O]^+^): calcd. 300.9228. IR (film): ν̃_max_ = 2953 (C–H), 2892 (C–H) cm^–1^.

**3,3‐Dichloro‐2‐(*p*‐tolyl)tetrahydrofuran (17):** 118 mg, quantitative, colourless oil. ^1^H NMR (CDCl_3_, 600 MHz): *δ* = 7.45 (d,* J *= 8.0 Hz, 2 H, ArH), 7.24 (d,* J *= 8.0 Hz, 2 H, ArH), 5.10 (s, 1 H, CH), 4.30 (td, *J *= 8.6, 7.2 Hz, 1 H, 1 × CH_2_O), 4.21 (td, *J *= 8.6, 3.7 Hz, 1 H, 1 × CH_2_O), 3.02 (ddd, *J *= 13.5, 7.2, 3.7 Hz, 1 H, 1 × CH_2_CCl_2_), 2.96 (dt, *J *= 13.5, 8.9 Hz, 1 H, 1 × CH_2_CCl_2_) 2.41 (s, 3 H, CH_3_) ppm. ^13^C NMR (CDCl_3_, 150 MHz): *δ* = 138.7, 132.1, 128.7, 127.5, 89.7, 89.7, 65.5, 47.7, 21.5 ppm. LRMS (EI) 234 (11 %, [M]^+^, 2 × ^37^Cl), 232 (58 %, [M]^+^, ^35^Cl,^37^Cl), 230 (100 %, [M]^+^, 2 × ^35^Cl), 215 (34 %), 217 (24 %), 194 (44 %). HRMS Found 230.02585, C_11_H_12_Cl_2_O: calcd. 230.02597. IR (film): ν̃_max_ = 2953 (C–H), 2894 (C–H) cm^–1^.

**2,2‐Dichloro‐4‐methyl‐1‐phenylpentane‐1,3‐diol (18a):** Ketone **7n** (131 mg, 0.5 mmol) was added to a stirring solution of NMe_4_HB(OAc)_3_ (8 equiv.) under argon atmosphere in MeCN (0.75 mL) and glacial AcOH (0.75 mL) at –40 °C. After 1 h, the reaction mixture was washed with saturated NaHCO_3_ solution (10 mL) and extracted with CH_2_Cl_2_ (10 mL × 3). The combined organic phases were dried with MgSO_4_ and concentrated under reduced pressure which afforded the *anti* diol as a yellow oil (112.6 mg, 85 %). ^1^H NMR (CDCl_3_, 400 MHz): *δ* = 7.56–7.55 (m, 2 H, ArH), 7.39–7.37 (m, 3 H, ArH), 5.29 [s, 1 H, Ph*CH*C(Cl)_2_], 4.10 (d,* J *= 5.3 Hz, 1 H, OH), 3.48 (s, 1 H, OH), 2.79 [d, *J *= 8.0 Hz, 1 H, *CH*CH(CH_3_)_2_], 2.46 [heptd, *J *= 7.0, 2.8 Hz, 1 H, *CH*(CH_3_)_2_], 1.14 [d, *J *= 3.0 Hz, 3 H, CH(*CH*_3_)_2_], 1.12 [d, *J *= 3.0 Hz, 3 H, CH(*CH*_3_)_2_] ppm. ^13^C NMR (CDCl_3_, 176 MHz): *δ* = 137.3, 129.3, 128.8, 127.7, 97.2, 80.0, 79.1, 29.8, 23.1, 16.8 ppm. LRMS (ES+) 262.1 (90 %, [M – H]^+^), 263.1 (55 %, [M]^+^). HRMS Found 285.0421, C_12_H_16_Cl_2_O_2_Na: calcd. 285.0420 ([M + Na]^+^). IR (film): ν̃_max_ = 3405.17 (O–H), 2961.23 (C–H), 2928.81 (C–H), 2871.73 (C–H), 1452.12, 1049.77, 698.36 cm^–1^.

**2,2‐Dichloro‐1,3‐diphenylpropane‐1,3‐diol (18b):** Prepared according to the same procedure as **18a**. Purified via column chromatography (30 % EtOAc in Petrol) which afforded diol **18b** as a White solid (53 mg, 53 %, m.p. 94–96 °C). ^1^H NMR (600 MHz, CDCl_3_): *δ* = 7.57 (br. m, 4 H, ArH), 7.40–7.39 (m, 6 H, ArH), 5.34 (d,* J *= 4.5 Hz, 2 H, *CH*OH), 3.48 (d,* J *= 4.7 Hz, 2 H, OH) ppm. ^13^C NMR (151 MHz, CDCl_3_): *δ* = 137.1, 129.2, 129.1, 127.9, 79.5 ppm. LRMS (NSI) 319 (100 %, [M + Na]^+^, 2 × ^35^Cl), 321 (60 %, [M + Na]^+^, ^35^Cl, ^37^Cl), 323 (10 %, [M + Na]^+^, 2 × ^37^Cl). HRMS Found 319.0263: calcd. 319.0266 ([M + Na]^+^). IR (film): ν̃_max_ = 3388 (O–H), 3060 (C–H), 2920 (C–H), 1452, 1026, 907, 698, 670 cm^–1^.

***anti*‐2,2‐Dibromo‐4‐methyl‐1‐phenylpentane‐1,3‐diol (19):** Prepared according to the same procedure as **18a**: purified via column chromatography (20–70 % EtOAc/Petrol) which afforded **19** as white crystals (31 mg, 0.09 mmol, 29 %); m.p. 79–82 °C. ^1^H NMR (400 MHz, CDCl_3_): *δ* = 7.63–7.59 (m, 2 H, ArH), 7.39–7.36 (m, 3 H, ArH), 5.19 [d, *J *= 4.8 Hz, 1 H, Ph*CH*C(Br)_2_], 4.03 [dd, *J *= 7.9, 2.2 Hz, 1 H, *CH*(OH)CH], 3.61 (d,* J *= 5.0 Hz, 1 H, OH), 2.86 (d,* J *= 7.9 Hz, 1 H, OH), 2.54 [heptd, *J *= 6.9, 2.2 Hz, 1 H, *CH*(CH_3_)_2_], 1.15 [d, *J *= 6.8 Hz, *CH*(*CH*_3_)_2_], 1.13 [d, *J *= 7.0 Hz, 3 H, *CH*(*CH*_3_)_2_] ppm. ^13^C NMR (151 MHz, CDCl_3_): *δ* = 138.0 (C), 129.6 (CH), 128.9 (CH), 127.6 (CH), 85.0 (C), 81.3 (CH), 80.1 (CH), 31.2 (CH), 23.8 (CH_3_), 16.9 (CH_3_) ppm. LRMS (ES+) 373 (51 %, [M + Na]^+^, 2 × ^79^Br), 375 (100 %, [M + Na]^+^, ^79^Br, ^81^Br), 377 (45 %, [M + Na]^+^, 2 × ^81^Br). HRMS (ES+) Found 372.9417, C_12_H_16_Br_2_O_2_Na: calcd. 372.9409 ([M + Na]^+^). IR (film): ν̃_max_ = 3389 (O–H), 2961 (C–H), 2869 (C–H), 1083 (C–O), 733 (C–Br) cm^–1^.

**(±)‐(1*S*,3*R*)‐2,2‐Dibromo‐1‐(*p*‐tolyl)hexane‐1,3‐diol (20) and 2‐Bromo‐1‐(*p*‐tolyl)hexane‐1,3‐diol (21):** Method A: Using flame dried glassware and under an argon atmosphere, **6g** (36 mg, 0.10 mmol) was dissolved in THF (1 mL) and cooled to –78 °C. ZnCl_2_‐TMEDA (25 mg, 0.10 mmol) was added, followed by DIBAL‐H (1 m in hexanes, 300 µL, 0.300 mmol). The reaction was stirred at –78 °C for 1 h. The reaction mixture was poured into a mixture of HCl (6 m, 2 mL) and sat. NH_4_Cl (2 mL). The organic layer was extracted with Et_2_O (3 × 1 mL) and the combined organic layers were dried (Na_2_SO_4_), filtered and concentrated in vacuo. The resultant residue was purified by column chromatography (10 % EtOAc in petrol).

Method B: Using flame dried glassware and under an argon atmosphere, **6g** (36 mg, 0.10 mmol) was dissolved in THF (1 mL) and cooled to –78 °C. DIBAL‐H (1 m in hexanes, 300 µL, 0.300 mmol) was added. The reaction was stirred at –78 °C for 1 h. The reaction mixture was poured into a mixture of HCl (6 m, 2 mL) and sat. NH_4_Cl (2 mL). The organic layer was extracted with Et_2_O (3 × 1 mL) and the combined organic layers were dried (Na_2_SO_4_), filtered and concentrated in vacuo. The resultant residue was purified by column chromatography (10 % EtOAc in petrol).

**2‐Bromo‐1‐(*p*‐tolyl)hexane‐1,3‐diol (21):** Method A: 7 mg, 24 µmol, 24 %; Method B: 5 mg, 17 µmol, 17 %. ^1^H NMR (CDCl_3_, 600 MHz): *δ* = 7.29 (d,* J *= 8.1 Hz, 2 H, ArH), 7.19 (d,* J *= 8.1 Hz, 2 H, ArH), 5.01 (d,* J *= 6.2 Hz, 1 H, ArCH), 4.22 (dd, *J *= 6.2, 1.7 Hz, 1 H, CHBr), 3.48 (br. t, *J *= 6.2 Hz, 1 H, *CH*CH_2_), 3.07 (br. s, 1 H, OH), 2.07 (br. s, 1 H, OH), 2.63 (s, 3 H, Ar*CH*_3_), 1.68–1.55 (m, 1 H, 1 × CH*CH*_2_), 1.55–1.44 (m, 1 H, 1 × CH*CH*_2_), 1.44–1.23 (m, 2 H, *CH*_2_CH_3_), 0.88 (t,* J *= 7.4 Hz, 3 H, CH_2_ *CH*_3_) ppm. ^13^C NMR (CDCl_3_, 150 MHz): *δ* = 138.3, 137.2, 129.4, 126.5, 76.3, 72.3, 70.6, 39.0, 21.3, 18.7, 14.0 ppm. LRMS (CI) 306 (96 %, [M + NH_4_]^+^, ^81^Br), 304 (100 %, [M + NH_4_]^+^, ^79^Br), 288 (25 %, M^+^, ^81^Br), 286 (28 %, M^+^, ^79^Br). HRMS Found 286.0563, C_13_H_19_BrO_2_: calcd. 286.0563. IR (film): ν̃_max_ = 3351 (O–H), 2962 (C–H), 2923 (C–H), 1511, 1461 cm^–1^.

**2,2‐Dibromo‐1‐(*p*‐tolyl)hexane‐1,3‐diol (20):** Using flame dried glassware and under an argon atmosphere, catecholborane (1 m in THF, 10 mL, 10 mmol) was added to a stirring solution of **6g** (182 mg, 0.5 mmol) in THF (35 mL) at –10 °C. The reaction was stirred at –10 °C for 6 h. MeOH (10 mL), saturated sodium potassium tartrate (10 mL) and pinacol (750 mg, 6.36 mmol) were added and the reaction was stirred for 18 h at room temperature. The reaction mixture was diluted with EtOAc (30 mL) and washed with NaOH (0.5 m) until the aqueous layer was colourless. The organic layer was washed with brine (2 × 50 mL), dried (Na_2_SO_4_), filtered and concentrated in vacuo. The resultant residue was purified by column chromatography (20 % EtOAc in petrol) to give **20** as a colourless oil (117 mg, 64 %). ^1^H NMR (CDCl_3_, 600 MHz): *δ* = 7.52 (d,* J *= 8.1 Hz, 2 H, ArH), 7.18 (d,* J *= 8.1 Hz, 2 H, ArH), 5.21 (s, 1 H, Ar*CH*), 3.42 (br. t, *J *= 8.0 Hz, 1 H, *CH*CH_2_), 3.31 (br. s, 1 H, OH), 2.36 (s, 3 H, Ar*CH*_3_), 2.23–2.14 (br. d, *J *= 7.5 Hz, 1 H, OH), 2.19 (m, 1 H, 1 × CH*CH*_2_), 1.68 (dtd, *J *= 14.3, 9.7, 4.8 Hz, 1 H, 1 × CH*CH*_2_), 1.64–1.53 (m, 1 H, 1 × *CH*_2_CH_3_), 1.41–1.33 (m, 1 H, 1 × *CH*_2_CH_3_), 0.93 (t,* J *= 7.4 Hz, 3 H, CH_2_ *CH*_3_) ppm. ^13^C NMR (CDCl_3_, 150 MHz): *δ* = 138.8, 134.6, 128.8, 128.7, 90.8, 80.0, 77.7, 37.3, 21.4, 19.2, 14.0 ppm. LRMS (NSI) 386 (50 %, [M + NH_4_]^+^, 2 × ^81^Br), 384 (100 %, [M + NH_4_]^+^, ^79^Br, ^81^Br), 382 (52 %, [M + NH_4_]^+^, 2 × ^79^Br) 227 (41 %). HRMS Found 382.0016, [C_13_H_18_Br_2_O_2_ + NH_4_]^+^: calcd. 382.0012. IR (film): ν̃_max_ = 3274 (O–H), 2959 (C–H), 2928 (C–H), 1516 (C=C), 1453 cm^–1^.

**(±)‐(*4R*,*6S*)‐5,5‐Dibromo‐2,2‐dimethyl‐4‐propyl‐6‐(p‐tolyl)‐1,3‐dioxane (23) and (±)‐(*4R*,*6S*)‐5‐Bromo‐2,2‐dimethyl‐4‐propyl‐6‐(*p*‐tolyl)‐1,3‐dioxane (23b):** Using flame dried glassware and under an argon atmosphere, **20** (7 mg, 19 µmol) was dissolved in THF (1 mL). *p*‐Toluenesulfonic acid (4 mg, 21 µmol) was added, followed by 2,2‐dimethoxypropane (20 mg, 19 µmol). The reaction was refluxed for 18 h. The reaction mixture was diluted with sat. sodium hydrogen carbonate (1 mL) and extracted with Et_2_O (2 × 1 mL). The combined organic layers were dried (Na_2_SO_4_), filtered and concentrated in vacuo. The resultant residue was purified by column chromatography (2 % EtOAc in petrol) to give **23** and **23b** as a colourless film in a 1:1.3 ratio (4 mg). ^1^H NMR (CDCl_3_, 600 MHz): *δ* = 7.53 (d,* J *= 8.2 Hz, 2 H, *23* ArH), 7.32 (d,* J *= 8.1 Hz, 2 H, **23b** ArH), 7.20–7.15 (m, 4 H, **23** ArH, **23b** ArH), 4.94 (s, 1 H, **23** Ar*CH*), 4.82 (d,* J *= 10.3 Hz, 1 H, **23b** Ar*CH*), 4.03 (ddd, *J *= 10.4, 8.4, 2.3 Hz, 1 H, **23b** *CH*CH_2_), 3.89 (dd, *J *= 9.3, 1.7 Hz, 1 H, **23** *CH*CH_2_), 3.69 (t,* J *= 10.4 Hz, 1 H, **23b** CHBr), 2.37 (s, 3 H, **23** Ar*CH*_3_), 2.35 (s, 3 H, **23b** Ar*CH*_3_), 2.11–2.01 (m, 1 H, **23** 1 × CH*CH*_2_), 1.95 (ddd, *J *= 9.9, 5.8, 5.0, 2.1 Hz, 1 H, **23b** 1 × CH*CH*_2_), 1.78 (dtd, *J *= 14.1, 9.4, 4.8 Hz, 1 H, **23** 1 × CH*CH*_2_), 1.61 [s, 3 H, **23b** 1 × C(CH_3_)_2_], 1.57 [s, 3 H, **23** 1 × C(CH_3_)_2_], 1.56 [s, 3 H, **23** 1 × C(CH_3_)_2_], 1.55–1.49 (m, 1 H,B 1 × CH*CH*_2_), 1.46 [s, 3 H, **23b** 1 × C(CH_3_)_2_], 1.45–1.36 (m, 2 H, **23** *CH*_2_CH_3_), 0.99 (t,* J *= 7.4 Hz, 3 H, **23** CH_2_ *CH*_3_), 0.95 (t, 3 H, **23b** CH_2_ *CH*_3_) ppm. ^13^C NMR (CDCl_3_, 150 MHz): *δ* = 138.8, 138.6, 136.1 (**23b**), 132.9 (**23**), 129.7 (**23**), 129.1 (**23**), 128.0 (**23b**), 127.9 (**23b**), 100.6 (**23**), 99.8 (**23b**), 81.0 (**23**) 79.4 (**23**), 77.9 (**23**), 74.0 (**23b**), 55.0 (**23b**), 35.5 (**23b**), 34.7 (**23**), 29.9, 29.6, 21.4, 21.4, 19.7, 19.4, 19.1, 18.1, 13.9, 13.9 ppm. LRMS (CI) 426 (49 %, [MA + NH_4_]^+^, 2 × ^81^Br), 424 (100 %, [MA + NH_4_]^+^, ^79^Br, ^81^Br), 422 (51 %, [MA + NH_4_]^+^, 2 × ^81^Br), 368 (23 %), 366 (48 %), 364 (48 %), 346 (11 %, [MB + NH_4_]^+^, ^81^Br), 344 (11 %, [MB + NH_4_]^+^, ^79^Br), 312 (87 %, [MB – CH_3_]^+^, ^81^Br), 310 (85 %, [MB – CH_3_]^+^, ^79^Br), 296 (47 %), 294 (50 %), 279 (54 %), 277 (54 %). HRMS Found 422.0325, [C_16_H_23_Br_2_O_2_ + NH_4_]^+^: calcd. 422.0325. IR (film): ν̃_max_ = 2955 (C–H), 2919 (C–H), 2868 (C–H), 2850 (C–H), 1513, 1455 cm^–1^.

An nOe was observed between the methine protons on the six‐membered ring in acetal **23**, confirming the *syn* stereochemistry of the diol.

**2,2‐Dichloro‐4‐methyl‐1‐phenylpentane‐1,3‐diol (24):** In flame dried glassware and under an argon atmosphere, catecholborane (1 m in THF, 8 mL, 8 mmol) was added to a stirring solution of **7a** (261 mg, 1 mmol) in THF (26 mL) at –10 °C. The reaction was stirred at –10 °C for 2 h 30 min. MeOH (8 mL), saturated sodium potassium tartrate (8 mL) and pinacol (568 mg) were added and the reaction was stirred for 20 h at room temp. The reaction mixture was diluted with EtOAc (30 mL) and washed with NaOH (1 m) until the aqueous phase was colourless. The organic phase was washed with brine (3 × 30 mL), dried (MgSO_4_), filtered and concentrated in vacuo. The residue was purified by column chromatography (50 % EtOAc in petrol) to give a mixture of diastereomers *anti/syn* 1:10 as a yellow oil (91 mg, 90 %). ^1^H NMR (CDCl_3_, 400 MHz): *δ* = 7.60–7.57 (m, 2 H, ArH), 7.39–7.35 (m, 3 H, ArH), 5.27 [d, *J *= 3.7 Hz, 1 H, Ar*CH*C(Cl)_2_], 3.64 (dd, *J *= 9.9, 2.5 Hz, 1 H, *CH*CH), 3.34 (d,* J *= 3.9 Hz, 1 H, OH), 2.46 [heptd, *J *= 6.9, 2.6 Hz, 1 H, *CH*(CH_3_)_2_], 2.32 (d,* J *= 10.0 Hz, 1 H, OH), 1.09 [d, *J *= 6.9 Hz, 3 H, CH(*CH*_3_)_2_], 1.01 [d, *J *= 7.0 Hz, 3 H, CH(*CH*_3_)_2_] ppm. ^13^C NMR (CDCl_3_,176 MHz): *δ* = 137.1, 129.0, 128.9, 128.0, 100.1, 80.43, 80.37, 30.0, 22.9, 16.4 ppm. LRMS (ES–) 264.0 (30 %, [M + H]^+^), 263. HRMS Found 280.0866, C_12_H_16_Cl_2_O_2_NH_4_: calcd. 280.0868 ([M + NH_4_]^+^). IR (film): ν̃_max_ = 3401 (O–H), 2961 (C–H), 2928 (C–H), 2872 (C–H), 1492, 699 cm^–1^.

**Preparation of 3,3‐Dihalo‐2‐methoxy‐tetrahydrofurans:** A solution of acetyl chloride in MeOH (1 m, 2 mL mmol^–1^, 2 equiv.) was added to lactol **12**/**13** (1 equiv.) in MeOH (0.5 mL mmol^–1^) and the reaction stirred for 18 h. The reaction mixture was concentrated in vacuo and the product used without further purification.

**3,3‐Dibromo‐2‐methoxy‐2‐(*p*‐tolyl)tetrahydrofuran (25a):** 540 mg, 77 %, white solid, m.p. 106–107 °C. ^1^H NMR (CDCl_3_, 400 MHz): *δ* = 7.63 (d,* J *= 8.0 Hz, 2 H, ArH), 7.21 (d,* J *= 8.0 Hz, ArH), 4.29 (ddd, *J *= 9.0, 8.3, 7.0 Hz, 1 H, 1 × OCH_2_), 4.03 (ddd, *J *= 10.1, 8.3, 2.0 Hz, 1 H, 2 H, 1 × OCH_2_), 3.44 (dt, *J *= 13.3, 9.5 Hz, 1 H, 1 × CH_2_CBr_2_) 3.12 (s, 3 H, OCH_3_), 3.07 (ddd, *J *= 13.3, 7.0, 2.0 Hz, 1 H, 1 × CH_2_CBr_2_), 2.39 (s, 3 H, Ar*CH*_3_) ppm. ^13^C NMR (CDCl_3_, 100 MHz): *δ* = 139.1, 131.8, 128.4, 127.4, 105.9, 68.3, 64.8, 51.4, 47.2, 21.4 ppm. LRMS (CI) 352 (5 %, [M]^+^, 2 × ^81^Br), 350 (11 %, [M]^+^, ^79^Br, ^81^Br), 348 (4 %, [M]^+^, 2 × ^79^Br), 321 (50 %, [M – OCH_3_]^+^, 2 × ^81^Br), 319 (100 %, [M – OCH_3_]^+^, ^79^Br, ^81^Br), 317 (48 %, [M – OCH_3_]^+^, 2 × ^79^Br). HRMS Found 347.9356, C_12_H_14_Br_2_O_2_: calcd. 347.9355. IR (film): ν̃_max_ = 2958 (C–H), 2932 (C–H) cm^–1^.

**3,3‐Dichloro‐2‐methoxy‐2‐(*p*‐tolyl)tetrahydrofuran (25b):** 372 mg, quantitative, white solid, m.p. 96–97 °C. ^1^H NMR (CDCl_3_, 600 MHz): *δ* = 7.56 (d,* J *= 8.2 Hz, 2 H, ArH), 7.21 (d,* J *= 8.2 Hz, 2 H, ArH), 4.26 (ddd, *J *= 9.0, 8.4, 7.4 Hz, 1 H, 1 × OCH_2_), 4.12 (ddd, *J *= 9.6, 8.4, 2.2 Hz, 1 H, 1 × OCH_2_), 3.24–3.16 (m, 1 H, 1 × CH_2_CCl_2_), 3.13 (s, 3 H, OCH_3_), 2.90 (ddd, *J *= 13.0, 7.3, 2.2 Hz, 1 H, 1 × CH_2_CCl_2_), 2.39 (s, 3 H, Ar*CH*_3_) ppm. ^13^C NMR (CDCl_3_, 150 MHz): *δ* = 139.1, 131.0, 128.6, 128.3, 108.9, 91.4, 64.3, 50.9, 44.6, 21.4 ppm. LRMS (CI) 264 (1 %, [M]^+^, 2 × ^37^Cl), 262 (8 %, [M]^+^, ^37^Cl, ^35^Cl), 260 (13 %, [M]^+^, 2 × ^35^Cl), 233 (13 %, [M – OCH_3_]^+^, 2 × ^37^Cl), 231 (67 %, [M – OCH_3_]^+^, ^35^Cl, ^37^Cl) 229 (100 %, [M – OCH_3_]^+^, 2 × ^35^Cl). HRMS Found 260.0366, C_12_H_14_Cl_2_O_2_: calcd. 260.0365. IR (film): ν̃_max_ = 2966 (C–H), 2936 (C–H), 2349, 1509, 1436 cm^–1^.

**3,3‐Dibromo‐2‐methoxy‐5‐methyl‐2‐(*p*‐tolyl)tetrahydrofuran (25c):** Isolated as a mixture of diastereomers (1:2.7); 207 mg, quantitative, pale pink gum. ^1^H NMR (CDCl_3_, 600 MHz): *δ* = 7.70–7.56 (m, 4 H, major ArH, minor ArH), 7.25–7.18 (m, 4 H, major ArH, minor ArH), 4.72–4.62 (m, 1 H, major CH), 4.46–4.36 (m, 1 H, minor CH), 3.61 (dd, *J *= 13.9, 9.4 Hz, 1 H, minor 1 × CH_2_), 3.17–3.12 (m, 7 H, major OCH_3_, minor OCH_3_, major 1 × CH_2_); 3.09 (dd, *J *= 13.2, 9.2 Hz, 1 H, major 1 × CH_2_), 2.97 (dd, *J *= 14.0, 3.2 Hz, 1 H, minor 1 × CH_2_), 2.40 (s, 3 H, minor Ar*CH*_3_), 2.40 (s, 3 H, major Ar*CH*_3_), 1.56 (d,* J *= 6.4 Hz, 3 H, minor CH*CH*_3_), 1.45 (d,* J *= 6.3 Hz, 3 H, major CH*CH*_3_) ppm. ^13^C NMR (CDCl_3_, 150 MHz): *δ* = 139.1, 139.0, 136.0, 132.6, 131.9, 128.4, 128.4, 127.4, 109.3, 109.1, 74.5, 72.2, 69.3, 67.5, 54.4, 53.8, 51.4, 51.1, 21.9, 21.5, 21.5, 21.2 ppm. LRMS (APCI+) 335 (48 %, [M – OCH_3_]^+^, 2 × ^81^Br), 333 (100 %, [M – OCH_3_]^+^, ^79^Br, ^81^Br), 331 (49 %, [M – OCH_3_]^+^, 2 × ^79^Br), 179 (12 %). HRMS Found 330.9326, C_12_H_13_OBr_2_: calcd. 330.9333. IR (film): ν̃_max_ = 2974 (C–H), 2927 (C–H), 1649 (C=C), 1606, 1512 cm^–1^.

**Preparation of 3‐Halofurans:** DBU (10 equiv.) was added to mixed acetal **24** (1 equiv.) in THF (2 mL mmol^–1^) and the mixture heated at reflux until the reaction was complete by TLC. Acetyl chloride in MeOH (1 m) was added to acidify the mixture which was then concentrated in vacuo. The residue was purified by column chromatography (petrol) to give the 3‐halofuran.

**3‐Bromo‐2‐(*p*‐tolyl)furan (26a):** 101 mg, 77 %, colourless oil. ^1^H NMR (CDCl_3_, 600 MHz): *δ* = 7.85 (d,* J *= 8.0 Hz, 2 H, ArH), 7.40 (d, 1.4, 1 H, OCH), 7.25 (d,* J *= 8.0 Hz, 2 H, ArH), 6.52 (d,* J *= 1.4 Hz, 1 H, CBrCH), 2.38 (s, 3 H, CH_3_) ppm. ^13^C NMR (CDCl_3_, 150 MHz): *δ* = 149.3, 141.5, 138.1, 129.3, 127.1, 125.6, 116.2, 95.4, 21.5 ppm. LRMS (EI) 238 (100 %, [M]^+^, ^81^Br), 236 (98 %, [M]^+^, ^79^Br), 209 (26 %), 207 (24 %), 157 (16 %, [M – Br]^+^), 129 (100 %). IR (film): ν̃_max_ = 3026 (C–H), 2920 (C–H), 1565, 1519, 1490 cm^–1^.

**3‐Chloro‐2‐(*p*‐tolyl)furan (26b):** 53 mg, 44 %, colourless oil. ^1^H NMR (CDCl_3_, 600 MHz): *δ* = 7.81 (d,* J *= 8.3 Hz, 2 H, ArH), 7.38 (d,* J *= 2.0 Hz, 1 H, OCH), 7.24 (d,* J *= 8.3 Hz, 2 H, ArH), 6.47 (d,* J *= 2.0 Hz, 1 H, CHCCl), 2.39 (s, 3 H, CH_3_) ppm. ^13^C NMR (CDCl_3_, 150 MHz): *δ* = 147.7, 140.8, 137.9, 129.4, 126.9, 125.1, 114.1, 110.9, 21.5 ppm. LRMS (APCI+) 194 (34 %, [M]^+^, ^37^Cl), 192 (100 %, [M]^+^, ^35^Cl), 179 (87 %), 161 (47 %). HRMS Found: 192.0346, C_11_H_9_ClO: calcd. 192.0342. IR (film): ν̃_max_ = 3035 (C–H), 2920 (C–H), 1519, 1490 cm^–1^.

**3‐Bromo‐5‐methyl‐2‐(*p*‐tolyl)furan (26c):** 42 mg, 76 %, orange oil. ^1^H NMR (CDCl_3_, 600 MHz): *δ* = 7.82 (d,* J *= 8.3 Hz, 2 H, ArH), 7.22 (d,* J *= 8.3 Hz, 2 H, ArH), 6.12 (q,* J *= 0.9 Hz, 1 H, CH), 3.27 (s, 3 H, Ar*CH*_3_), 2.35 (d,* J *= 0.9 Hz, 3 H, OCCH_3_) ppm. ^13^C NMR (CDCl_3_, 150 MHz): *δ* = 151.4, 147.5, 137.5, 129.2, 127.4, 125.2, 112.2, 95.8, 21.5, 13.8 ppm. LRMS (EI) 252 (98 %, [M]^+^, ^81^Br), 250 (100 %, [M]^+^, ^79^Br), 209 (22 %), 207 (24 %), 143 (21 %), 128 (36 %). HRMS Found 249.9989, C11H12BrO: calcd. 249.9988. IR (film): ν̃_max_ = 3022 (C–H), 2915 (C–H), 1599, 1553, 1497 cm^–1^.

**(±)‐(*3aS*,*4S*,*7S*,*7aR*)‐5‐Bromo‐2,7‐dimethyl‐4‐(p‐tolyl)‐3a,4,7,7a‐tetrahydro‐1*H*‐4,7‐epoxyisoindole‐1,3(2H)‐dione (*endo*‐27):** *N*‐Methylmaleimide (63 mg, 0.57 mmol) was added to a stirring solution of **26c** (24 mg, 0.096 mmol) in dimethylcarbonate (1 mL). The reaction was stirred for 3 d at room temp. The reaction mixture was concentrated in vacuo and purified by column chromatography (10 % EtOAc in petrol) to give ***endo*‐27** as a pale orange solid (29 mg, 84 %, m.p. 114–116 °C). ^1^H NMR (CDCl_3_, 600 MHz): *δ* = 7.72 (d,* J *= 8.2 Hz, 2 H, ArH), 7.27 (d,* J *= 8.2 Hz, 2 H, ArH), 6.42 (s, 1 H, C=CH), 3.84 (d,* J *= 7.5 Hz, 1 H, CH_3_C*CH*), 3.38 (d,* J *= 7.5 Hz, 1 H, ArC*CH*), 2.93 (s, 3 H, NCH_3_), 2.40 (s, 2 H, Ar*CH*_3_), 1.88 (s, 3 H, CCH_3_) ppm. ^13^C NMR (CDCl_3_, 150 MHz): *δ* = 174.7, 173.5, 139.1, 136.4, 134.3, 131.8, 129.2, 127.7, 93.8, 88.5, 54.7, 52.6, 25.0, 21.4, 19.0 ppm. LRMS (APCI+) 364 (7 %, [M + H]^+^, ^81^Br), 362 (8 %, [M + H]^+^, ^79^Br), 282 (22 %, [M – Br]^+^), 269 (98 %, [M – C_7_H_7_]^+^, ^81^Br), 267 (100 %, [M – C_7_H_7_]^+^, ^79^Br), 187 (23 %), 172 (43 %). HRMS Found 362.0384, C_17_H_17_BrNO_3_: calcd. 362.0392. IR (film): ν̃_max_ = 2918 (C–H), 2851 (C–H), 1689 (C=O), 1584, 1431 cm^–1^.

**(±)‐(*3aS*,*4S*,*7S*,*7aR*)‐5‐Chloro‐2‐methyl‐4‐(*p*‐tolyl)‐3a,4,7,7a‐tetrahydro‐1*H*‐4,7‐epoxyisoindole‐1,3(2H)‐dione (*endo*‐28) and (±)‐(3*aR*,*4S*,*7S*,*7aS*)‐5‐Chloro‐2‐methyl‐4‐(*p*‐tolyl)‐3a,4,7,7a‐tetrahydro‐1*H*‐4,7‐epoxyisoindole‐1,3(2*H*)‐dione (*exo*‐28):** *N*‐Methylmaleimide (40 mg, 0.36 mmol) was added to a solution of **26b** (14 mg, 0.075 mmol) in [D_4_]MeOD (0.6 mL) and the reaction mixture placed in an NMR tube. After 7 d the reaction was complete by NMR and the reaction mixture was concentrated in vacuo and purified by column chromatography (10 % EtOAc in petrol) to give ***endo*‐28** (5.8 mg, 25 %). Further elution gave ***exo*‐28** (6.6 mg, 29 %).

***endo*‐28:** 5.8 mg, 25 %, white solid, m.p. 106–109 °C. ^1^H NMR ([D_4_]MeOD, 600 MHz): *δ* = 7.70 (d,* J *= 8.2 Hz, 2 H, ArH), 7.26 (d,* J *= 8.2 Hz, 2 H, ArH), 6.46 (d,* J *= 2.0 Hz, 1 H, C=CH), 5.37 (dd, *J *= 5.5, 2.0 Hz, 1 H, OCH), 3.91 (dd, *J *= 7.5, 5.5 Hz, 1 H, OCH*CH*), 3.79 (d,* J *= 7.5 Hz, 1 H, ArC*CH*), 2.87 (s, 3 H, NCH_3_), 2.38 (s, 3 H, Ar*CH*_3_) ppm. ^13^C NMR ([D_4_]MeOD, 150 MHz): *δ* = 176.4, 175.3, 142.0, 140.1, 132.7, 130.2, 129.9, 128.9, 94.5, 79.7, 51.6, 51.2, 24.9, 21.3 ppm. LRMS (CI) 323 (40 %, [M + NH_4_]^+^, ^37^Cl), 321 (100 %, [M + NH_4_]^+^, ^35^Cl), 193 (17 %). HRMS Found 321.1001, C_16_H_18_ClN_2_O_3_^+^ ([M + NH_4_]^+^): calcd. 321.1000. IR (film): ν̃_max_ = 2922 (C–H), 1772, 1689 (C=O), 1589, 1519 cm^–1^.

***exo*‐28:** 6.6 mg, 29 %, white solid, m.p. 143–144 °C. ^1^H NMR ([D_4_]MeOD, 600 MHz): *δ* = 7.42 (d,* J *= 8.2 Hz, 2 H, ArH), 7.21 (d,* J *= 8.2 Hz, 2 H, ArH), 6.57 (d,* J *= 2.1 Hz, 1 H, C=CH), 5.31 (d,* J *= 2.1 Hz, 1 H, OCH), 3.53 (d,* J *= 6.4 Hz, 1 H, OCH*CH*), 3.37 (d,* J *= 6.4 Hz, 1 H, ArC*CH*), 2.79 (s, 3 H, NCH_3_), 2.36 (s, 3 H, Ar*CH*_3_) ppm. ^13^C NMR ([D_4_]MeOD, 150 MHz): *δ* = 177.5, 175.2, 144.7, 139.5, 132.0, 130.0, 129.3, 128.3, 95.0, 81.7, 53.6, 50.0, 24.9, 21.3 ppm. LRMS (CI) 323 (40 %, [M + NH_4_]^+^, ^37^Cl), 321 (100 %, [M + NH_4_]^+^, ^35^Cl), 193 (18 %). HRMS Found 321.1001, C_16_H_18_ClN_2_O_3_: calcd. 321.1000. IR (film): ν̃_max_ = 2920 (C–H), 1764, 1687 (C=O), 1591, 1519 cm^–1^.

**Preparation of Halogenated Cyclobutanols:** Dibromoisocyanuric acid (0.6 equiv.) was added to a stirring solution of propargylic alcohol **1q** or **1r** (1.0 equiv.) in MeCN/H_2_O (7:3, 8.5 mL mmol^–1^). The solution was stirred for 1 h. Saturated Na_2_S_2_O_3_ was added and the mixture extracted with EtOAc. The organic layers were dried (MgSO_4_), filtered and concentrated in vacuo. The resultant residue was purified by column chromatography (10 % EtOAc in petrol) to give the cyclobutane products.

**(*E*)‐2‐[1‐Bromo‐2‐hydroxy‐2‐(*p*‐tolyl)ethylidene]cyclobutan‐1‐ol (29a):** Isolated as a mixture of diastereomers (1:1.6); 168 mg, 24 %, pale yellow oil. ^1^H NMR (CDCl_3_, 600 MHz): *δ* = 7.35 (d, 8.1, 2 H, minor ArH), 7.33 (d,* J *= 8.1 Hz, 2 H, major ArH), 7.19 (d,* J *= 8.1 Hz, 4 H, minor ArH, major ArH), 5.54 (br. d, *J *= 3.6 Hz, 1 H, major Ar*CH*), 5.52 (br. s, 1 H, minor Ar*CH*), 4.97–4.89 (m, 2 H, major CH_2_ *CH*, minor CH_2_ *CH*), 3.58 (br. d, *J *= 5.7 Hz, 1 H, OH), 3.50 (br. d, *J *= 3.6 Hz, 1 H, OH), 3.48 (br. s, 1 H, OH), 3.28 (br. s, 1 H, OH), 2.63–2.48 (m, 2 H, major 1 × C=CCH_2_, minor 1 × C=CCH_2_), 2.42–2.29 (m, 4 H, major 2 × CH*CH*_2_, minor 2 × CH*CH*_2_), 2.36 (s, 6 H, major CH_3_, minor CH_3_) ppm. ^13^C NMR (150 MHz, CDCl_3_): *δ* = 144.7 (major), 144.7 (minor), 138.3, 138.2, 138.0, 137.9, 129.4, 127.0 (major), 126.9 (minor), 121.7 (minor), 120.5 (major), 76.3, 75.9, 70.9, 70.5, 27.5, 27.2, 25.8, 25.3, 21.4 ppm. LRMS (CI) 284 (96 %, [M]^+^, ^81^Br), 282 (100 %, [M]^+^, ^79^Br), 267 (17 %, [M – OH]^+^, ^81^Br), 265 (18 %, [M – OH]^+^, ^79^Br), 185 (19 %). HRMS Found 282.02508, C_13_H_15_BrO_2_: calcd. 282.02499. IR (film): ν̃_max_ = 3313 (O–H), 2977 (C–H), 2945 (C–H), 2917 (C–H), 1683, 1510, 1418 cm^–1^; Relative geometry assigned by analogy to **29b** below.

**(*E*)‐2‐[1‐Bromo‐2‐hydroxy‐2‐(*p*‐tolyl)ethylidene]cyclobutan‐1‐ol (29b):** Isolated as a mixture of diastereomers: (1:1.1); 68 mg, 39 %, pale yellow oil. ^1^H NMR (CDCl_3_, 600 MHz): *δ* = 7.51–7.50 (m, 2 H, major ArH), 7.50–7.48 (m, 2 H, minor ArH), 7.36 (d,* J *= 8.2 Hz, 2 H, minor ArH), 7.31 (d,* J *= 8.2 Hz, 2 H, major ArH), 5.57 (s, 1 H, minor Ar*CH*), 5.56 (s, 1 H, major Ar*CH*), 4.98–4.90 (m, 2 H, major CH_2_ *CH*, minor CH_2_ *CH*), 2.61–2.49 (m, 2 H, major 1 × C=CCH_2_, minor 1 × C=CCH_2_), 2.42–2.30 (m, 4 H, major 1 × C=CCH_2_, major 1 × CH*CH*_2_, minor 1 × C=C*CH*_2_, minor 1 × CH*CH*_2_), 2.05–1.94 (m, 2 H, major 1 × CH*CH*_2_, minor 1 × CH*CH*_2_) ppm. ^13^C NMR (CDCl_3_, 150 MHz): *δ* = 145.4 (minor), 145.3 (major), 140.0 (minor), 139.9 (major), 131.8 (major), 131.7 (minor), 128.8 (major), 128.7 (minor), 122.3 (major), 122.3 (minor), 121.6 (minor), 120.2 (major), 75.1 (major), 75.1 (minor), 70.9 (major), 70.6 (minor), 27.7 (major), 27.6 (minor), 25.7 (minor), 25.2 (major) ppm. LRMS (NSI) 373 (51 %, [M + Na]^+^, 2 × ^81^Br), 371 (100 %, [M + Na]^+^, ^81^Br, ^79^Br), 369 (49 %, [M + Na]^+^, 2 × ^79^Br), HRMS Found 370.9076. C_12_H_12_Br_2_O_2_Na requires 370.9076. IR (film): ν̃_max_ = 3313 (O–H), 2947 (C–H), 1691, 1591 cm^–1^. Relative geometry assigned based on NOESY spectra with key correlations indicated (See supporting information, Figure S1).

## Supporting information

Supporting Information
