# Supplementary material for: Dihalohydration of Alkynols: A Versatile Approach to Diverse Halogenated Molecules
Source: European J Org Chem. 2018 Jul 18;2018(29):4018–28. doi: 10.1002/ejoc.201800668 (PMC6099344; doi:10.1002/ejoc.201800668)
Supplement: Supplementary file 1 — Supporting Information [file EJOC-2018-4018-s001.pdf]

Eur. J. Org. Chem. · ISSN 1099–0690

https://doi.org/10.1002/ejoc.201800668

SUPPORTING INFORMATION

Title: Dihalohydration of Alkynols: A Versatile Approach to Diverse Halogenated Molecules

Author(s): Samantha M. Gibson, Jarryl M. D'Oyley, Joe I. Higham, Kate Sanders, Victor Laserna, Abil E. Aliev, Tom D. Sheppard*

Table of Contents

1. General experimental procedures 1
2. Experimental procedures: Preparation of propargylic alcohols 2
3. Structural assignment of cyclobutane **29b** 7
4. ^{1}H and ^{13}C NMR spectra 11
5. References 60

General experimental procedures

All solvents and chemicals were used as received. Column chromatography was carried out using either Merck Geduran Si 60 (40-63 µm) silica gel or a Biotage purification system using Biotage or Grace silica columns. Analytical thin layer chromatography was carried out using Merck TLC Silica Gel 60 F_{254} aluminium-backed plates. Components were visualised using combinations of ultra-violet lights, potassium permanganate or ninhydrin.

Proton magnetic resonance spectra (^{1}H NMR) were recorded at 400, 500 or 600 MHz on a Bruker Avance spectrometer and are reported as follows: chemical shift δ in ppm (number of protons, multiplicity, coupling constant J in Hz, assignment). The solvent used was deuterated chloroform or deuterated methanol unless stated otherwise. Residual protic solvent was used as the internal reference, setting $CDCl_3$ to δ 7.26 and MeOD-d_4 to 3.31. The following abbreviations are used: s, singlet; d, doublet; t, triplet; q, quartet; m, mulitplet; br, broad or a combination of these. Carbon magnetic resonance spectra (^{13}C NMR) were recorded at 100, 125 or 150 MHz on a Bruker Avance spectrometer using deuterated chloroform or deuterated methanol and using the central reference of $CDCl_3$ to δ 77.0 or MeOD-d_4 to δ 49.15 as the internal standard. Mass Spectrometry data were collected on either TOF or magnetic sector analysers either at the Department of Chemistry, University College London or at the EPSRC UK National Mass Spectroscopy Facility at Swansea University. The ionization method is reported in the experimental data.

Experimental Procedures

Propargylic Alcohols

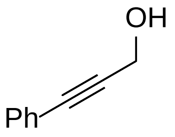

1a

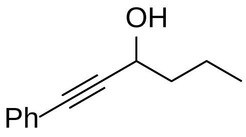

1b

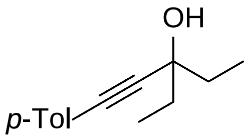

1c

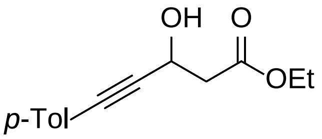

1d

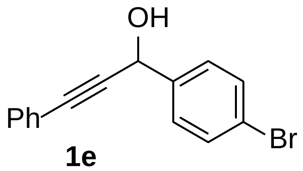

1e

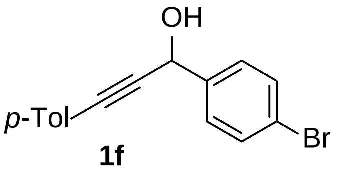

1f

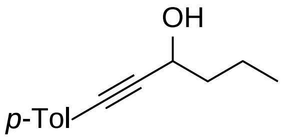

1g

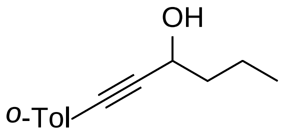

1h

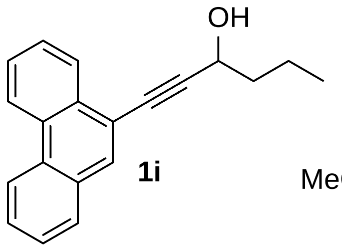

1i

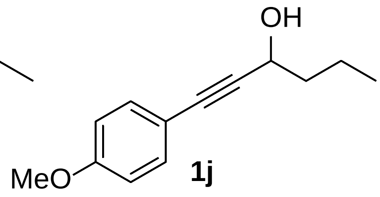

1j

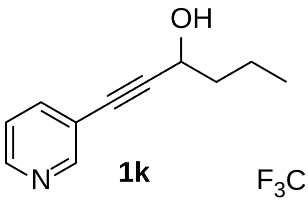

1k

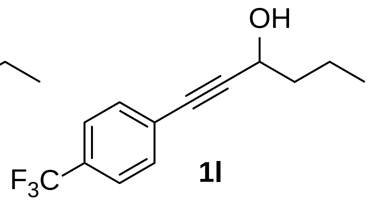

1l

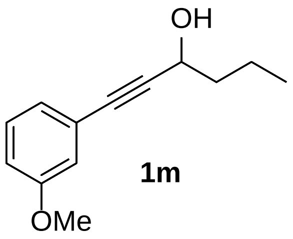

1m

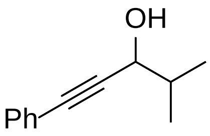

1n

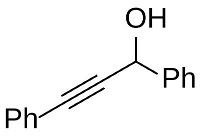

1o

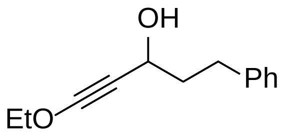

1p

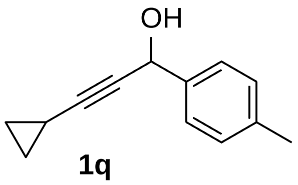

1q

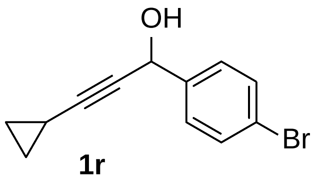

1r

Propargylic alcohol **1a** was purchased from a commercial supplier. We have previously reported the synthesis of propargylic alcohols **1b**,[1] **1c**,[2] **1d**,[2] **1g**,[2] **1h**,[2] **1j**,[3] **1l**,[1] **1n**,[1] **1o**,[4] and **1p**[2]

Preparation of propargylic alcohols

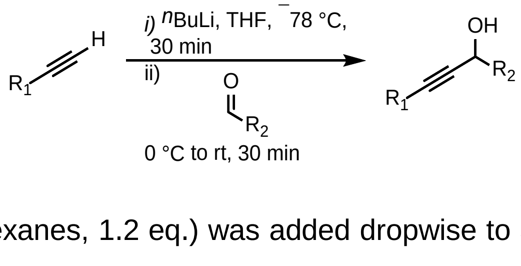

n-Butyllithium (1.6 M in hexanes, 1.2 eq.) was added dropwise to a stirred solution of alkyne (1 eq.) in anhydrous THF (1 mL.mmol^{-1}) at –78 ˚C under an argon atmosphere. After 30 min

aldehyde (1 eq.) was added and the resulting solution was stirred for 5 min at 0 ˚C and then 30 min at rt. The reaction was diluted with aq. saturated NH_4Cl and the organic phase extracted with Et_2O. The combined organic phases were washed with brine, dried ($MgSO_4$), filtered and concentrated *in vacuo*. The residue was purified by column chromatography (EtOAc/Petrol) to give the propargylic alcohol.

1-(4-Bromophenyl)-3-phenylprop-2-yn-1-ol (1e)[5]

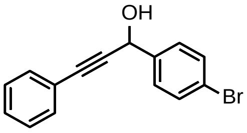

235 mg, 41%, white solid, mp 71-73 °C (lit. 72-73 °C)[5]; **^{1}H NMR** ($CDCl_3$, 400 MHz) *δ* 7.56-7.43 (6H, m, ArH), 7.39-7.29 (3H, m, ArH), 5.66 (1H, d, *J* = 5.9, CH), 2.36 (1H, d, *J* = 5.9, OH); **^{13}C NMR** ($CDCl_3$, 150 MHz) *δ* 139.7, 131.9, 128.9, 128.5, 128.5, 128.4, 122.6, 122.2, 88.2, 87.1, 64.6; **LRMS** (CI) 287 (98%, $[M+H]^+$, Br^{81}), 285 (100%, $[M+H]^+$, Br^{79}), 236 (20%) 234 (21%), 218 (32%), 216 (30%)

1-(4-Bromophenyl)-3-(*p*-tolyl)prop-2-yn-1-ol (1f)[6]

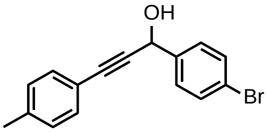

512 mg, 68%, pale yellow solid, mp 97-98 °C; **^{1}H NMR** ($CDCl_3$, 500 MHz) *δ* 7.55-7.50 (2H, m, ArH), 7.50-7.45 (2H, m, ArH), 7.36 (2H, d, *J* = 8.0, ArH), 7.13 (2H, d, *J* = 8.0, ArH), 5.64 (1H, s, CH), 2.52 (3H, s, CH_3); **^{13}C NMR** ($CDCl_3$, 125 MHz) *δ* 139.8, 139.1, 131.8, 131.7, 129.2, 128.5, 122.4, 119.1, 87.6, 87.3, 64.5, 21.6; **LRMS** (EI) 302 (34%, M^+, Br^{81}), 300 (39%, M^+, Br^{79}), 221 (100%, $[M-Br]^+$)

1-(Phenanthren-9-yl)-hex-1-yn-3-ol (1i)

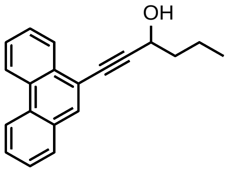

285 mg, 97%, pale yellow solid, mp 80-81 °C; **^{1}H NMR** ($CDCl_3$, 500 MHz) *δ* 8.70-8.63 (2H, m, ArH), 8.44-8.38 (1H, m, ArH), 7.99 (1H, br s, ArH), 7.84 (1H, dd, *J* = 7.9, 0.9, ArH), 7.71-7.63 (3H, m, ArH), 7.63-7.56 (1H, m, ArH), 4.80 (1H, t, *J* = 6.6, CH), 1.98-1.88 (2H, m, CHC**H$_2$**), 1.72-1.62 (2H, m, C**H$_2$**CH_3), 1.05 (3H, t, *J* = 7.4, CH_3); **^{13}C NMR** ($CDCl_3$, 125 MHz) *δ* 132.2, 131.2, 131.1, 130.4, 130.1, 128.6, 127.6, 127.1, 127.1, 127.0, 126.9, 122.8, 122.7, 119.1, 94.9, 83.1, 63.1, 40.2, 18.7, 13.9; **LRMS** (EI) 274 (52%, M^+), 231 (100%, $[M-CH_2CH_2CH_3]^+$) 202 (65%); **HRMS** Found 274.1351, $C_{20}H_{18}O$ requires 274.1352; **IR** ν_{max} (solid/cm^{-1}) 3360 (O-H), 3056 (C-H), 2955 (C-H), 2930 (C-H), 2196 (C≡C), 1450

1-(Pyridin-3-yl)hex-1-yn-3-ol (1k)

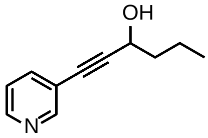

202 mg, 92%, orange oil; **^{1}H NMR** ($CDCl_3$, 600 MHz) *δ* 8.71 (1H, br s, ArH), 8.53 (1H, dd, *J* = 4.9, 1.6, ArH), 7.72 (1H, dt, *J* = 7.9, 1.9, ArH), 7.25-7.22 (1H, m, ArH), 4.36 (1H, q, *J* = 6.6, CH), 2.76 (1H, br s, OH), 1.86-1.73 (2H, m, CHC**H$_2$**), 1.61-1.50 (2H, m, C**H$_2$**CH_3), 1.00 (3H, t, *J* = 7.4, CH_3); **^{13}C NMR** ($CDCl_3$, 150 MHz) *δ* 152.3, 148.6, 138.9, 123.2, 120.2, 94.3, 81.3, 62.6, 39.9, 18.7, 13.9; **LRMS** (ES+) 176 (100%, $[M+H]^+$); **HRMS** Found 176.1078, $C_{11}H_{14}NO$ requires 176.1075; **IR** ν_{max} (film/cm^{-1}) 3220 (O-H), 2957 (C-H), 2932 (C-H), 2871 (C-H), 2201 (C≡C), 1476

1-(3-methoxyphenyl)hex-1-yn-3-ol (1m)

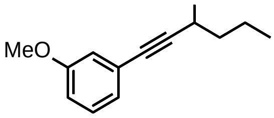

2.28 g, 74%; yellow oil; **^{1}H NMR** (500 MHz, $CDCl_3$) 7.20 (1H, t, J = 7.9, Ar), 7.02 (1H, dt, J = 1.3, 7.6, Ar), 6.96 (1H, m, Ar), 6.87 (1H, ddd, J = 1.0, 2.7, 8.4, Ar), 4.60 (1H, t, J = 5.0, -

CHOH), 3.78 (3H, s, MeO-), 2.20 (1H, br s, -OH), 1.55 (2H, m, $H_3C\mathit{H_2}CH_2C$-), 0.98 (3H, t, J = 7.4, $\mathit{H_3}CH_2C$-); **^{13}C NMR** ($CDCl_3$, 150 MHz) 159.4, 129.5, 124.4, 123.8, 116.6, 115.0, 90.3, 84.8, 62.8, 55.4, 40.1, 18.7, 13.9; **LRMS** (EI) 204 (55%, M^+), 161 (100%, $[M-CH_2CH_2CH_3]^+$); **HRMS** Found 204.11450, $C_{11}H_{14}NO$ requires 204.11448; **IR** ν_{max} (film/cm^{-1}) 3340 (O-H), 2958, 2935, 2872, 1598, 1574.

3-Cyclopropyl-1-(*p*-tolyl)prop-2-yn-1-ol (1q)[7]

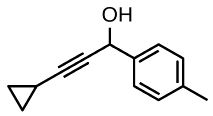

1.85 g, 25%, colourless oil. **^{1}H NMR** ($CDCl_3$, 600 MHz) δ 7.41 (2H, d, J = 8.1, ArH), 7.18 (2H, d, J = 8.1, ArH), 5.39 (1H, s, C**H**OH), 2.36 (3H, s, CH_3), 2.07 (1H, br s, OH), 1.36-1.29 (1H, m, C**H**CH_2), 0.82-0.78 (2H, m, 2 × CH_2), 0.76-0.71 (2H, m, 2 × CH_2); **^{13}C NMR** ($CDCl_3$, 150 MHz) δ 138.5, 138.2, 129.3, 126.7, 90.6, 75.4, 64.8, 21.3, 8.4, 0.3; **LRMS** (CI) 186 (18%, M+), 170 (100%, $[M-OH]^+$); **HRMS** Found 186.1040, $C_{13}H_{14}O$ requires 186.1039; **IR** ν_{max} (film/cm^{-1}) 3370 (O-H), 3007 (C-H), 2917 (C-H), 2862 (C-H), 2232 (C≡C), 1601, 1509.

1-(4-Bromophenyl)-3-cyclopropylprop-2-yn-1-ol (1r)

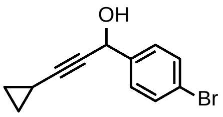

7.47 g, quantitative, orange oil. **^{1}H NMR** ($CDCl_3$, 600 MHz) δ 7.46 (2H, d, J = 8.4, ArH), 7.36 (2H, d, J = 8.4, ArH), 5.33 (1H, s, C**H**OH), 2.82 (1H, br s, OH), 1.36-1.29 (1H, m, C**H**CH_2), 0.82-0.78 (2H, m, 2 × CH_2), 0.76-0.71 (2H, m, 2 × CH_2); **^{13}C NMR** ($CDCl_3$, 150 MHz) δ 140.3, 131.7, 128.5, 122.2, 91.0, 74.9, 64.1, 8.5, 0.4; **LRMS** (CI) 252 (11%, $[M+H]^+$, Br^{81}), 250 (13%, $[M+H]^+$, Br^{79}), 235 (98%, $[M-OH]^+$, Br^{81}), 233 (100%, $[M-OH]^+$, Br^{81}); **HRMS** Found 249.9989, $C_{12}H_{11}BrO$ requires 249.9988; **IR** ν_{max} (film/cm^{-1}) 3349 (O-H), 3006 (C-H), 2877 (C-H), 2230 (C≡C), 1585, 1482.

Homopropargylic alcohols

We have previously reported the preparation of alkynols **4b**[2] and **4d**;[2] **4c** is available commercially.

4-(*p*-Tolyl)but-3-yn-1-ol (4a)[8]

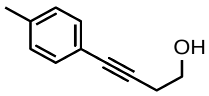

4-Iodotoluene (9.37 g, 43 mmol) and 4-pentyn-1-ol (2.17 g, 25.8 mmol) were dissolved in anhydrous THF (10 mL) under argon. $Pd(PPh_3)_2Cl_2$ (150 mg, 0.215 mmol), CuI (82 mg, 0.43 mmol) and Et_3N (30 mL, 215 mmol) were added and the reaction stirred for 18 h. The mixture was diluted with EtOAc (50 mL) and washed with H_2O (2 × 50 mL) and then brine (2 x 50 mL). The organic layer was dried ($MgSO_4$), filtered and concentrated *in vacuo*. The resultant oil was purified by column chromatography (10% EtOAc in petrol) to give **4a** as a yellow oil (3.52g, 88%). **^{1}H NMR** ($CDCl_3$, 500 MHz) δ 7.30 (2H, d, J = 8.1, ArH), 7.09 (2H, d, J = 8.1, ArH), 3.80 (2H, t, J = 6.3, C**H**$_2$OH), 2.67 (2H, t, J = 6.3, C**H**$_2$CH$_2$OH), 2.33 (3H, s, CH_3); **^{13}C NMR** ($CDCl_3$, 125 MHz) δ 138.0, 131.6, 129.1, 120.3, 85.6, 82.6, 61.3, 23.9, 21.5; **LRMS** (EI) 160 (91%, $[M]^+$) 129 (100%, $[M-CH_2OH]^+$), 115 (32%, $[M-CH_2CH_2OH]^+$)

Structural assignment of cyclobutane 29b

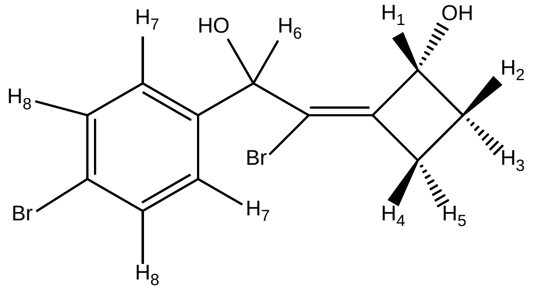

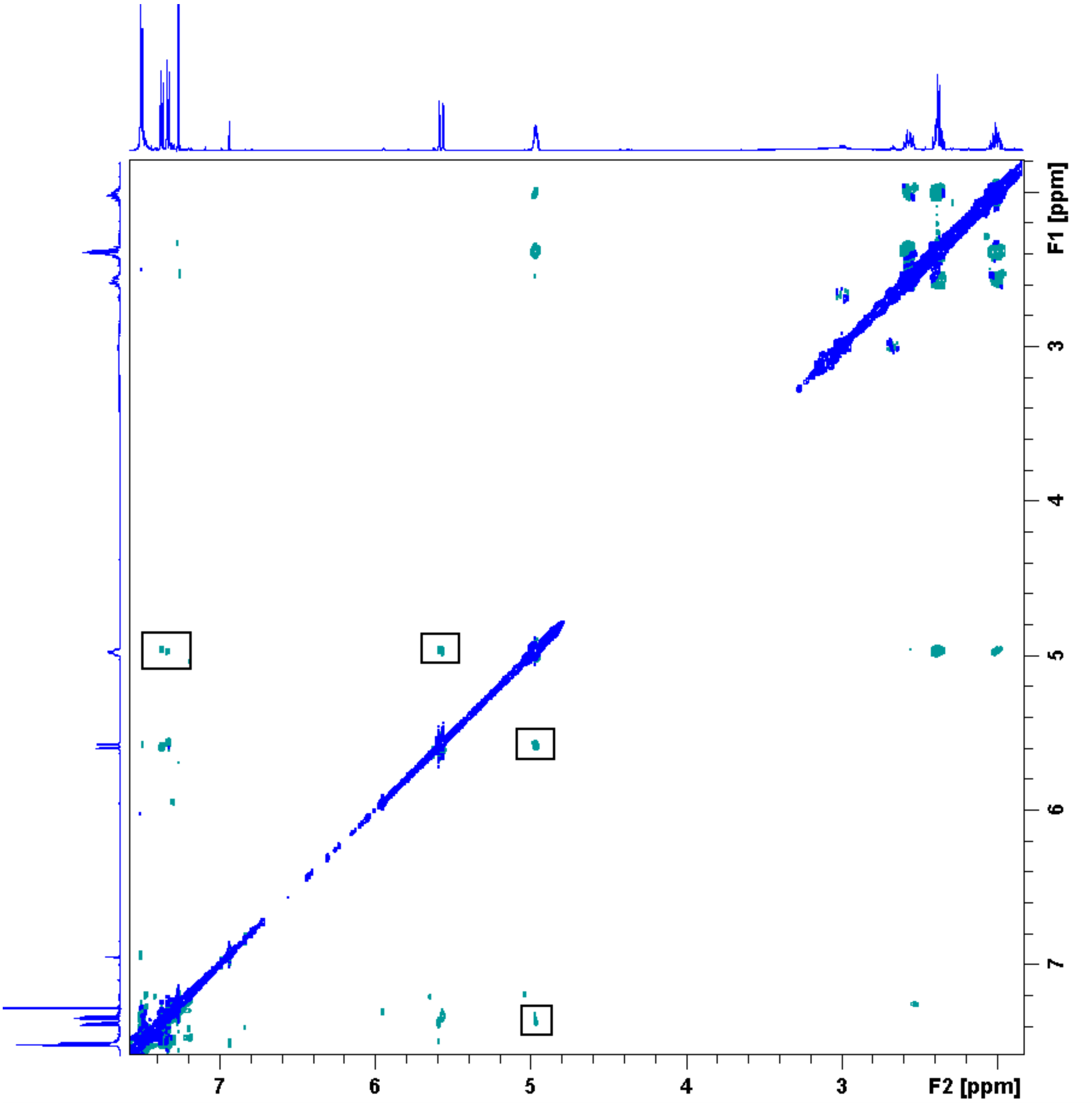

Figure S1. 2D NOESY spectrum of **29b** in $CDCl_3$ (mixing time 600 ms, 298 K, 600 MHz). Cross-peaks for the major diastereomer at (4.97,5.56) and (4.97,7.33) ppm corresponding to proton pairs (H_1, H_6) and (H_1, H_7), respectively, allow to identify the configuration about the double bond shown above. The same configuration is also found for the minor streoisomer,

as confirmed by the cross-peaks at (4.96,5.59) and (4.96,7.37) ppm corresponding to proton pairs (H_1, H_6) and (H_1, H_7), respectively.

The ^{1}H NMR spectrum for a mixture of diastereomers with a ratio of 1:1.1 showed clear signs of strongly coupled spin systems due to very small chemical shift differences for some of the protons. In order to determine accurate values of ^{1}H-^{1}H *J* couplings, full lineshape analysis[9] was carried out using gNMR program.[10] The results of this analysis is summarised in Figure S2.

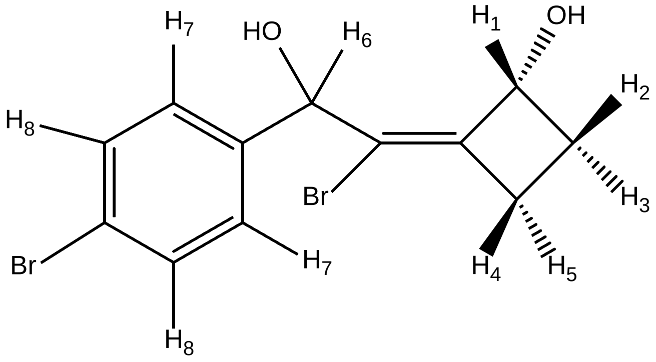

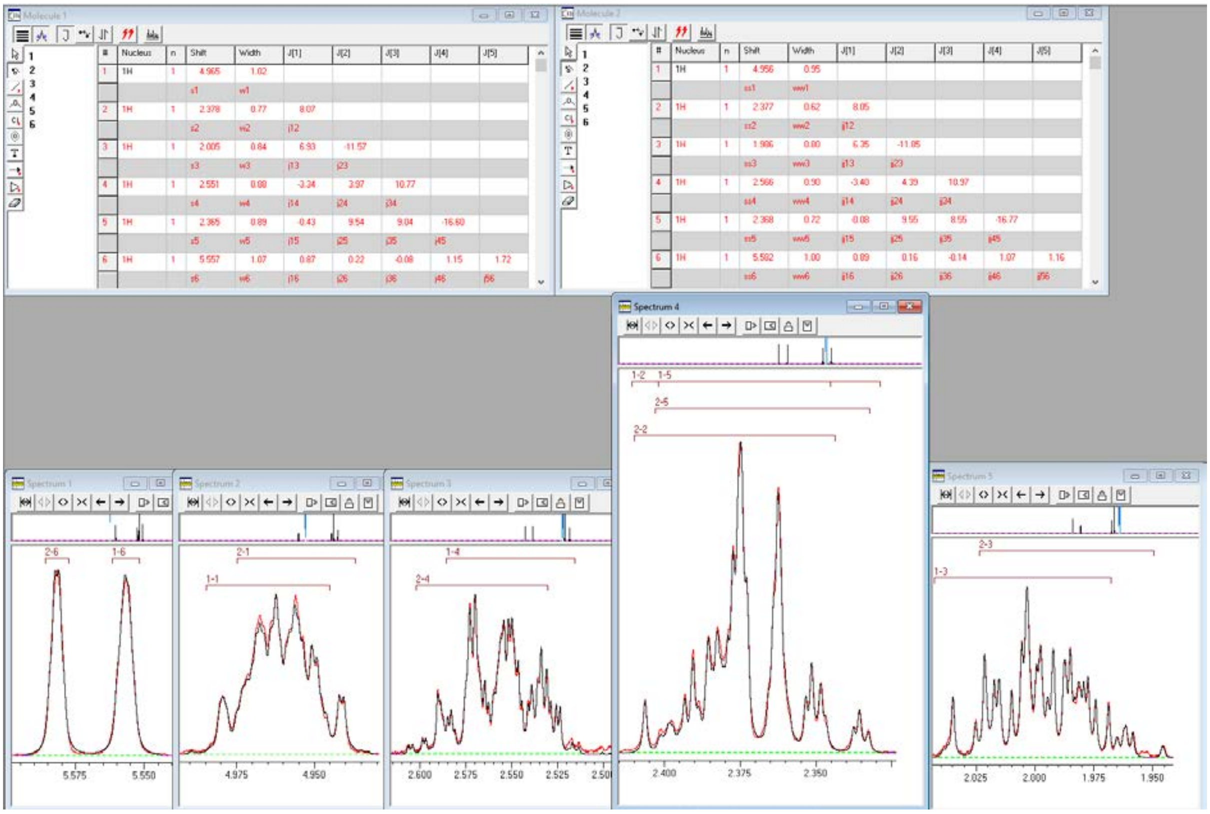

Figure S2. The overlaid view of experimental (red) and fitted (black) ^{1}H NMR lineshapes of **29b** (in $CDCl_3$, 600 MHz, 25 °C). The proton numbering used in spectral fittings is also shown. Molecules **1** and **2** denote major and minor diastereomers, respectively, observed with a ratio of 1.1:1 in the experimental ^{1}H

NMR spectrum. The screenshot of the table of fitting results is also shown (chemical shifts are in ppm; *J* couplings and linewidths are in Hz).

Table S1. Proton *J* couplings (in Hz) derived from the full lineshape analysis of the experimental ^{1}H NMR spectrum in $CDCl_3$ (600 MHz) for major and minor stereoisomers (estimated uncertainty ±0.08 Hz) and DFT calculations at the B3LYP/6-311+G(2d,p) level of theory. Prior to DFT calculations of *J* couplings, molecular geometries for two diastreoisomers (shown below as stuctures **A** and **B**) were optimised at the M062X/def2TZVP level of theory. Chloroform solvent effects were introduced via self-consistent reaction field (SCRF) theory calculations using the IEFPCM method in all DFT calculations.[11] Additional frequency calculations were also undertaken in order to verify that the optimized geometries correspond to true minima. All quantum mechanical calculations were carried out using *Gaussian 09*.[12]

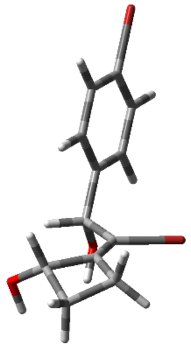

Structure **A**

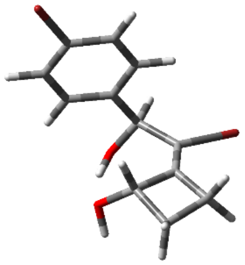

Structure **B**

Proton Pair	*J* couplings (in Hz) in major stereoisomer	*J* couplings (in Hz) in minor stereoisomer	DFT predicted *J* couplings (in Hz) in structure **A**	DFT predicted *J* couplings (in Hz) in structure **B**
1-2	8.07	8.05	7.88	8.07
1-3	6.93	6.35	6.45	5.39
2-3	-11.57	-11.85	-10.31	-11.43
2-5	9.54	9.55	8.90	9.14
2-4	3.97	4.39	2.23	3.13
3-5	9.04	8.55	8.71	7.55
3-4	10.77	10.97	10.03	10.53
1-5	-0.43	-0.08	-1.18	-0.86
1-4	-3.34	-3.40	-3.63	-4.28
4-5	-16.60	-16.77	-15.24	-16.42
5-6	1.72	1.16	0.19	-0.12
1-6	0.87	0.89	0.39	0.01

4-6	1.15	1.07	-0.03	-0.24

The iterative spectral simulations revealed a large negative ^4J coupling between protons H_1 and H_4. To verify the derived *J* couplings, additional DFT calculations of *J* couplings in two diastereomers of **29b** were carried out (Table S1). The DFT calculated *J* couplings were in good agreement with the *J* couplings derived from the full lineshape analysis. The observed differences between DFT-predicted and experimental *J* couplings was similar to those reported previously.[13]

1-(Phenanthren-9-yl)-hex-1-yn-3-ol (1i)

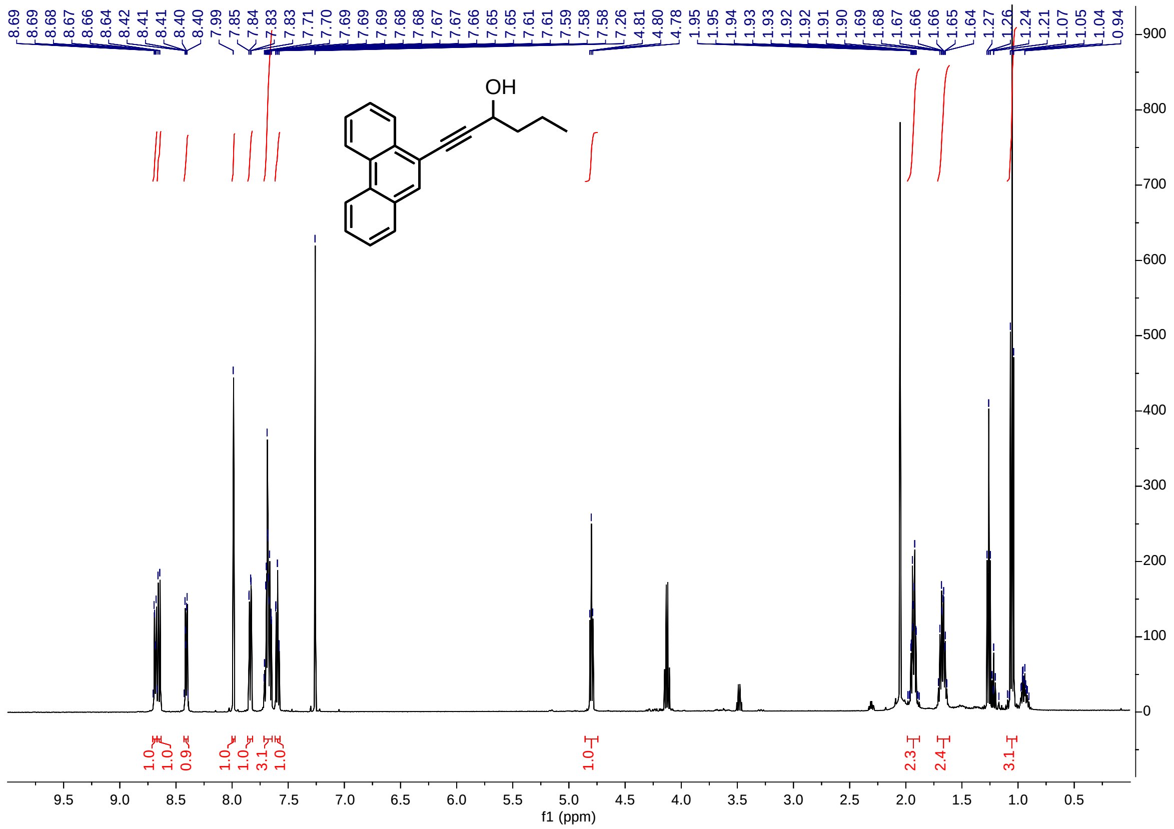

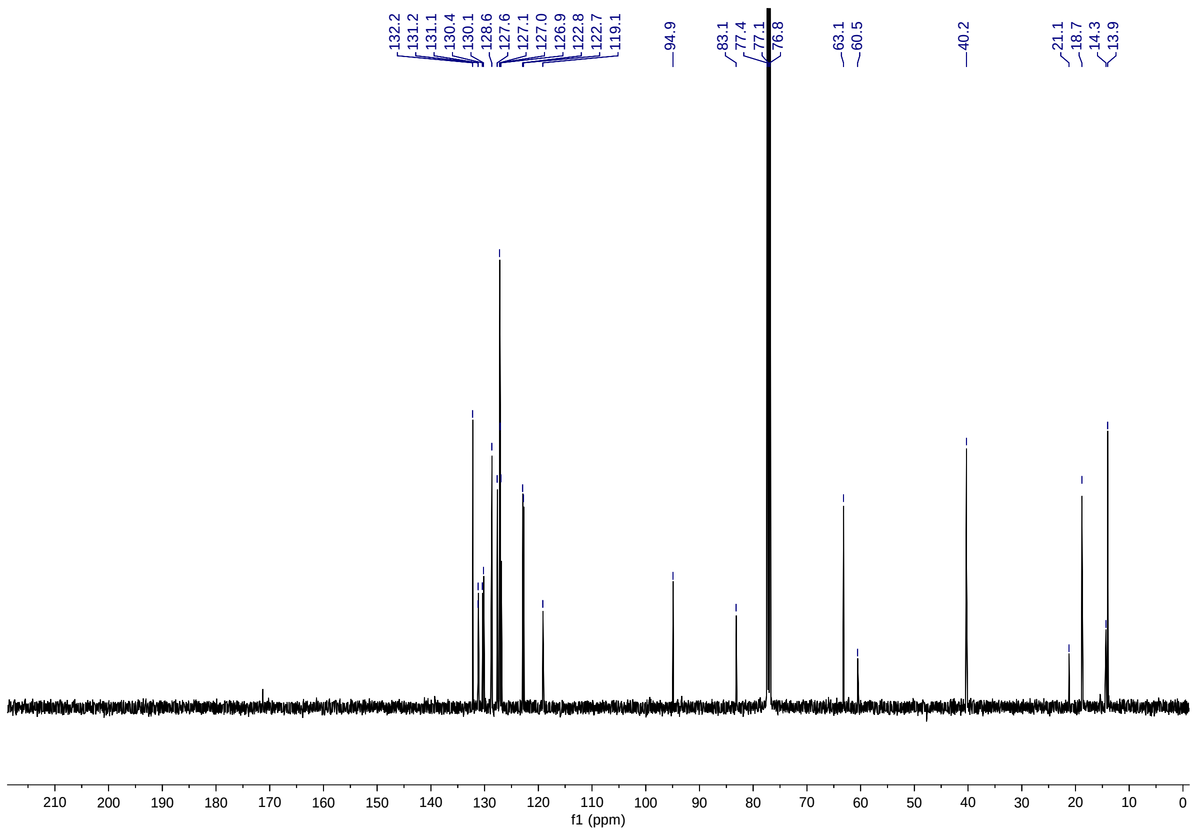

1-(Pyridin-3-yl)hex-1-yn-3-ol (1k)

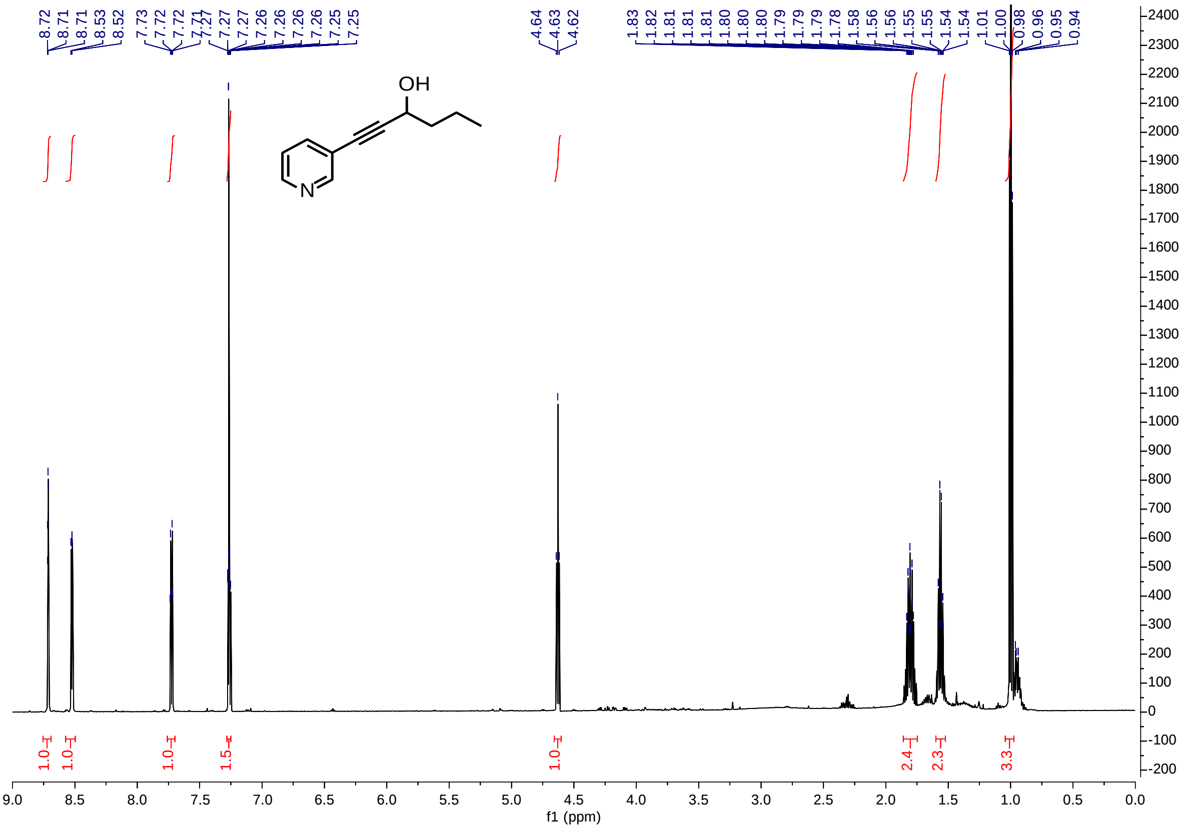

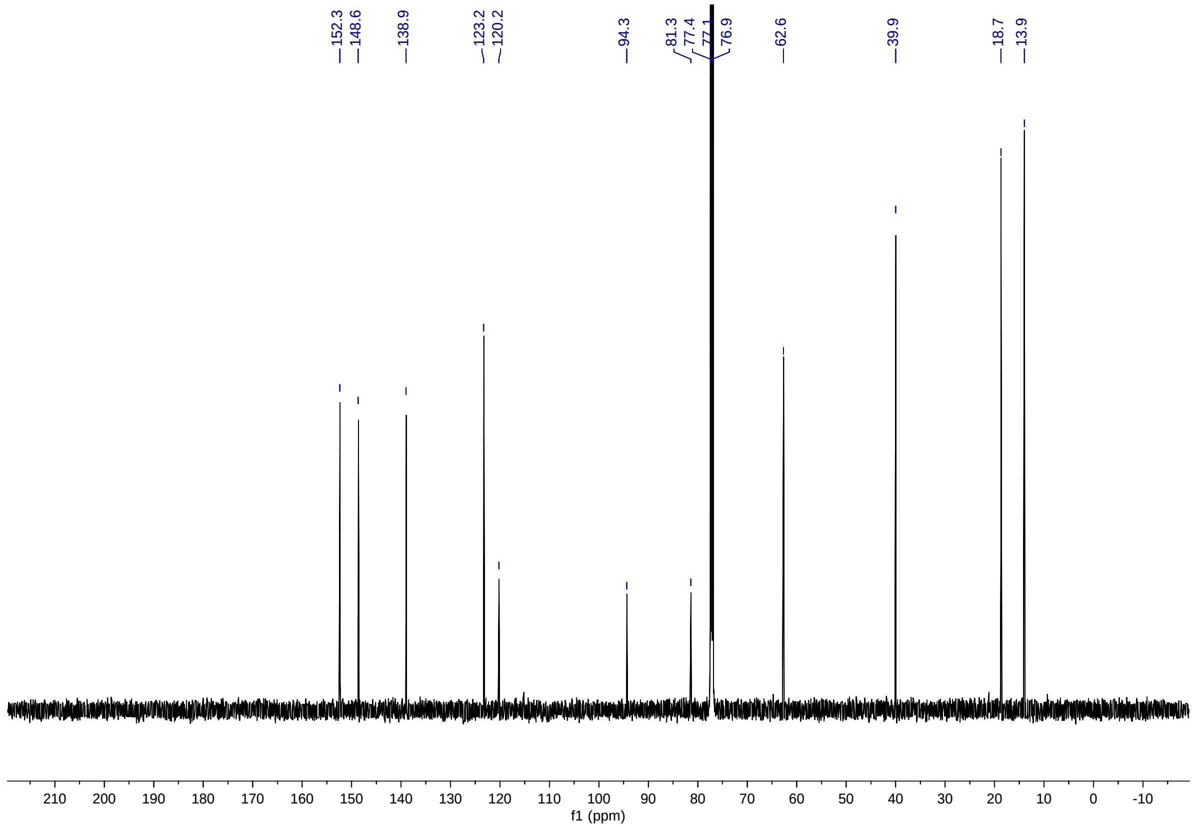

1-(3-Methoxyphenyl)hex-1-yn-3-ol (1m)

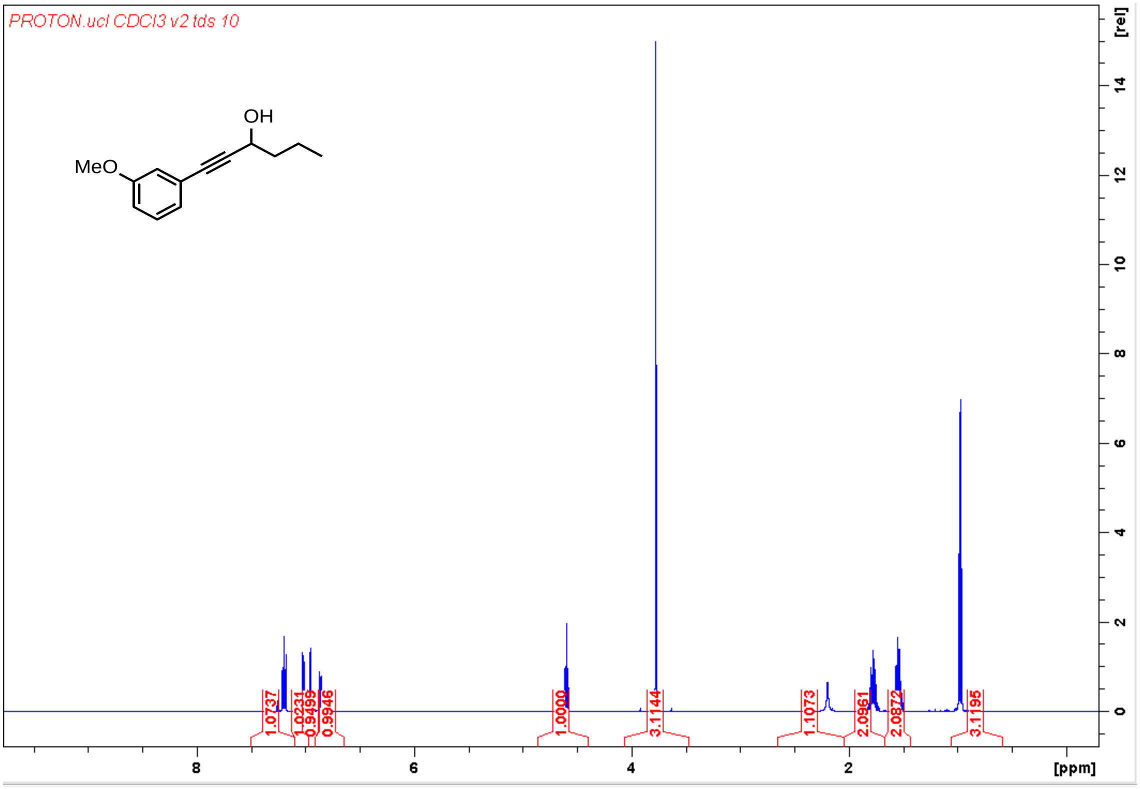

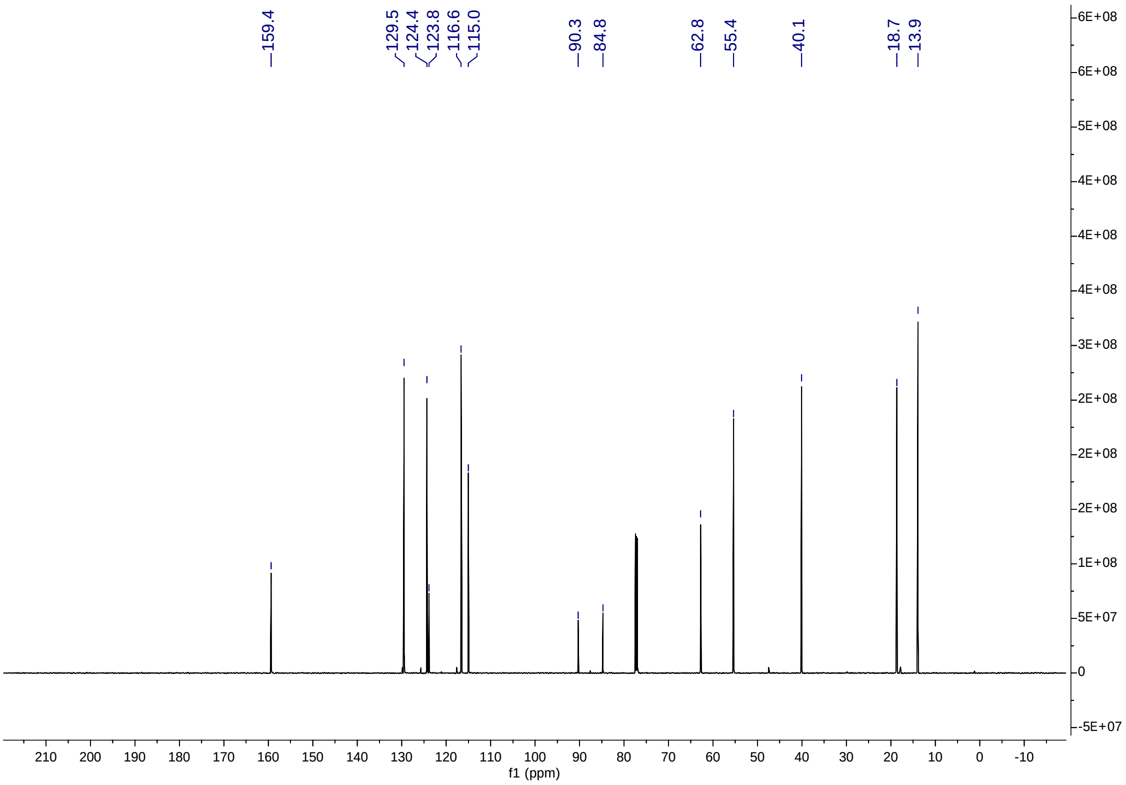

2,2-Dibromo-3-hydroxy-1-phenylpropan-1-one (6a)

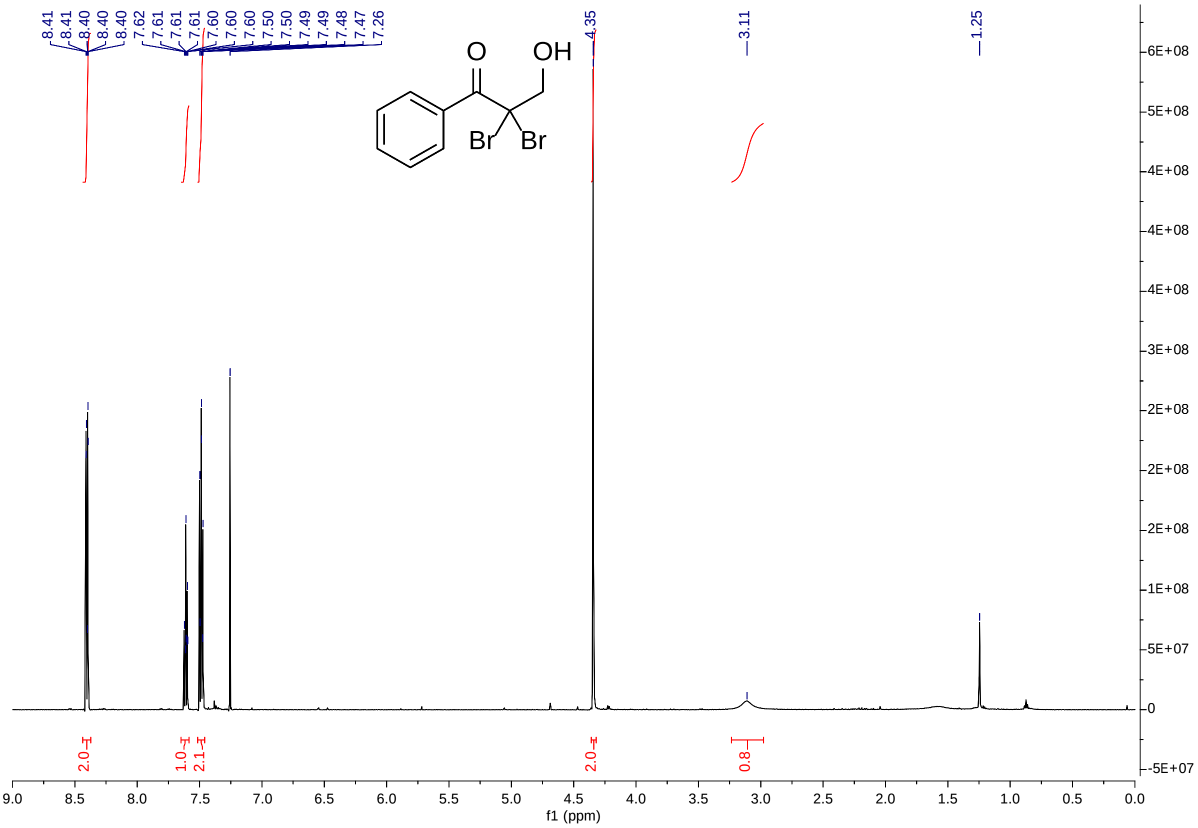

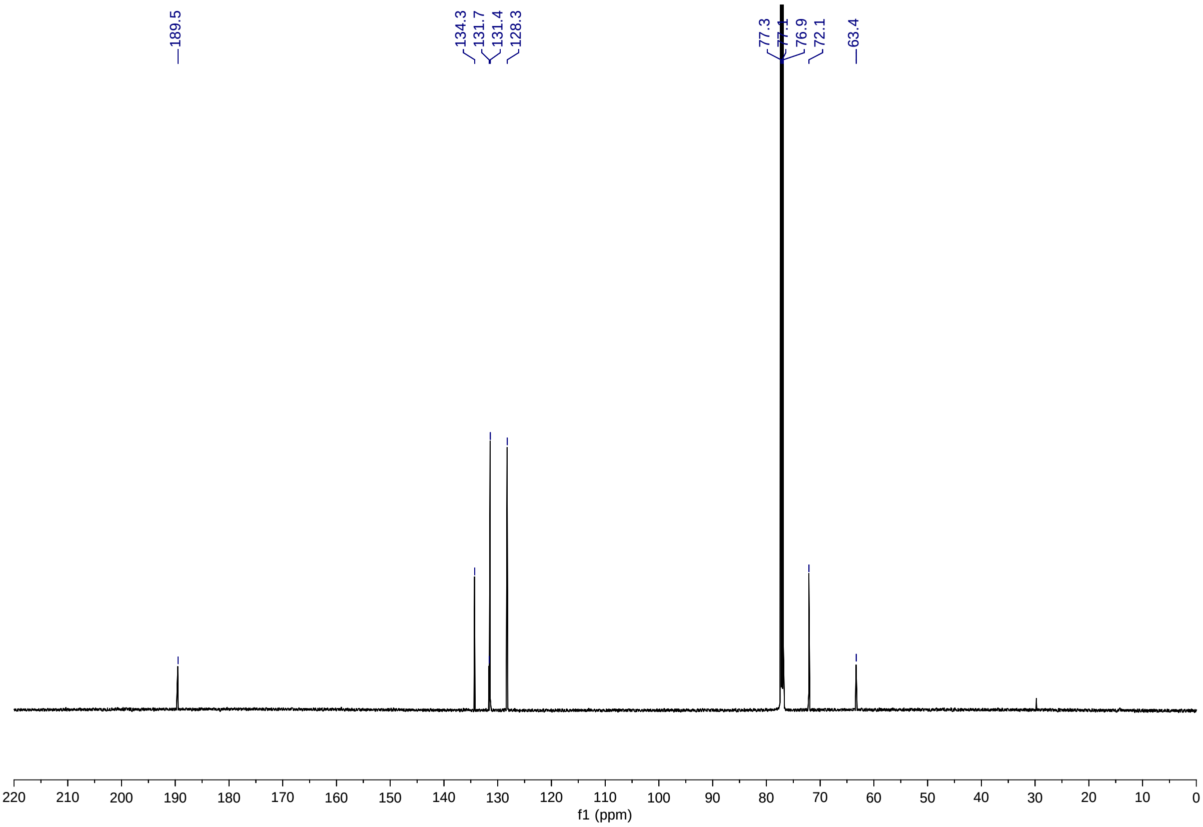

2,2-Dibromo-3-hydroxy-1-phenylhexan-1-one (6b)

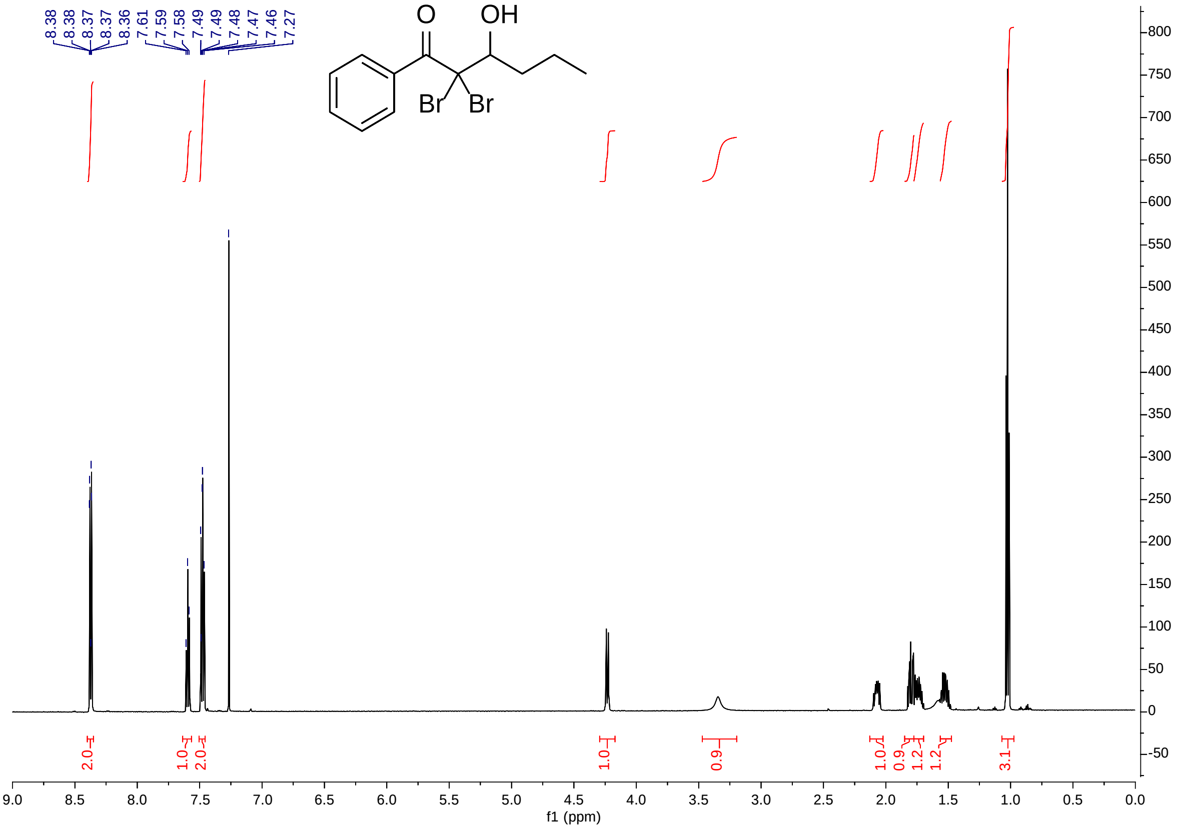

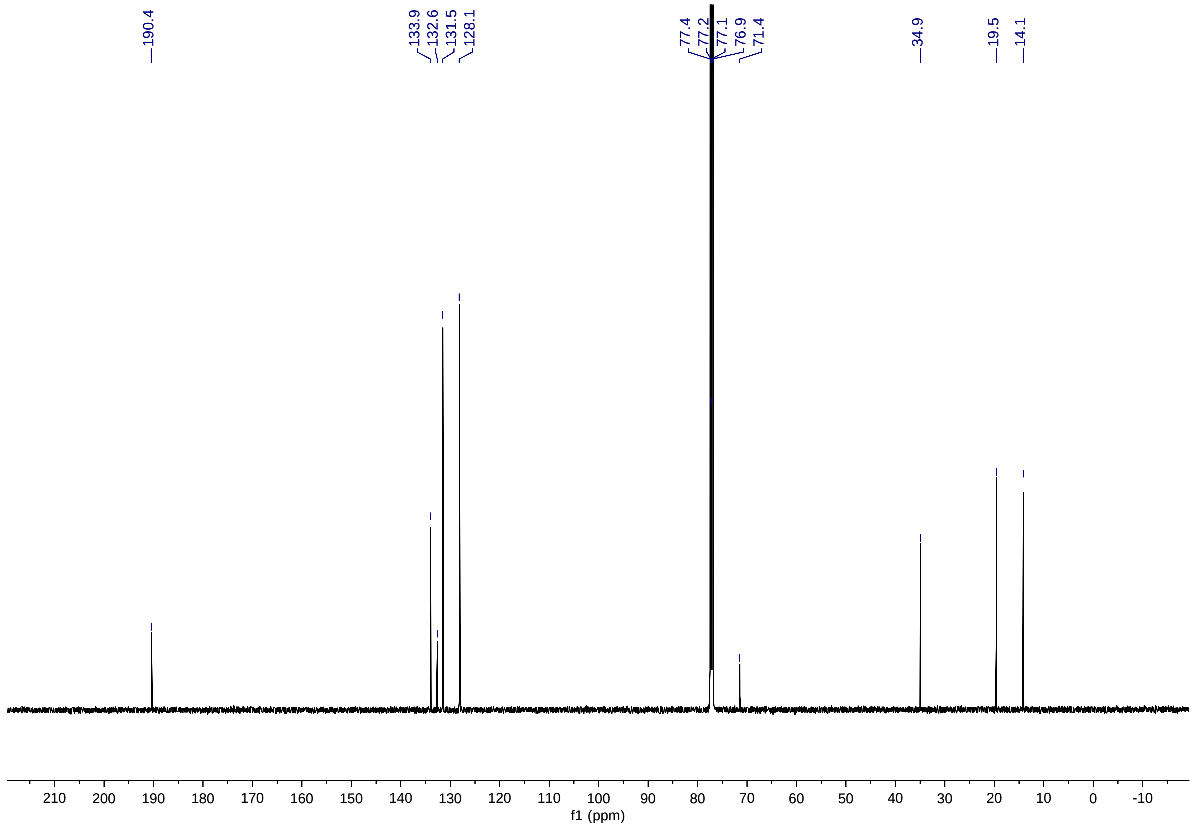

2,2-Dibromo-3-ethyl-3-hydroxy-1-(p-tolyl)pentan-1-one (6c)

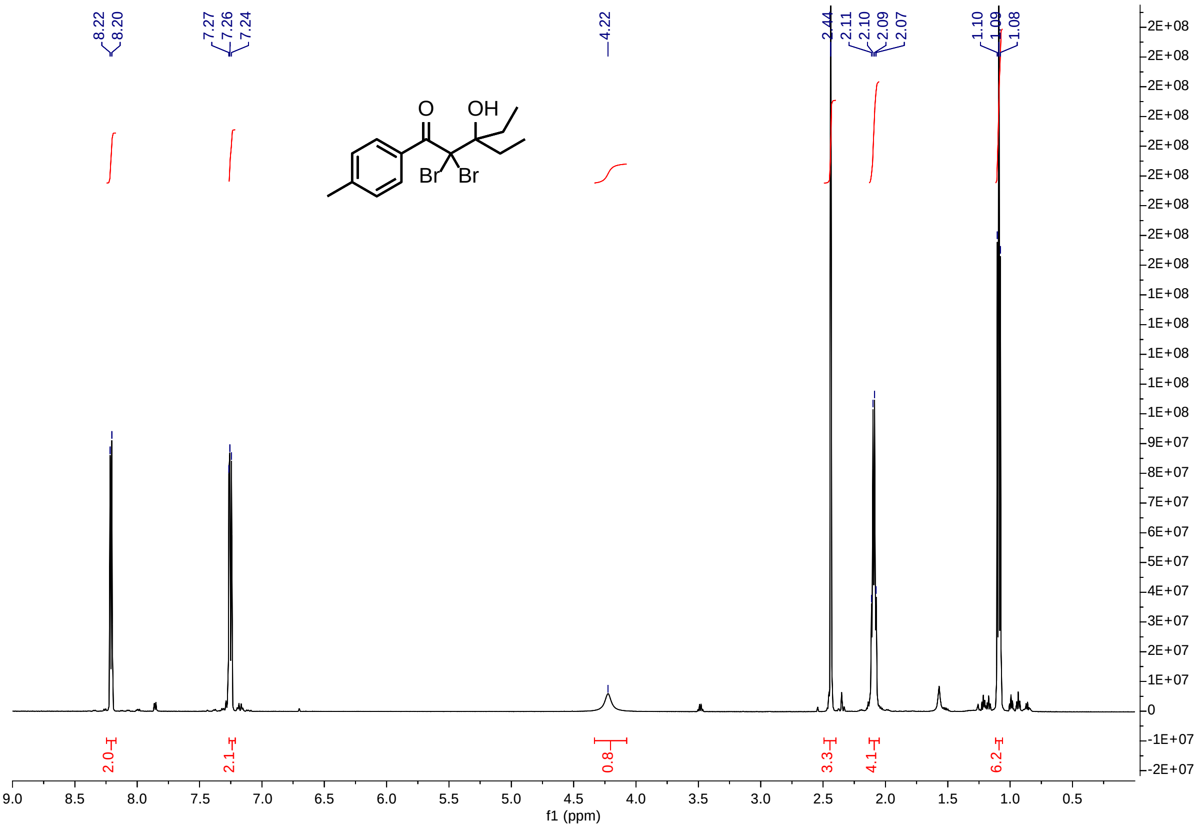

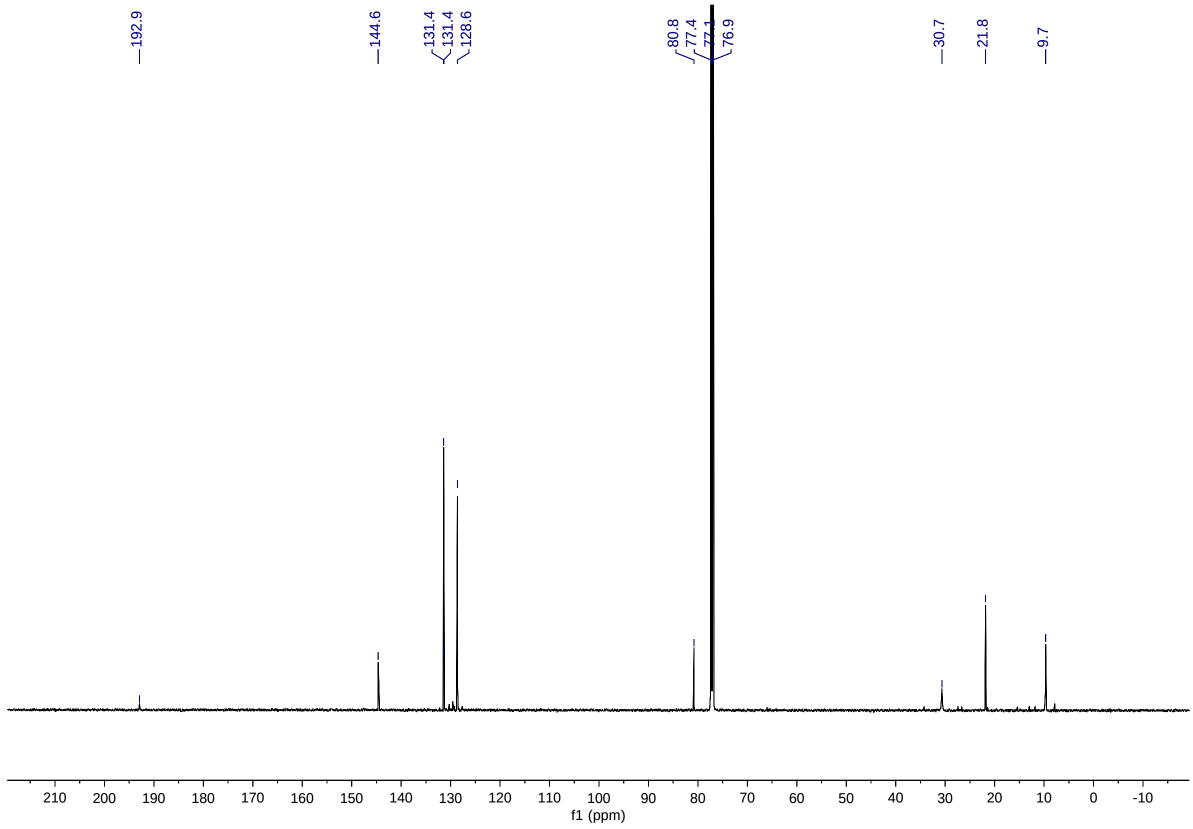

Ethyl 4,4-dibromo-3-hydroxy-5-oxo-5-(p-tolyl)pentanoate (6d)

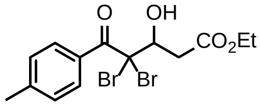

862a
63%*

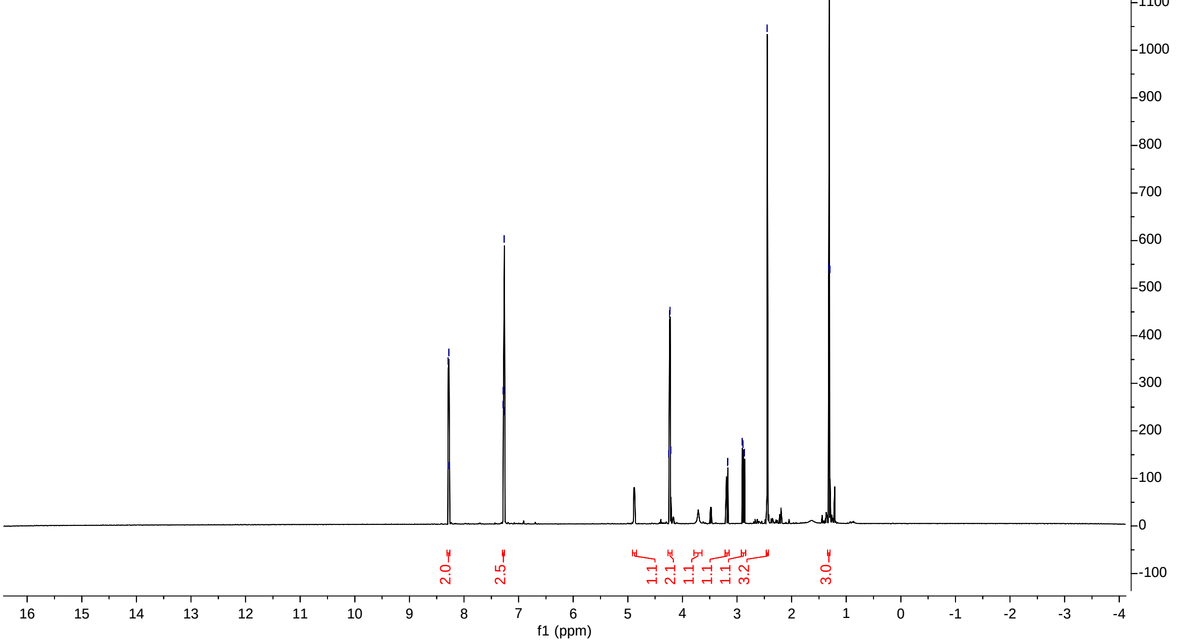

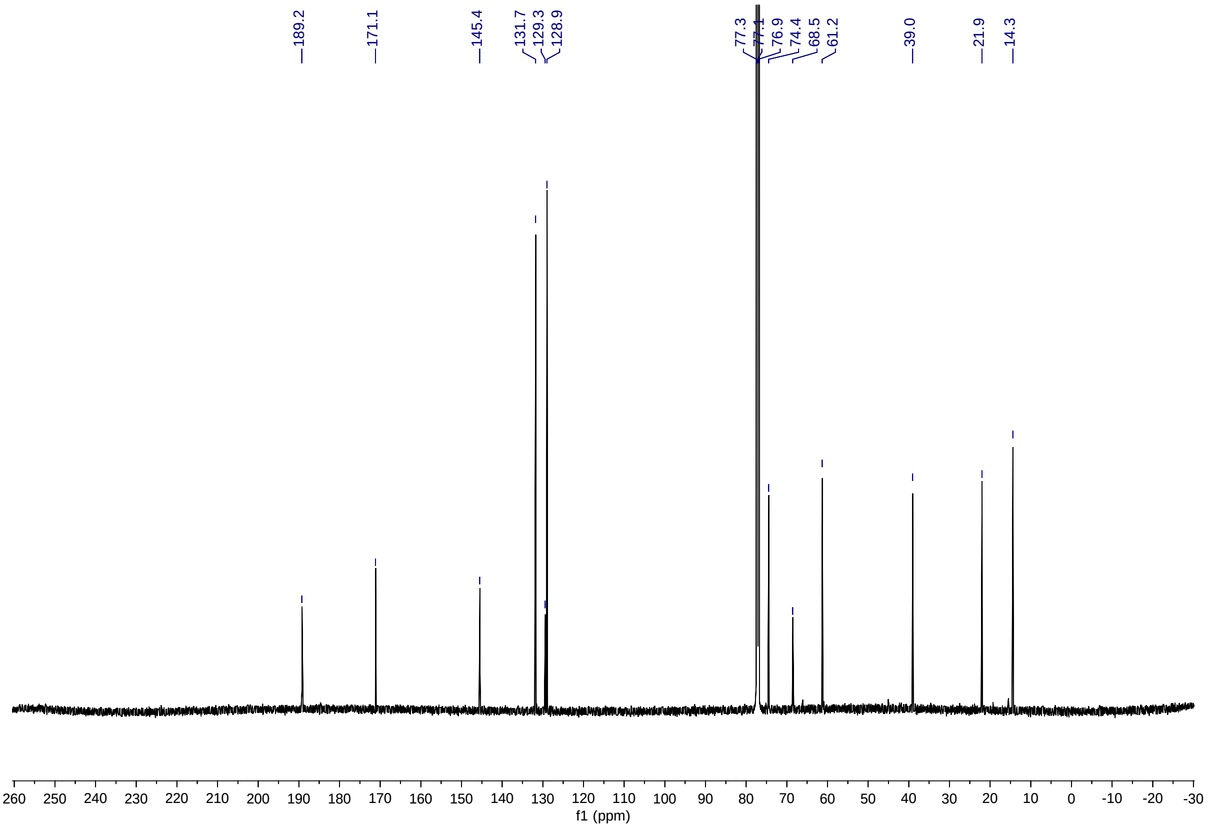

2,2-Dibromo-3-(4-bromophenyl)-3-hydroxy-1-phenylpropan-1-one (6e)

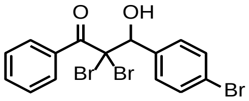

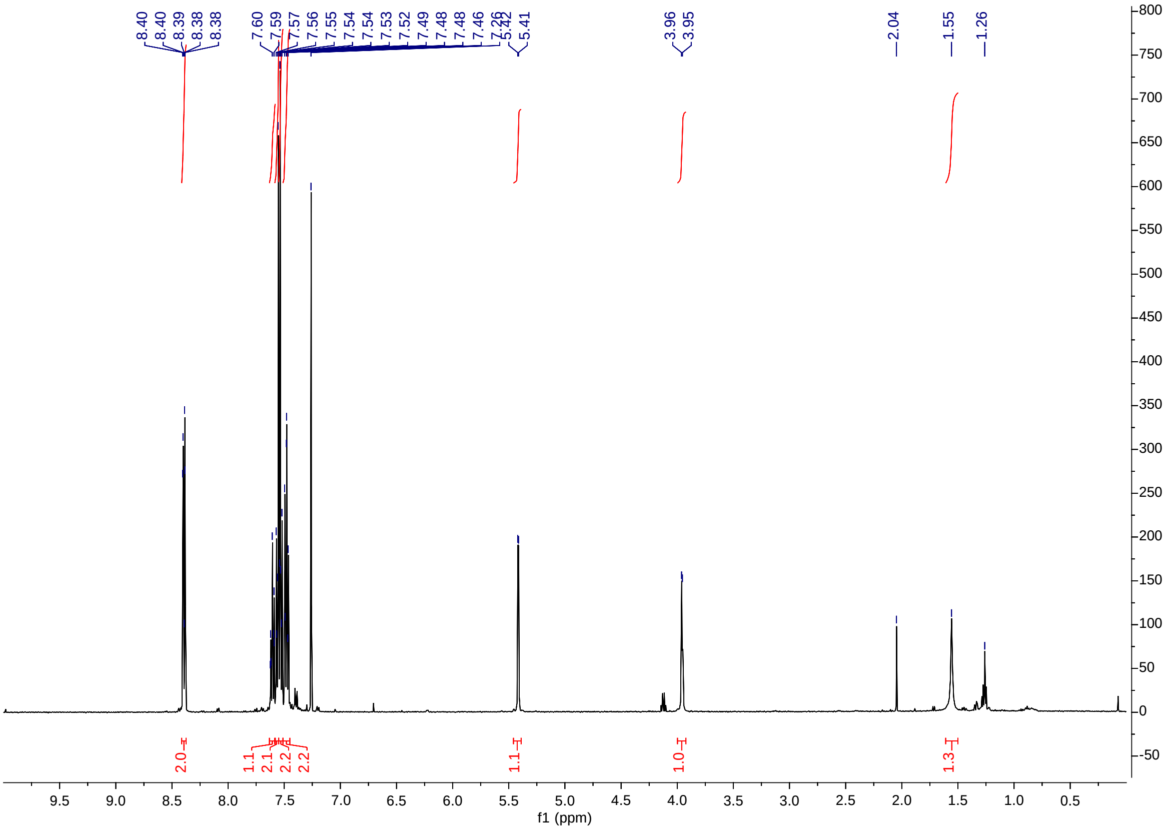

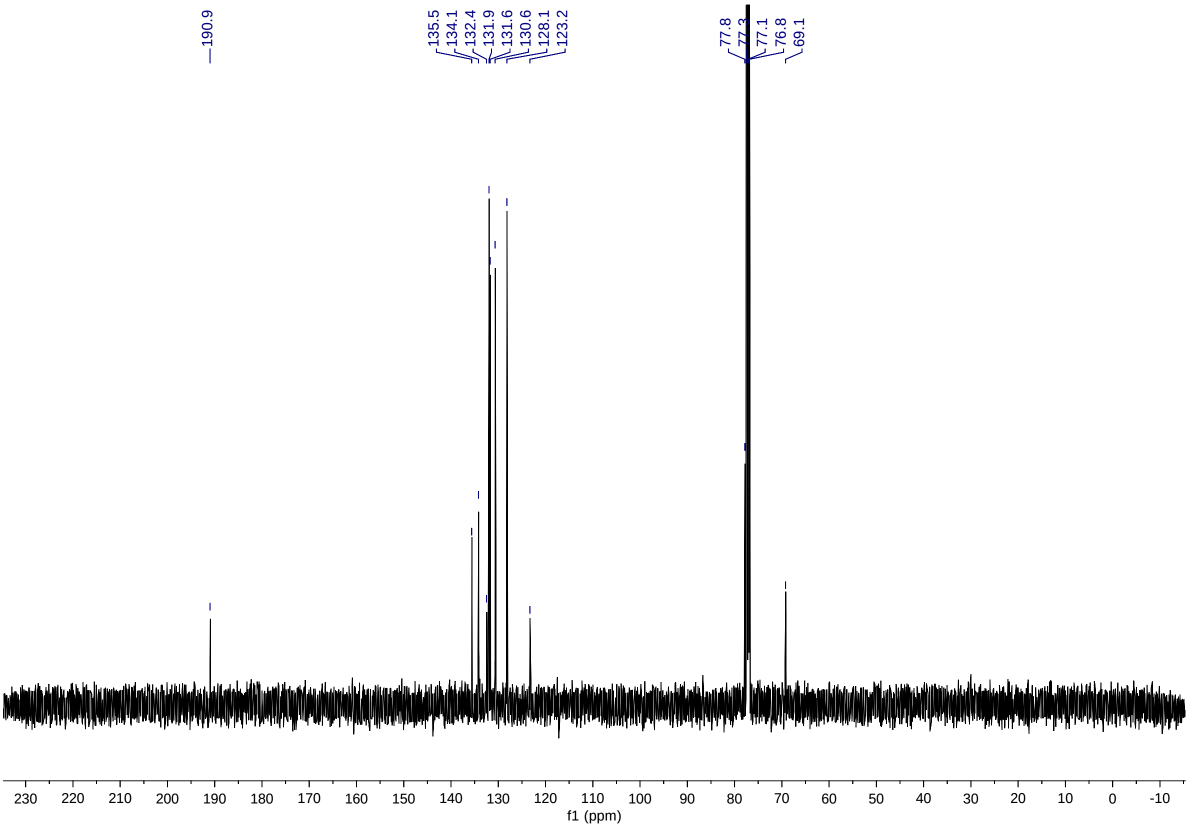

2,2-Dibromo-3-(4-bromophenyl)-3-hydroxy-1-(*p*-tolyl)propan-1-one (6f)

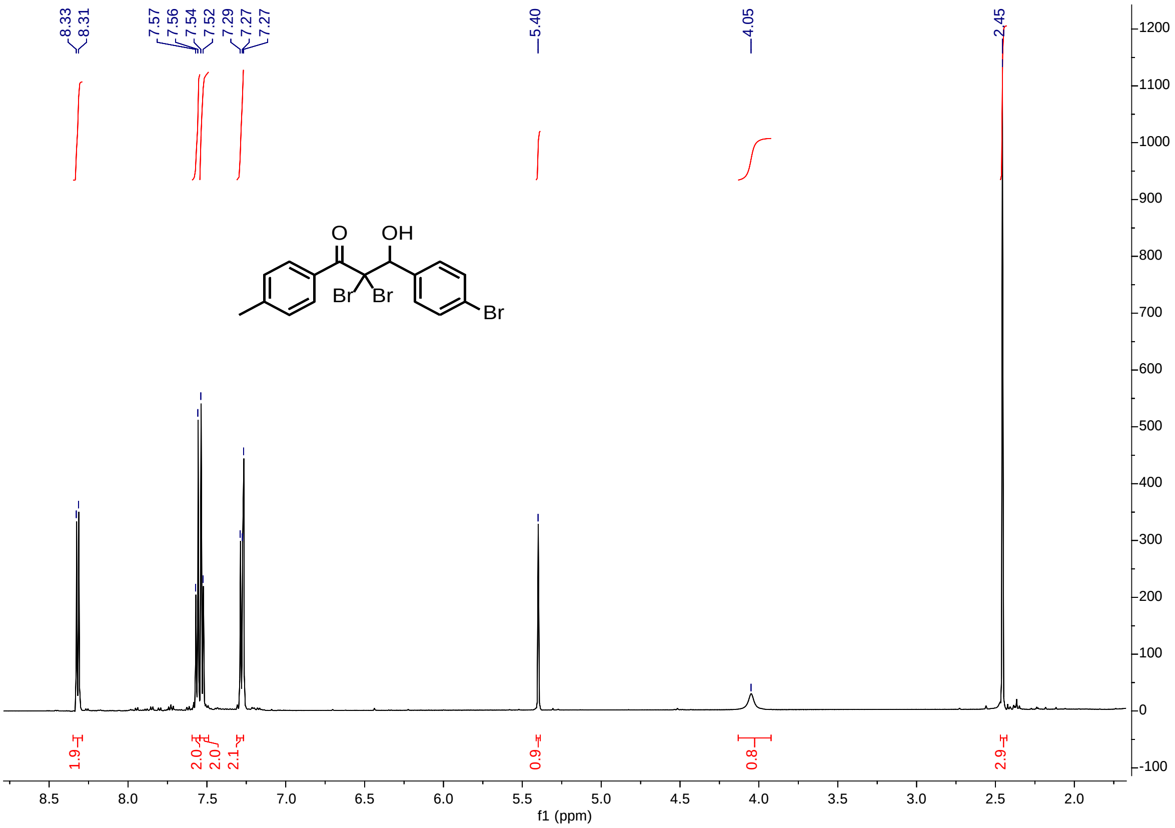

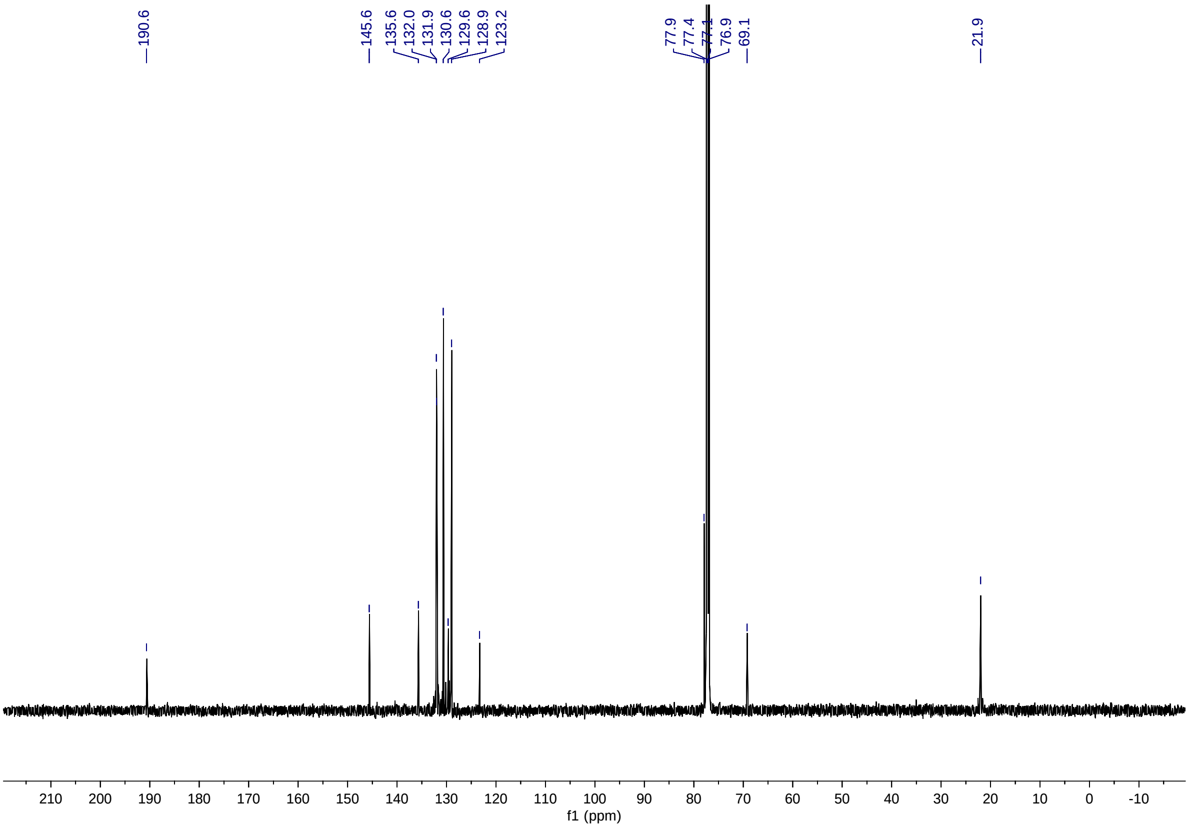

2,2-Dibromo-3-hydroxy-1-(*p*-tolyl)hexan-1-one (6g)

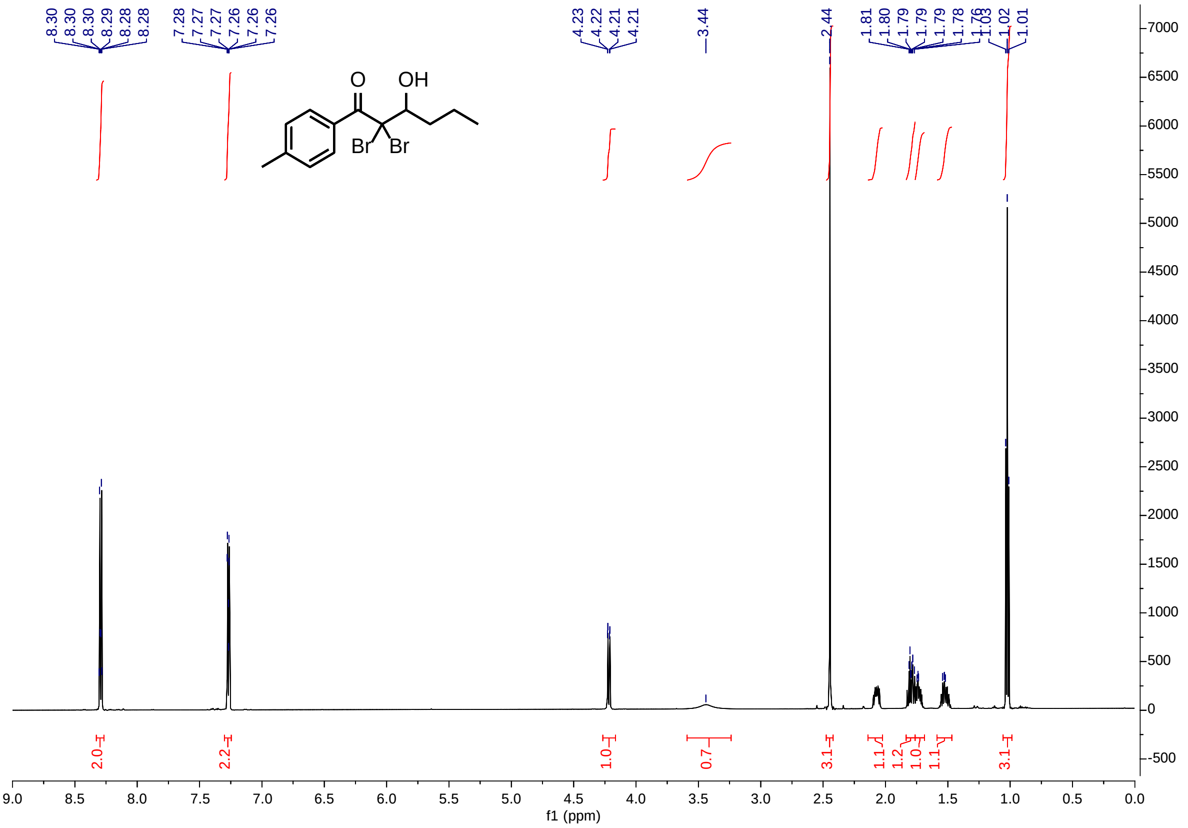

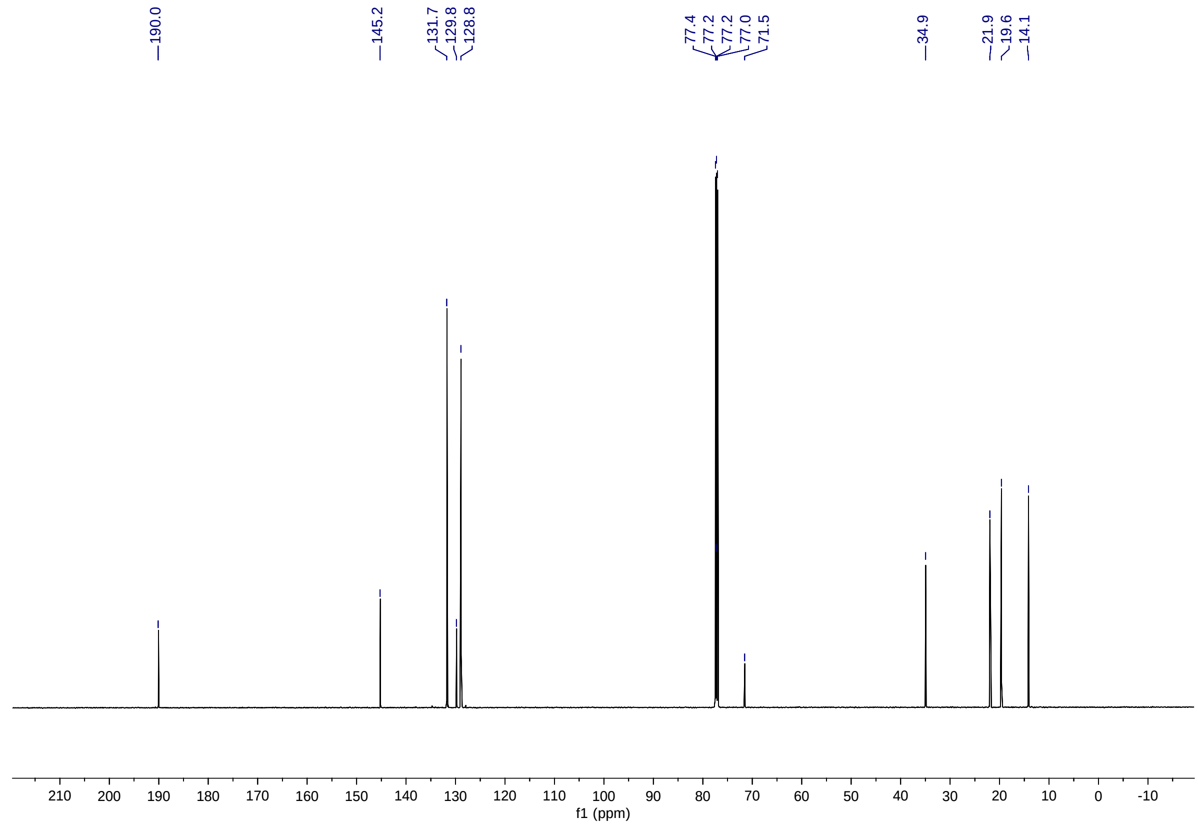

2,2-dibromo-3-hydroxy-1-(o-tolyl)hexan-1-one (6h)

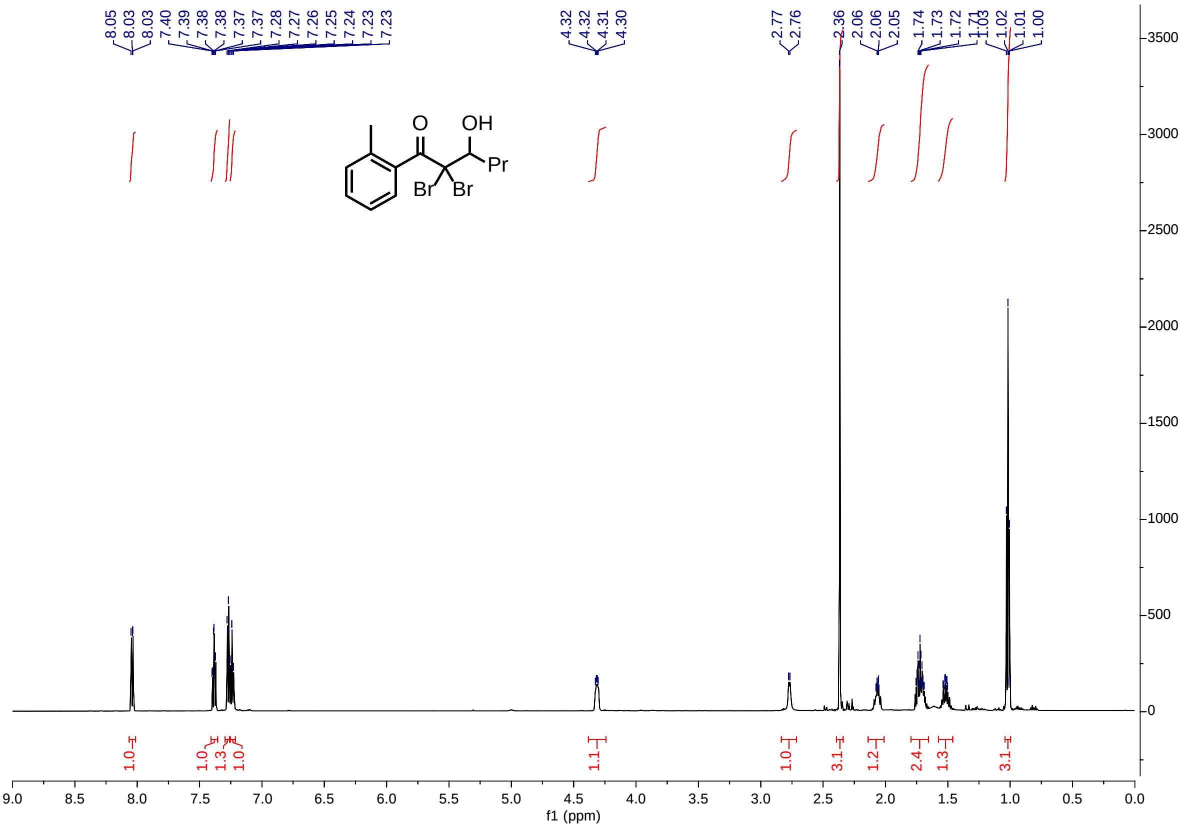

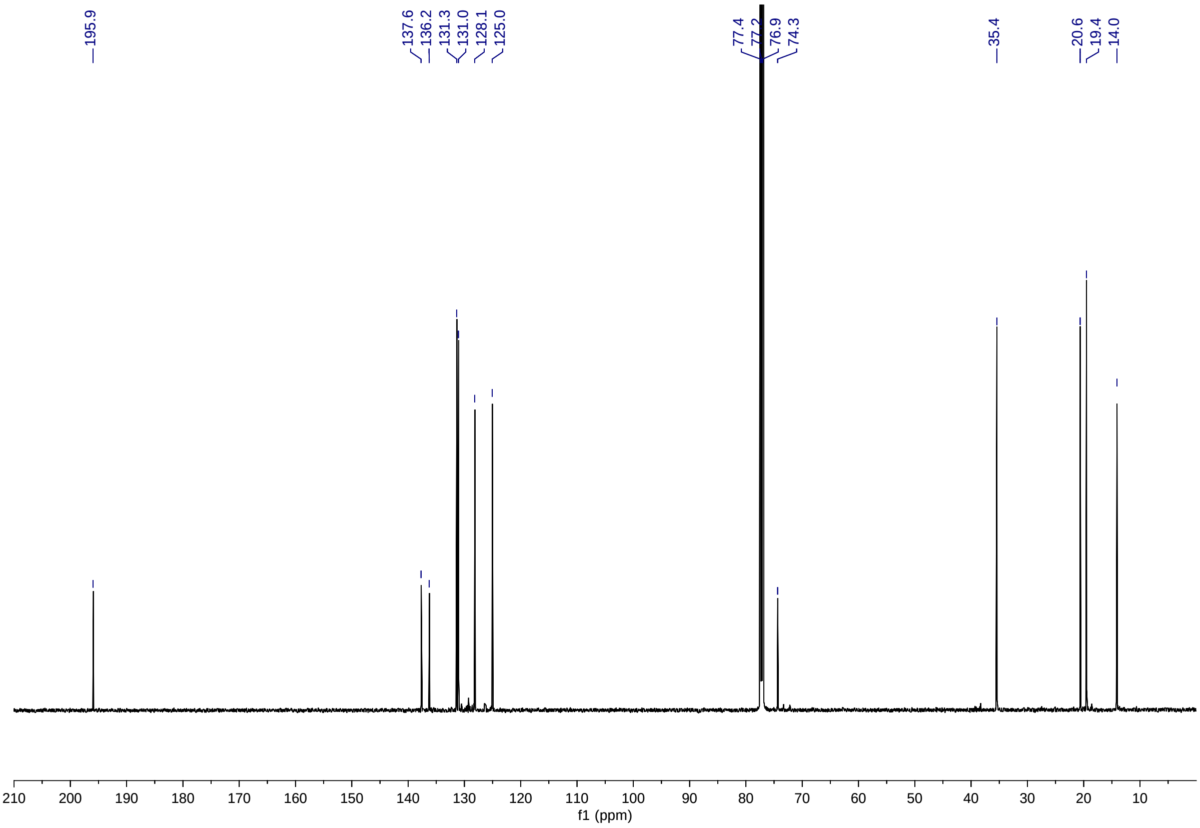

2,2-Dibromo-3-hydroxy-1-(phenanthren-9-yl)hexan-1-one (6i)

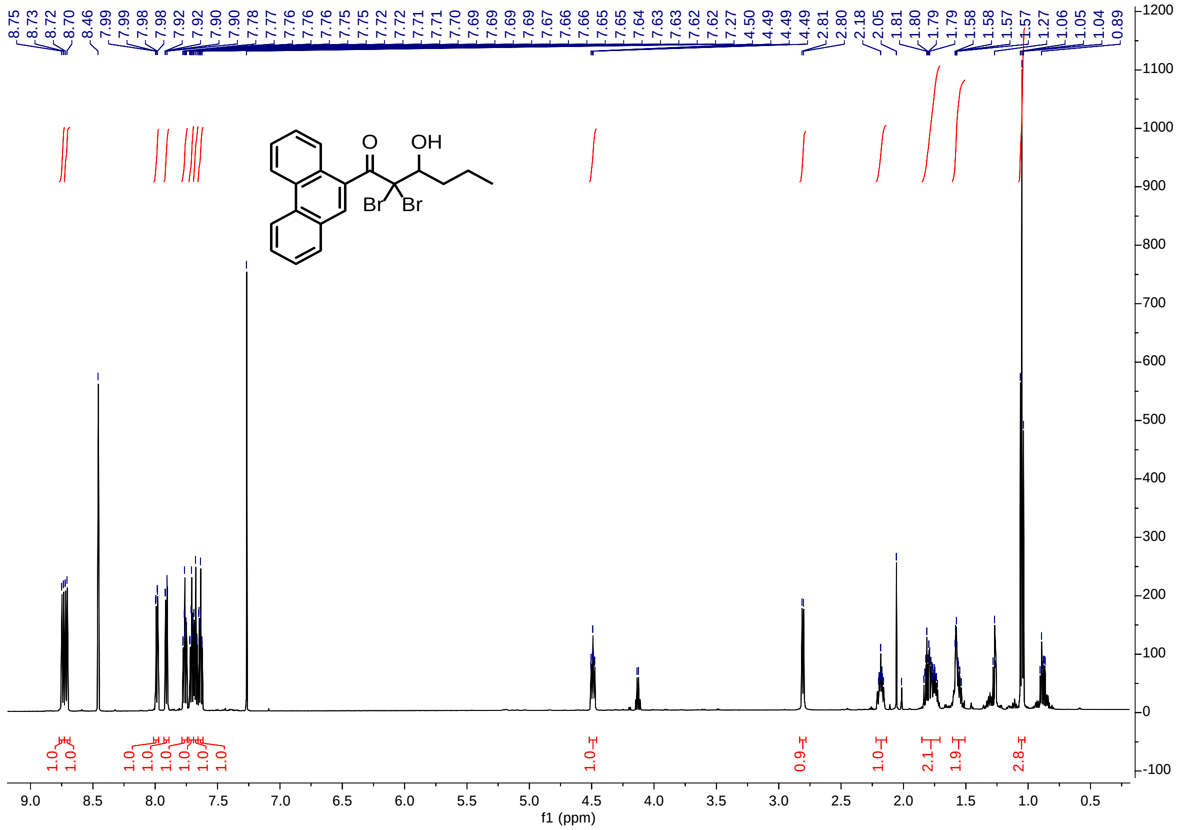

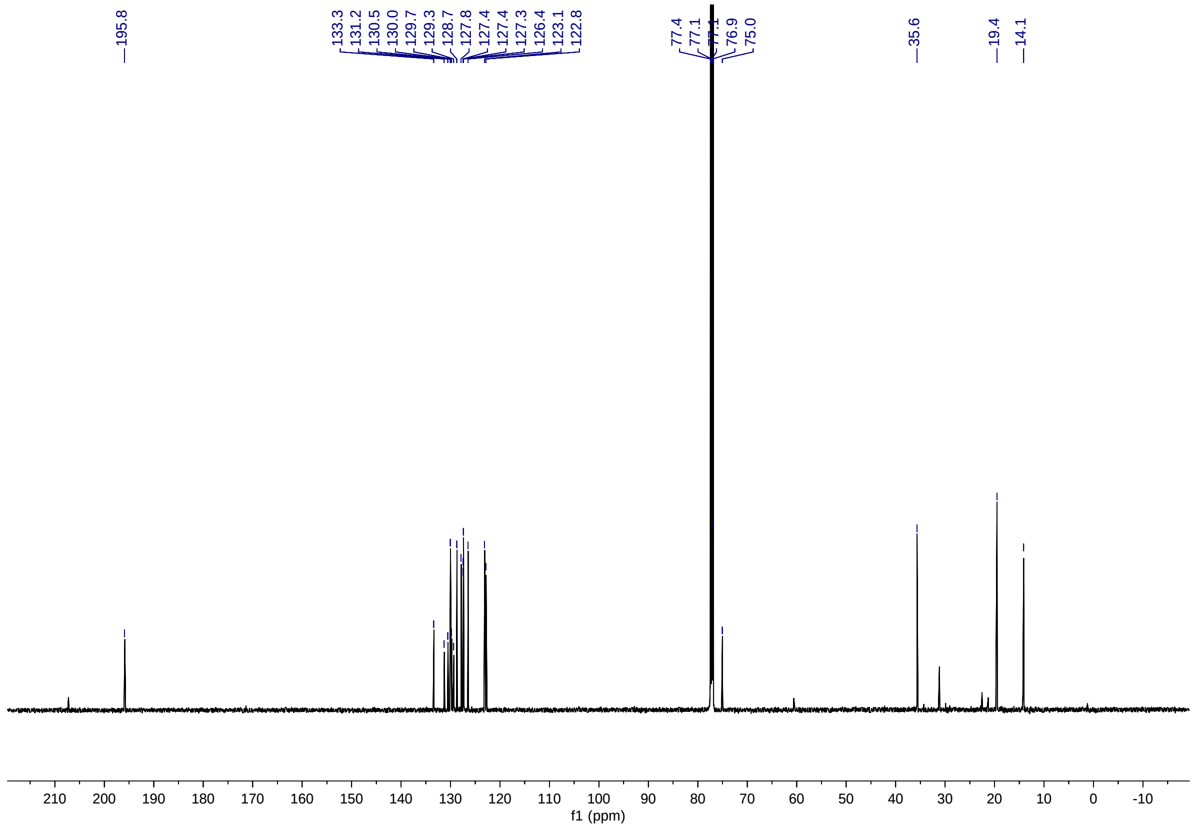

2,2-Dibromo-3-hydroxy-1-(4-methoxyphenyl)hexan-1-one (6j)

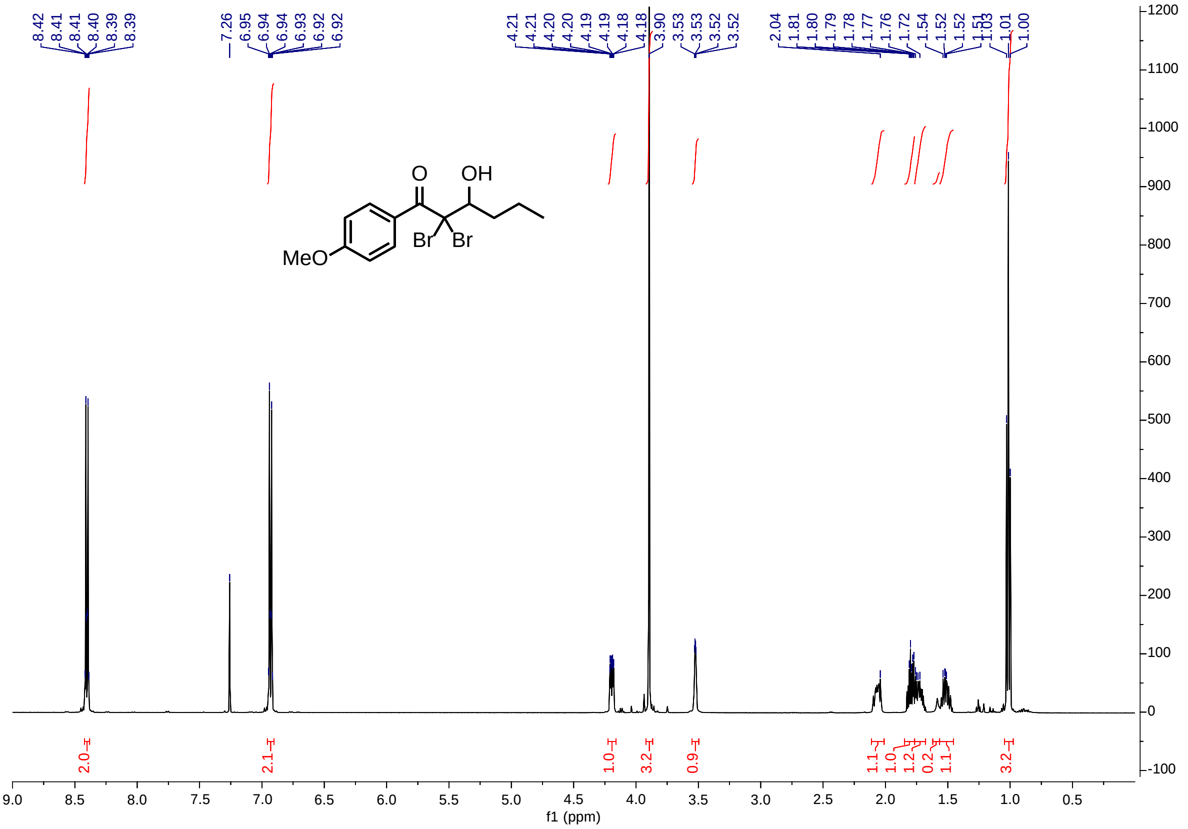

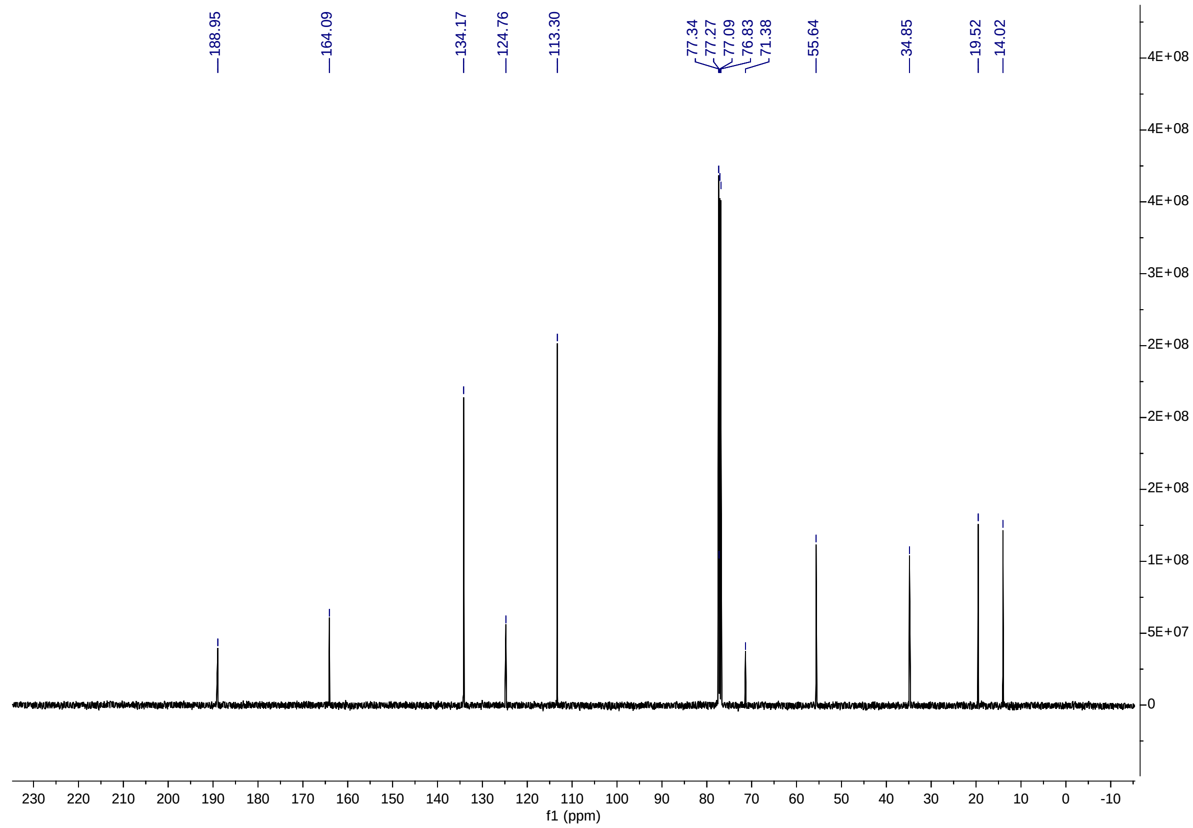

2,2-Dibromo-3-hydroxy-1-(pyridin-3-yl)hexan-1-one (6k)

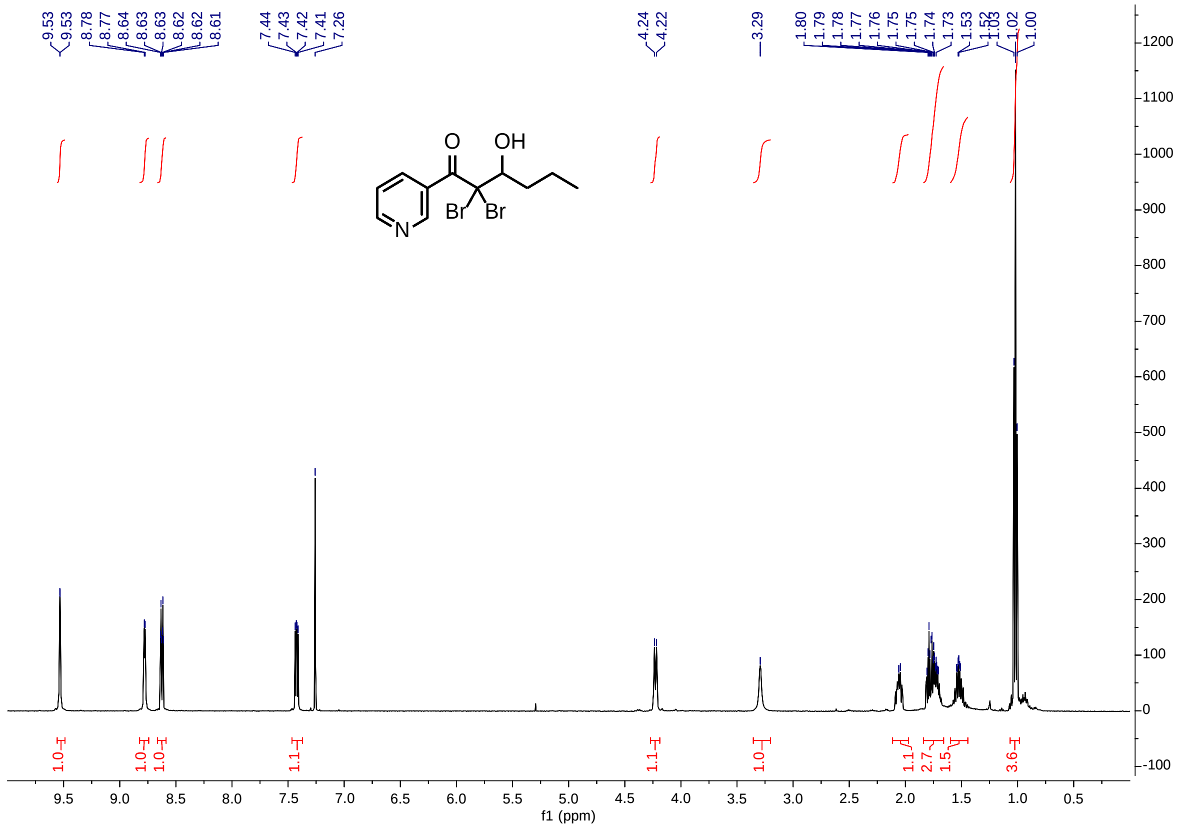

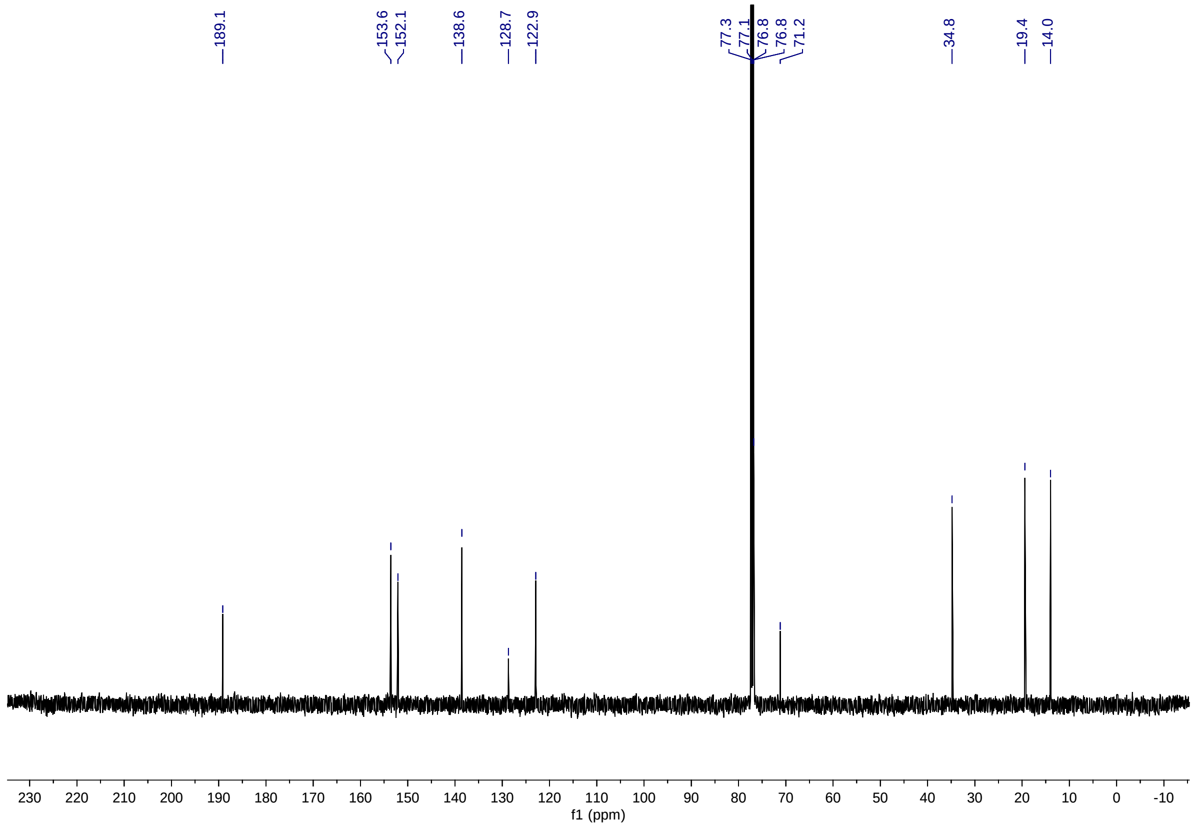

2,2-Dibromo-3-hydroxy-1-(4-(trifluoromethyl)phenyl)hexan-1-one (6l)

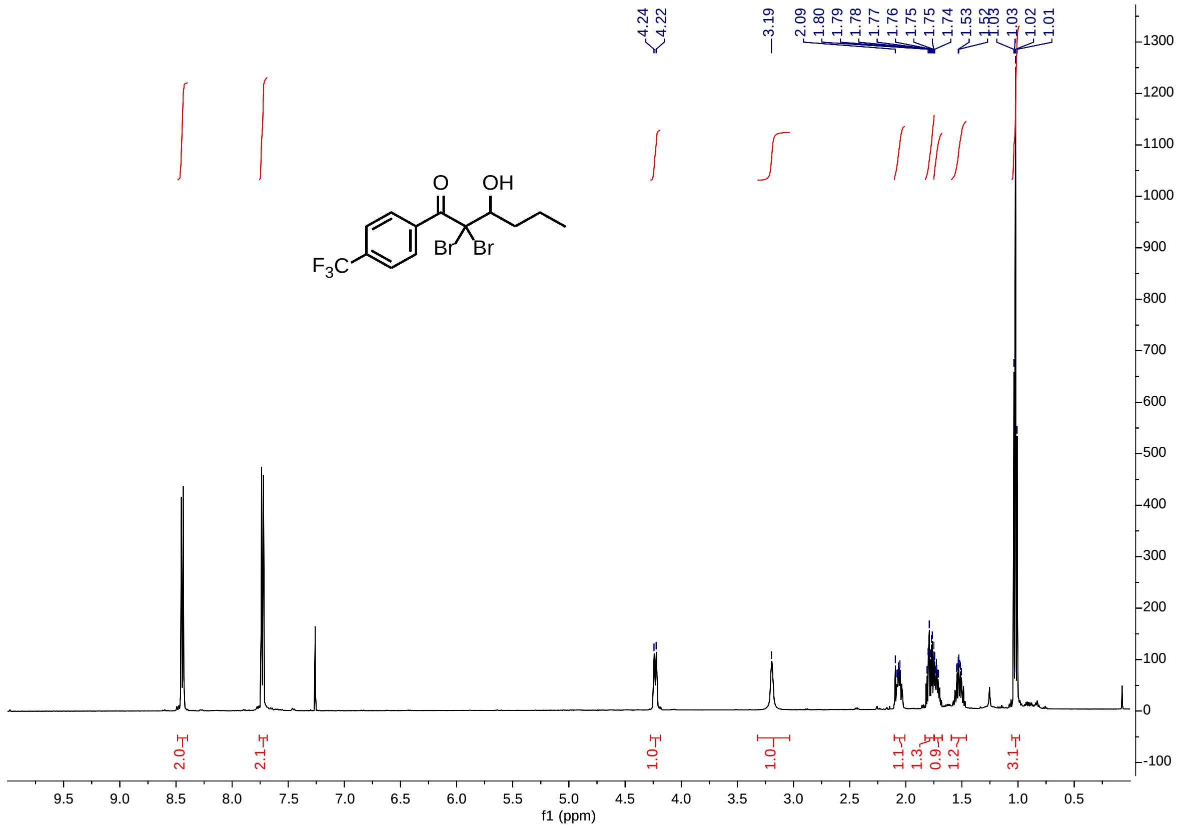

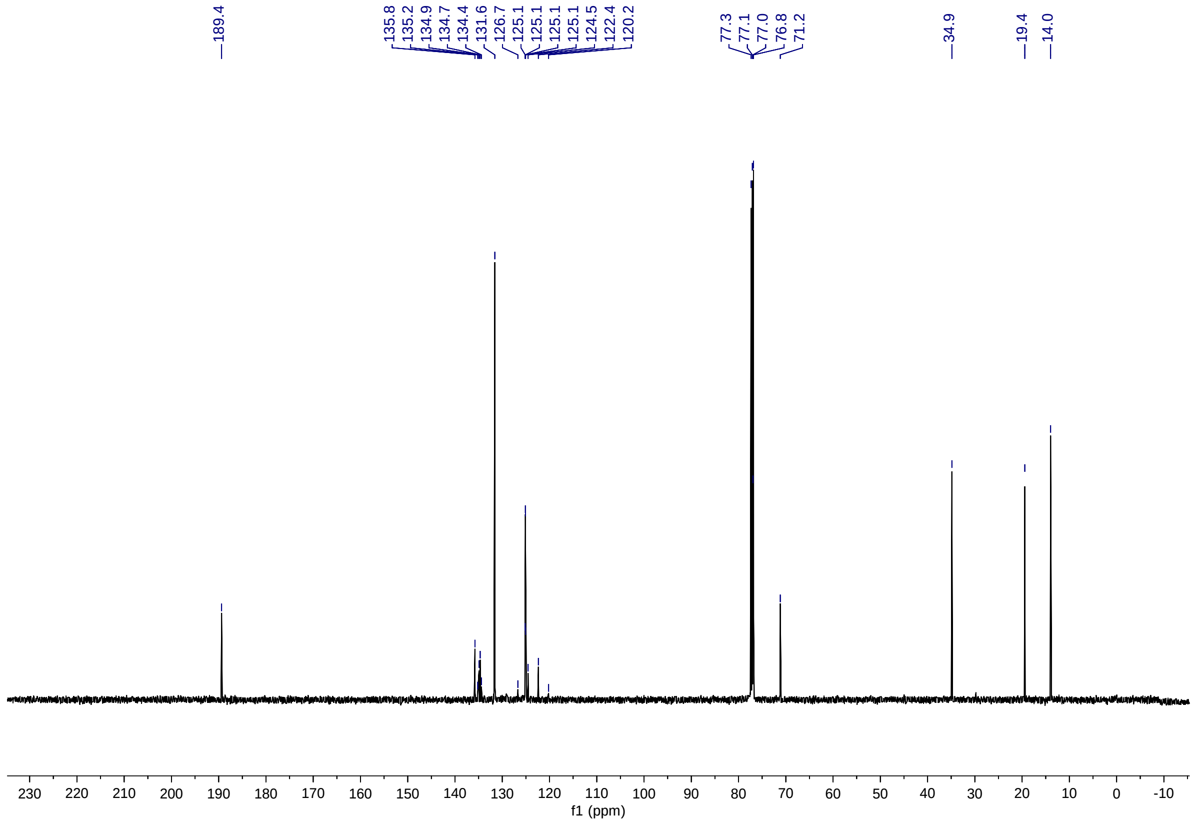

2,2-Dibromo-3-hydroxy-1-(3-methoxyphenyl)hexan-1-one (6m)

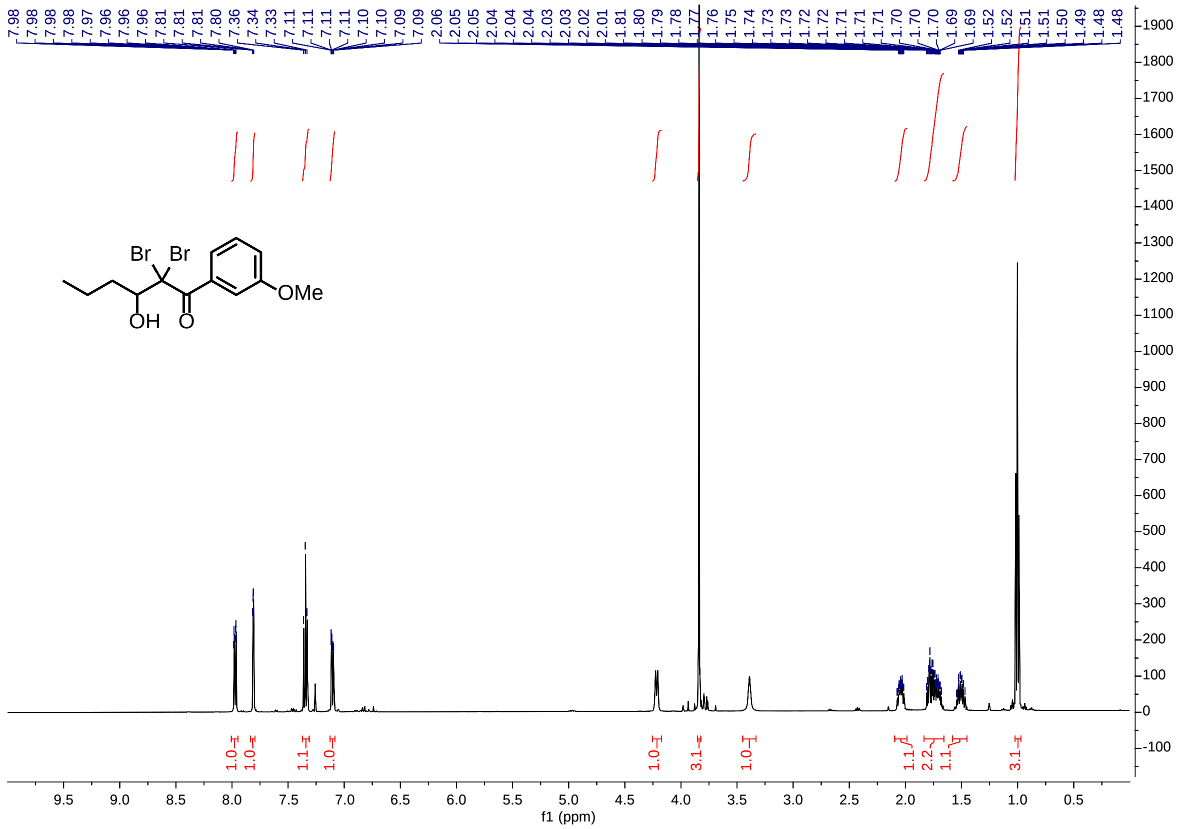

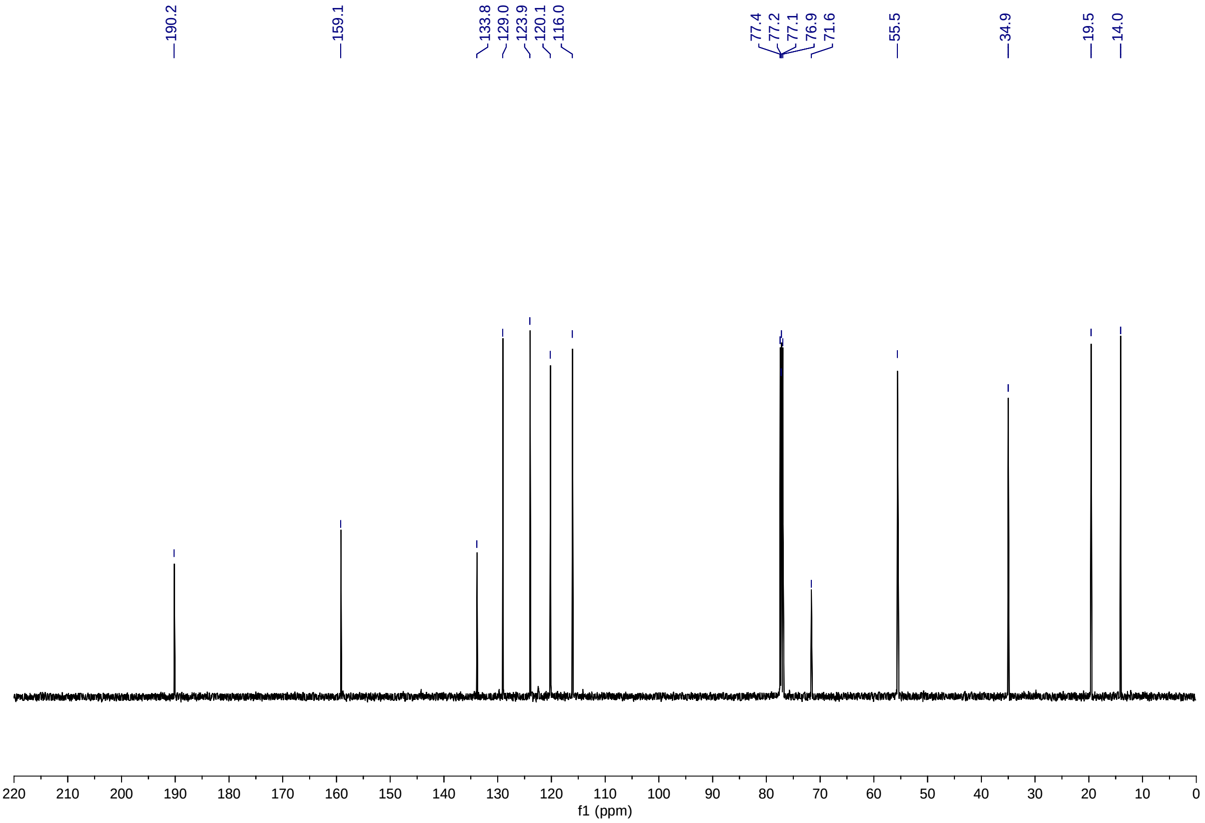

2,2-dibromo-3-hydroxy-4methyl-1-phenylpentan-1-one (6n)

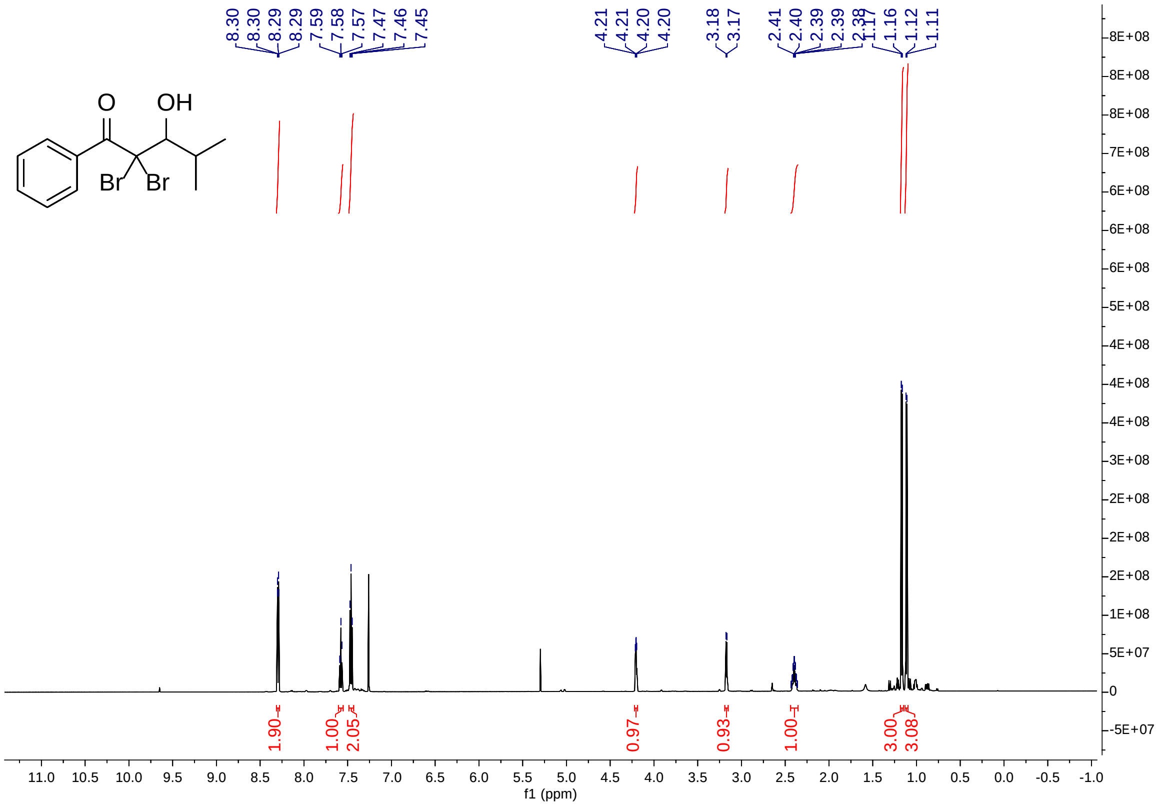

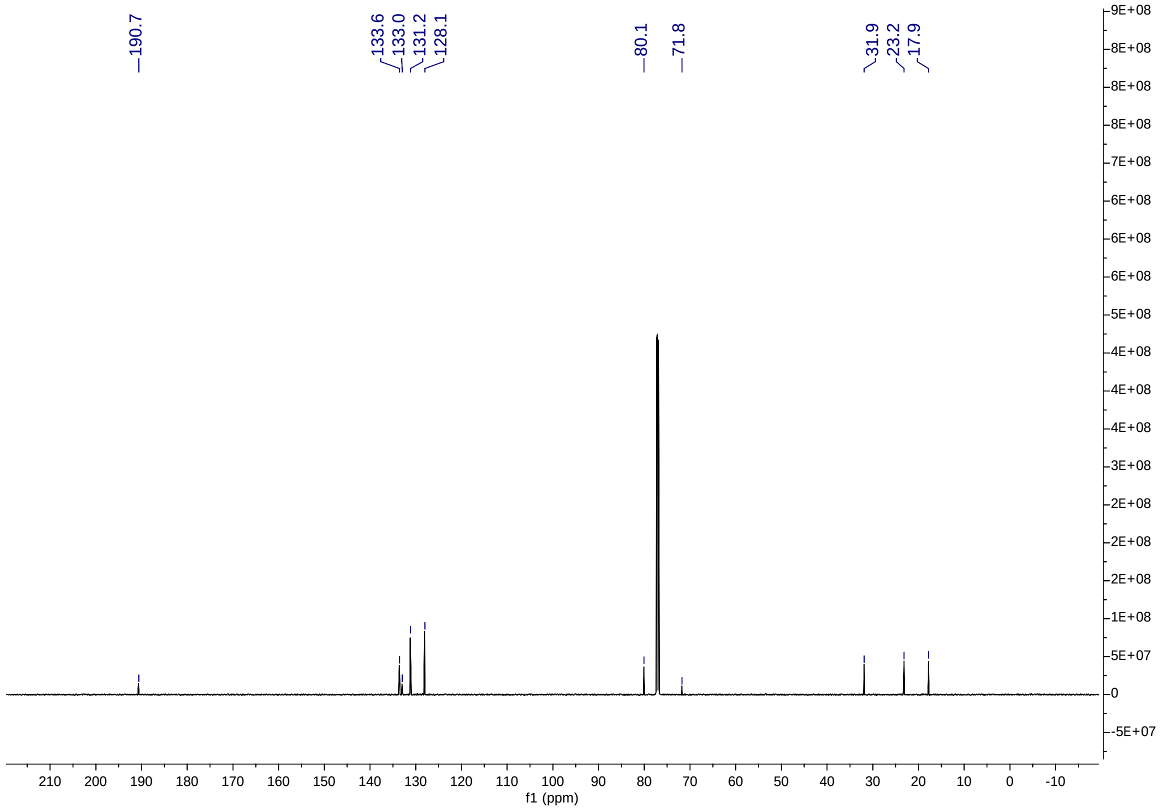

Ethyl 2,2-dibromo-3-hydroxy-5-phenylpentanoate (6o)

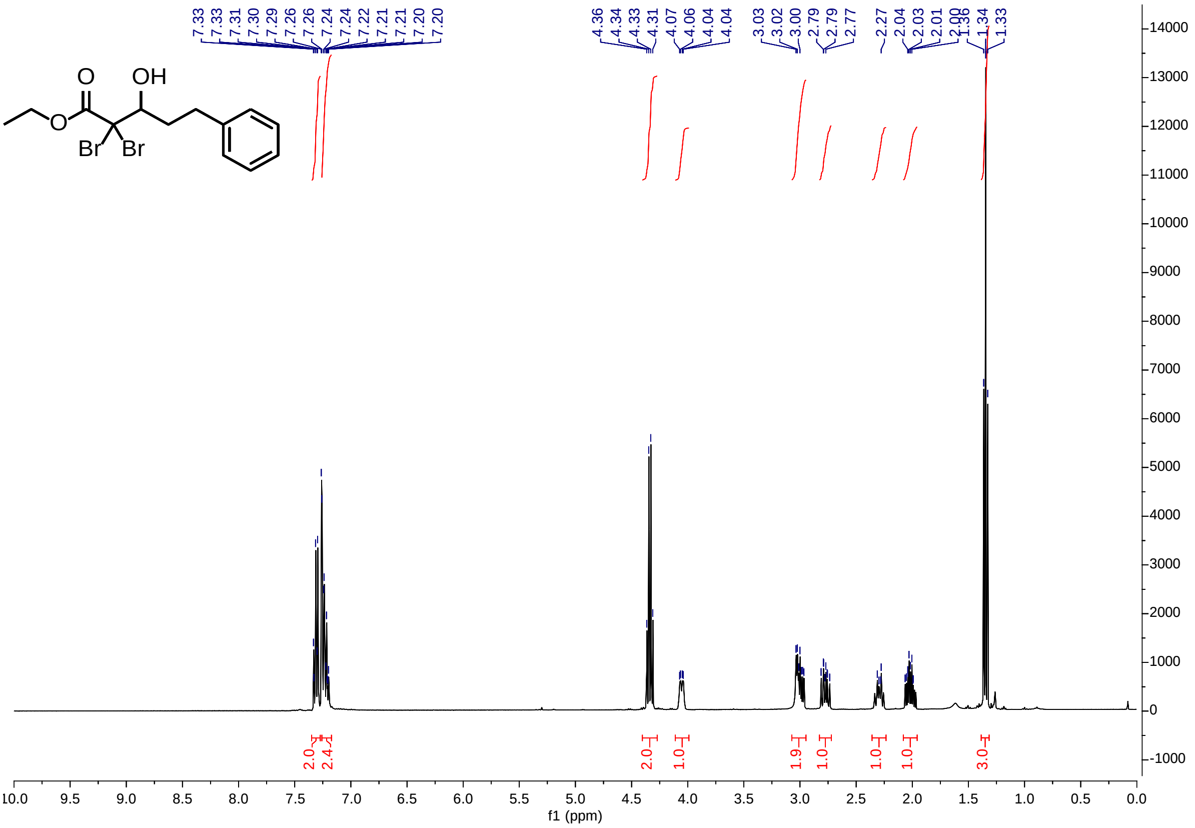

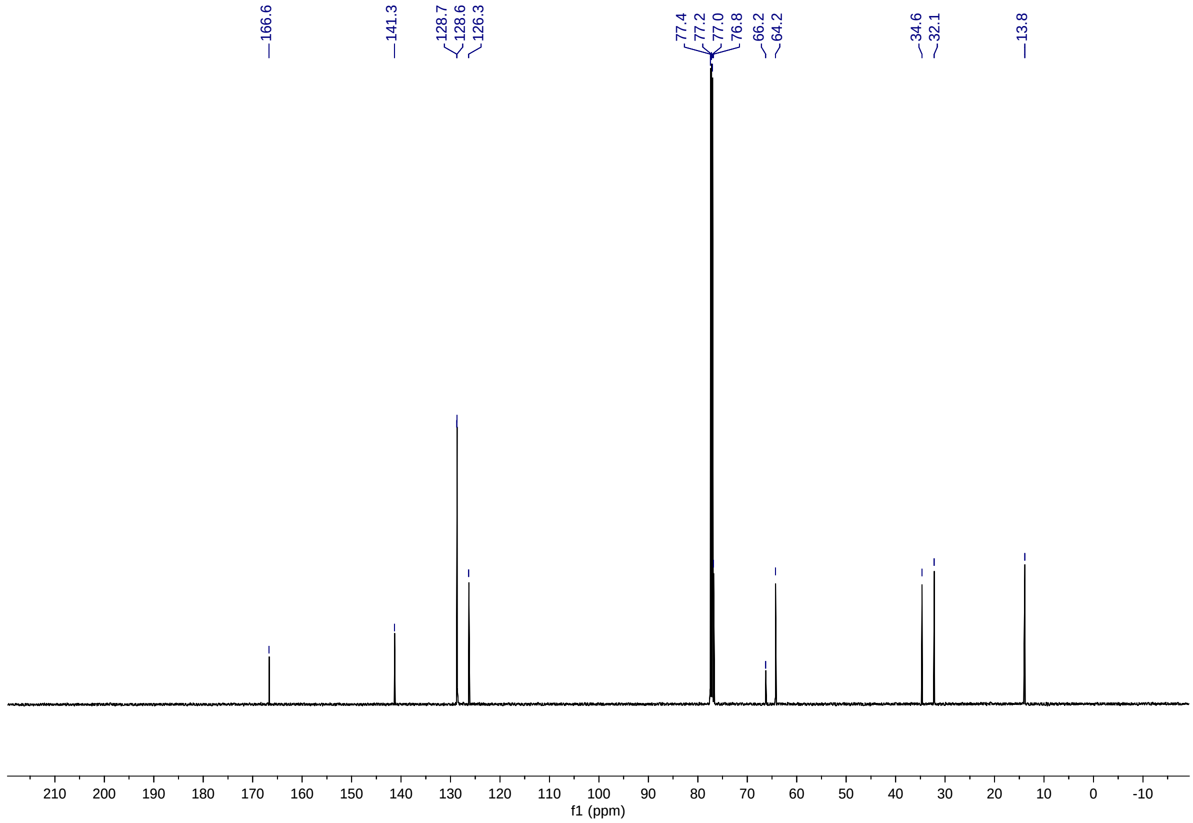

2,2-Dichloro-3-hydroxy-4-methyl-1-phenylpentan-1-one (7a)

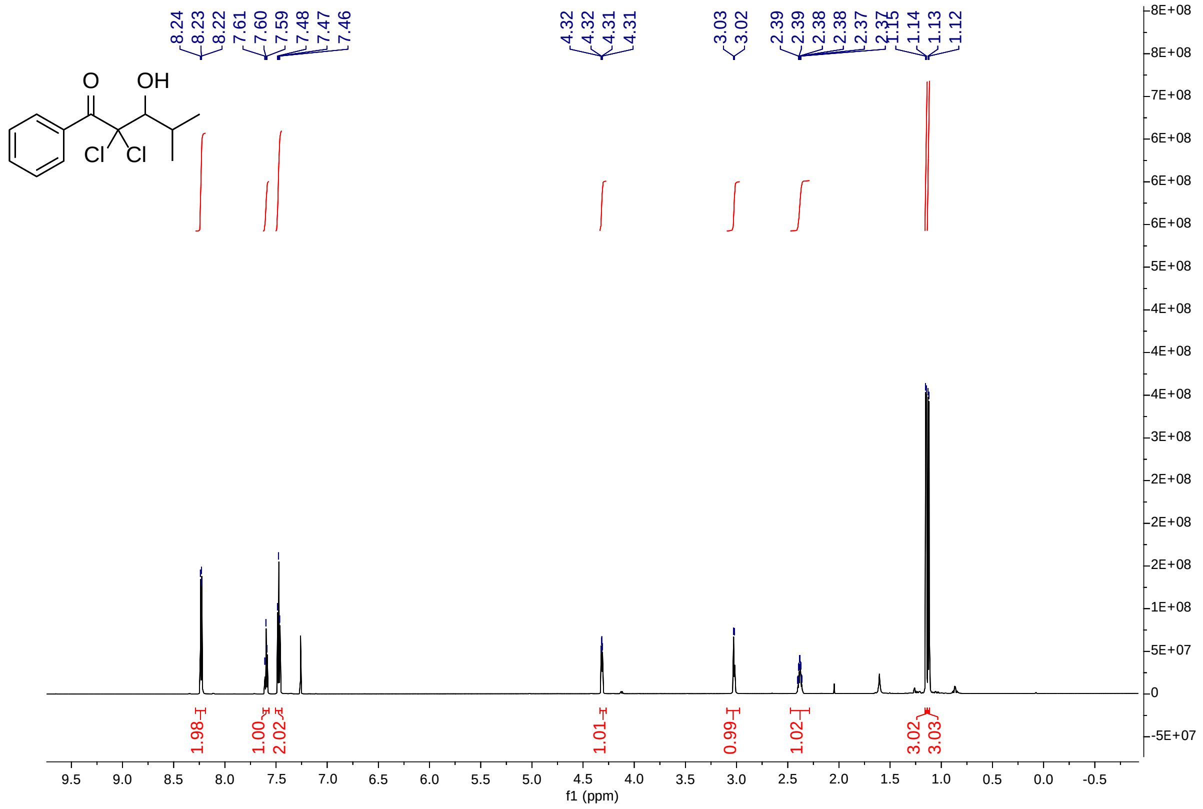

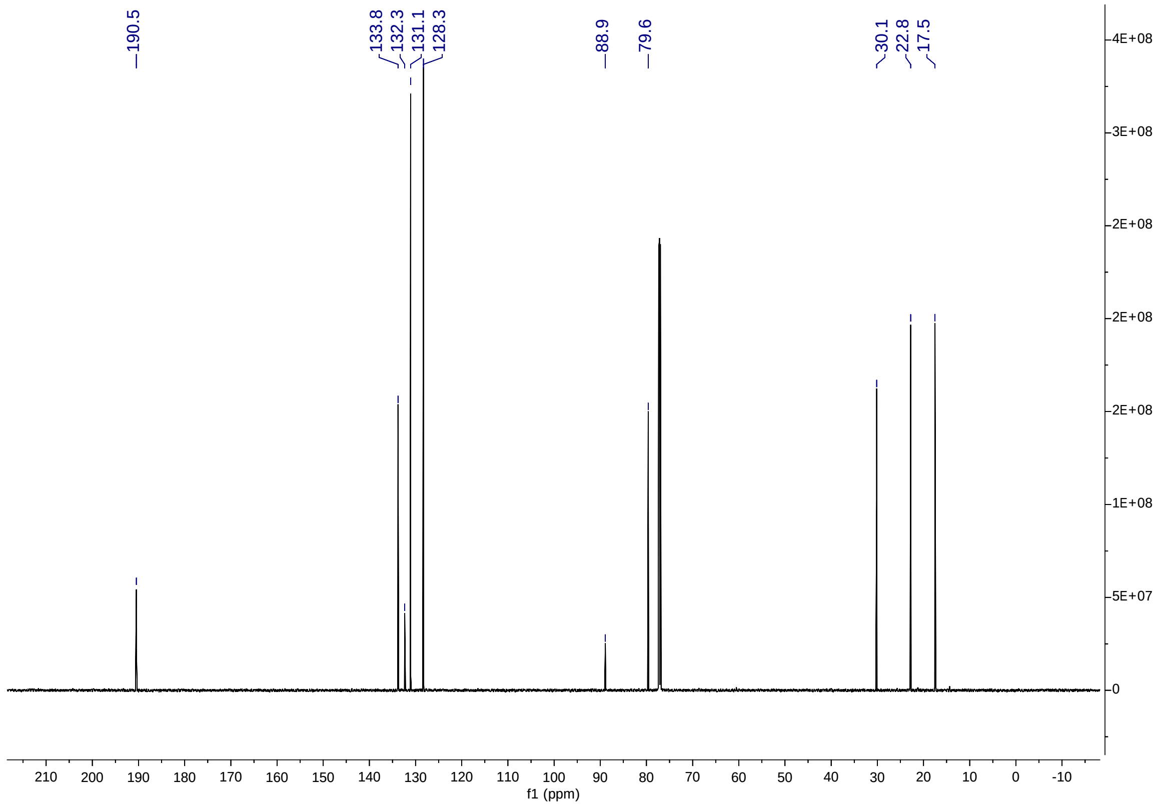

2,2-Dichloro-3-hydroxy-1,3-diphenylpropan-1-one (7b)

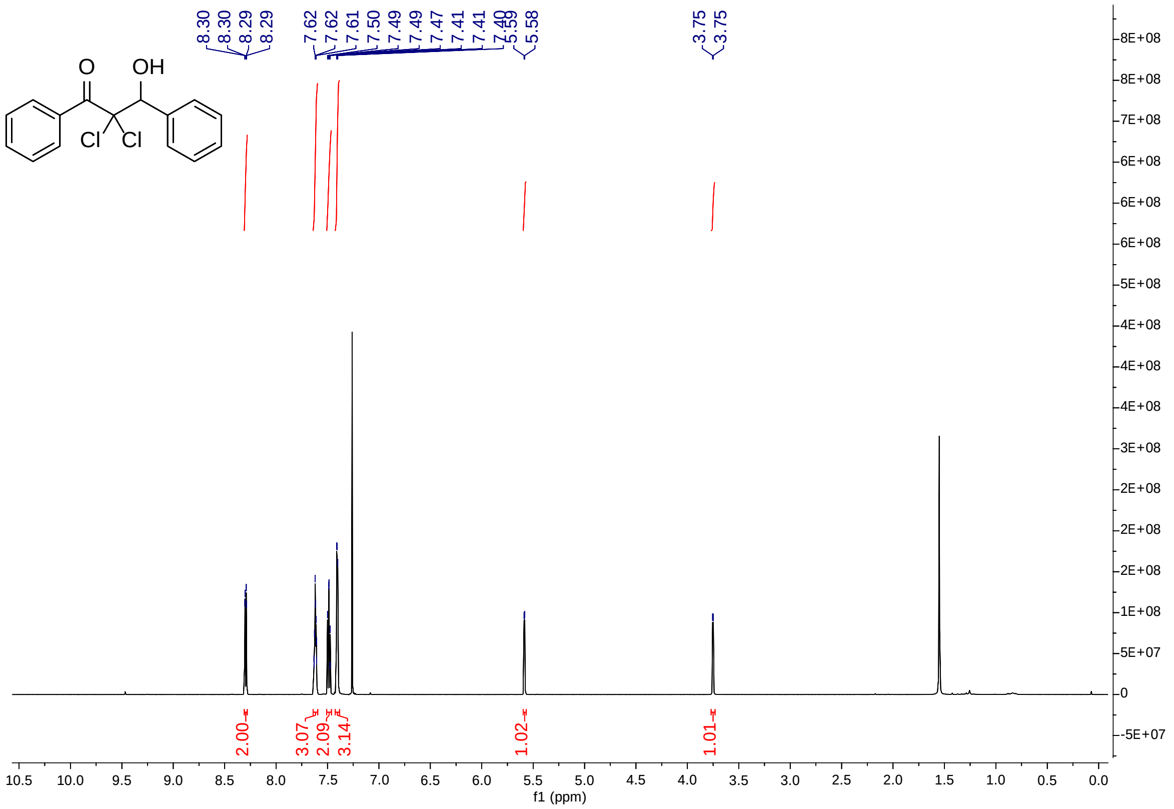

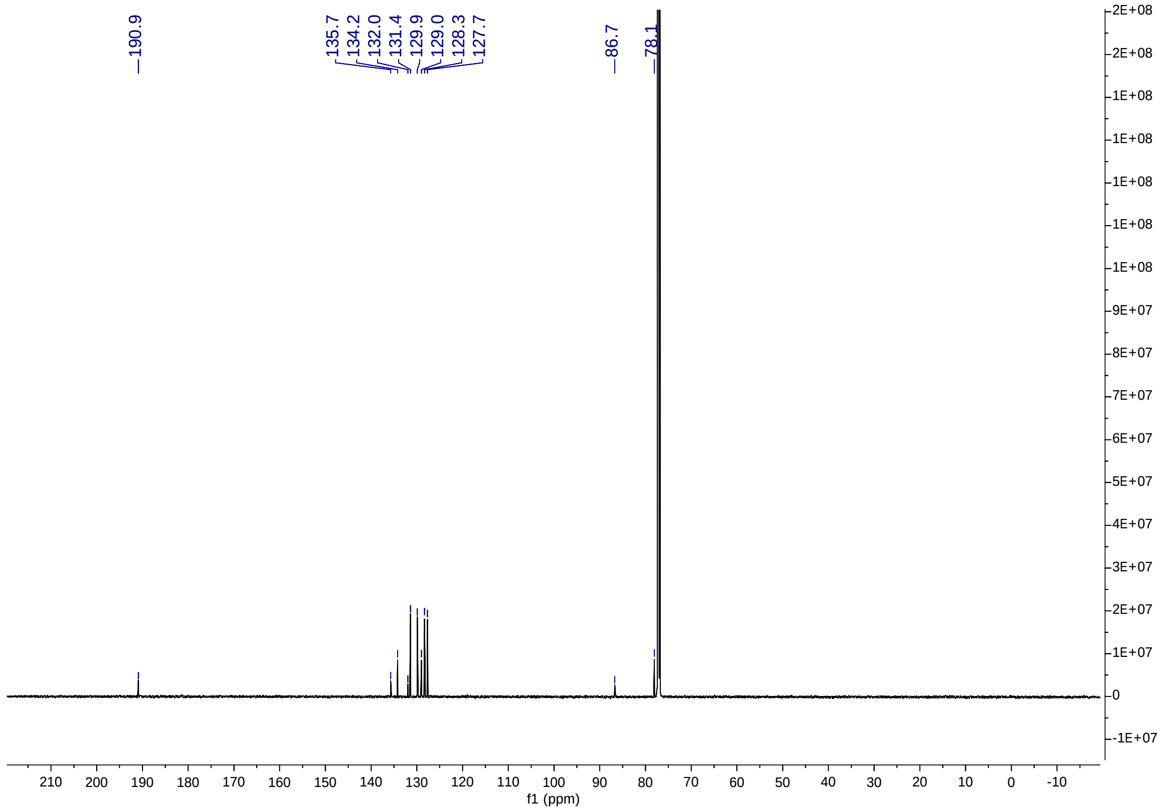

Ethyl 2-bromo-3-hydroxy-5-phenylpentanoate (8)

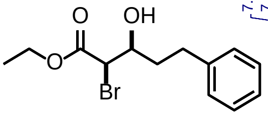

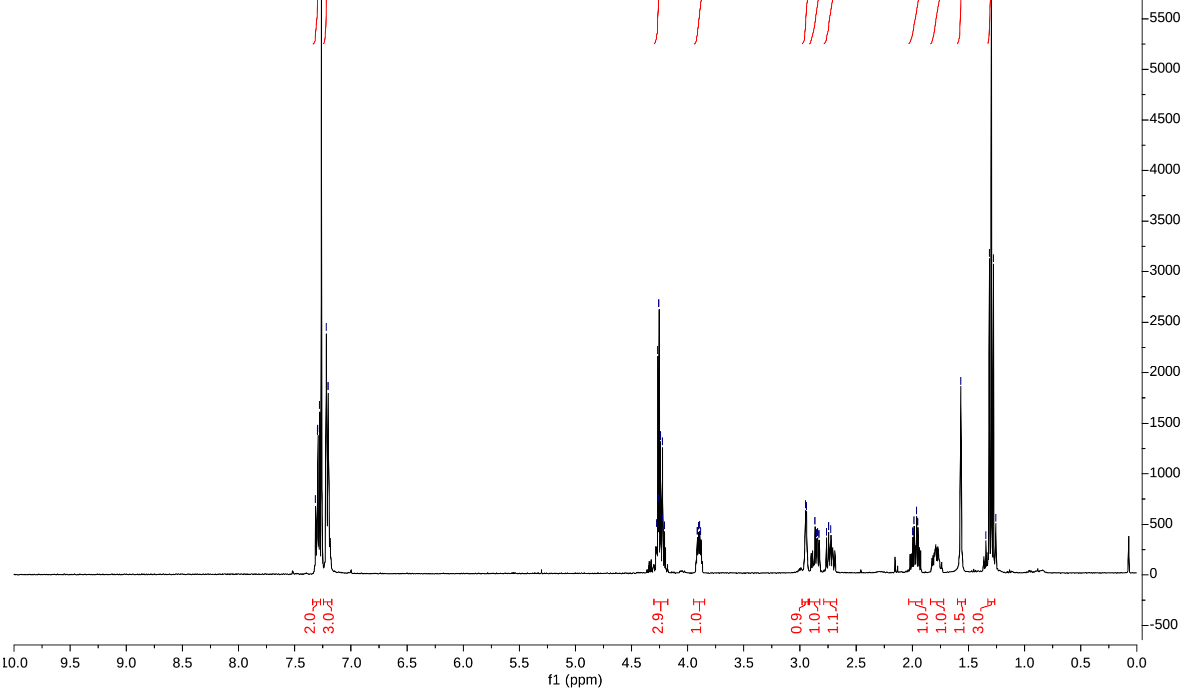

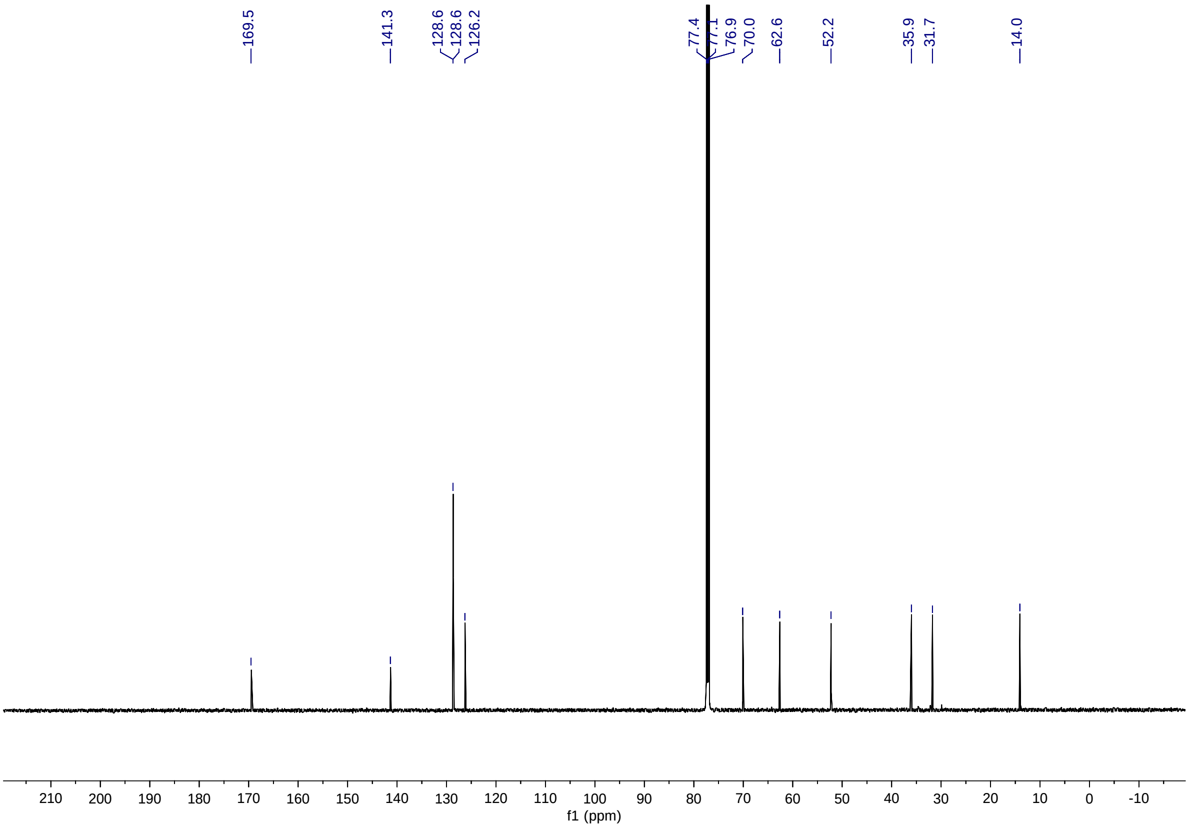

3,3-Dibromo-2-(*p*-tolyl)tetrahydrofuran-2-ol (12a)

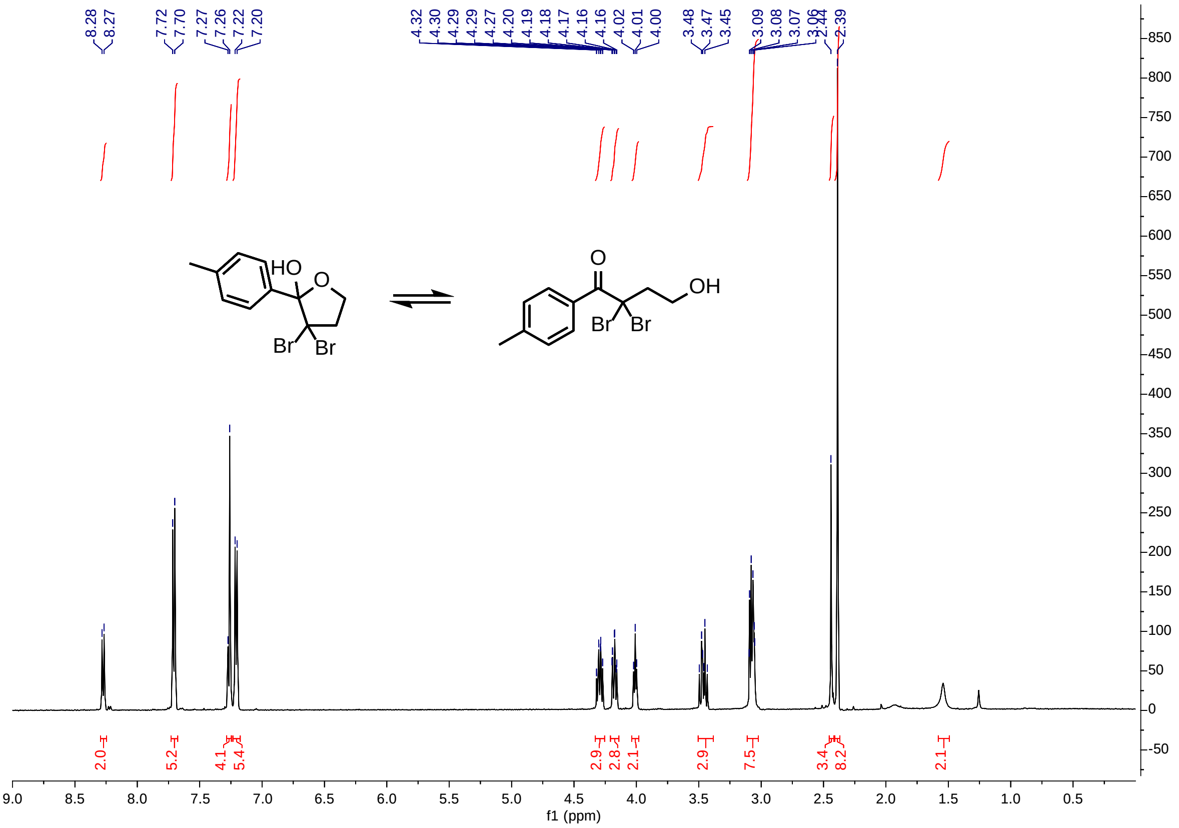

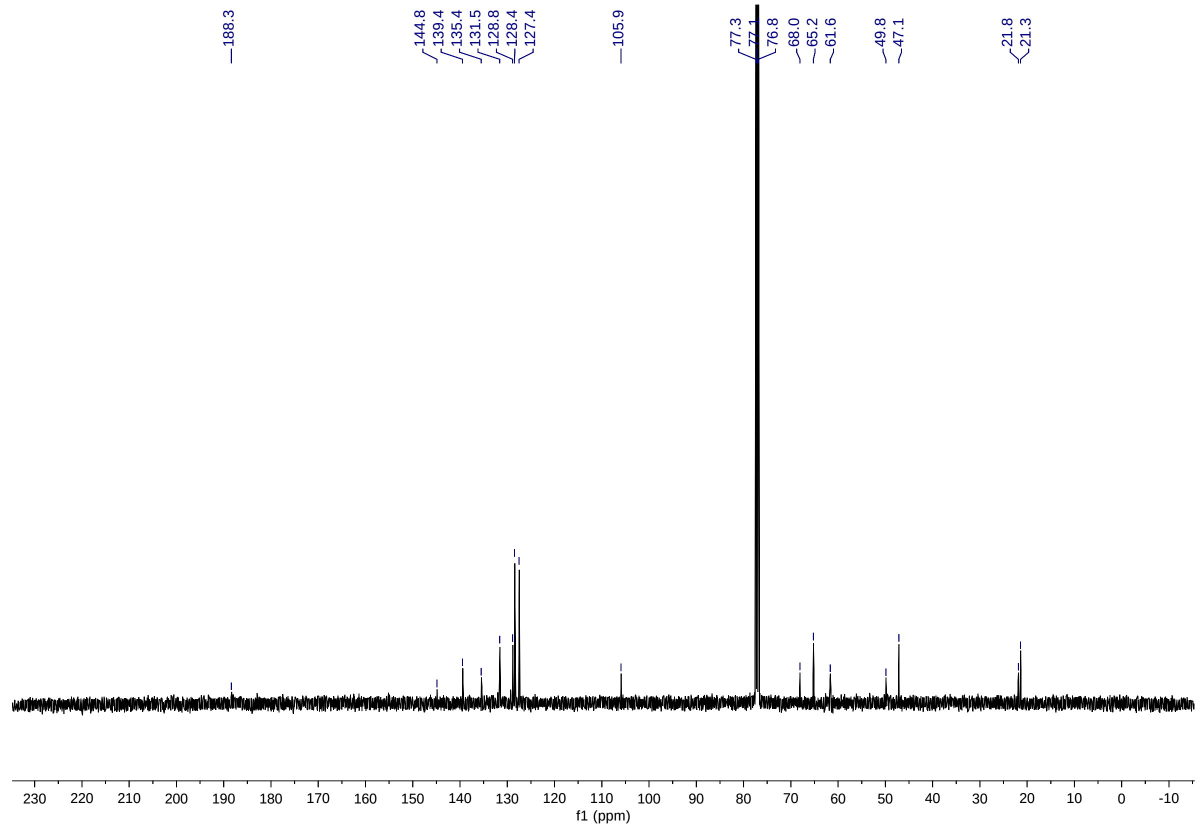

3,3-Dibromo-5,5-dimethyl-2-(p-tolyl)tetrahydrofuran-2-ol (12b)

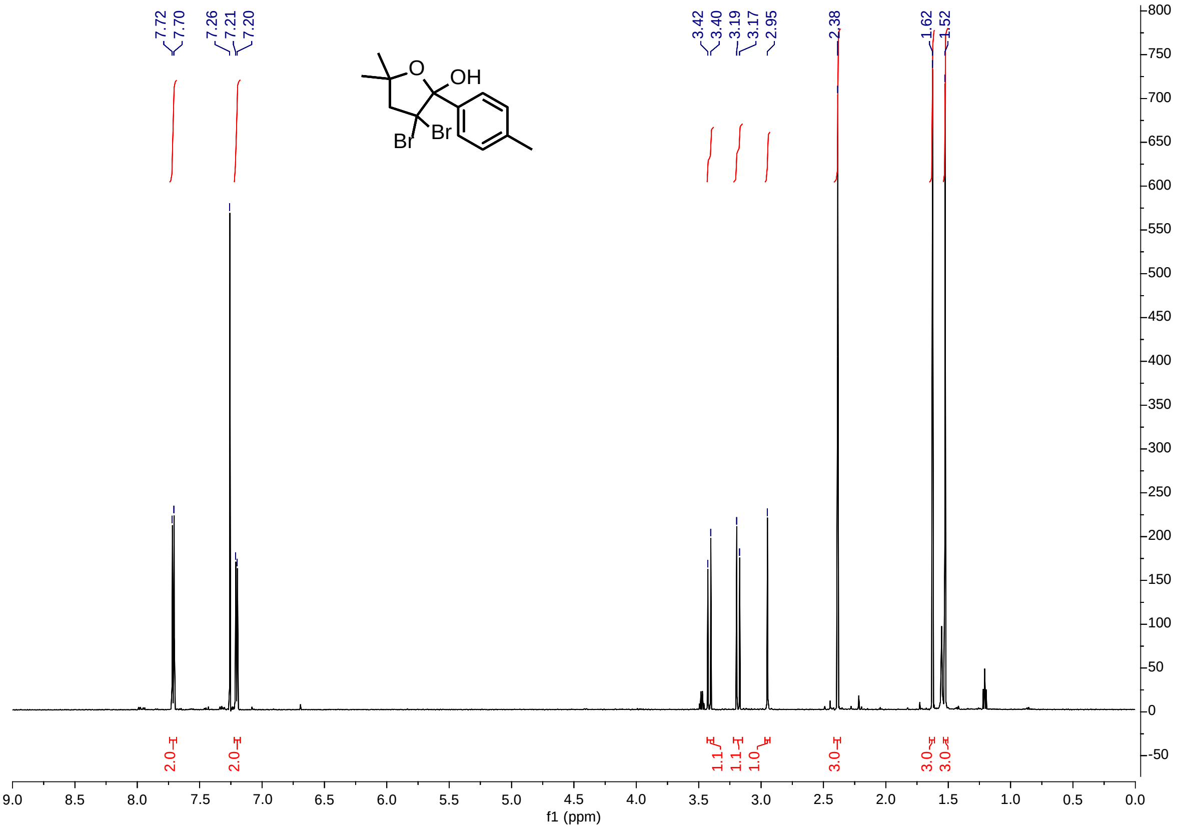

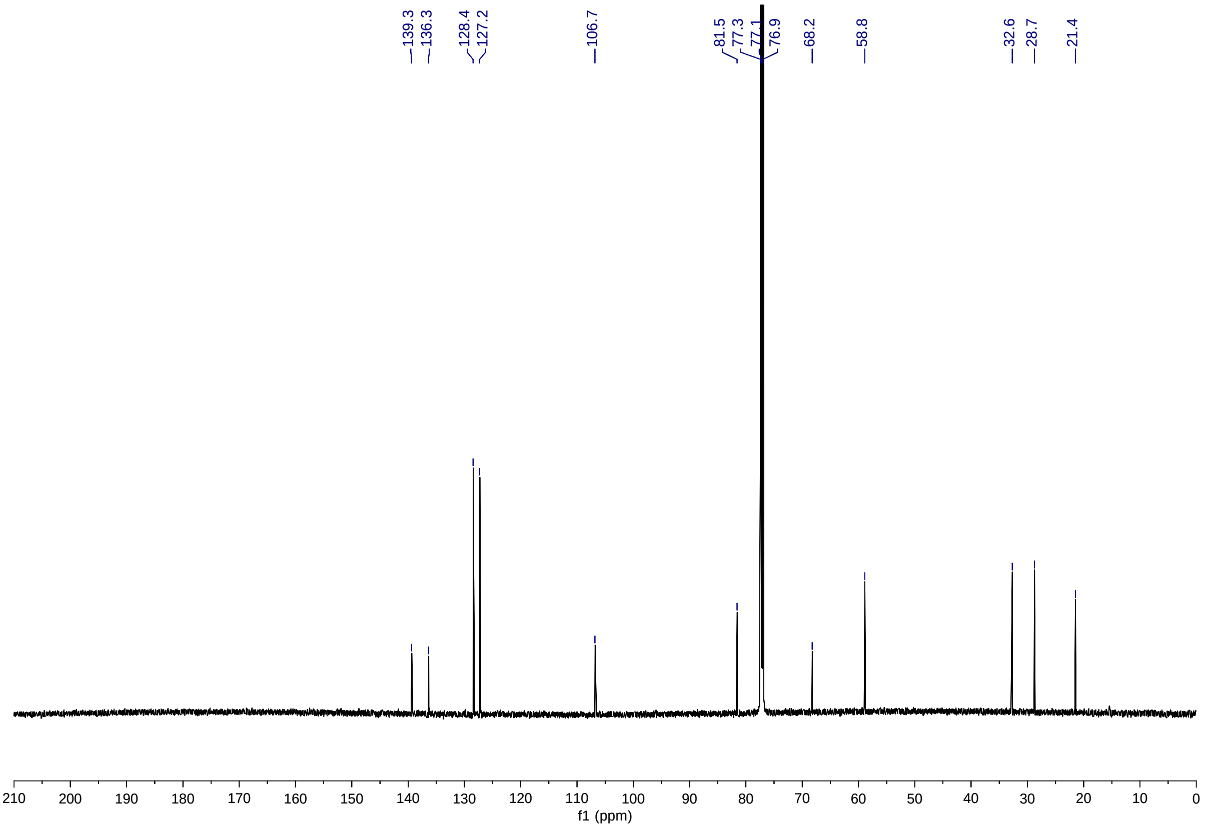

2-(Dibromomethyl)tetrahydrofuran-2-ol (12c)

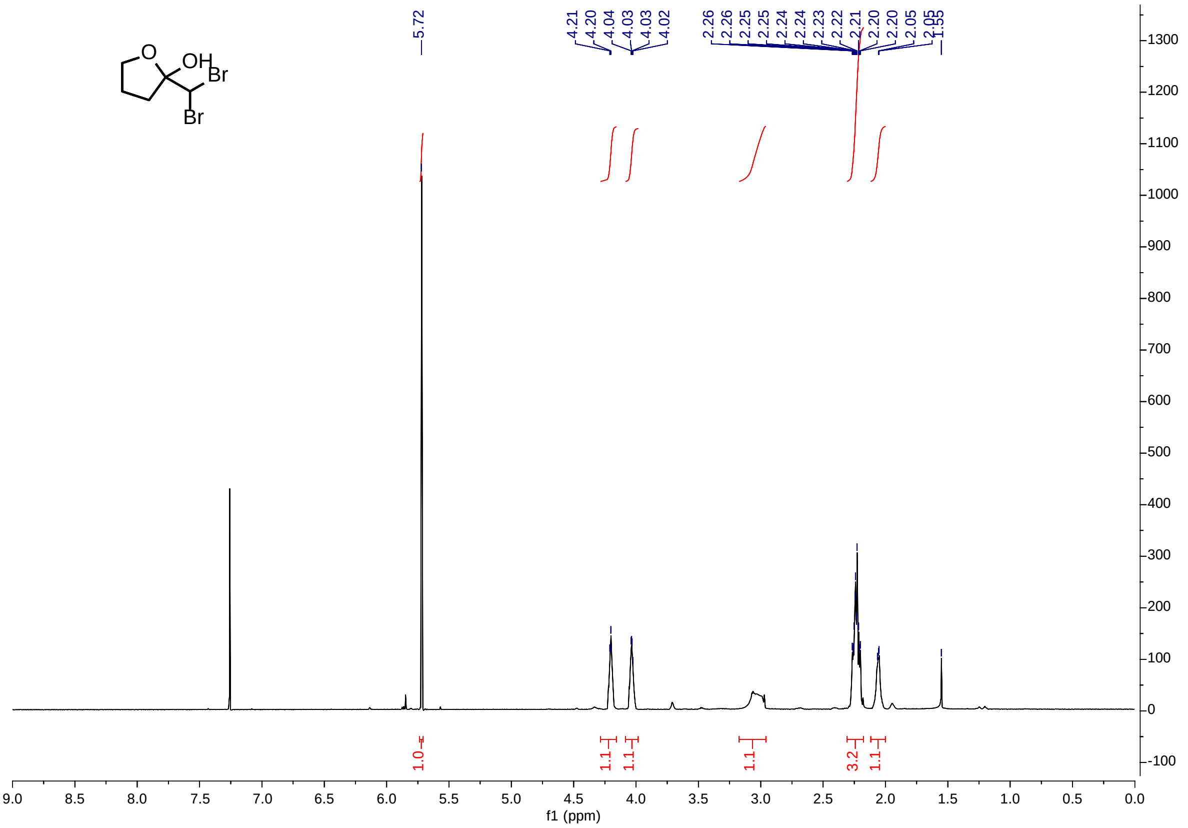

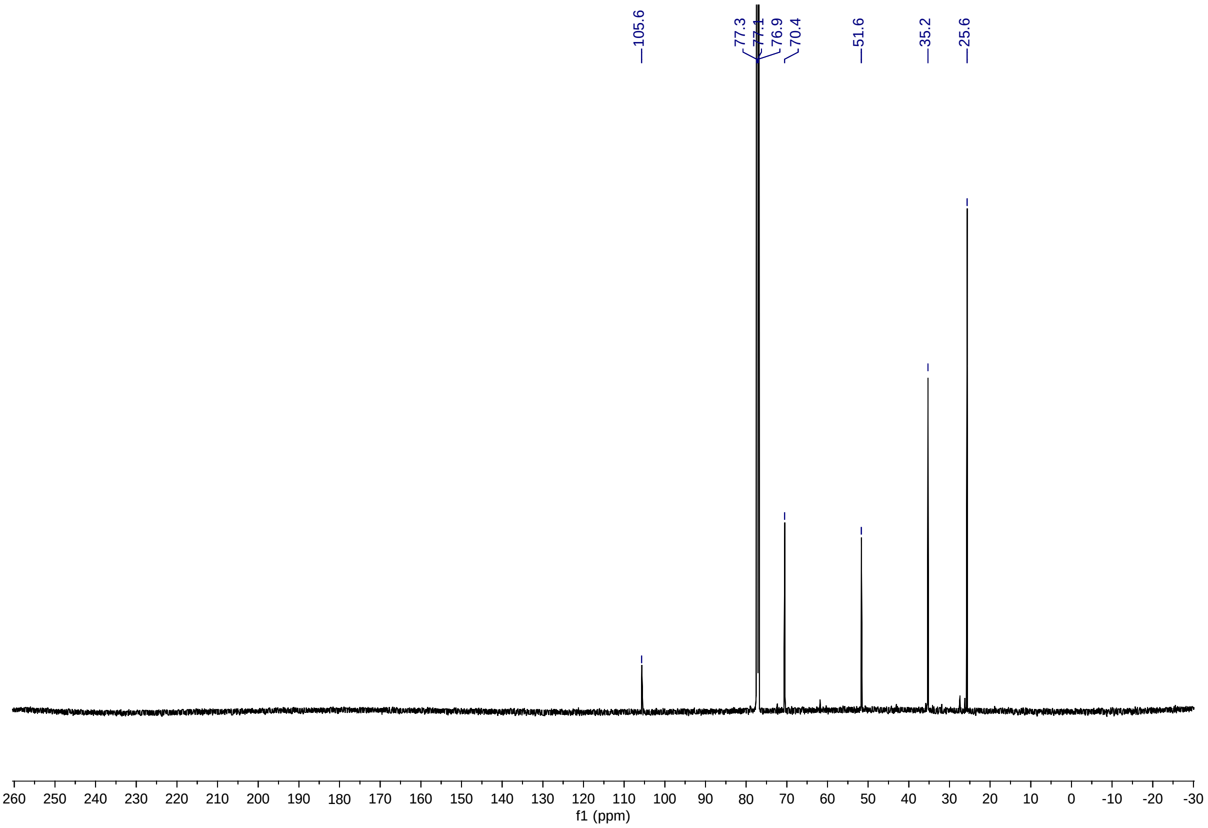

3,3-Dibromo-5-methyl-2-(*p*-tolyl)tetrahydrofuran-2-ol (12d)

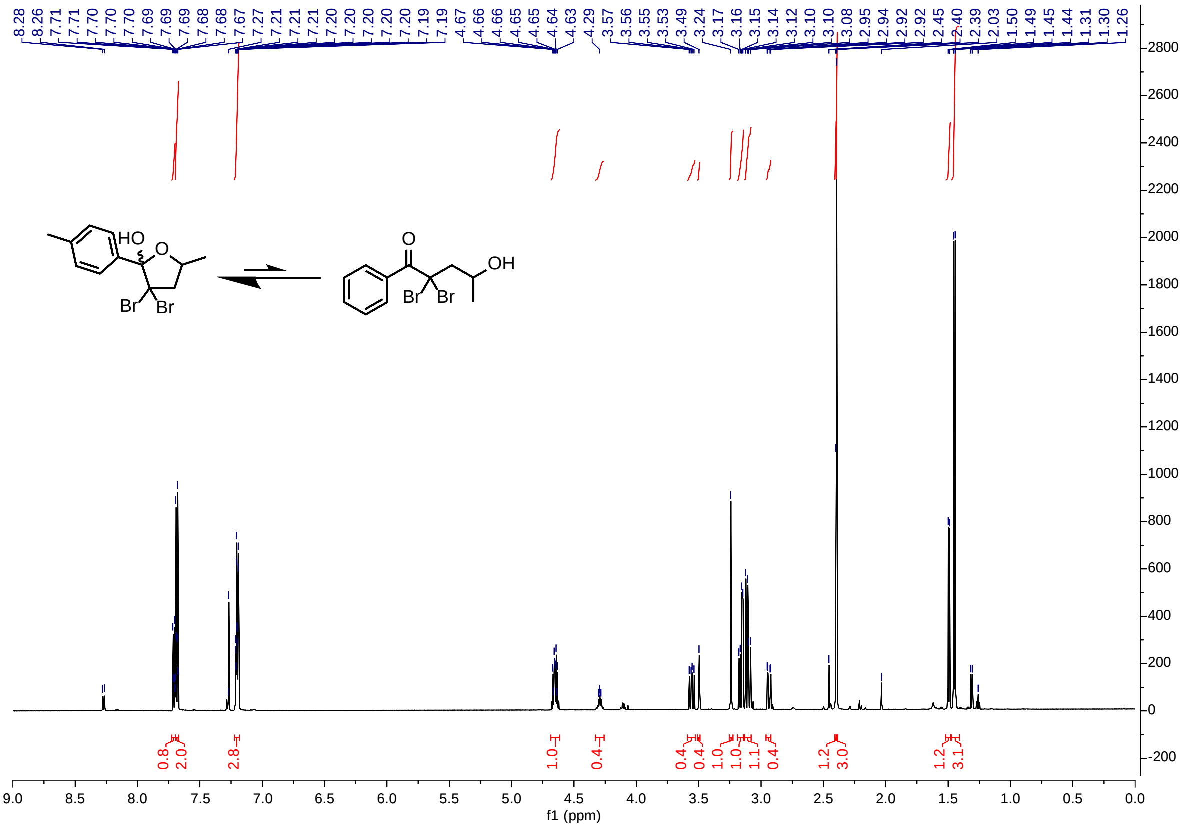

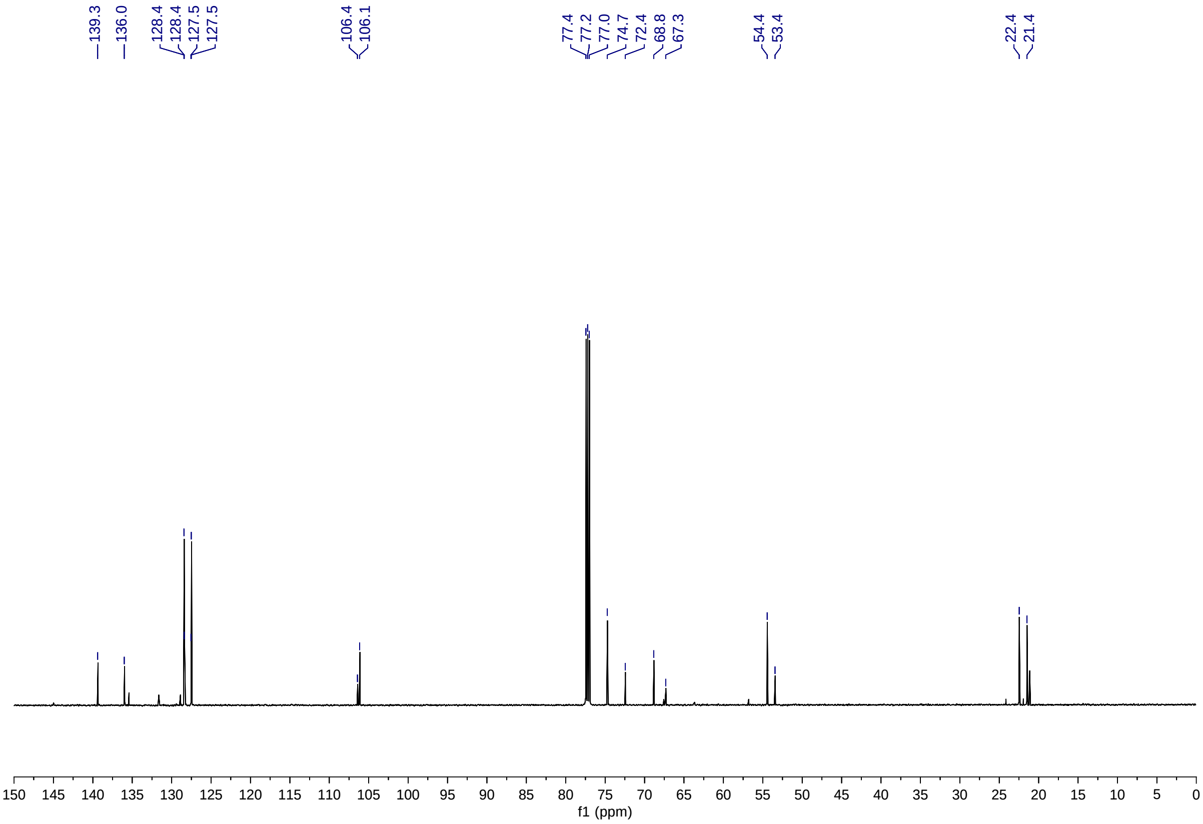

3,3-Dichloro-2-(*p*-tolyl)tetrahydrofuran-2-ol (13a)

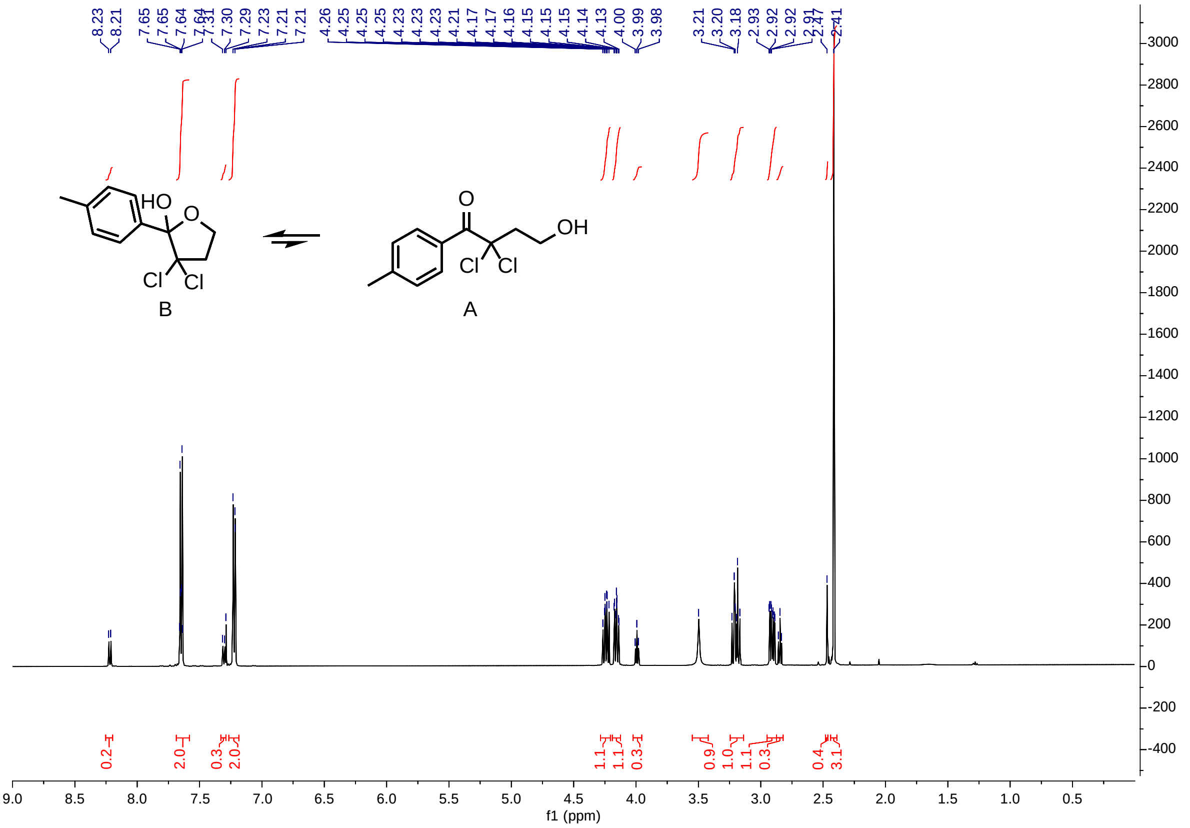

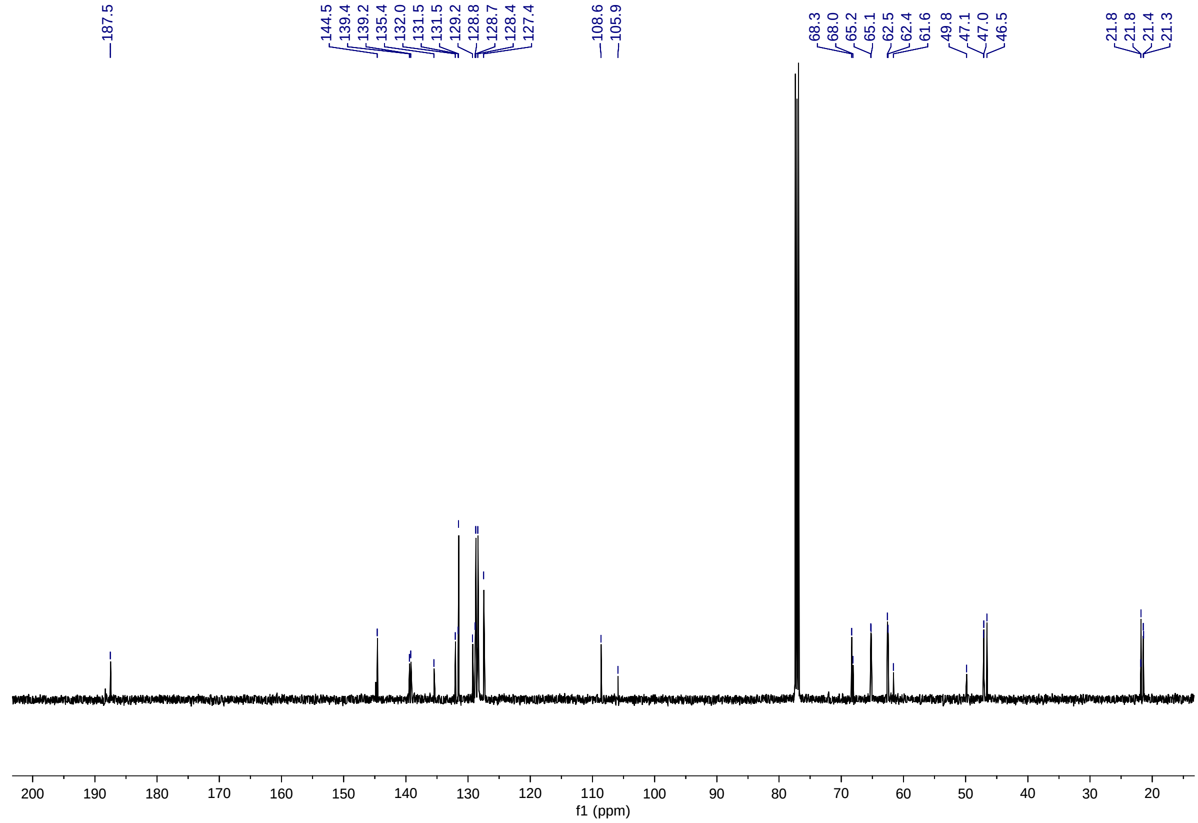

3,3-Dichloro-5-methyl-2-(*p*-tolyl)tetrahydrofuran-2-ol (13d)

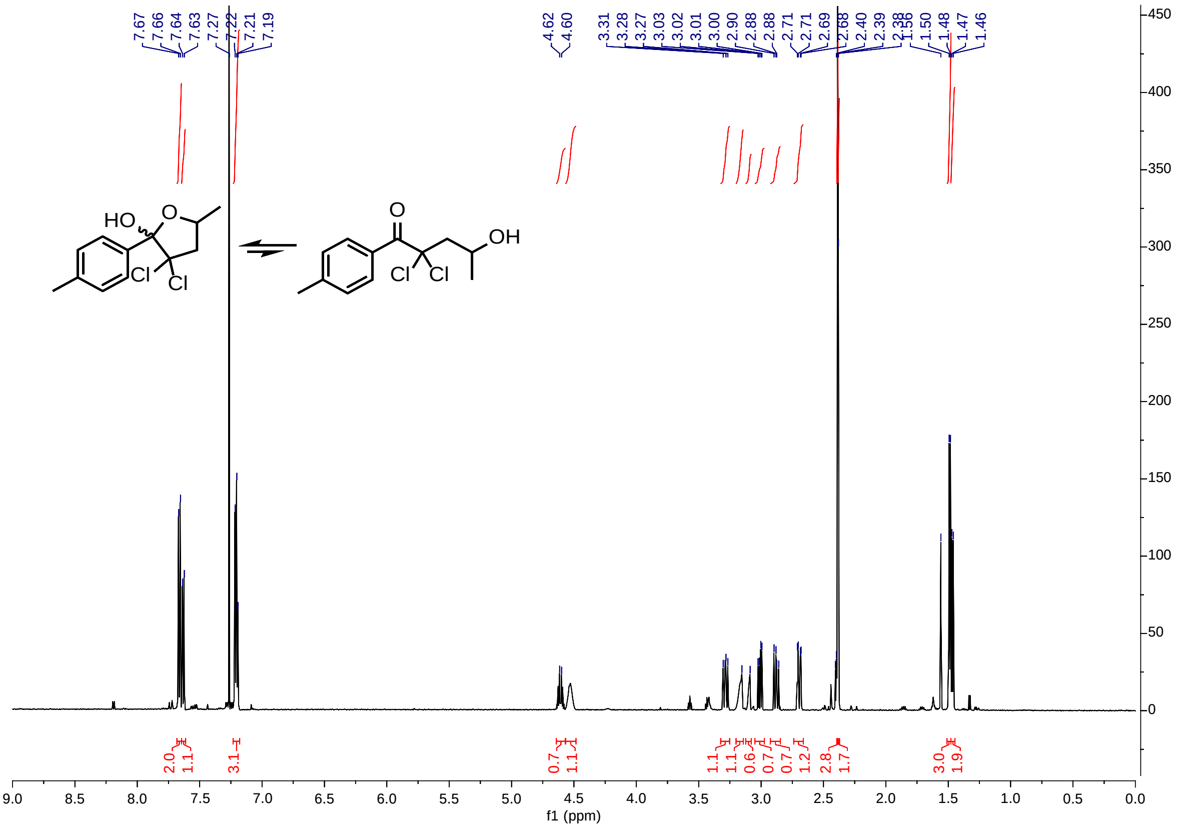

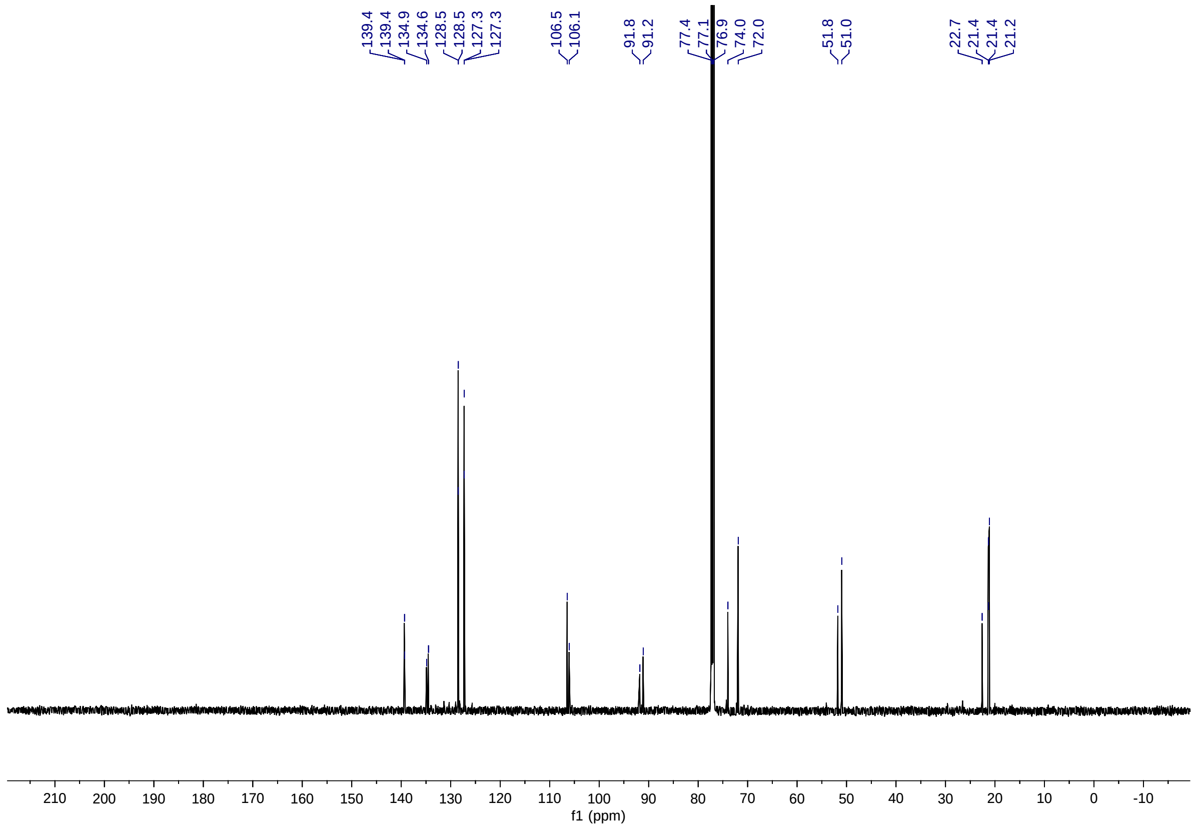

2,2-Dibromo-1-(*p*-tolyl)butane-1,4-diol (14)

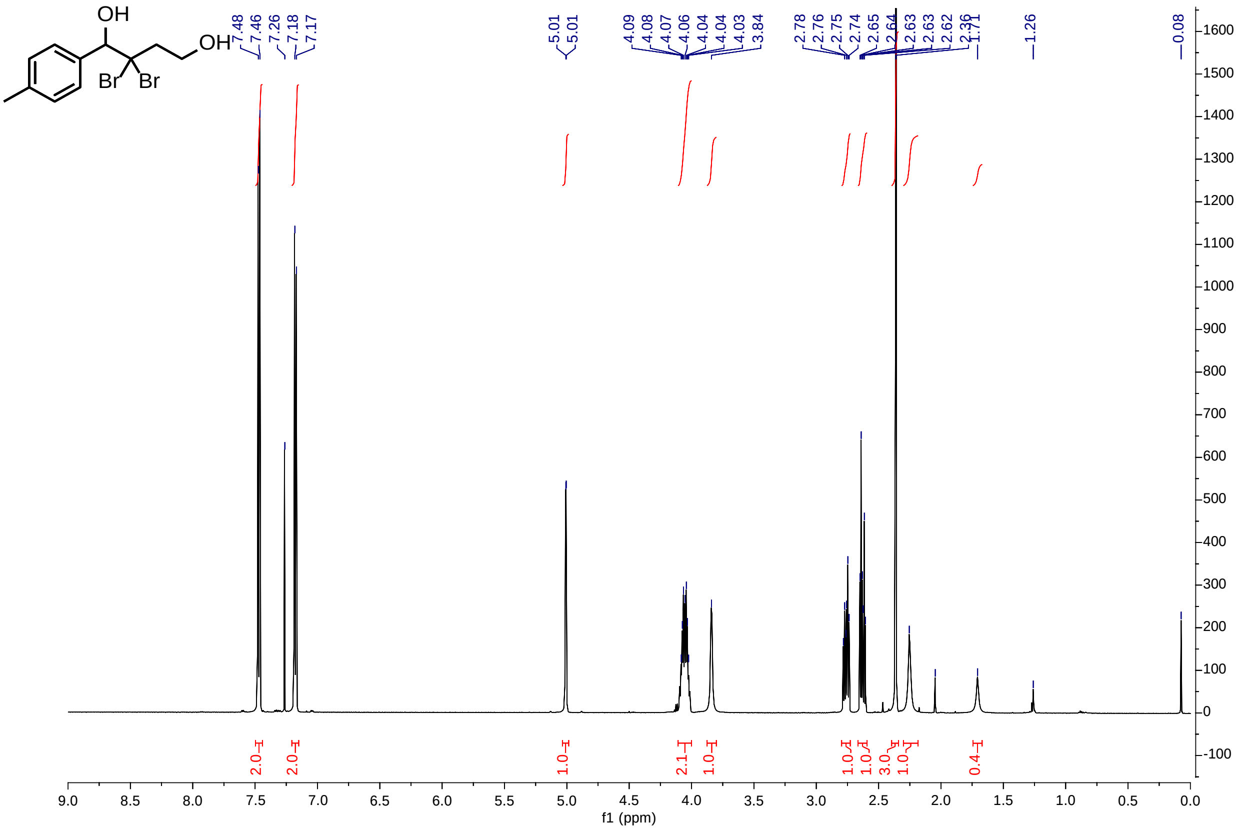

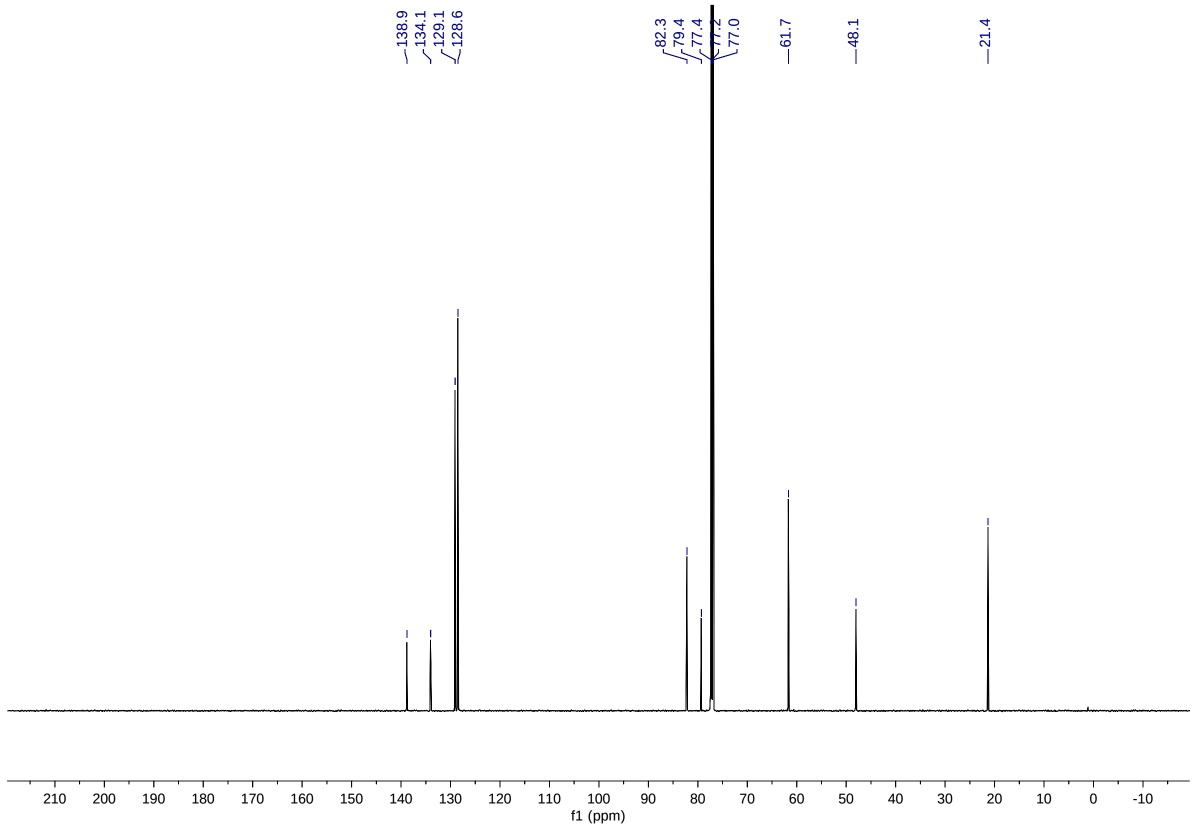

2,2-Dichloro-1-(*p*-tolyl)butane-1,4-diol (15)

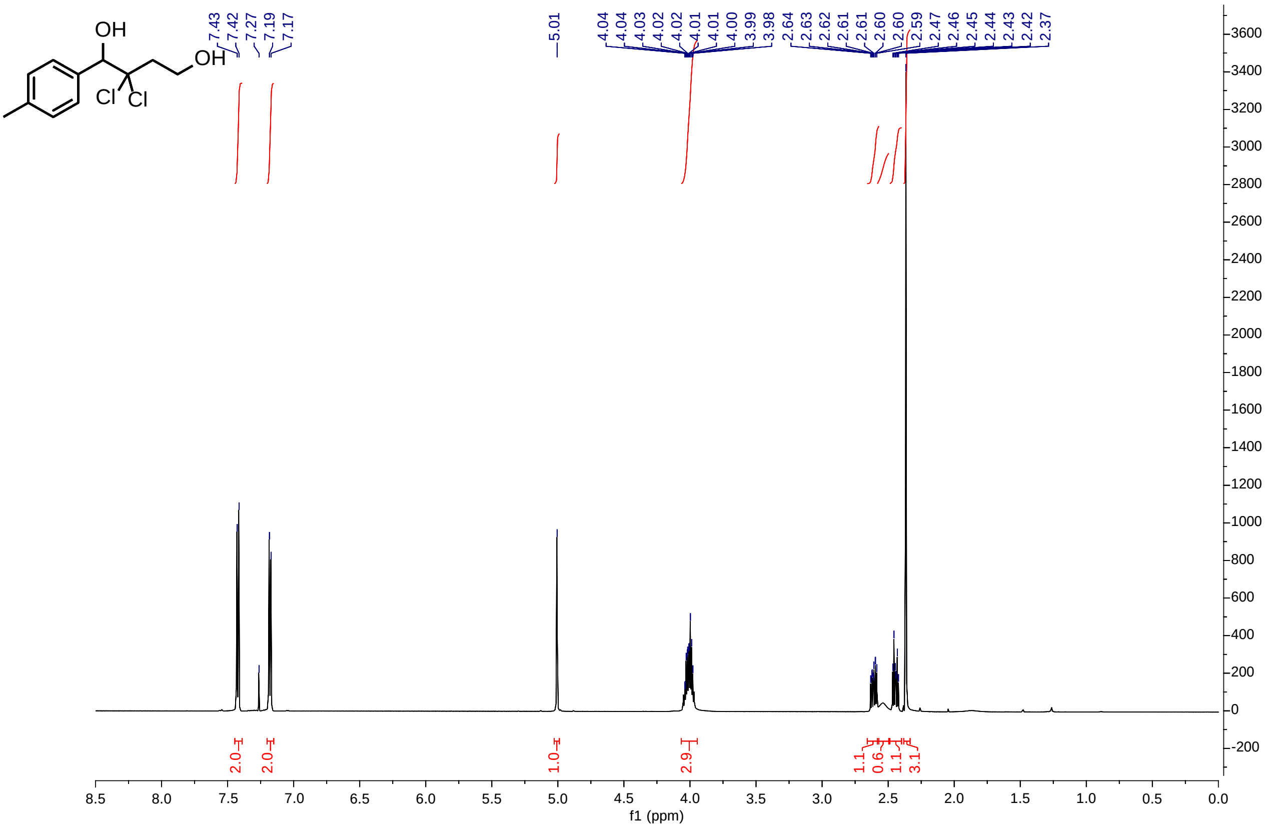

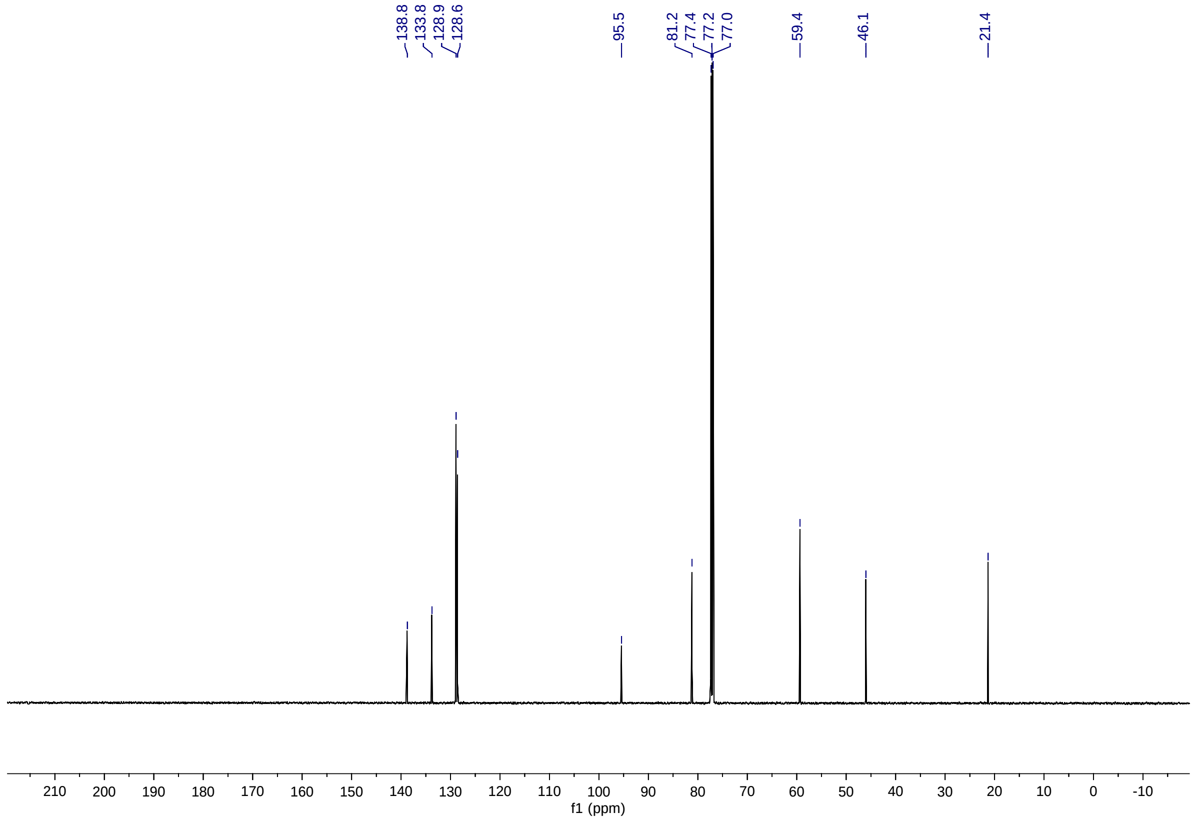

3,3-Dibromo-2-(*p*-tolyl)tetrahydrofuran (16)

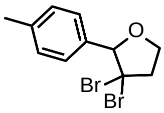

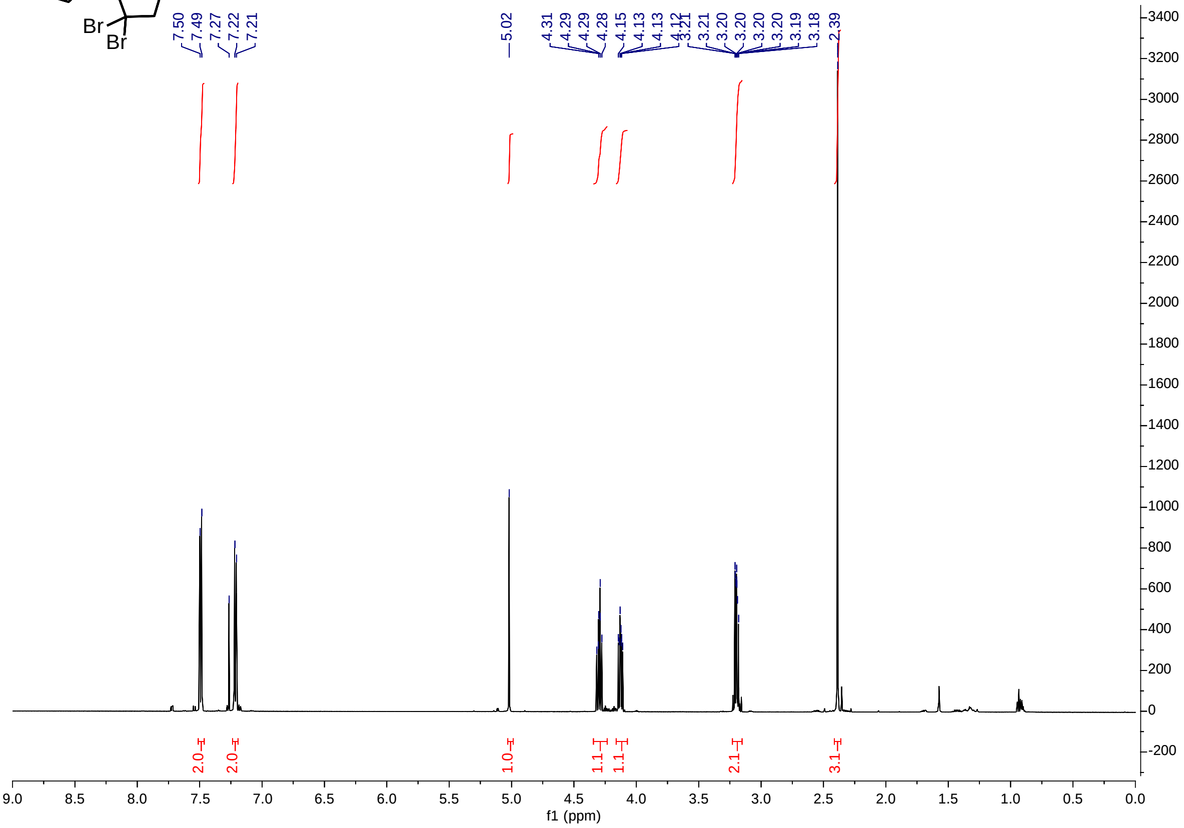

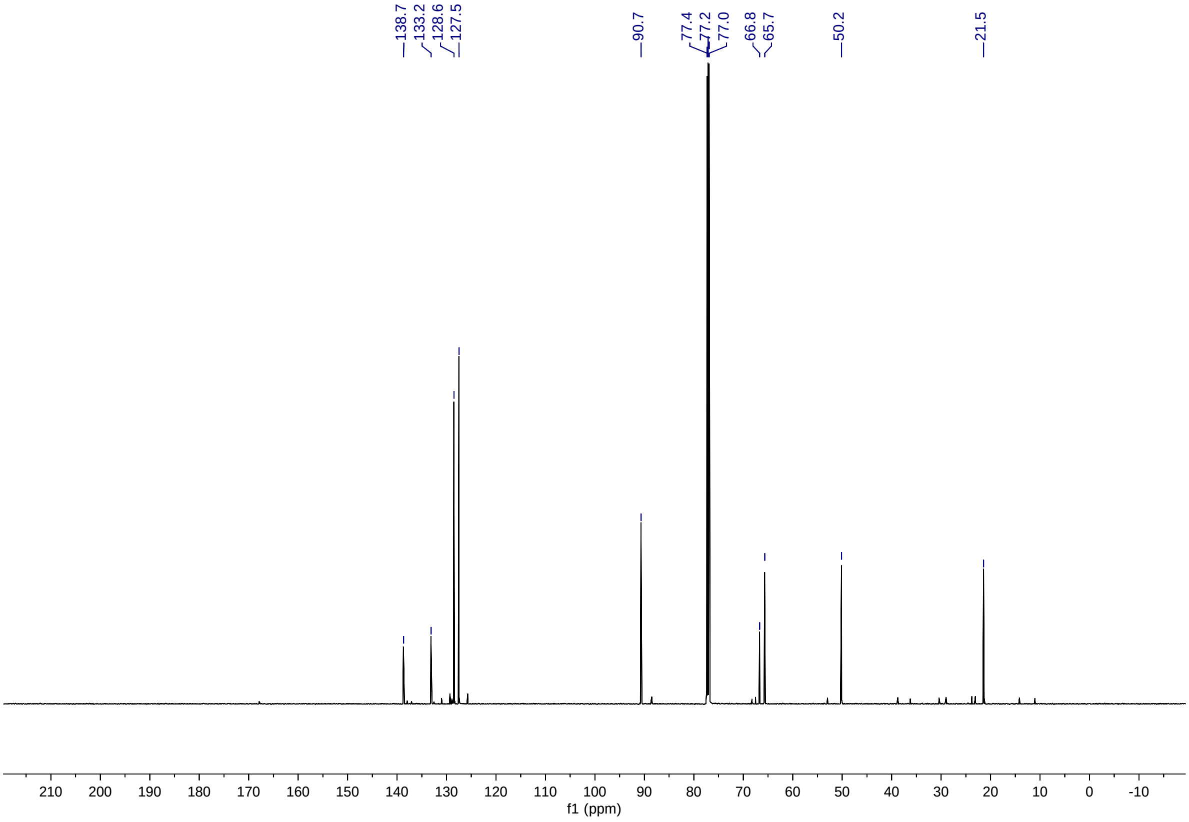

3,3-Dichloro-2-(*p*-tolyl)tetrahydrofuran (17)

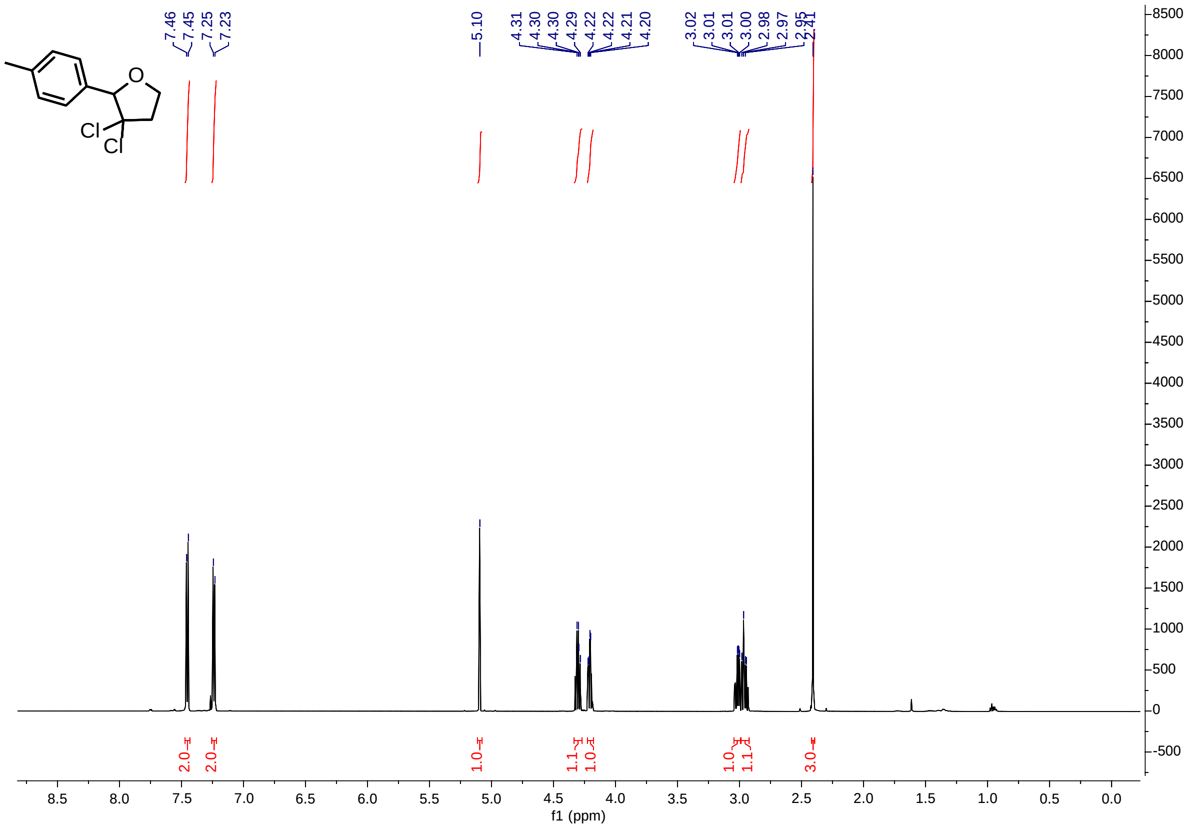

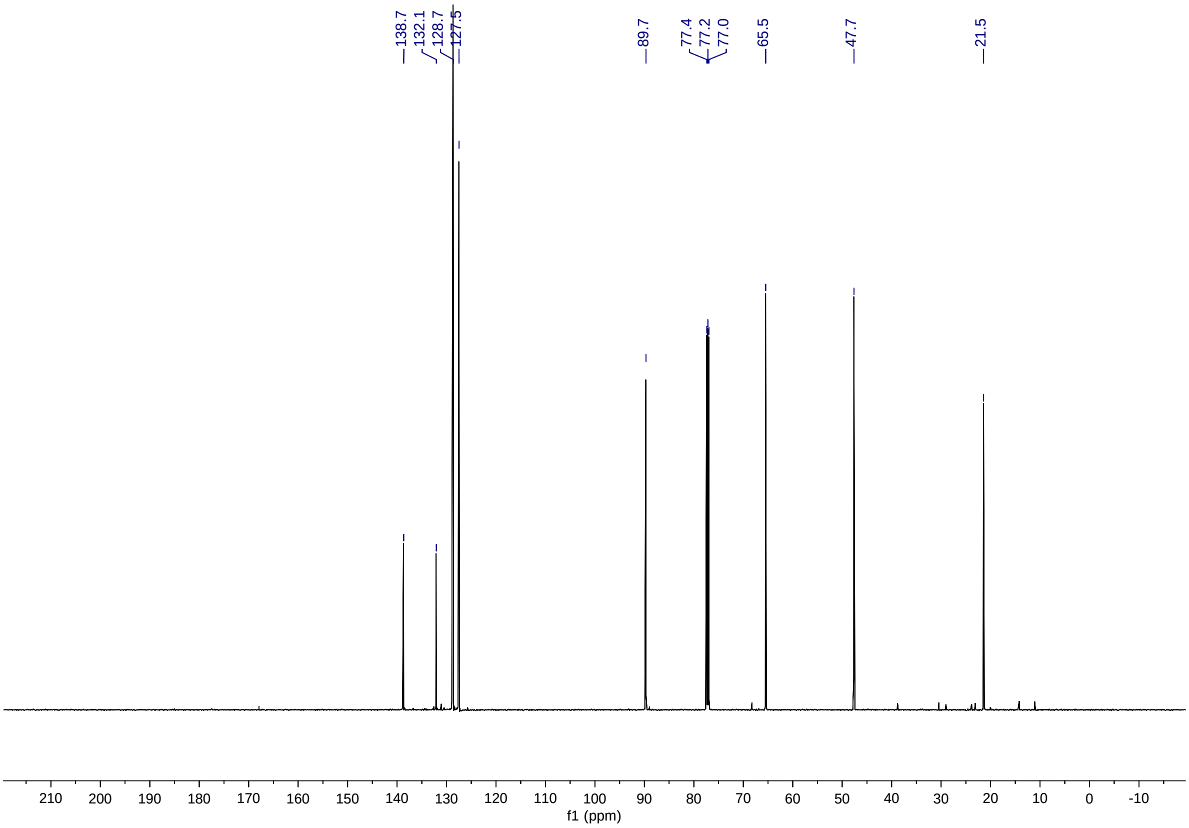

***anti*-2,2-Dichloro-4-methyl-1-phenylpentane-1,3-diol (18a)**

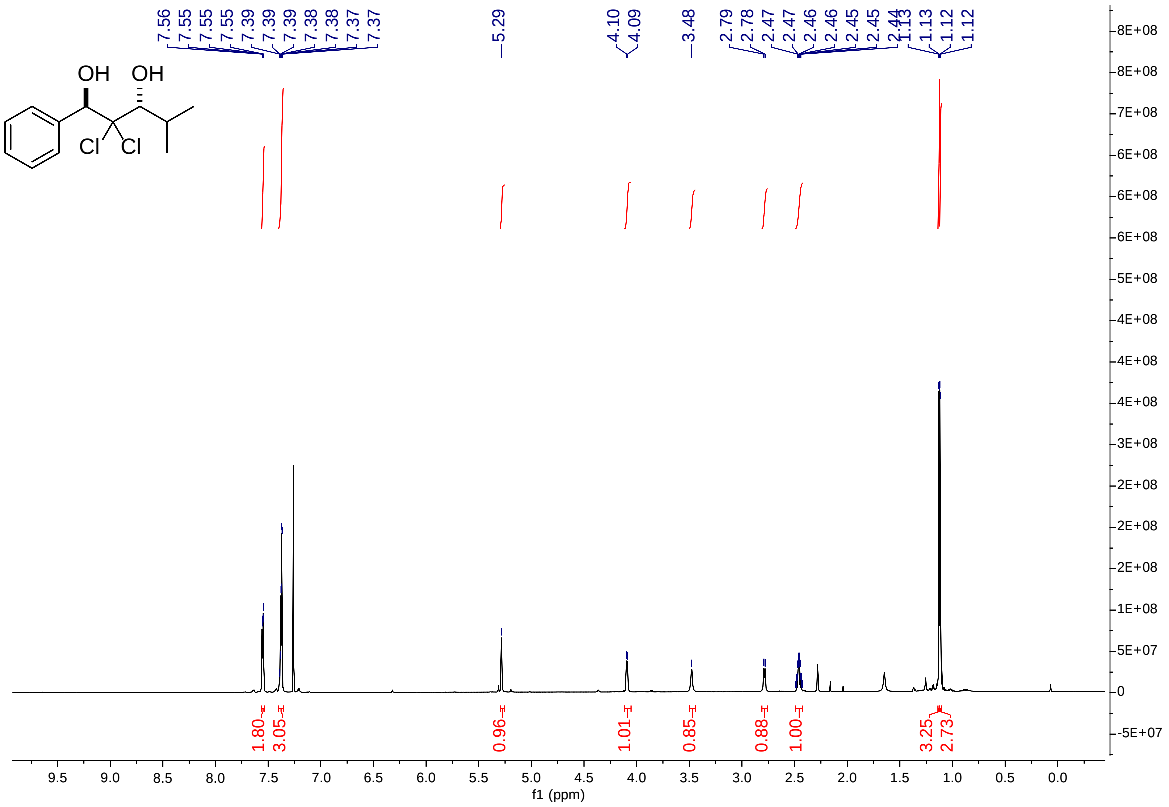

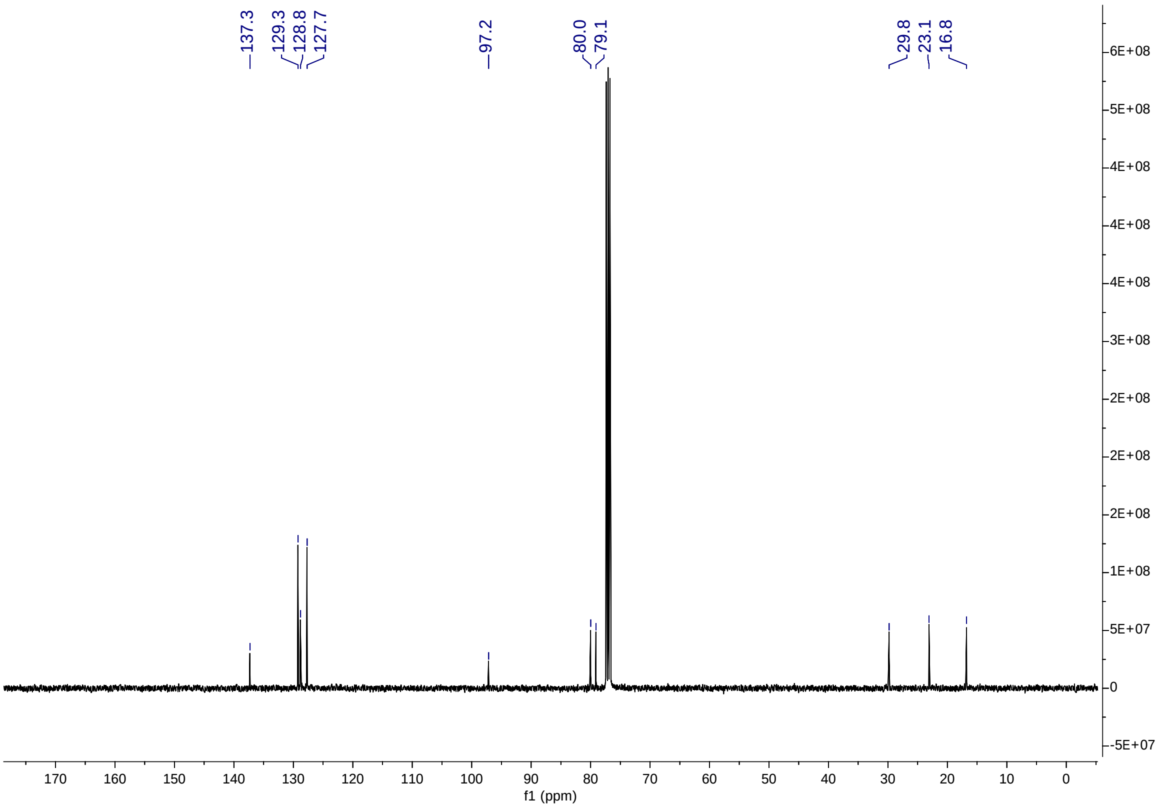

anti-2,2-Dichloro-1,3-diphenylpropane-1,3-diol (18b)

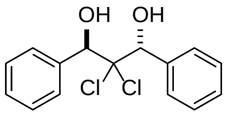

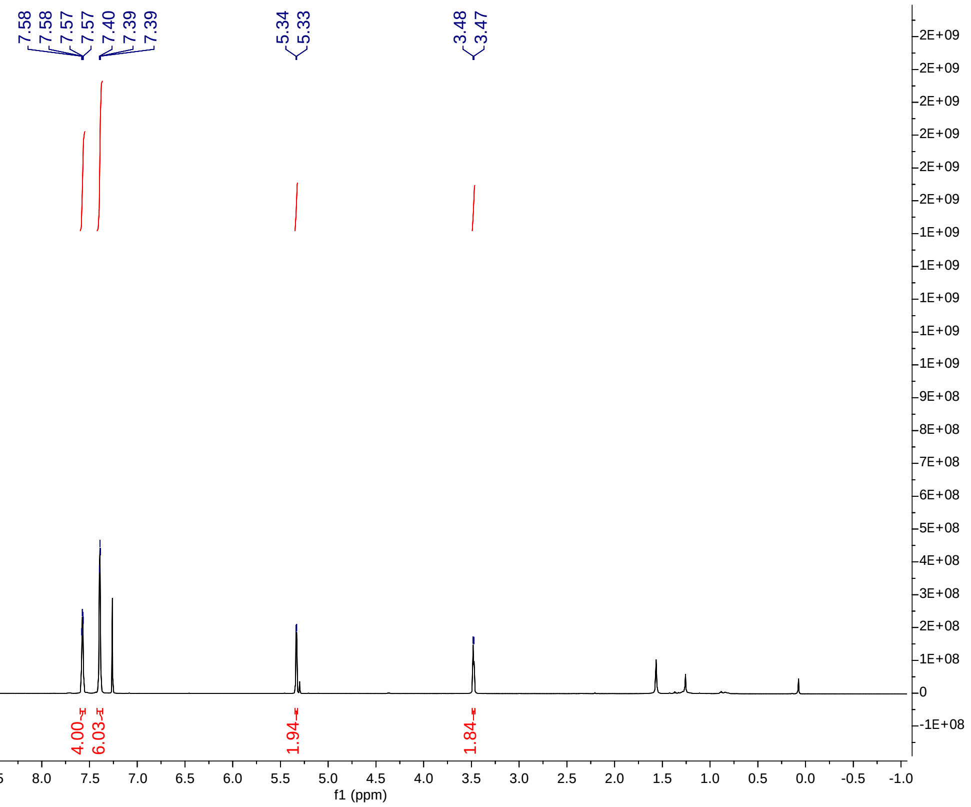

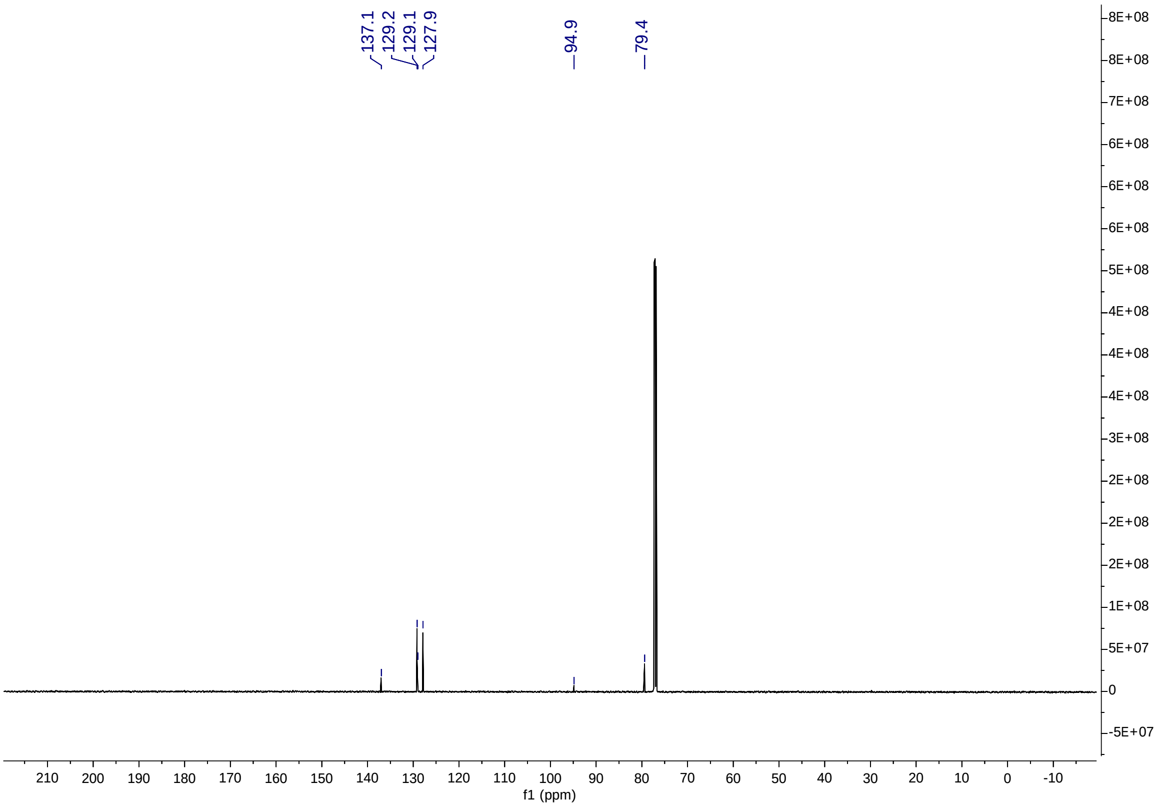

anti-2,2-Dibromo-4-methyl-1-phenylpentane-1,3-diol (19)

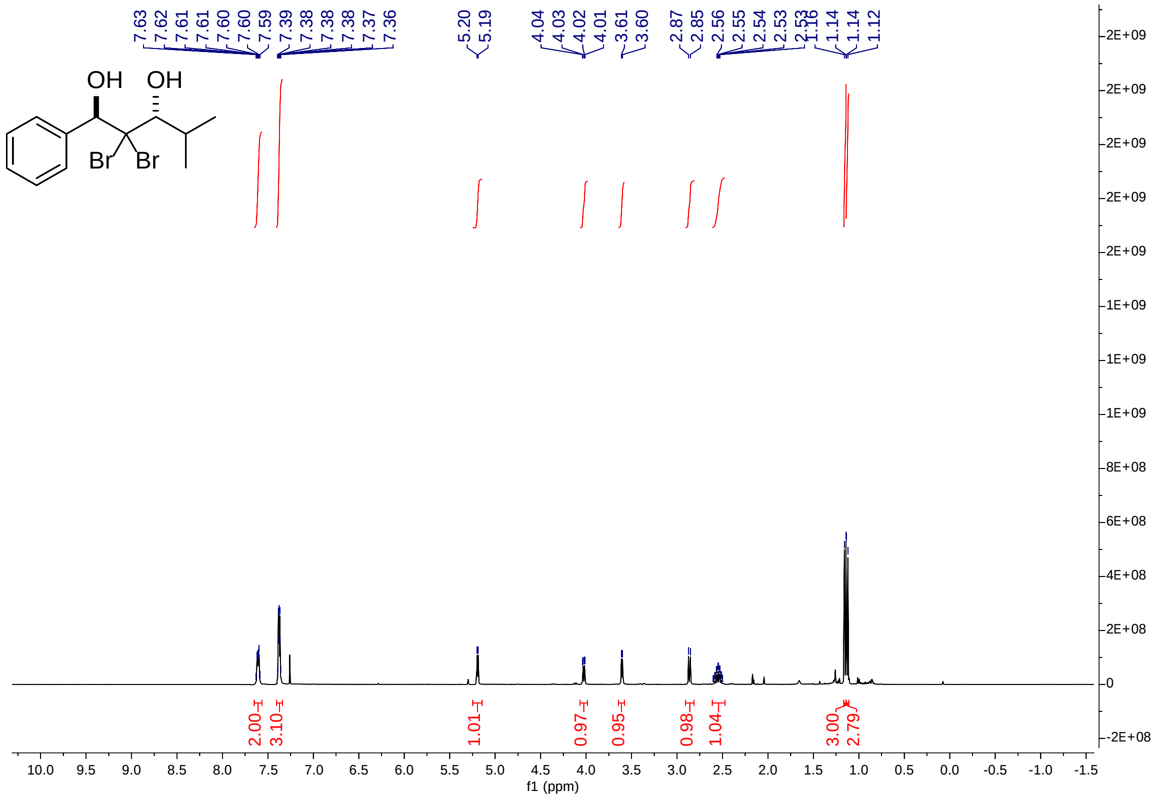

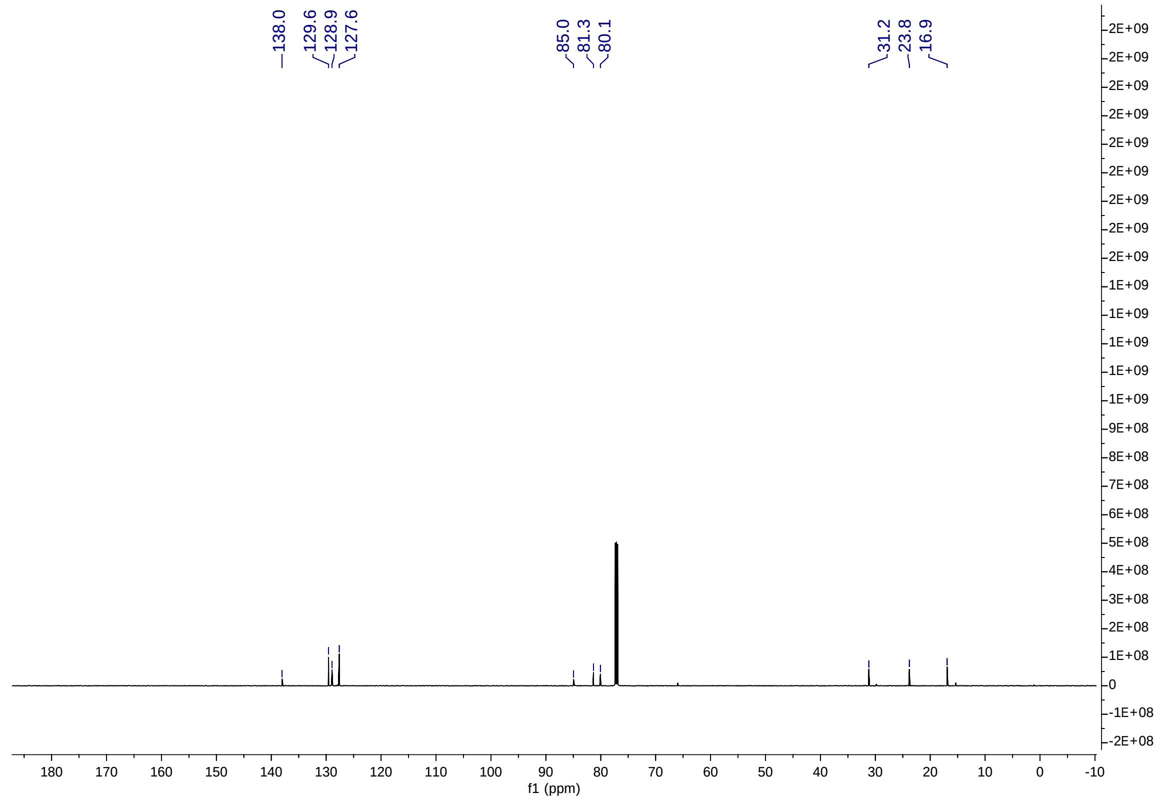

(1*S*,3*R*)-2,2-Dibromo-1-(*p*-tolyl)hexane-1,3-diol (20)

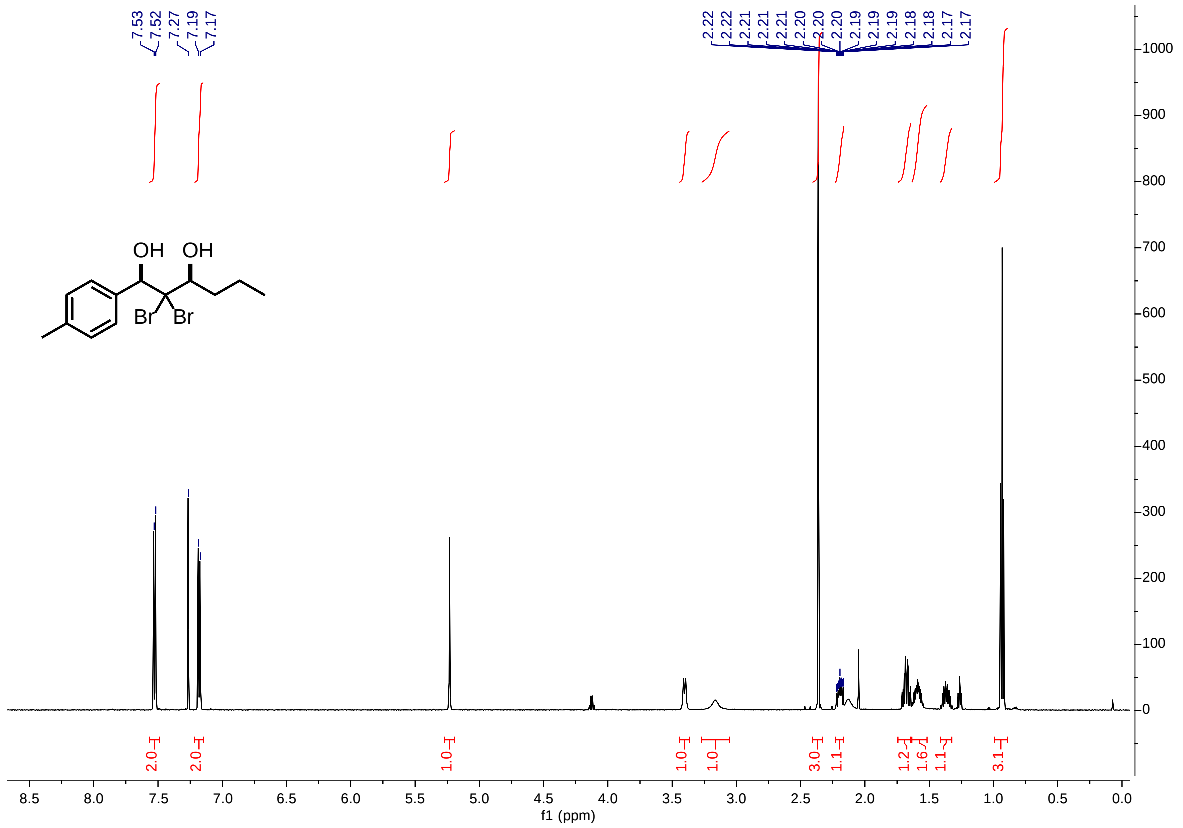

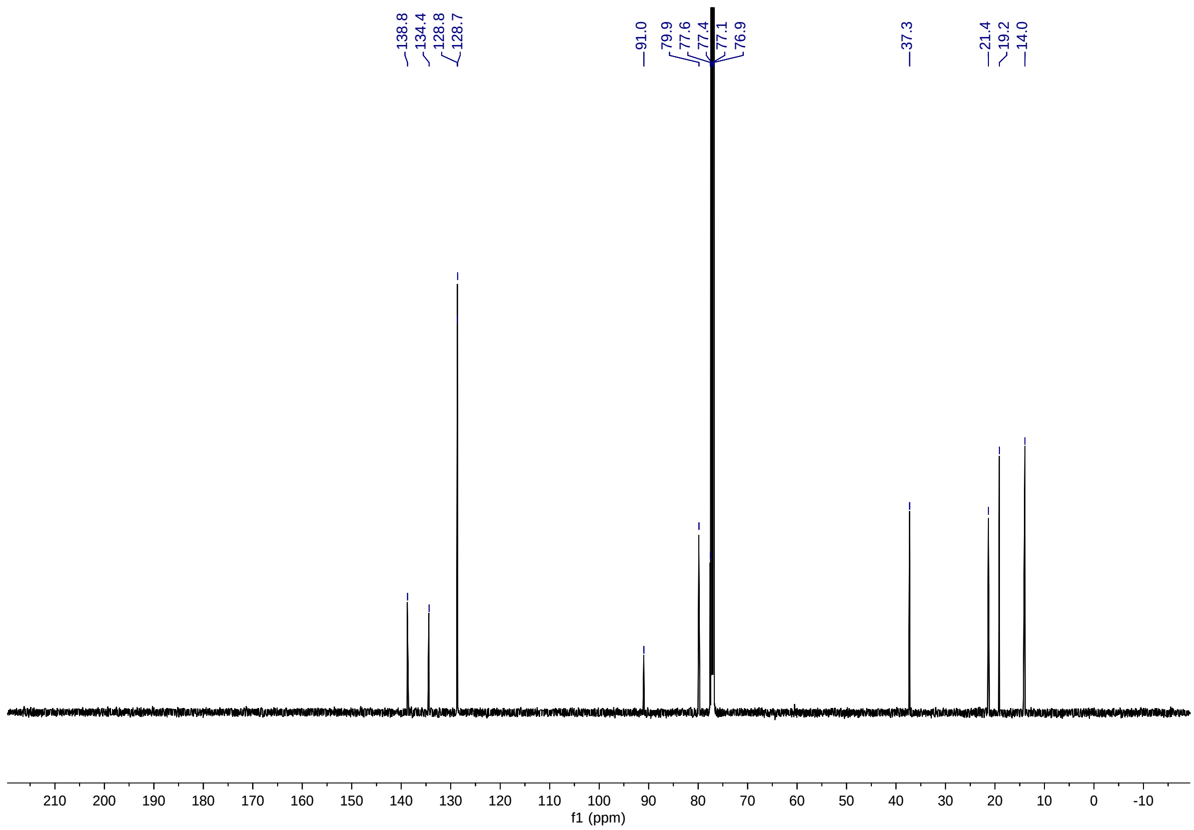

2-Bromo-1-(p-tolyl)hexane-1,3-diol (21)

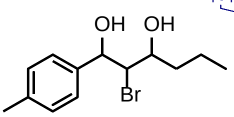

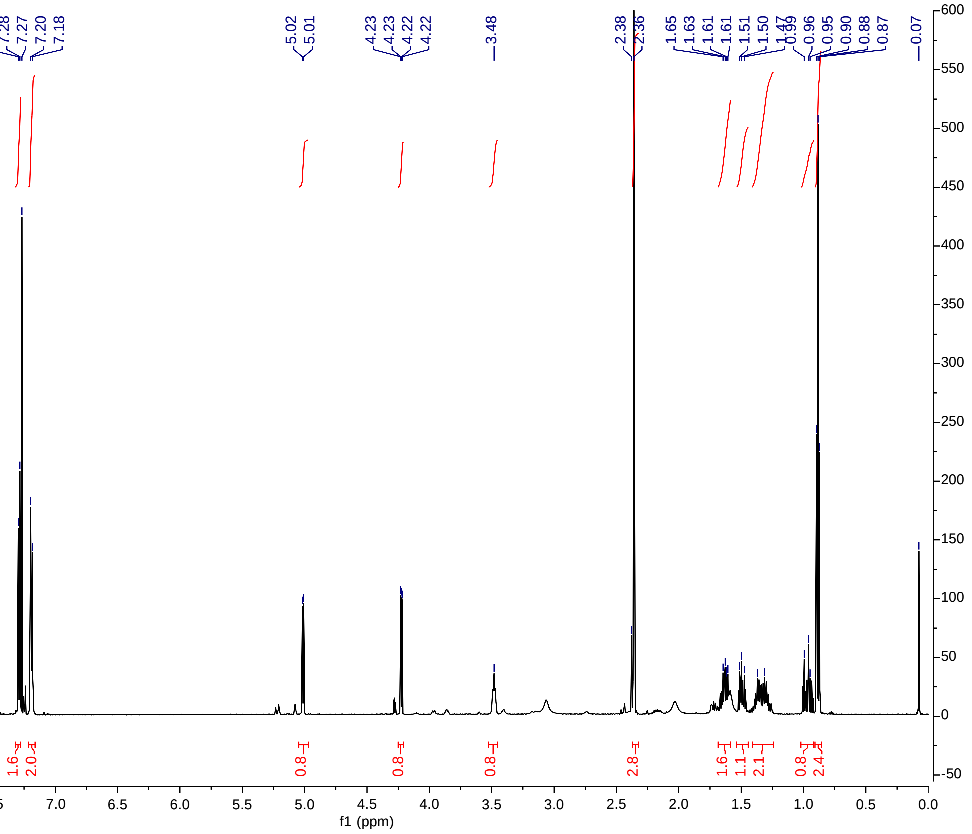

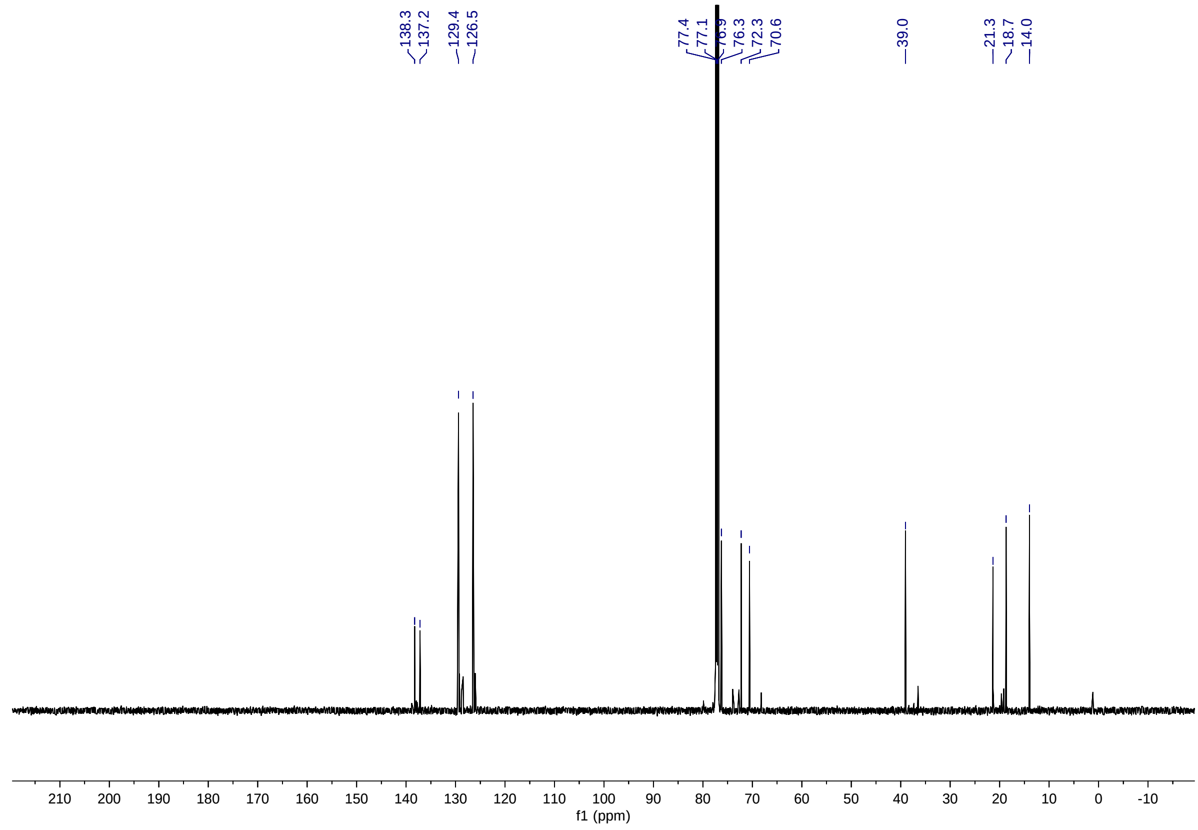

(4R,6S)-5,5-Dibromo-2,2-dimethyl-4-propyl-6-(p-tolyl)-1,3-dioxane (23)

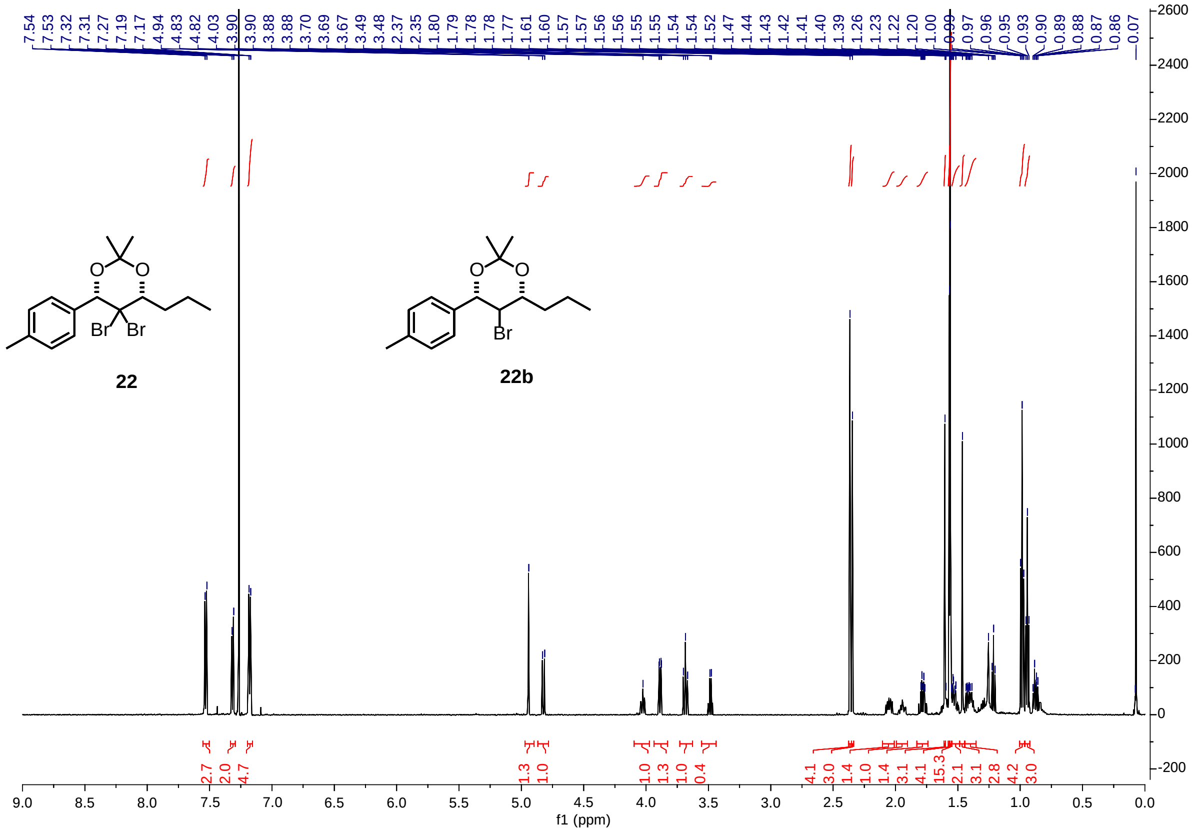

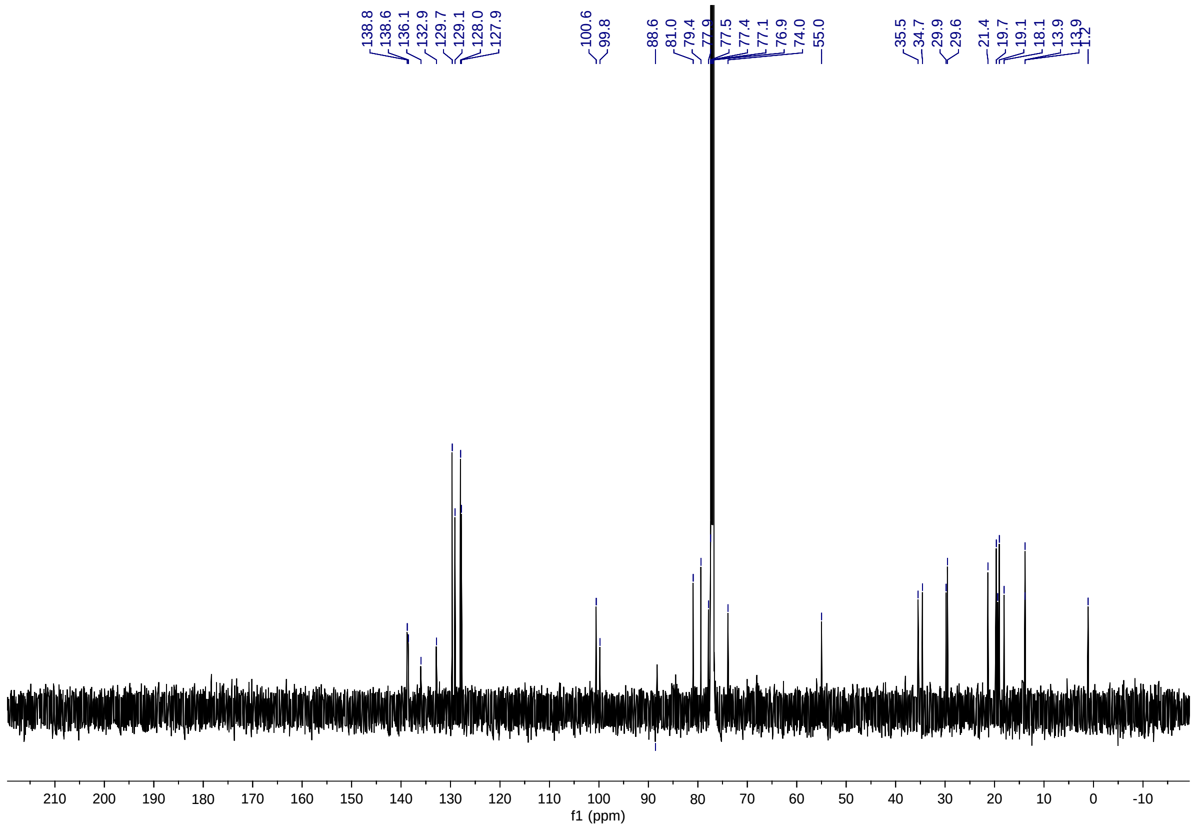

syn-2,2-Dichloro-4-methyl-1-phenylpentane-1,3-diol (24)

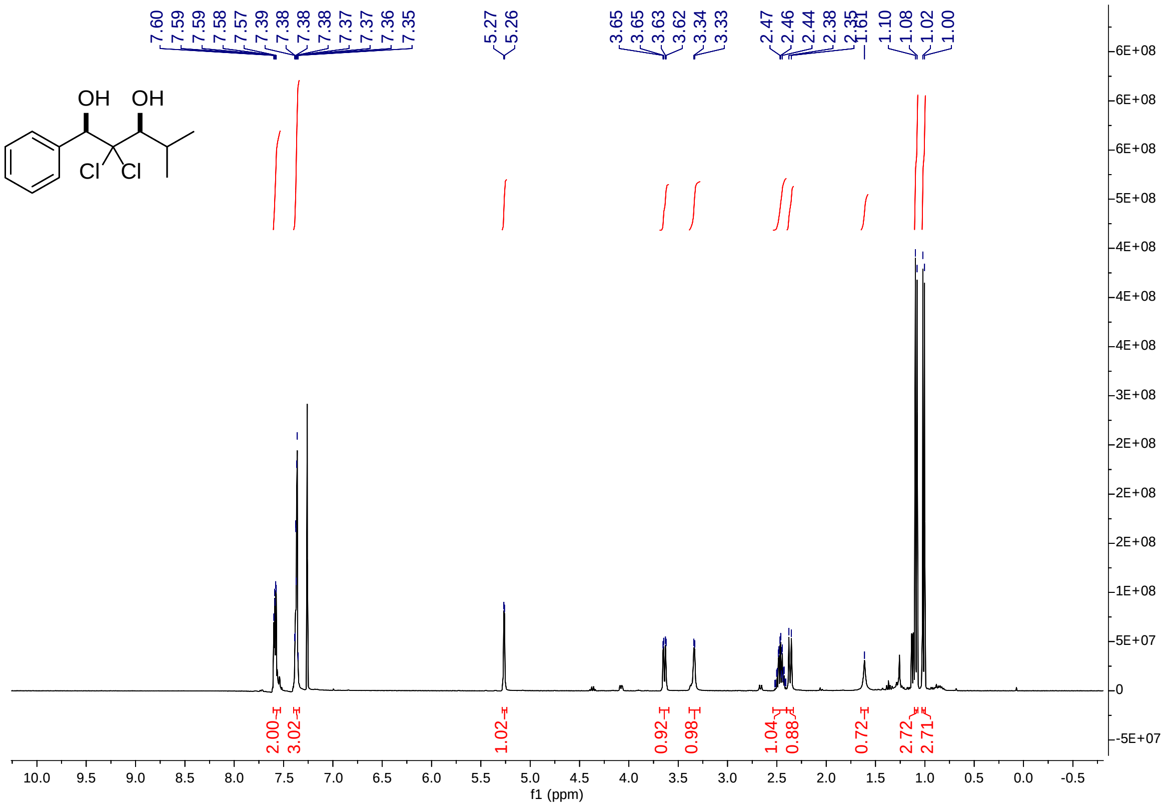

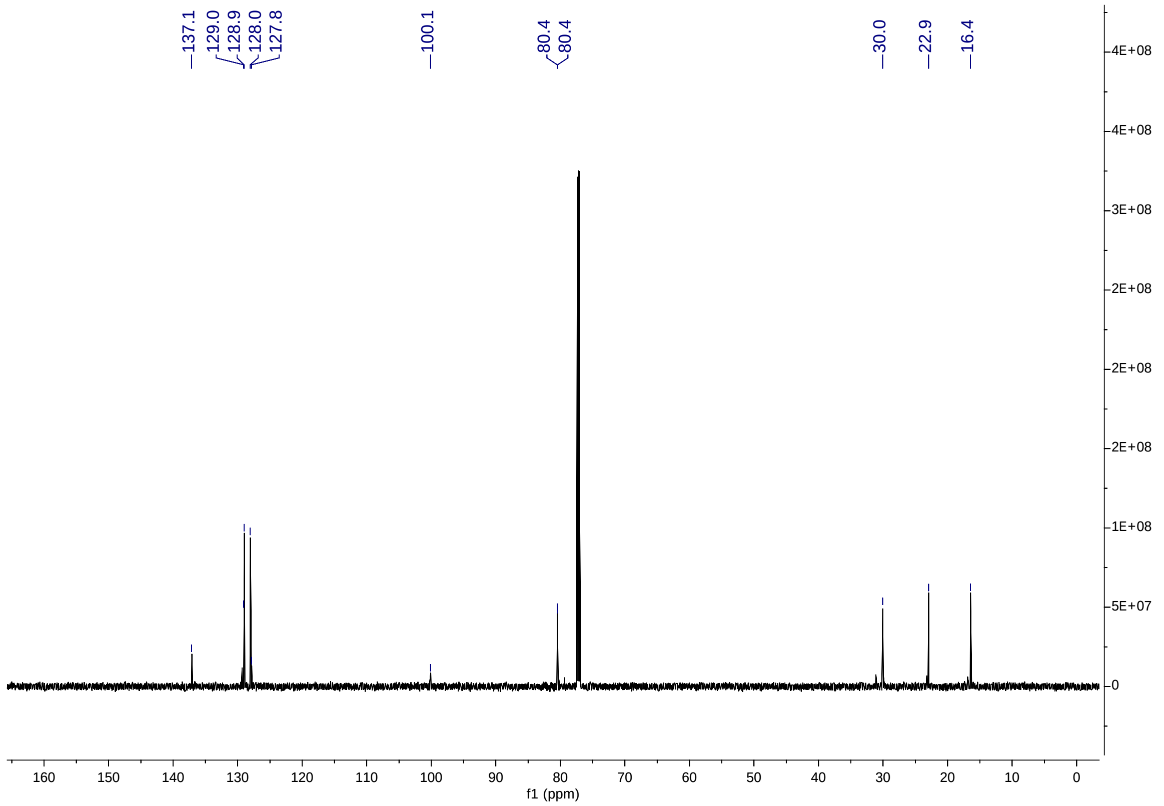

3,3-Dibromo-2-methoxy-2-(*p*-tolyl)tetrahydrofuran (25a)

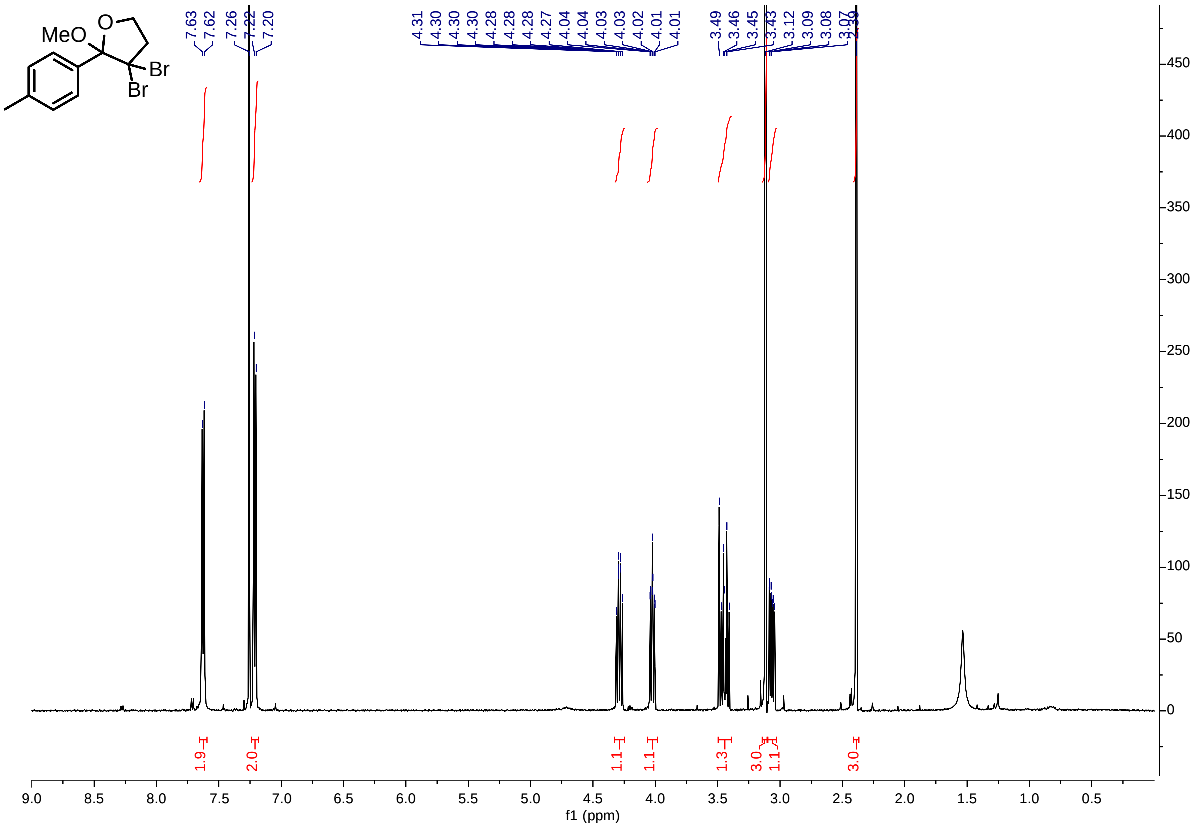

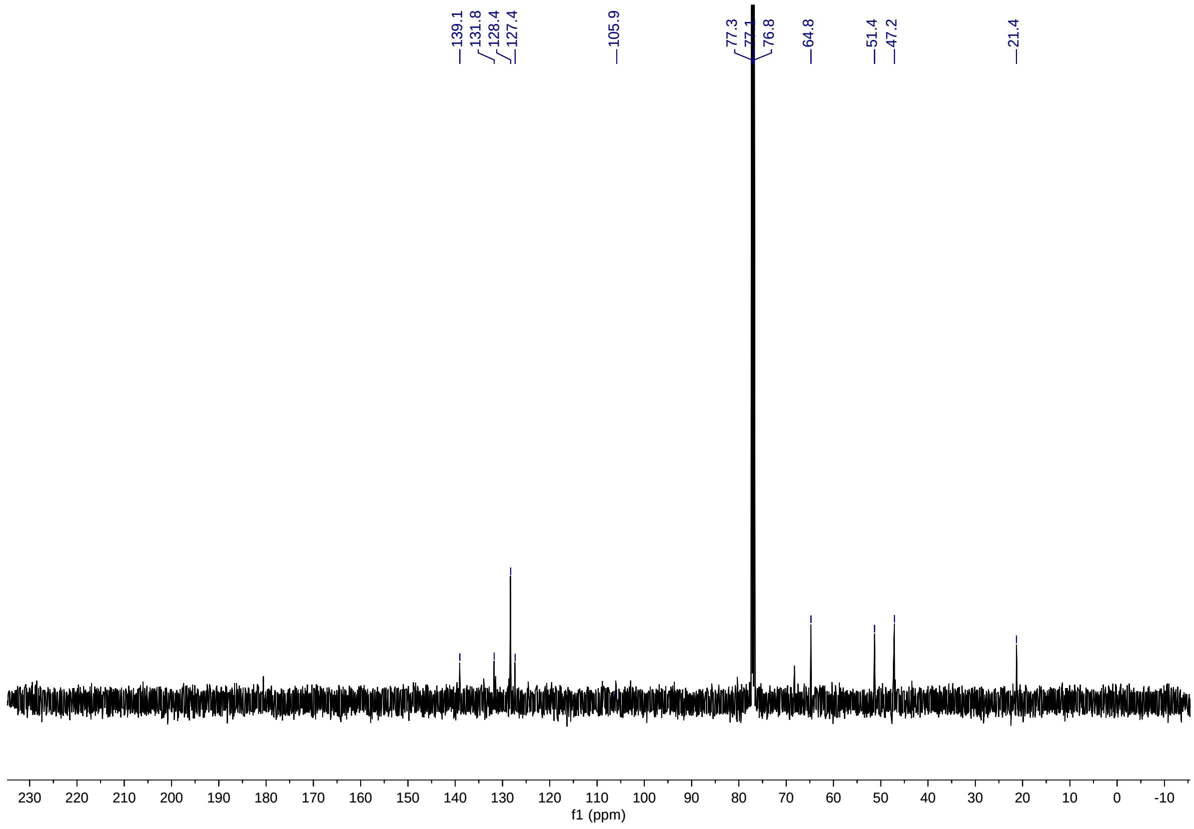

3,3-Dichloro-2-methoxy-2-(*p*-tolyl)tetrahydrofuran (25b)

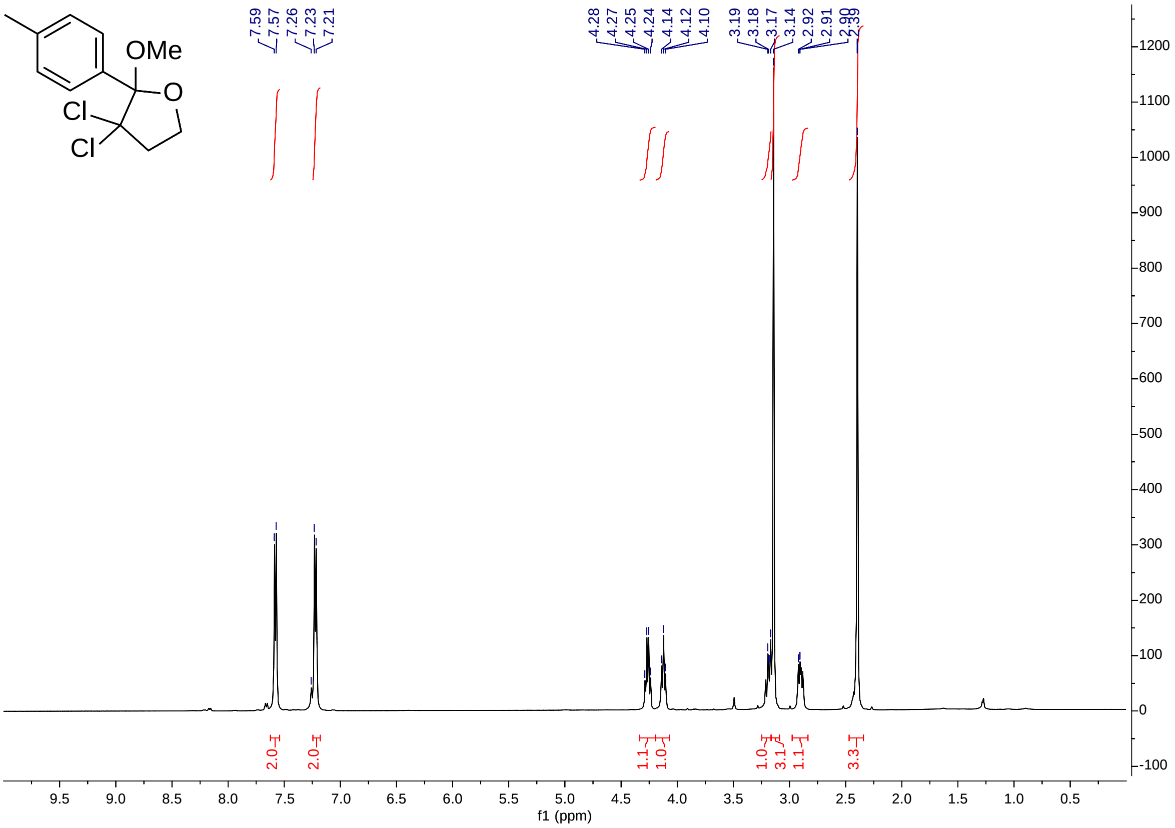

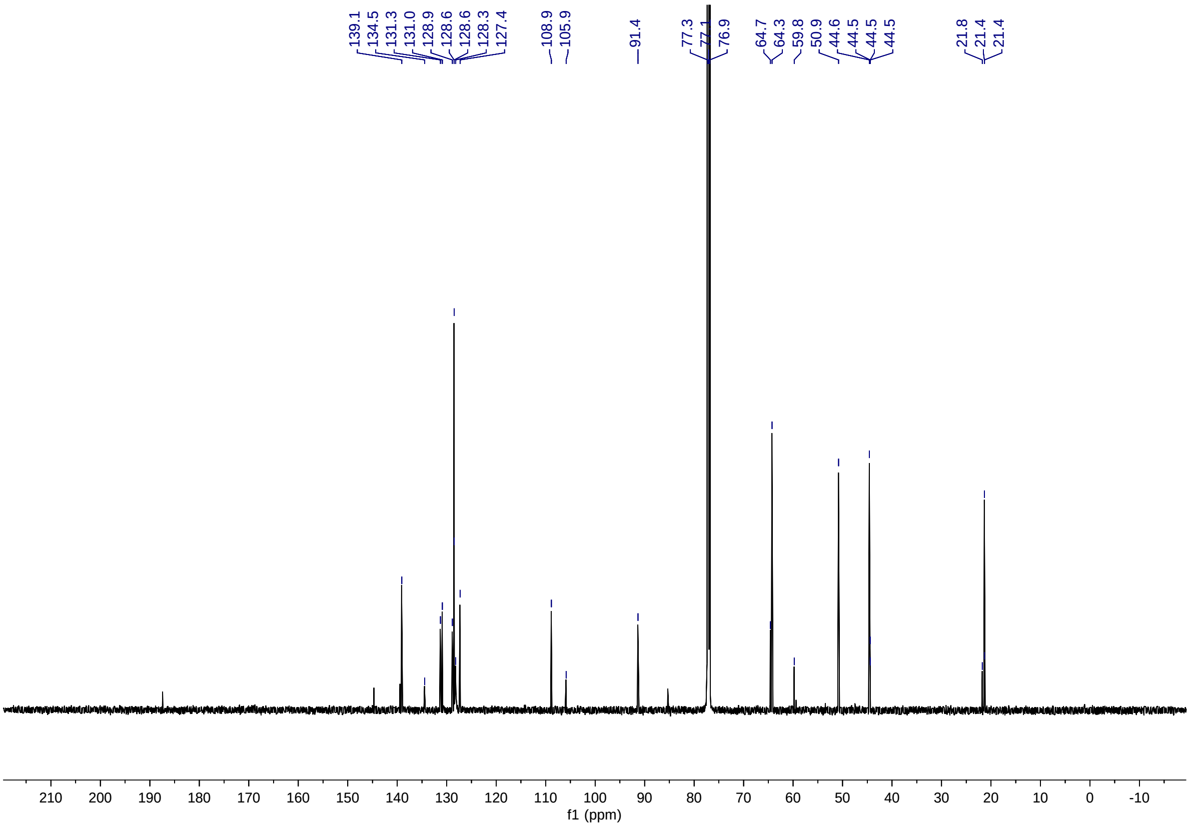

3,3-Dibromo-2-methoxy-5-methyl-2-(*p*-tolyl)tetrahydrofuran (25c)

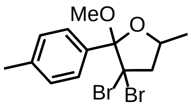

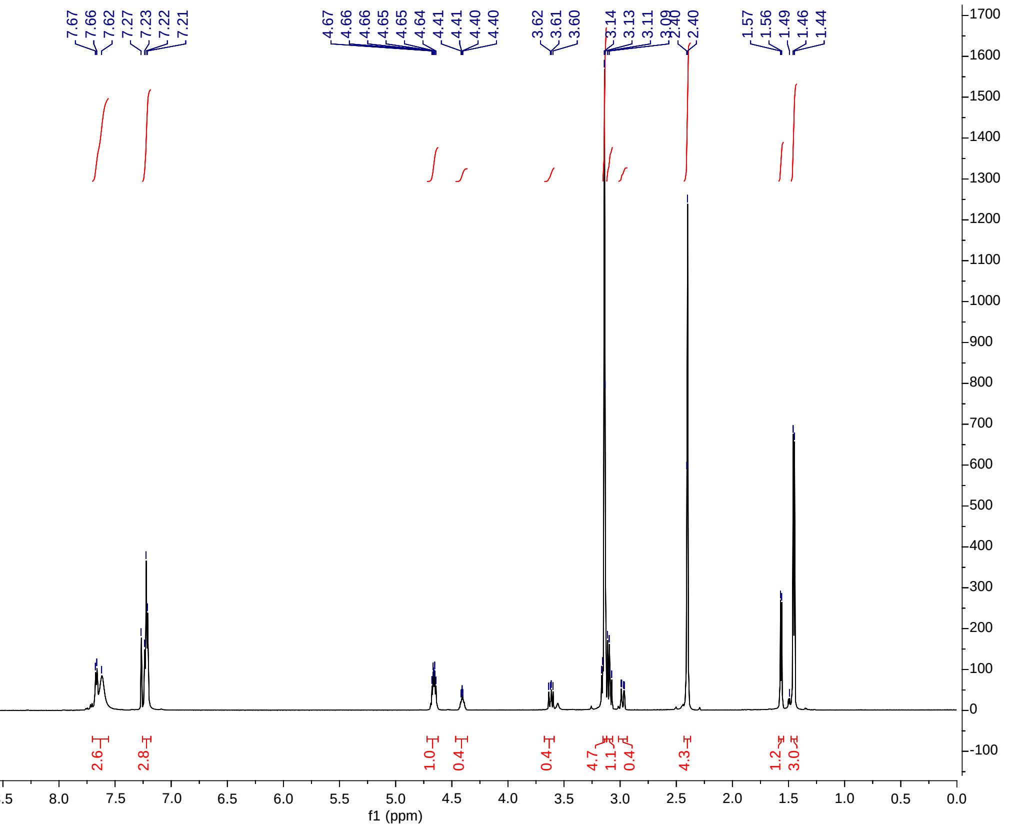

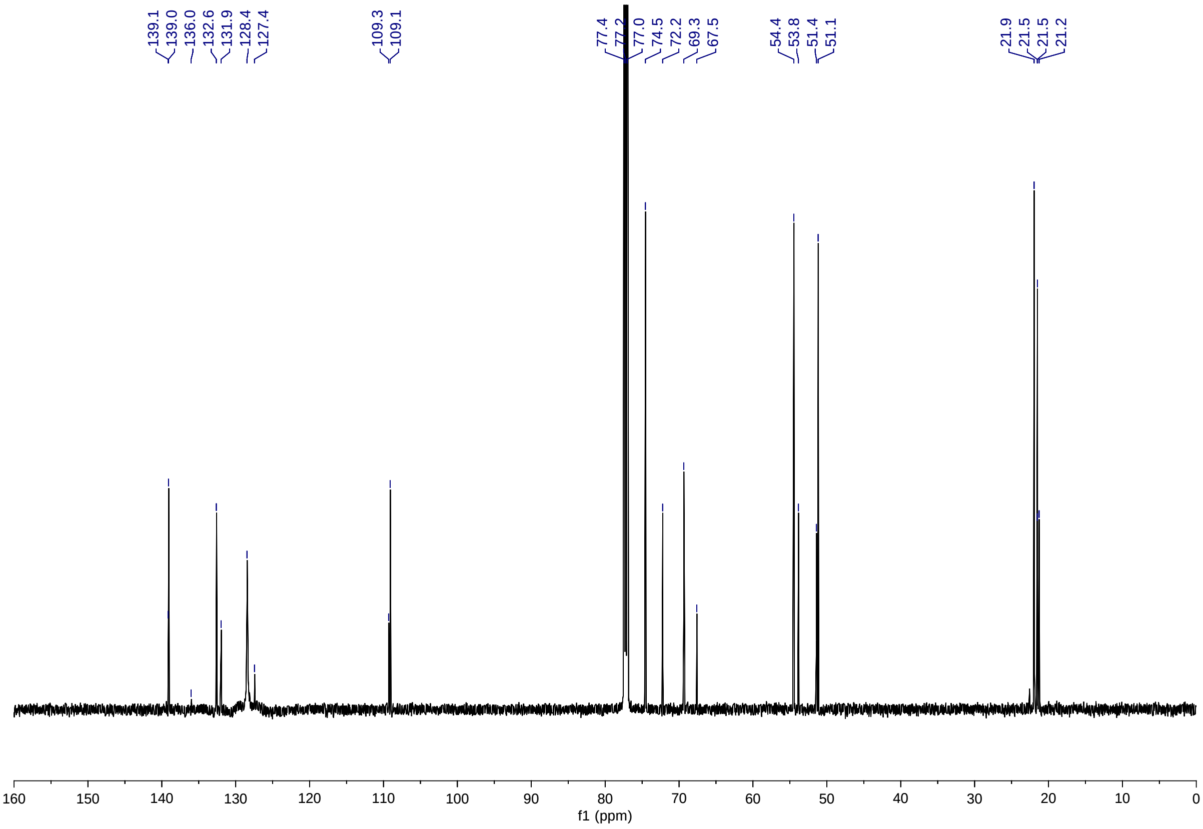

3-Bromo-2-(*p*-tolyl)furan (26a)

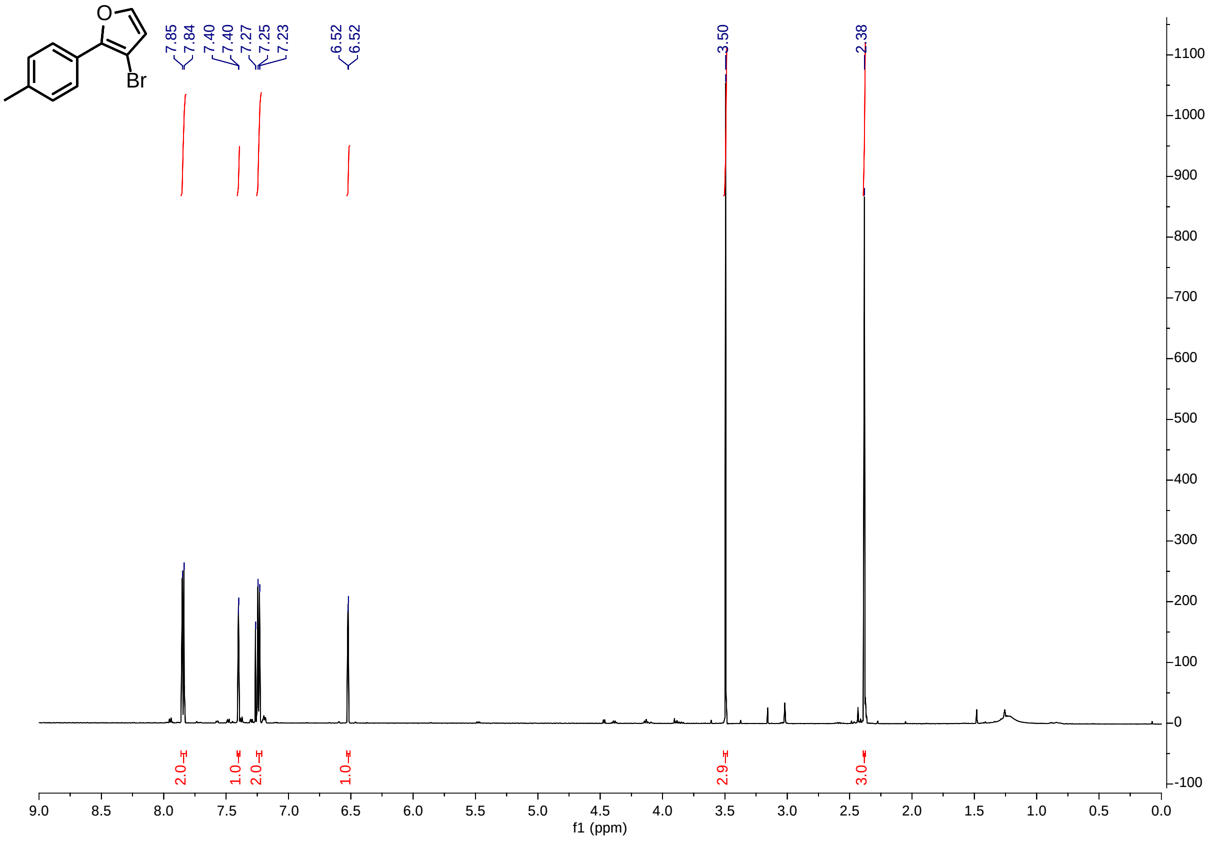

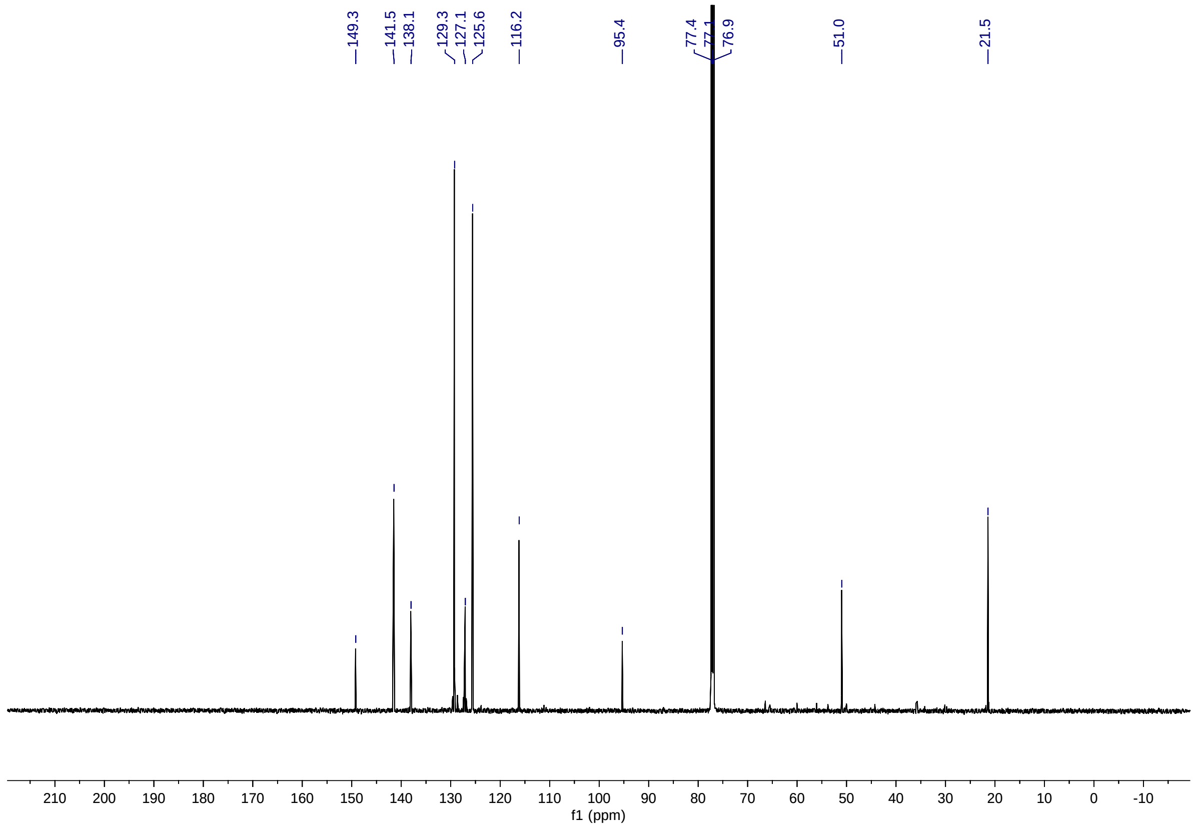

3-Chloro-2-(*p*-tolyl)furan (26b)

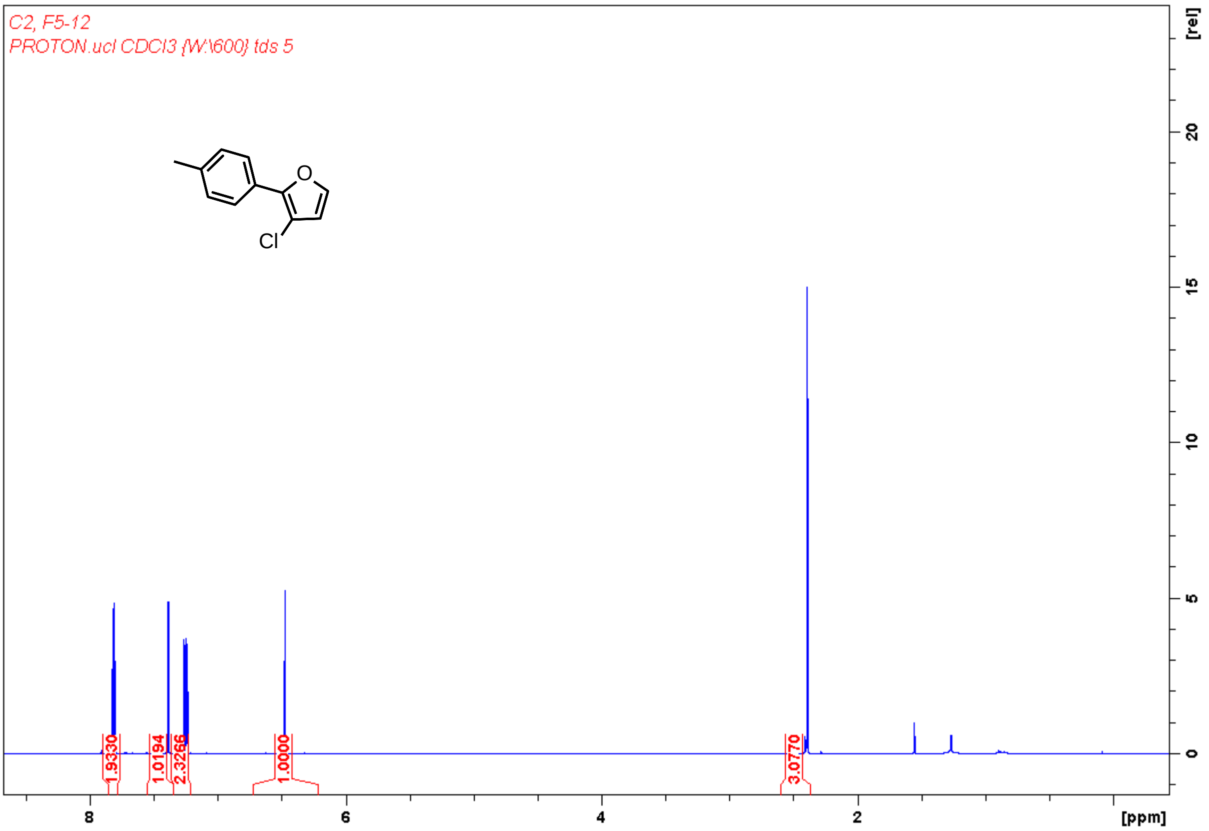

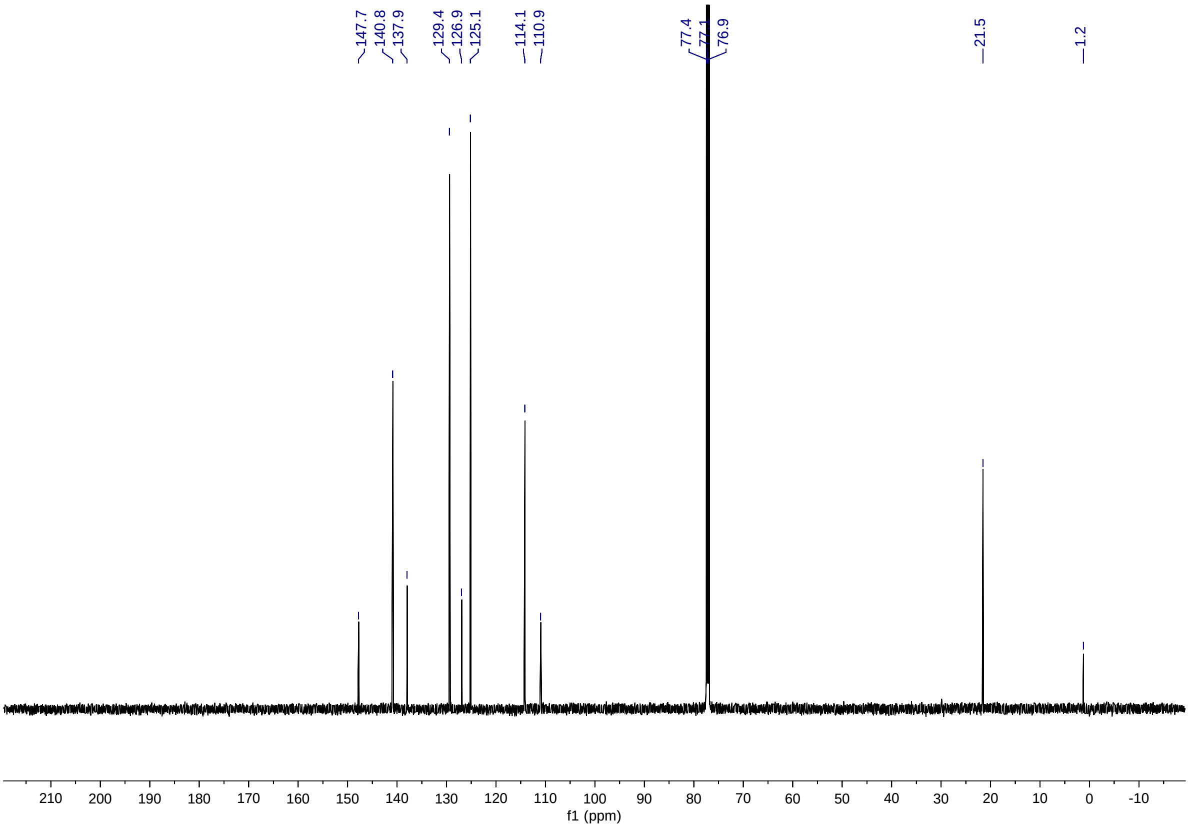

3-Bromo-5-methyl-2-(*p*-tolyl)furan (26c)

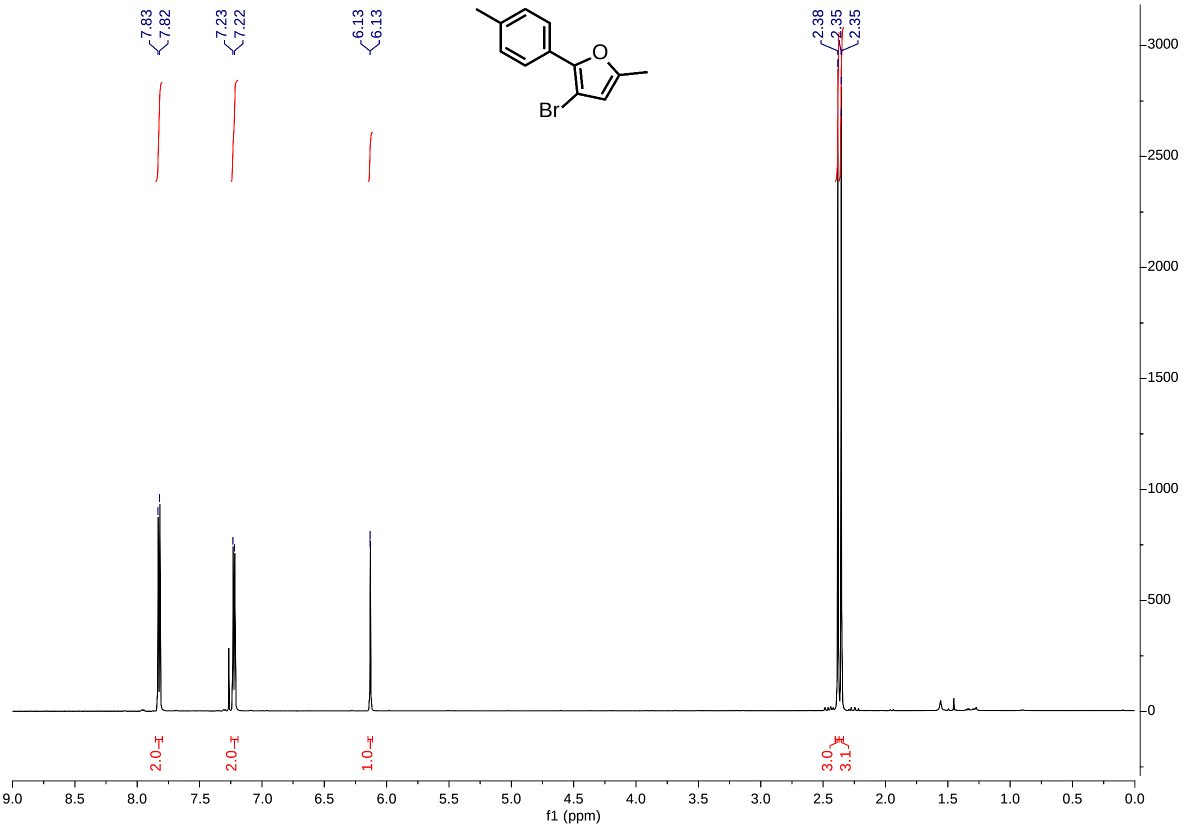

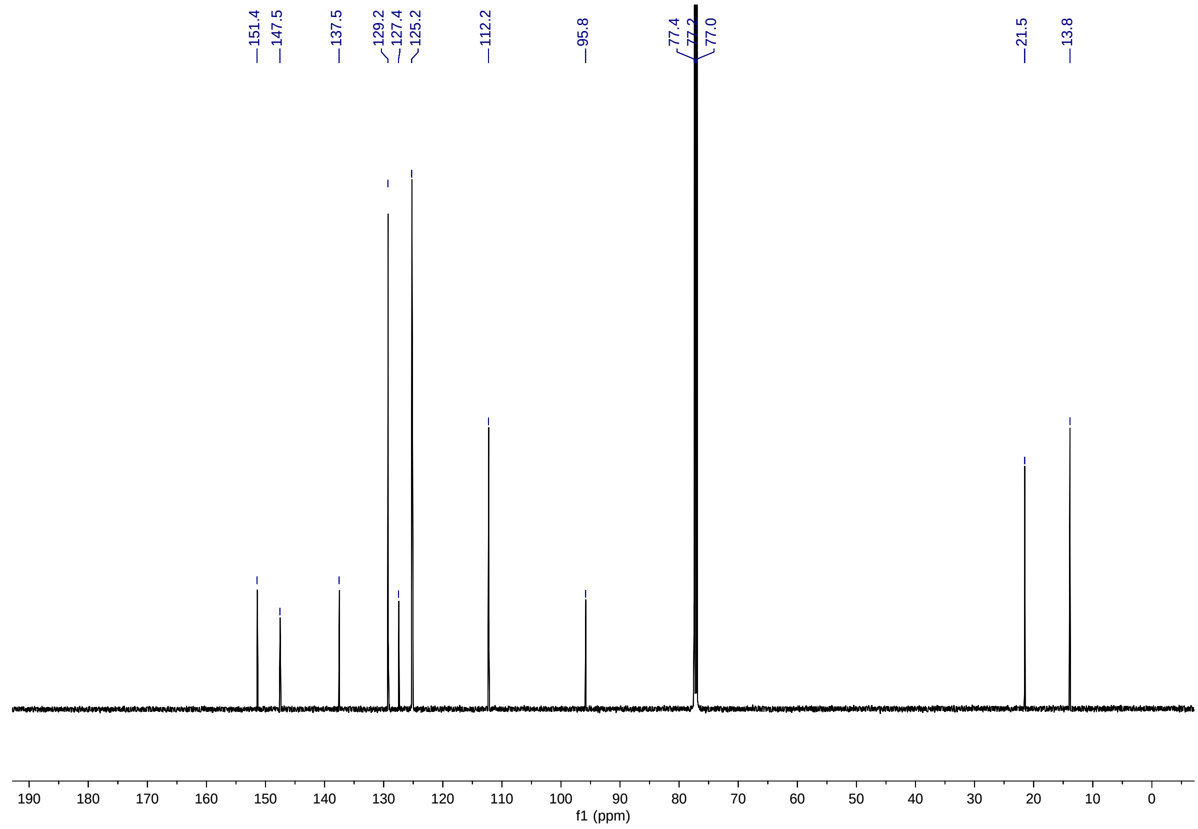

(3a*S*,4*S*,7*S*,7a*R*)-5-Bromo-2,7-dimethyl-4-(*p*-tolyl)-3a,4,7,7a-tetrahydro-1*H*-4,7-epoxyisoindole-1,3(2*H*)-dione (*endo*-27)

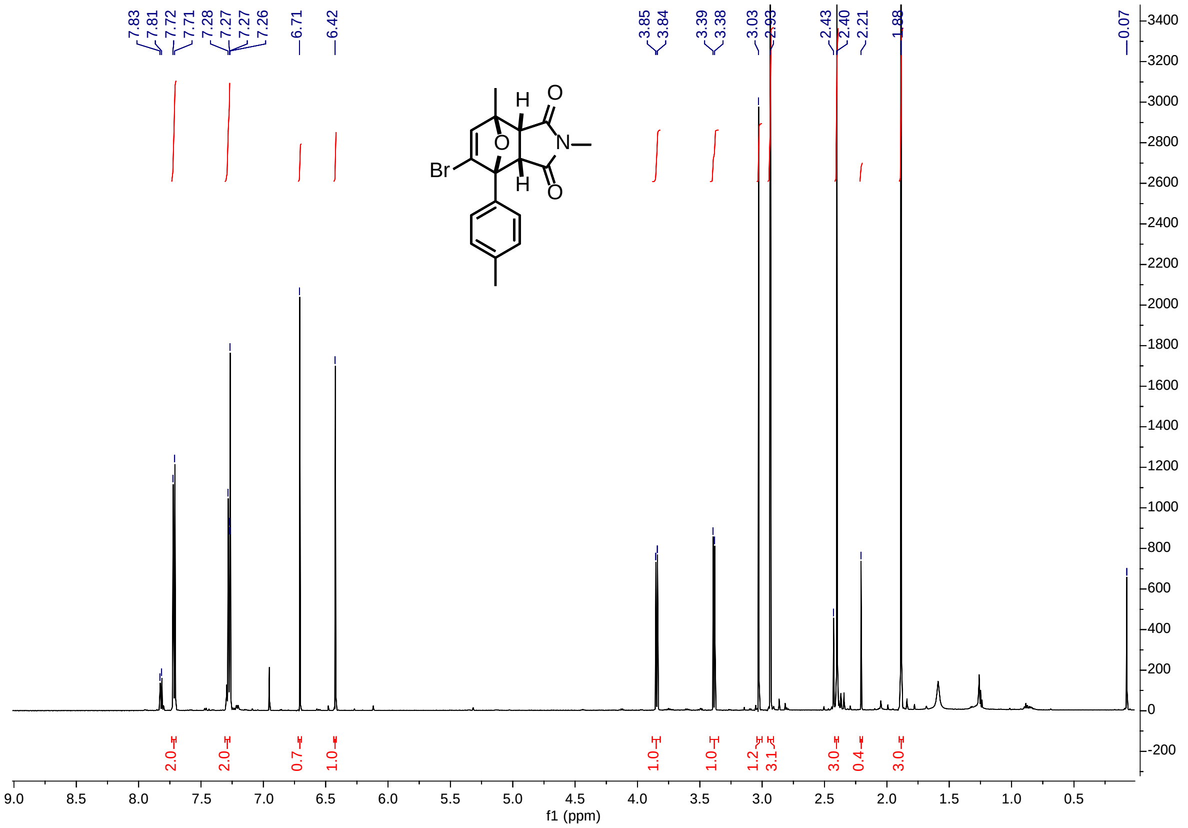

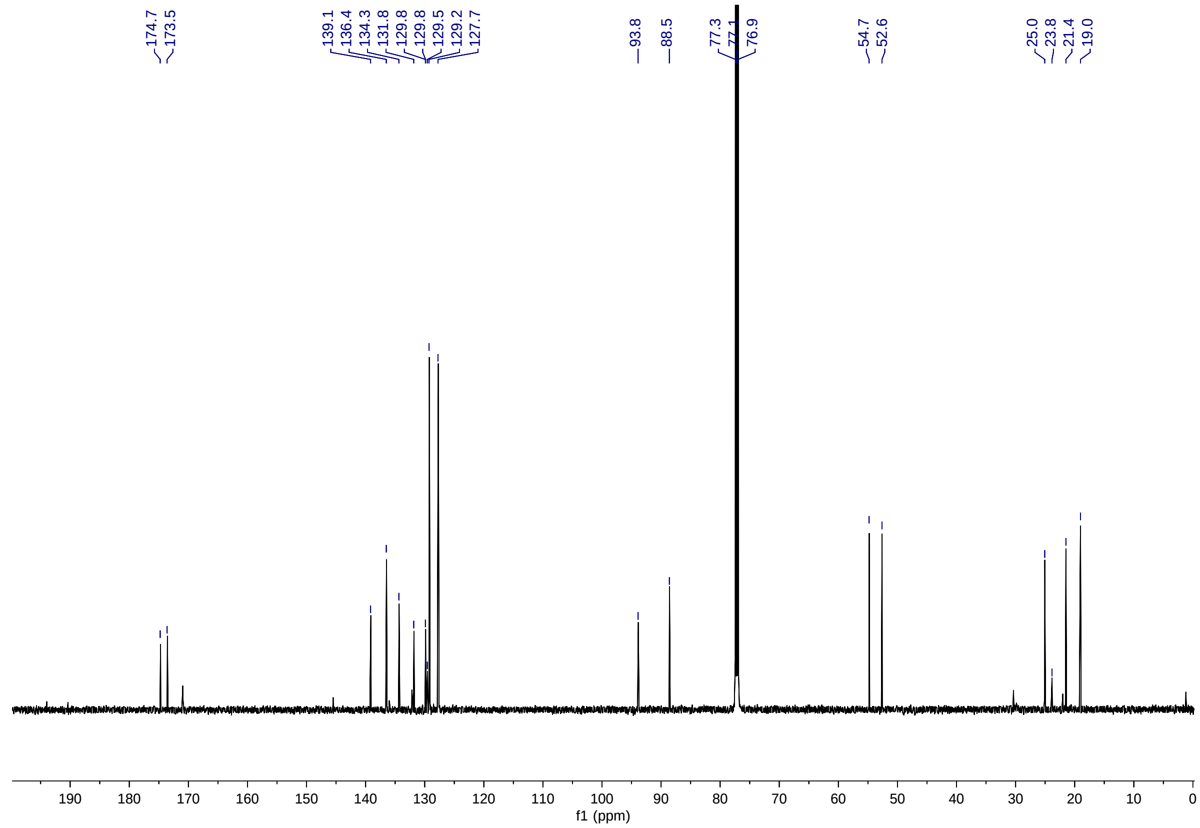

(3a*S*,4*S*,7*S*,7a*R*)-5-Chloro-2-methyl-4-(*p*-tolyl)-3a,4,7,7a-tetrahydro-1*H*-4,7-epoxyisoindole-1,3(2*H*)-dione (*endo*-28)

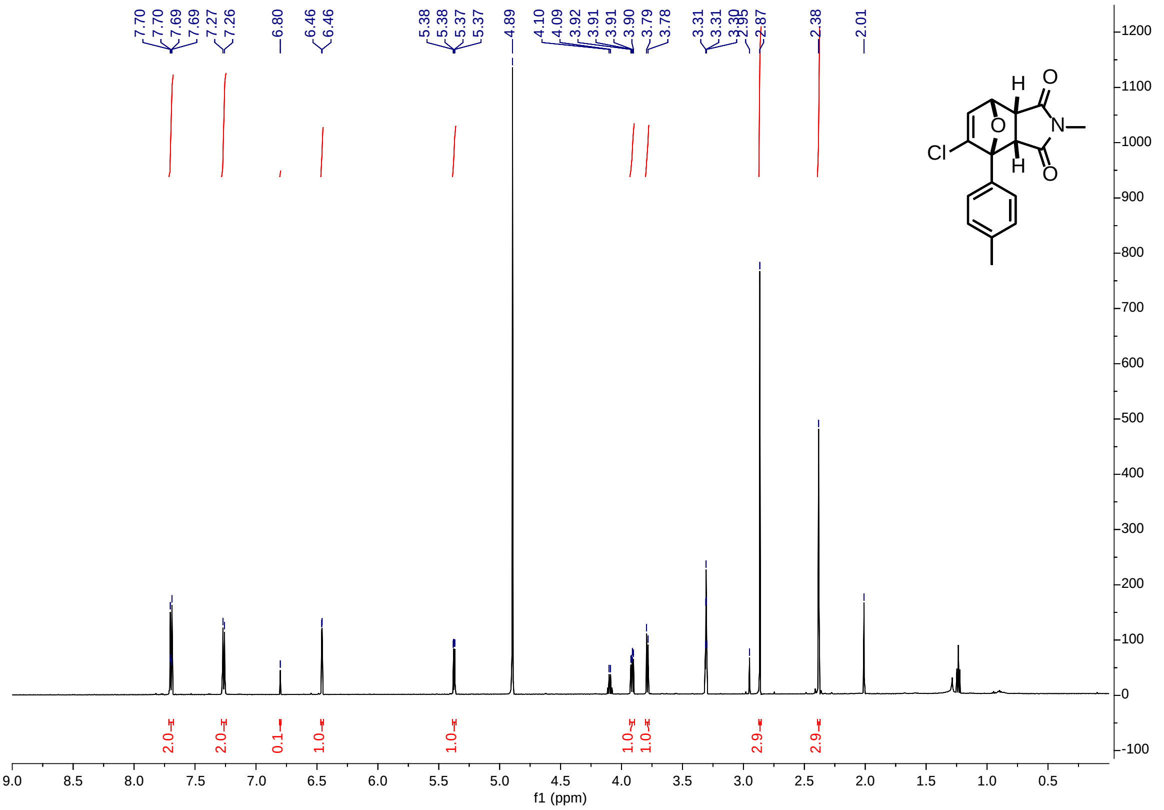

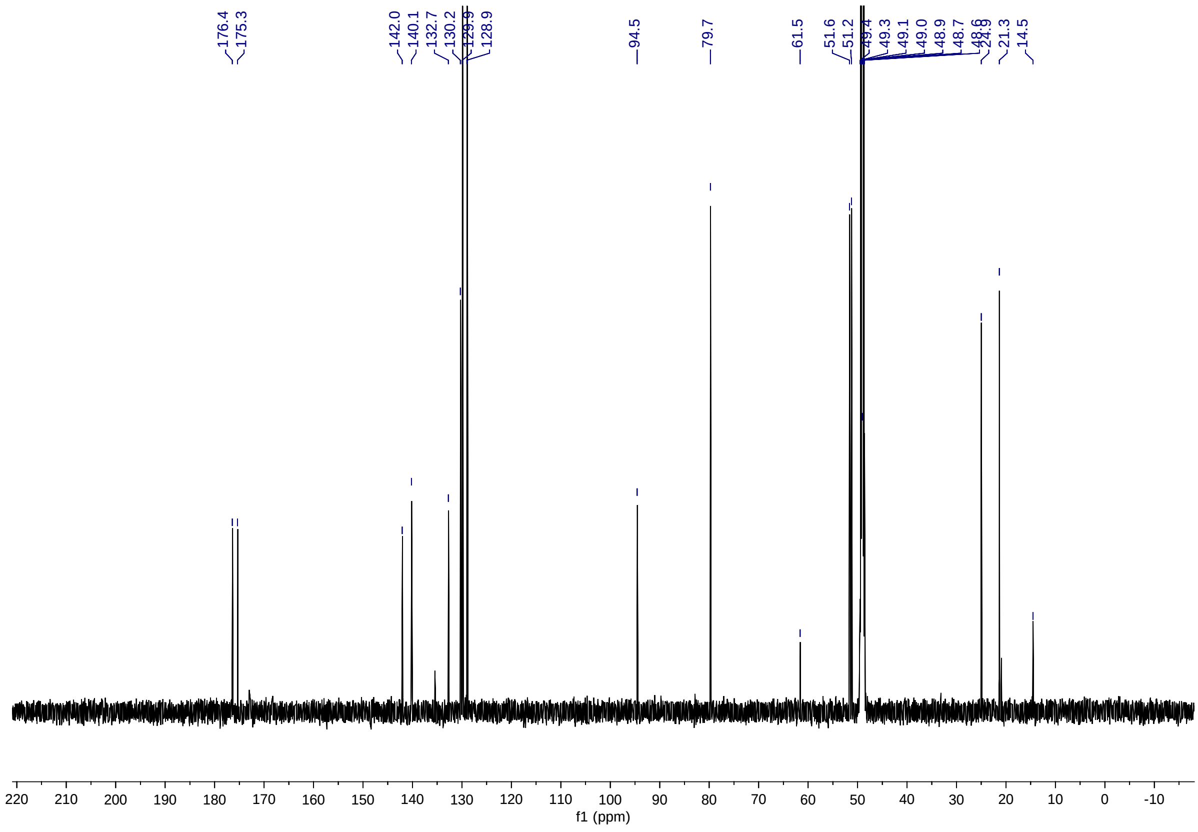

(3a*R*,4*S*,7*S*,7a*S*)-5-Chloro-2-methyl-4-(*p*-tolyl)-3a,4,7,7a-tetrahydro-1*H*-4,7-epoxyisoindole-1,3(2*H*)-dione (*exo*-28)

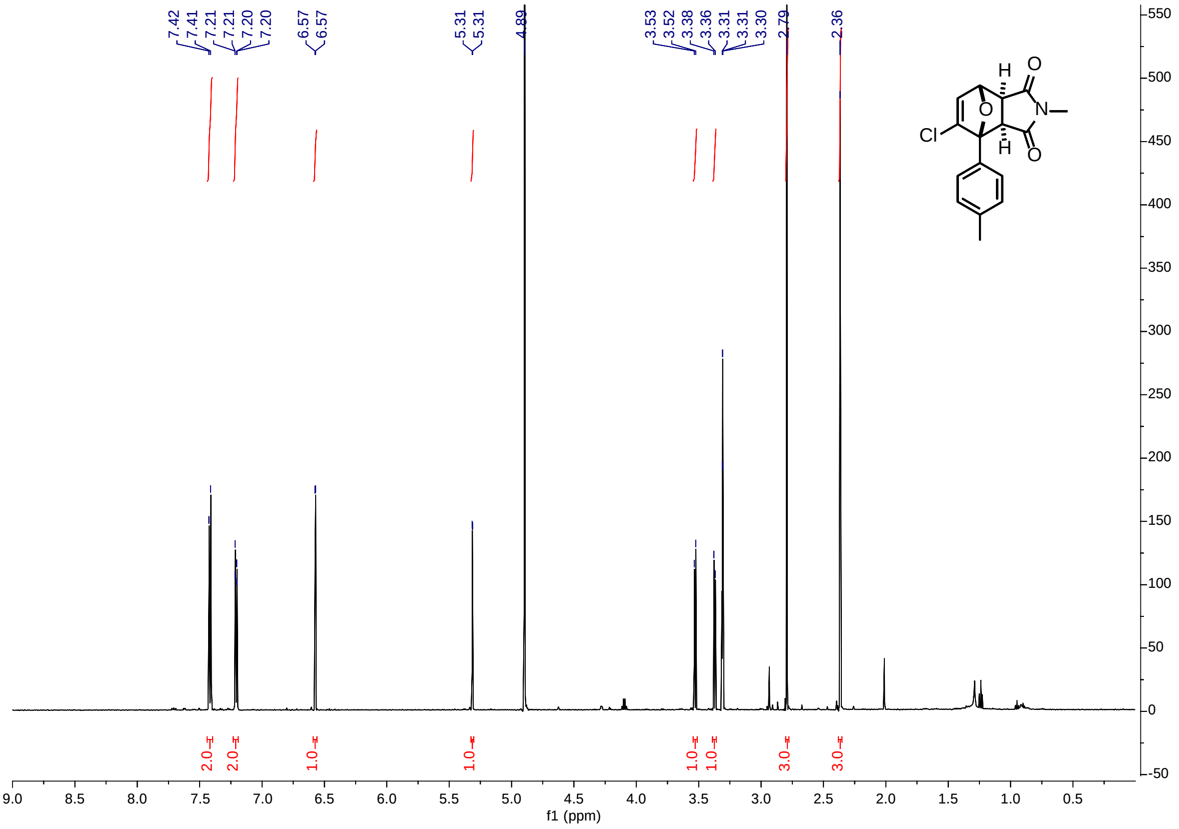

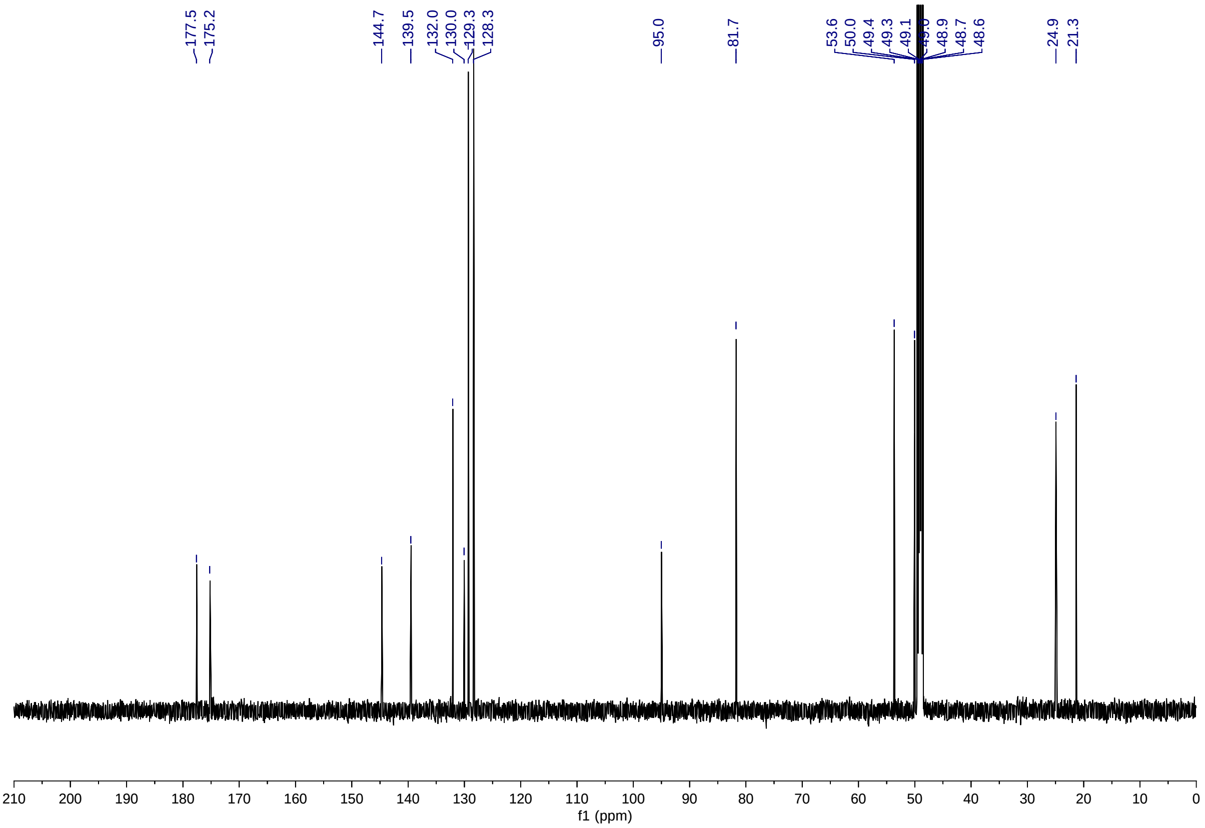

(*E*)-2-(1-Bromo-2-hydroxy-2-(*p*-tolyl)ethylidene)cyclobutan-1-ol (29a)

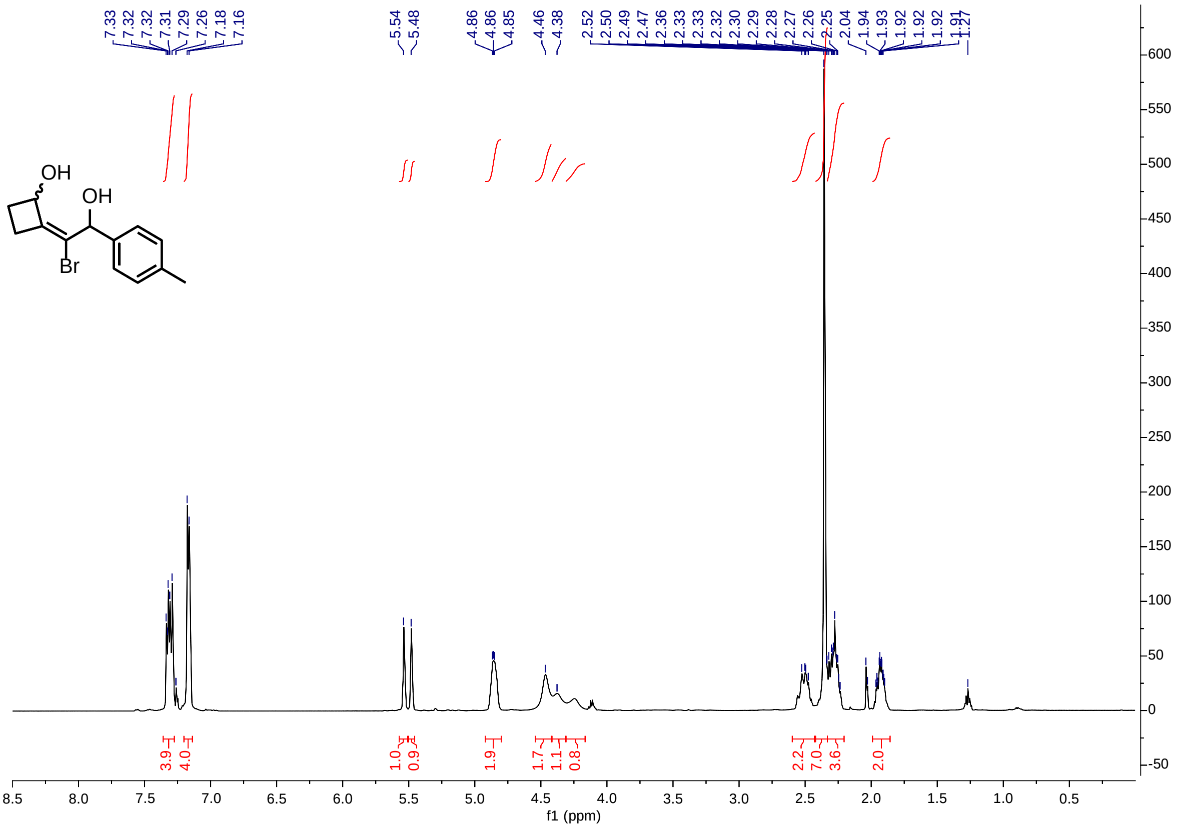

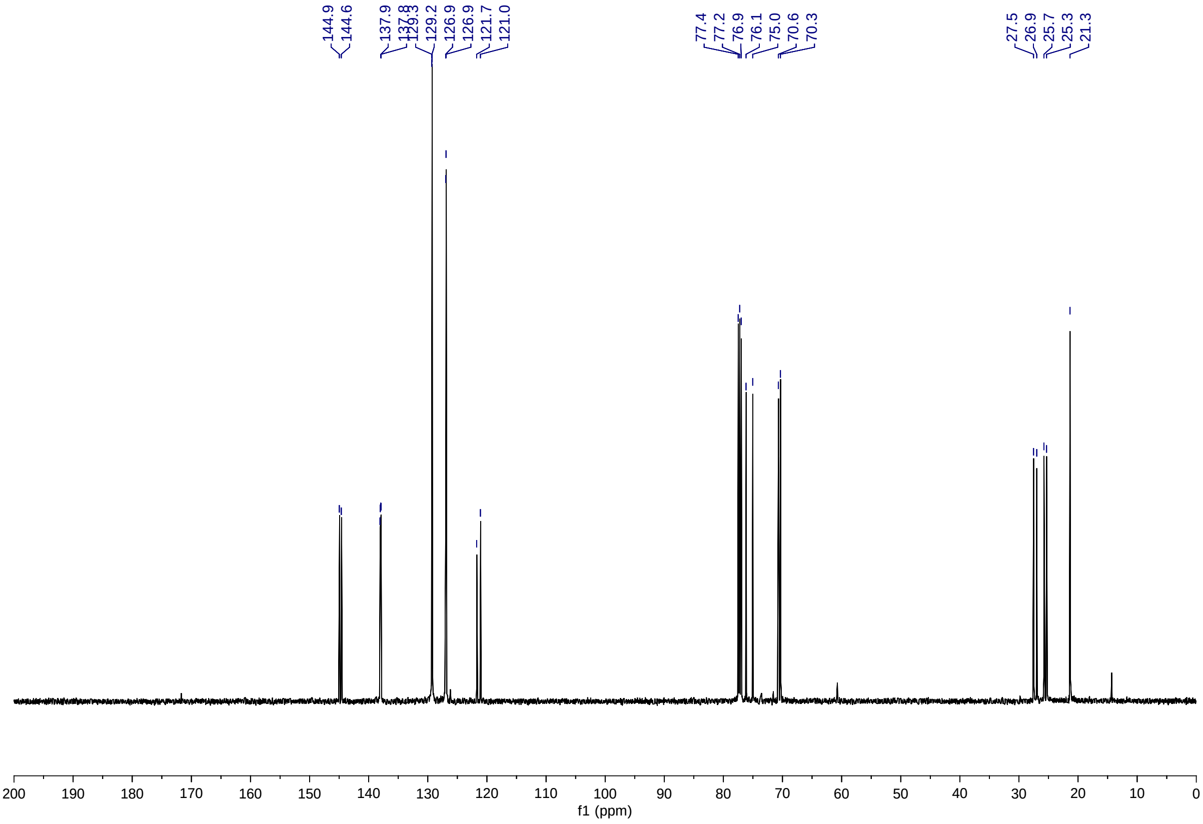

(*E*)-2-(1-Bromo-2-hydroxy-2-(*p*-tolyl)ethylidene)cyclobutan-1-ol (29b)

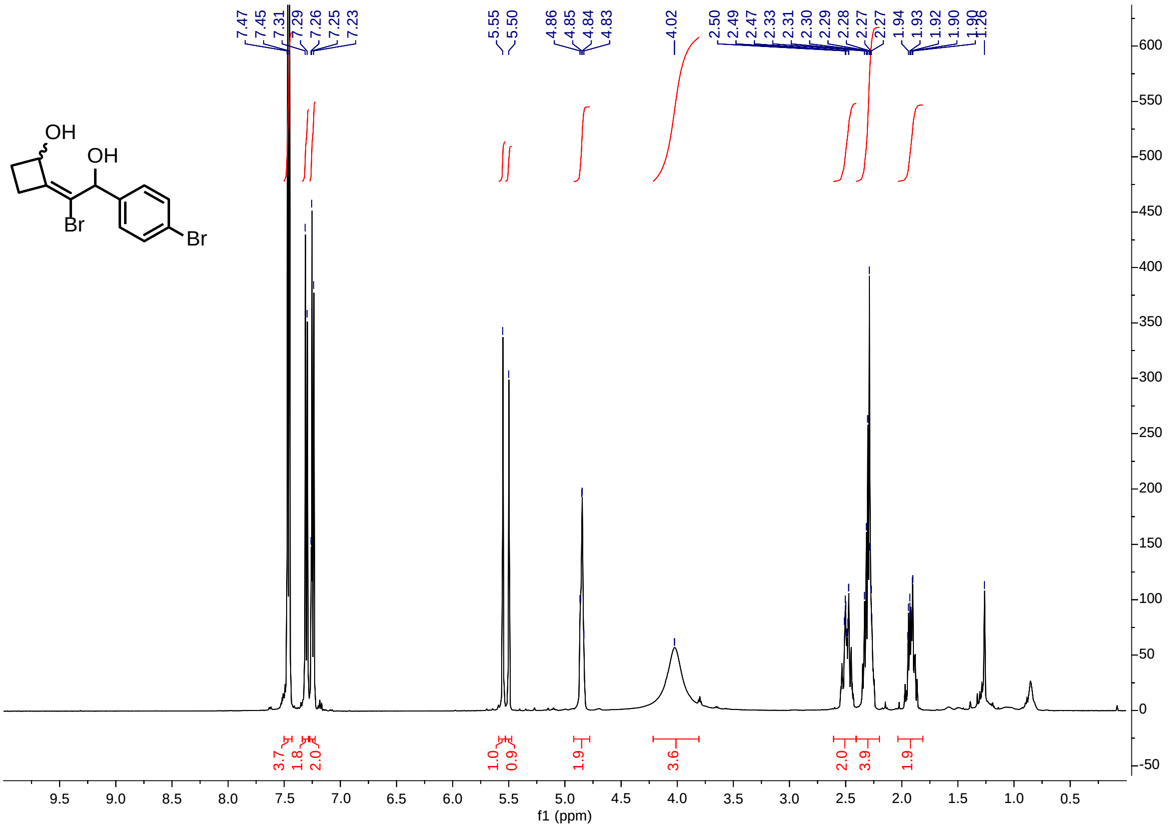

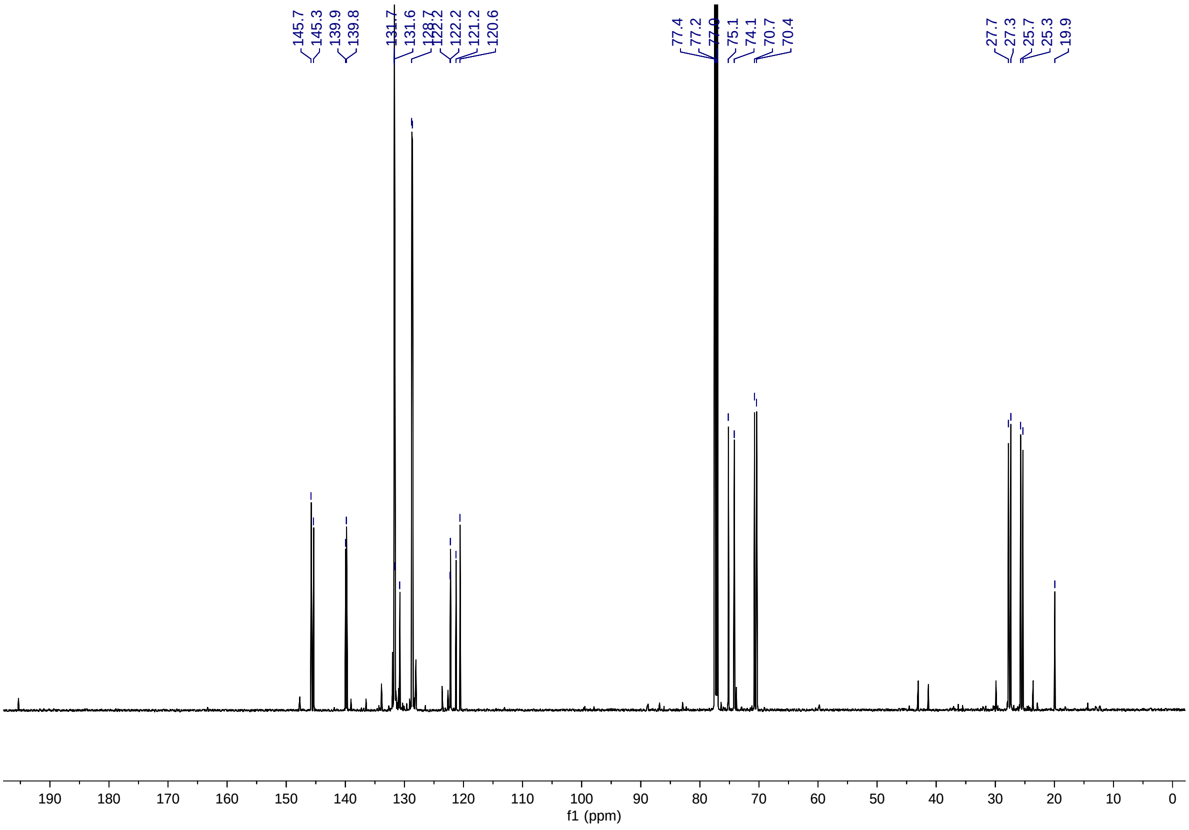

References

[1] J. M. D'Oyley, A. E. Aliev, T. D. Sheppard, *Angew. Chem. Int. Ed.* **2014,** *53*, 10747

[2] M. N. Pennell, M. P. Kyle, S. M. Gibson, L. Male, P. G. Turner, R. S. Grainger, T. D. Sheppard, *Adv. Synth. Catal.* **2016**, *358*, 1519.

[3] M. N. Pennell, M. G. Unthank, P. Turner, T. D. Sheppard, *J. Org. Chem.* **2011**, 76, 1479

[4] M. N. Pennell, P. G. Turner and T. D. Sheppard, *Chem. Eur. J.* **2012**, *18*, 4748.

[5] P. Liu, C.-L. Deng, X. Lei, G. Lin, *Eur. J. Org. Chem.* **2011**, 7308.

[6] J. Mao, Z. Bao, J. Guo, S. Ji; *Tetrahedron*, **2008**, 64, 9901.

[7] Yu, Y.; Yang, W.; Pflästerer, D.; Hashmi, A. S. K. *Angew. Chem. Int. Ed.* **2014**, *53*,1144.

[8] R. D. Grigg, J. W. Rigoli, S. D. Pearce, J. M. Schomaker, *Org. Lett.* **2012**, *14*, 280.

[9] D. S. Stephenson, G. Binsch, *J. Magn. Reson.* **1980**, *37*, 395.

[10] *gNMR*, Version 5.0.6; NMR Simulation Program, Budzelaar, P. H. M., **2006**.

[11] E. Cances, B. Mennucci, *J. Math. Chem.* **1998**, *23*, 309.

[12] *Gaussian 09*. Revision D.01. M. J. Frisch, G. W. Trucks, H. B. Schlegel, G. E. Scuseria, M.A. Robb, J. R. Cheeseman, G. Scalmani, V. Barone, B. Mennucci, G. A. Petersson, H. Nakatsuji, M. Caricato, X. Li, H. P. Hratchian, A. F. Izmaylov, J. Bloino, G. Zheng, J. L. Sonnenberg, M. Hada, M. Ehara, K. Toyota, R. Fukuda, J. Hasegawa, M. Ishida, T. Nakajima, Y. Honda, O. Kitao, H. Nakai, T. Vreven, J. A. Montgomery, Jr., J. E. Peralta, F. Ogliaro, M. Bearpark, J. J. Heyd, E. Brothers, K. N. Kudin, V. N. Staroverov, R. Kobayashi, J. Normand, K. Raghavachari, A. Rendell, J. C. Burant, S. S. Iyengar, J. Tomasi, M. Cossi, N. Rega, J. M. Millam, M. Klene, J. E. Knox, J. B. Cross, V. Bakken, C. Adamo, J. Jaramillo, R. Gomperts, R. E. Stratmann, O. Yazyev, A. J. Austin, R. Cammi, C. Pomelli, J. W. Ochterski, R. L. Martin, K. Morokuma, V. G. Zakrzewski, G. A. Voth, P. Salvador, J. J. Dannenberg, S. Dapprich, A. D. Daniels, Ö. Farkas, J. B. Foresman, J. V. Ortiz, J. Cioslowski, and D. J. Fox, Gaussian, Inc., Wallingford CT, **2013**.

[13] A. E. Aliev, D. Courtier-Murias, *J. Phys. Chem. B.* **2007**, *111*, 14034-14042.
